# Efficacy and safety of vitamin D in the treatment of asthma: an overview of systematic reviews and meta-analyses

**DOI:** 10.3389/fmed.2026.1783005

**Published:** 2026-05-26

**Authors:** Yongxiu Liu, Jiaheng Lu, Donghao Li, Zhuang Wang, Chi Zhang, Yuguo Li, Shuang Liang, Haimiao Yang, Lei Gao

**Affiliations:** 1 College of Traditional Chinese Medicine, Changchun University of Chinese Medicine, Changchun, Jilin, China; 2 College of Basic Medicine, Changchun University of Chinese Medicine, Changchun, Jilin, China; 3 Clinic of the 22 nd Haidian Vacation Home for Retired Cadres, Beijing Garrison Command, Beijing, China; 4 Affiliated Hospital of Changchun University of Chinese Medicine, Changchun, Jilin, China

**Keywords:** asthma, efficacy, overview of systematic reviews and meta-analyses, safety, vitamin D

## Abstract

**Background:**

Asthma is a common chronic airway disease that can cause recurrent symptoms, acute exacerbations, impaired quality of life, and substantial healthcare burden. Vitamin D has been proposed as a potential add-on therapy because of its roles in immune regulation and respiratory defense, but its clinical benefits in asthma remain uncertain.

**Objective:**

This umbrella review aimed to evaluate the efficacy and safety of vitamin D supplementation as an adjunctive treatment for asthma and to assess the credibility of the existing evidence.

**Methods:**

We systematically searched PubMed, Cochrane Library, Web of Science, Embase, CNKI, WANFANG, VIP, and CBM from inception to November 26, 2025. Published systematic reviews and meta-analyses evaluating vitamin D supplementation for asthma were included. We assessed methodological quality, risk of bias, reporting quality, certainty of evidence, and overlap among primary studies using standard appraisal tools.

**Results:**

21 systematic reviews and meta-analyses were included. Overlap among primary studies was substantial. Vitamin D supplementation was associated with a lower risk of asthma exacerbations, but its effects on asthma control, lung function, and inflammatory outcomes were limited or inconsistent. No significant increase in adverse events was observed. The certainty of evidence varied across outcomes, and many findings were supported by low or very low certainty evidence.

**Conclusion:**

Vitamin D supplementation may reduce asthma exacerbations and appears to be safe as an adjunctive treatment, but current evidence does not consistently support improvements in daily symptom control, lung function, or inflammatory outcomes. These findings should be interpreted cautiously because of heterogeneity, evidence uncertainty, and overlap among primary studies. Future studies should identify patients most likely to benefit and clarify optimal dose, duration, and biomarker-guided supplementation strategies.

**Systematic review registration:**

https://www.crd.york.ac.uk/PROSPERO/view/CRD420251239612, identifier: CRD420251239612.

## Introduction

Asthma, a chronic inflammatory airway disorder, affects an estimated 260 million people globally, with its prevalence increasing annually. The condition is clinically characterized by symptoms including wheezing, cough, chest tightness, and dyspnea. These manifestations not only interfere with daily activities and sleep but may also precipitate acute exacerbations, which can be life-threatening (1, 2). Current first-line therapies for asthma, such as inhaled corticosteroids, β_2_-agonists, and leukotriene receptor antagonists, are effective in symptom management but do not provide a cure, frequently resulting in disease recurrence (3, 4). There is consequently a pressing need for therapies that address the core pathophysiology of asthma. Apart from the acute risks of severe exacerbations, asthma contributes to a sustained decline in patients' quality of life, characterized by persistent symptoms like chronic breathlessness and restricted activity. It also entails substantial economic costs and considerable psychological distress, imposing a multifaceted long-term burden on individuals (2, 5).

Vitamin D has been proposed as a potential adjunctive treatment for asthma because of its roles in immune regulation and airway protection (6, 7). Previous studies suggest that vitamin D may influence asthma-related outcomes by enhancing innate airway defense, modulating adaptive inflammatory responses, and supporting airway epithelial barrier integrity (8–11). In addition, vitamin D has been linked to reduced susceptibility to respiratory infections, attenuation of airway inflammation, and potential modulation of airway remodeling, all of which are relevant to asthma exacerbation and long-term disease control (6, 7, 11, 12). However, mechanistic plausibility does not necessarily translate into consistent clinical benefit. Existing systematic reviews and meta-analyses have reported inconsistent findings regarding asthma exacerbations, symptom control, lung function, inflammatory biomarkers, and safety outcomes. Therefore, a higher-level synthesis of the existing evidence is needed to critically examine its reliability, consistency, and certainty. In addition, the optimal supplementation dose, whether the benefits are mainly restricted to individuals with baseline vitamin D deficiency, and the long-term efficacy and safety of vitamin D supplementation remain uncertain and require further validation in high-quality evidence-based studies (13, 14). Therefore, the specific aim of this umbrella review was to evaluate the efficacy and safety of vitamin D supplementation as an adjunctive treatment for asthma by synthesizing evidence from published systematic reviews and meta-analyses. The core research question was whether vitamin D supplementation improves asthma exacerbations, symptom control, lung function, inflammatory biomarkers, and safety outcomes. In addition, this study assessed the methodological quality, risk of bias, reporting quality, certainty of evidence, and overlap among primary studies to determine the credibility and reliability of the existing evidence.

## Materials and methods

### Protocol registration

The study protocol was registered on PROSPERO (registration number: CRD420251239612) prior to study initiation. The registration was completed before the commencement of database searches, and the search strategy, inclusion criteria, and analytical methods remained consistent with the registered protocol.

### Study design

This study was designed as an umbrella review, also known as an overview of systematic reviews. It re-evaluated published systematic reviews and meta-analyses on vitamin D supplementation for asthma and further conducted a re-analysis of meta-analytic findings to improve the overall quality, consistency, and reliability of the evidence.

### Inclusion criteria

(1) Population: Children and/or adults diagnosed with asthma, regardless of age, disease severity, phenotype, or baseline vitamin D levels.
(2) Intervention: Vitamin D supplementation, administered either alone or in combination with standard asthma treatment, with no restrictions on formulation, dosage, route of administration, or treatment duration.
(3) Comparison: Placebo, no vitamin D supplementation, or standard asthma treatment alone.
(4) Outcomes: Asthma exacerbations, asthma control level (e.g., ACT score), lung function parameters (e.g., FEV_1_, FVC, FeNO), serum 25(OH)D levels, inflammatory or immune-related biomarkers, and adverse events.
(5) Study design: Systematic reviews and meta-analyses (SRs/MAs) evaluating the efficacy and/or safety of vitamin D in the treatment of asthma.

(1) SRs/MAs in which vitamin D served as the primary intervention for asthma; (2) The treatment group received vitamin D supplementation in addition to conventional asthma therapy, while the control group received either the same conventional asthma therapy plus a placebo, no vitamin D supplementation, or conventional asthma therapy alone; (3) Participants and/or investigators were blinded (single-blind, double-blind, or unblinded) to the allocation of vitamin D or placebo; (4) Participants were assigned to the vitamin D or placebo group using either randomized or non-randomized methods.

### Exclusion criteria

Studies were excluded if they met any of the following criteria: (1) duplicate publications; (2) unretrievable full text or studies with incomplete outcome data; (3) review articles, commentaries, or editorials; (4) studies not relevant to the predefined research focus; (5) primary clinical trial reports; (6) animal or preclinical experimental studies; (7) *in vitro* or mechanistic studies.

### Search strategy

We conducted a systematic literature search across eight electronic databases, including PubMed, Embase, Cochrane Library, Web of Science, CNKI, VIP, WANFANG, and CBM, from inception to November 26, 2025, to identify SRs/MAs on vitamin D supplementation for asthma. Search terms and search strategy are as follows (using PubMed as an example): Search: [((Asthmas(MeSH Terms)] OR [(((((((((((Asthma, Bronchial(Title/Abstract)] OR [Bronchial Asthma(Title/Abstract))] OR [Allergic Asthma(Title/Abstract)] OR [Exercise-Induced Bronchospasm(Title/Abstract)] OR [Occupational Asthma(Title/Abstract)] OR [Severe Asthma(Title/Abstract)] OR [Refractory Asthma(Title/Abstract)] OR [Childhood-Onset Asthma(Title/Abstract)] OR (Pediatric Asthma[Title/Abstract])) OR (Adult-Onset Asthma[Title/Abstract])) OR [Cough-Variant Asthma(Title/Abstract)] OR [Eosinophilic Asthma(Title/Abstract))] AND [(Vitamin D(MeSH Terms))] OR [(((((((Ergocalciferols(Title/Abstract))] OR [Cholecalciferol(Title/Abstract)] OR [Calcifediol(Title/Abstract)] OR [Calcitriol(Title/Abstract)] OR [Hydroxycholecalciferols(Title/Abstract)] OR [Dihydroxycholecalciferols(Title/Abstract)] OR [Vitamin D-Binding Protein(Title/Abstract)] OR [1,25-dihydroxyvitamin D(Title/Abstract)))] AND [((((Meta-Analysis(MeSH Terms))] OR [((meta-analysis(Title/Abstract))] OR [metaanalysis(Title/Abstract))] OR [meta analysis(Title/Abstract)] OR [(review, systematic(Title/Abstract))] OR [systematic review(Title/Abstract))] OR [(Systematic Review(MeSH Terms))].

### Literature screening and data extraction

This study was conducted by two researchers trained in systematic review methodology, who independently performed literature searches and screening. Following a predefined search strategy, each researcher conducted searches in designated databases. Subsequently, search results were cross-checked to eliminate duplicate records and supplement omitted information, ensuring comprehensive literature collection. During the literature screening phase, if disagreement arose regarding a study's eligibility for inclusion, the two reviewers first revisited the literature content and inclusion criteria through internal discussion to reach consensus. If consensus could not be reached after discussion, a senior expert with advanced professional qualifications and extensive research experience was invited to serve as an arbitrator. This expert reviewed the literature and made the final decision on inclusion. The study selection process was conducted and reported in accordance with the PRISMA 2020 statement. A PRISMA 2020 flow diagram was prepared to present record identification, duplicate removal, title and abstract screening, full-text eligibility assessment, reasons for exclusion at each stage, and the final number of included systematic reviews and meta-analyses. Upon completion of the screening process, standardized data extraction forms were used to collect the following information: basic literature details (authors, publication year), study characteristics (sample size, study design type), interventions and control measures, quality assessment tools employed, outcome measures, and the authors' primary conclusions. This establishes a reliable data foundation for subsequent analysis.

### Extraction of repetition rate

One common limitation in SRs/MAs is the potential for overlapping inclusion of data from the same primary study, which can compromise the validity of the evidence synthesis. To address this, an overlap matrix was constructed between the included SRs/MAs and their primary studies. The degree of overlap was then quantitatively assessed using the corrected covered area (CCA), calculated as: CCA = (n – r) / (rc – r) (15). In this formula, n refers to the total number of primary study inclusions across all SRs/MAs (counting duplicates), *r* is the number of unique primary studies after deduplication, and c represents the number of SRs/MAs included in the present re-evaluation. Based on established methodological guidance, the degree of overlap is classified using the following CCA thresholds: ≤ 5% corresponds to minimal overlap, 5%−10% to moderate overlap, 10%−15% to substantial overlap, and >15% to extensive overlap.

In handling the overlapping of primary studies, the 21 systematic reviews/meta-analyses included in this umbrella review exhibited a certain degree of overlap in original studies, with a calculated corrected covered area (CCA) of 10.5%, indicating a high level of overlap. Given that the nature of an umbrella review involves re-evaluating existing systematic review evidence, completely eliminating overlap in primary studies presents inherent methodological challenges. To minimize the potential impact of overlap on the pooled conclusions, the following principles were adhered to during quantitative synthesis: for each outcome measure, the most recently published systematic review with the highest methodological quality or the broadest coverage was prioritized as the primary data source, while other overlapping systematic reviews were used for cross-validation of effect size consistency. Additionally, sensitivity analyses were conducted to test the robustness of the pooled results and to assess the potential influence of overlap on the conclusions.

### Quality assessment

#### Risk of bias assessment

This study employed the ROBIS tool to assess the risk of bias in the included SRs/MAs (16). The assessment was conducted in three stages. (1) Relevance assessment to determine the alignment of the SRs/MAs with the current study; (2) Risk of process bias assessment, examining potential biases in the processes of literature search, screening, data extraction, and synthesis; (3) Overall risk judgment. Each stage includes structured questions, and evaluators select “Yes”, “Probably Yes”, “No”, “Probably No” or “No Information” based on the literature information. Finally, the risk of bias for each SRs/MAs is classified as low risk, high risk, or uncertain based on the pattern of responses.

#### Methodological quality assessment

This study employed the AMSTAR-2 tool to assess the methodological quality of the included SRs/MAs (17, 18). This tool comprises 16 items, of which 7 are key items (items 2, 4, 7, 9, 11, 13, and 15). During the evaluation process, two researchers independently assessed each item based on the content of the literature, with evaluation options including “met”, “partially met” and “not met”. Finally, based on the deficiencies in key and non-key items, the overall methodological quality was rated as “high”, “moderate”, “low” or “critically low”. Through this structured grading evaluation mechanism, AMSTAR-2 can more accurately elucidate the methodological reliability and limitations of the studies, thereby providing a foundation for subsequent evidence synthesis.

#### Reporting quality assessment

This study utilized the PRISMA 2020 guidelines to assess the reporting quality of the included SRs/MAs (19, 20). The guidelines comprise 27 items and 42 sub-items across seven sections. During the evaluation, the reporting completeness of each item was scored based on the literature: fully reported (*Y*) = 1 point, partially reported (PY) = 0.5 point, and not reported (*N*) = 0 point, with a total possible score of 42. Reporting quality was categorized into three tiers based on the total score: 33–42 points (≥80%) indicated high quality, corresponding to “relatively complete reporting”; 25–32 points (60%−80%) indicated moderate quality, corresponding to “reporting with certain deficiencies”; and scores below 25 points (< 60%) indicated low quality, corresponding to “substantially incomplete reporting”. This assessment can be used to systematically evaluate the completeness and transparency of research reports.

#### Evidence quality assessment

This study employed the GRADE system to assess the quality of evidence for the included SRs/MAs (21). This hierarchical approach employs a multidimensional analysis of study limitations, inconsistency, indirectness, imprecision, and publication bias, alongside a systematic assessment of effect sizes and other key factors. The evidence is then categorized into one of four quality levels—high, moderate, low, or very low—providing a clear, structured reference for subsequent evidence synthesis.

### Quantitative analysis

For quantitative synthesis, dichotomous outcomes were expressed as odds ratios (ORs), and continuous outcomes were expressed as standardized mean differences (SMDs). All pooled effect estimates were reported with 95% confidence intervals (CIs) and *P*-values. Heterogeneity was assessed using the *I*^2^ statistic and the corresponding *P*-value. A fixed-effects model was used when *I*^2^ ≤ 50% and *P* > 0.10, whereas a random-effects model was used when *I*^2^ > 50% or *P* ≤ 0.10. When substantial heterogeneity was present, sensitivity analyses and, where data permitted, exploratory subgroup or narrative analyses were used to explore potential sources.

### Qualitative analysis

For outcome measures included in the systematic reviews and meta-analyses that were unsuitable for quantitative synthesis, this study employed a qualitative synthesis approach. Using thematic analysis, key themes were extracted from the relevant literature. The core findings of the evidence were systematically organized, consensus conclusions and contradictory results were identified, and future research directions were proposed based on the limitations of existing studies, thereby providing qualitative evidence for reference in related fields.

## Results

### Results of literature screening

**Figure 1:**
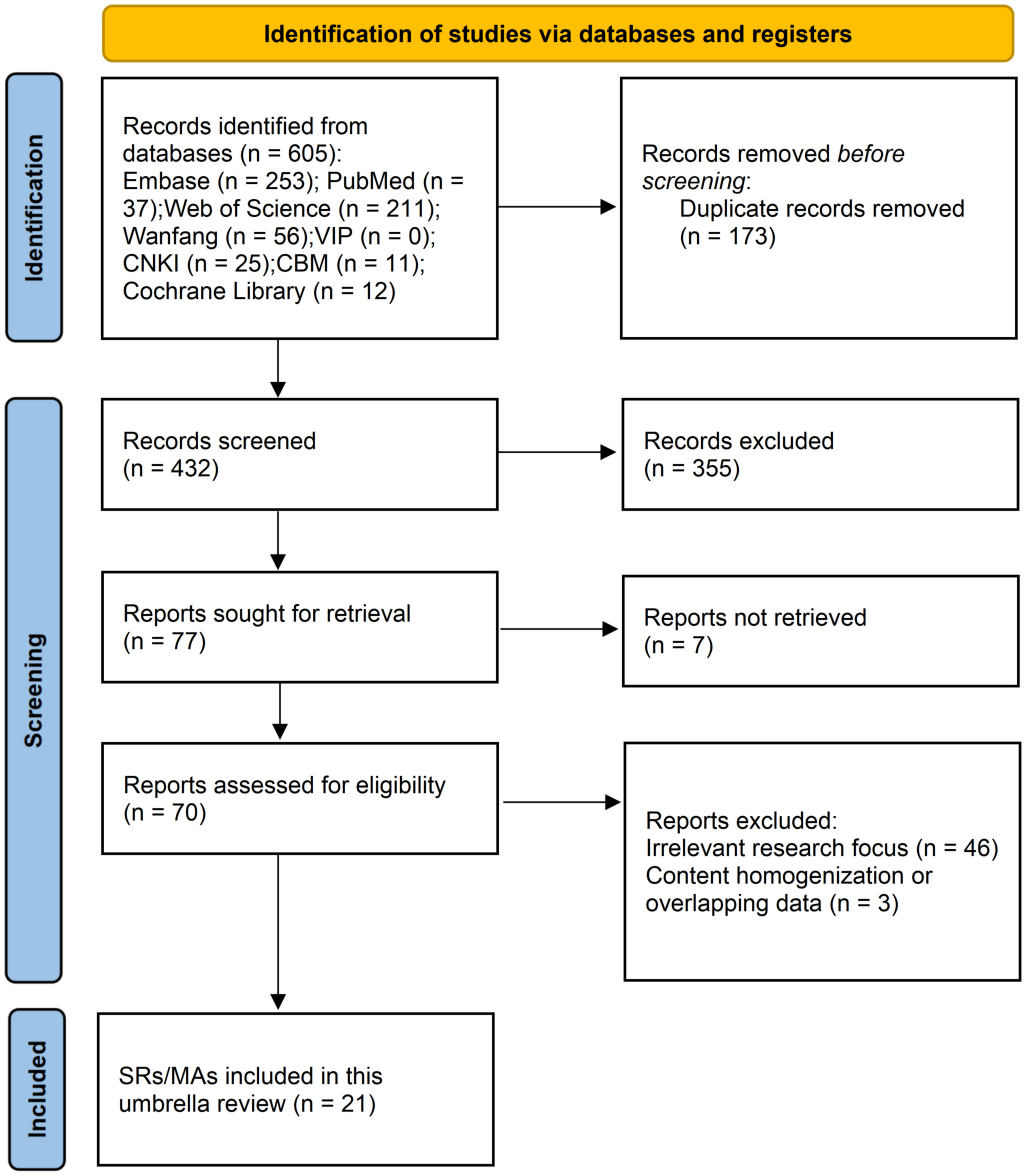
PRISMA 2020 flow diagram of the study selection process.

A total of 605 records were identified from eight databases. After removing 173 duplicate records, 432 records were screened by title and abstract. Of these, 355 records were excluded because they did not meet the predefined eligibility criteria: asthma was not the primary disease or treatment target (*n* = 148), vitamin D was not the eligible intervention or the control intervention was inappropriate (*n* = 99), the publication type was not an eligible systematic review/meta-analysis, such as non-systematic reviews, comments, or editorials (*n* = 67), the article was a primary clinical trial report (*n* = 27), an animal study (*n* = 13), or an *in vitro* study (*n* = 1). Full-text retrieval was sought for the remaining 77 reports, of which 7 could not be retrieved. Therefore, 70 full-text reports were assessed for eligibility. After full-text assessment, 49 reports were excluded, including 46 reports with an irrelevant research focus and 3 reports with content homogenization or overlapping data. Ultimately, 21 systematic reviews and meta-analyses (22–42) met the eligibility criteria and were included in the final analysis. The complete study selection process, including record identification, duplicate removal, title and abstract screening, full-text assessment, reasons for exclusion, and final inclusion, is presented in the PRISMA 2020 flow diagram in Figure 1.

### Basic characteristics of the included literature

This study included a total of 21 articles (15 in English, 6 in Chinese), comprising 3 dissertations and 18 journal papers published between 2015 and 2025. All eligible studies compared vitamin D levels between the treatment group and the control group. Regarding outcome measures, 17 studies assessed lung function-related indicators (22, 23, 25, 28–38, 40–42). 17 studies evaluated asthma exacerbations (23–25, 27–38, 40, 41). 13 studies reported vitamin D levels (serum 25(OH)D) (22, 23, 25, 26, 28, 30–34, 36, 38, 40). 9 studies evaluated Asthma Control Test (ACT) scores (23, 26, 31–33, 35–38). Adverse event rates were monitored in 9 studies (23, 27, 28, 31, 32, 34, 37, 38, 41). Fractional exhaled nitric oxide (FeNO) levels were measured in 8 studies (23, 31, 33–36, 38, 39). 4 studies evaluated inflammatory and immune biomarkers (31, 36, 37, 39), three assessed immune function indicators (37, 39, 42), 2 reported overall response rates (26, 42), 2 evaluated emergency department visits (33, 34), and 2 examined steroid use (33, 36). Other parameters included the annual frequency of asthma exacerbations (26), the number of children achieving well-controlled asthma (34), the proportion of participants who withdrew from the trial (37), the number of school days missed (36), and the provocative concentration of a substance required to induce a 20% fall in FEV1 (PC20-FEV1) (36). For the quality assessment of the literature, 19 studies (22–25, 27–35, 37–39, 41, 42) employed the Cochrane Risk of Bias assessment tool. Two studies (26, 33) utilized the Jadad scale. One study (40) applied the Joanna Briggs Institute (JBI) risk of bias assessment tool. One study (31) adopted the GRADE system. One study (36) conducted a risk of bias assessment but did not specify the tool used. The basic characteristics of the included studies are presented in Table 1.

**Table 1:** Characteristics of the included literature.

References	Number of literatures/ sample size	Intervention measures		Bias risk measurement tool	Endpoint measure	The main conclusions of the author
		Treatment group	Control group			
Fares et al. (22)	4/149	VD+ standard asthma therapy	Placebo or no VD + standard asthma therapy	Cochrane	①②	Current evidence regarding vitamin D supplementation in children with asthma remains inconclusive, with available studies being of very low to low quality. neither benefit nor lack of effect has been clearly established. To better evaluate the efficacy and safety of vitamin D in this population, large-scale, rigorously designed and well-conducted randomized controlled trials are needed.
Luo et al. (23)	12/271	VD+ standard asthma therapy	Placebo or no VD + standard asthma therapy	Cochrane	①②③④⑤⑥	While vitamin D supplementation safely elevates serum 25-hydroxyvitamin D levels, it does not reduce asthma exacerbations or fractional exhaled nitric oxide (FeNO), nor does it improve lung function or asthma symptoms when added to standard asthma controller therapy.
Pojsupap et al. (24)	5/625	VD+ standard asthma therapy	Placebo or no VD + standard asthma therapy	Cochrane	③	Evidence from this systematic review indicates that high-dose vitamin D may reduce asthma exacerbations. These findings warrant confirmation through larger, well-designed randomized controlled trials.
Riverin et al. (25)	8/573	VD+ standard asthma therapy	Placebo or no VD + standard asthma therapy	Cochrane	①②③	Randomized controlled trials offer limited, low-quality evidence that vitamin D supplementation may reduce asthma exacerbations. For other asthma-related outcomes in children, however, current evidence remains scarce or inconclusive. We suggest that future trials prioritize patient-centered outcomes that are comparable across studies, using standardized definitions of asthma exacerbations.
Jing et al. (26)	9/922	VD+ standard asthma therapy	Standard asthma therapy	Jadad	②⑤⑦⑧	Vitamin D combination therapy demonstrates satisfactory clinical efficacy in the adjunctive treatment of childhood asthma; however, given the limited number of existing clinical studies, future large-scale, multi-center, high-quality clinical trials are still required to provide more reliable and accurate evaluation results.
Jolliffe et al. (27)	Children: 5/297 adults: 2/658	VD + standard asthma therapy	Placebo + standard asthma therapy	Cochrane	③⑥	Vitamin D supplementation safely lowers the overall frequency of asthma exacerbations. However, definitive evidence indicating differential effects across patient subgroups was not established. Given the low cost of this intervention and the substantial economic burden posed by asthma exacerbations, vitamin D supplementation presents a potentially cost-effective strategy to mitigate this significant cause of morbidity and mortality.
Hao (28)	12/1,295	VD + standard asthma therapy	Placebo + standard asthma therapy	Cochrane	①②③⑥	“Supplementation with vitamin D provides a certain degree of evidence-based medical evidence for its use as an adjunct to conventional asthma therapy; however, further large-scale, rigorously designed randomized controlled trials are warranted to validate the clinical efficacy and safety of vitamin D supplements in patients with asthma.”
Tian et al. (29)	21/2,287	VD + standard asthma therapy	Placebo + standard asthma therapy	Cochrane	①③	Current low-quality evidence does not substantiate a beneficial effect of vitamin D supplementation in children with asthma. To better understand the relationship between vitamin D and pediatric asthma, large-scale, well-designed randomized controlled trials with standardized outcome measures and thorough safety assessments are warranted.
Hao (30)	9/702	VD + standard asthma therapy	Placebo + standard asthma therapy	Cochrane	①②③	Vitamin D supplementation can reduce the number of asthmatic attacks, but it only has limited effects on serum 25(OH)D, pulmonary function and asthma-symptom score. The existence of many potential confounding factors necessitates large-scale, long-term, well-designed RCTs in the future in order to understand the curative effect of vitamin D for pediatric asthma.
Wang et al. (31)	14/1,421	VD + standard asthma therapy	Placebo + standard asthma therapy	Cochrane+ GRADE	①③④⑤ ⑥⑨	Vitamin D supplementation was associated with a reduction in the rate of asthma exacerbations, particularly among patients with vitamin D insufficiency. Furthermore, in individuals with both airflow limitation and vitamin D insufficiency, vitamin D supplementation demonstrated a beneficial effect on pulmonary function.
Chen et al. (32)	12/1,543	VD + standard asthma therapy	Placebo + standard asthma therapy	Cochrane	①②③⑤⑥	vitamin D supplementation can safely reduce the rate of asthma exacerbation in both children and adults with asthma treated with corticosteroids.
Hao et al. (33)	8/738	VD + standard asthma therapy	Placebo + standard asthma therapy	Jadad cochrane	①②③④⑤ ⑩⑪	Vitamin D supplementation significantly increased serum vitamin D levels in children with asthma; however, it did not lead to improved asthma control. There was also evidence suggesting a potential decline in lung function among supplemented patients. The incidence of adverse events was comparable between the vitamin D and placebo groups, indicating that vitamin D supplementation is generally considered safe.
Kumar et al. (34)	18/1,579	VD + standard asthma therapy	Placebo + standard asthma therapy	Cochrane	①②③④⑥⑩⑫	No protective effect was observed with adjuvant vitamin D supplementation in preventing moderate to severe asthma exacerbations requiring rescue systemic corticosteroids in children.
Liu et al. (35)	10/1,349	VD + standard asthma therapy	Placebo or no VD + standard asthma therapy	Cochrane	①③④⑤	Vitamin D supplementation can reduce asthma exacerbations, especially in children, and within 6 months of follow up time. In addition, Vitamin D has a positive effect on improving FEV1 of patients whose FEV1 baseline value is less than 70%, but more RCTs are still needed to support this conclusion.
Nitzan et al. (36)	6/1,438	VD + standard asthma therapy	Placebo + standard asthma therapy	N/A	①②③④⑤⑨ ⑪⑮⑯	We conclude that currently available articles provide us with a relatively weak level of evidence. They do not allow us to confirm the beneficial effects of vitamin D supplementation in children with asthma that were suggested from retrospective and cross-sectional studies.
Williamson et al. (37)	20/2,225	VD + standard asthma therapy	Placebo + standard asthma therapy	Cochrane	①③⑤⑥ ⑨⑬⑭	Contrary to the conclusions of our earlier Cochrane Review on this subject, the current updated analysis finds no evidence that supplementation with vitamin D or its hydroxylated metabolites reduces the risk of asthma exacerbations or improves asthma control.
Sun (38)	21/2,287	VD + standard asthma therapy	Placebo + standard asthma therapy	Cochrane	①②③④ ⑤⑥	Supplementation with vitamin D and its analogs improves vitamin D deficiency while improving not only asthma control and reducing the risk of acute exacerbations in asthma patients, but also improving FEV1% in adult asthma patients. As a low-cost, low-risk adjunctive therapy, vitamin D supplementation offers a potential therapeutic option for disease control in bronchial asthma.
El Abd et al. (39)	13/1,459	VD + standard asthma therapy	Placebo + standard asthma therapy	Cochrane	④⑨⑬	Although vitamin D supplementation demonstrates no significant effect on key type 2 inflammatory biomarkers (such as serum IgE, blood and sputum eosinophils, and fractional exhaled nitric oxide) in asthma patients, it appears to elevate levels of the anti-inflammatory biomarker IL-10, suggesting a potential anti-inflammatory role in asthma.
Fedora et al. (40)	10/1,243	VD + standard asthma therapy	Placebo + standard asthma therapy or standard asthma therapy	JBI	①②③	Vitamin D supplementation reduces the rate of asthma exacerbations and improves FEV1 in children, with the magnitude of effect varying by dosage and treatment duration.
Niu et al. (41)	12/1,295	VD + standard asthma therapy	Placebo + standard asthma therapy	Cochrane	①②③⑥	Vitamin D supplementation plays an important role in asthma management by significantly reducing exacerbations, including those requiring systemic corticosteroids or emergency hospital visits.
Yang and Zhang (42)	12/1,380	VD + FP	FP	Cochrane	①⑦⑬	The combination of vitamin D and fluticasone propionate demonstrates superior efficacy in the treatment of childhood asthma, yielding improvements in both lung function and immune function, without increasing the incidence of adverse effects.

^①^Pulmonary function-related indicators; ^②^Vitamin D levels (serum 25(OH)D); ^③^Asthma exacerbation; ^④^FeNO; ^⑤^ACT; ^⑥^Adverse events; ^⑦^Total efficiency rate; ^⑧^Frequency of asthma attacks within 1 year; ^⑨^Inflammation and immune biomarkers; ^⑩^Emergency department visits; ^⑪^Use of glucocorticoids; ^⑫^Number of children with well-controlled asthma; ^⑬^Immune function index; ^⑭^Proportion of participants withdrawing from trial; ^⑮^School days missed; ^⑯^PC20-FEV1.

### Duplication rate of the original literature

This study ultimately included 21 SRs/MAs (22–42), which collectively encompass 220 primary studies from the original literature. After deduplication, a total of 71 independent primary studies were included. The calculated corrected covered area (CCA) value was (220–71) / (71 × 21–71) ≈ 0.1050, corresponding to a high degree of overlap. This result indicates a certain level of duplicate inclusion in the primary studies covered by the SRs/MAs included in this research. This finding suggests that some primary studies were repeatedly included across multiple reviews, which may have increased the apparent weight of repeated evidence in the overall interpretation. Therefore, overlapping SRs/MAs were not regarded as fully independent evidence sources when interpreting the findings. Instead, they were mainly used to assess the consistency of effect direction, heterogeneity, and certainty of evidence across reviews.

### Results of the risk of bias assessment

All included studies (22–42) were rated as “passed” in the first phase (relevance assessment) of the ROBIS tool. In the second phase, for Domain 1 (study eligibility criteria), the risk of bias was low. For Domain 2 (identification and selection of studies), 16 studies (22, 24, 25, 27–29, 31–39, 41) were assessed as low risk of bias, 4 studies (23, 30, 40, 42) as “unclear risk” and 1 study (26) as “high risk”, For Domain 3 (data collection and study appraisal), 18 studies (22–25, 27–35, 37–41) were assessed as low risk of bias, 2 studies (36, 42) as “unclear risk” and 1 study (26) as “high risk”, For Domain 4 (synthesis and findings), 20 studies (22–29, 31–42) were assessed as low risk of bias, and 1 study (30) as “unclear risk”. In the third phase (overall risk of bias assessment), 18 studies (22–25, 27–35, 37–41) were rated as low risk of bias, 1 study (36) as “unclear risk” and 2 studies (26, 42) as “high risk”.

### Results of the methodological quality assessment

**Figure 2:**
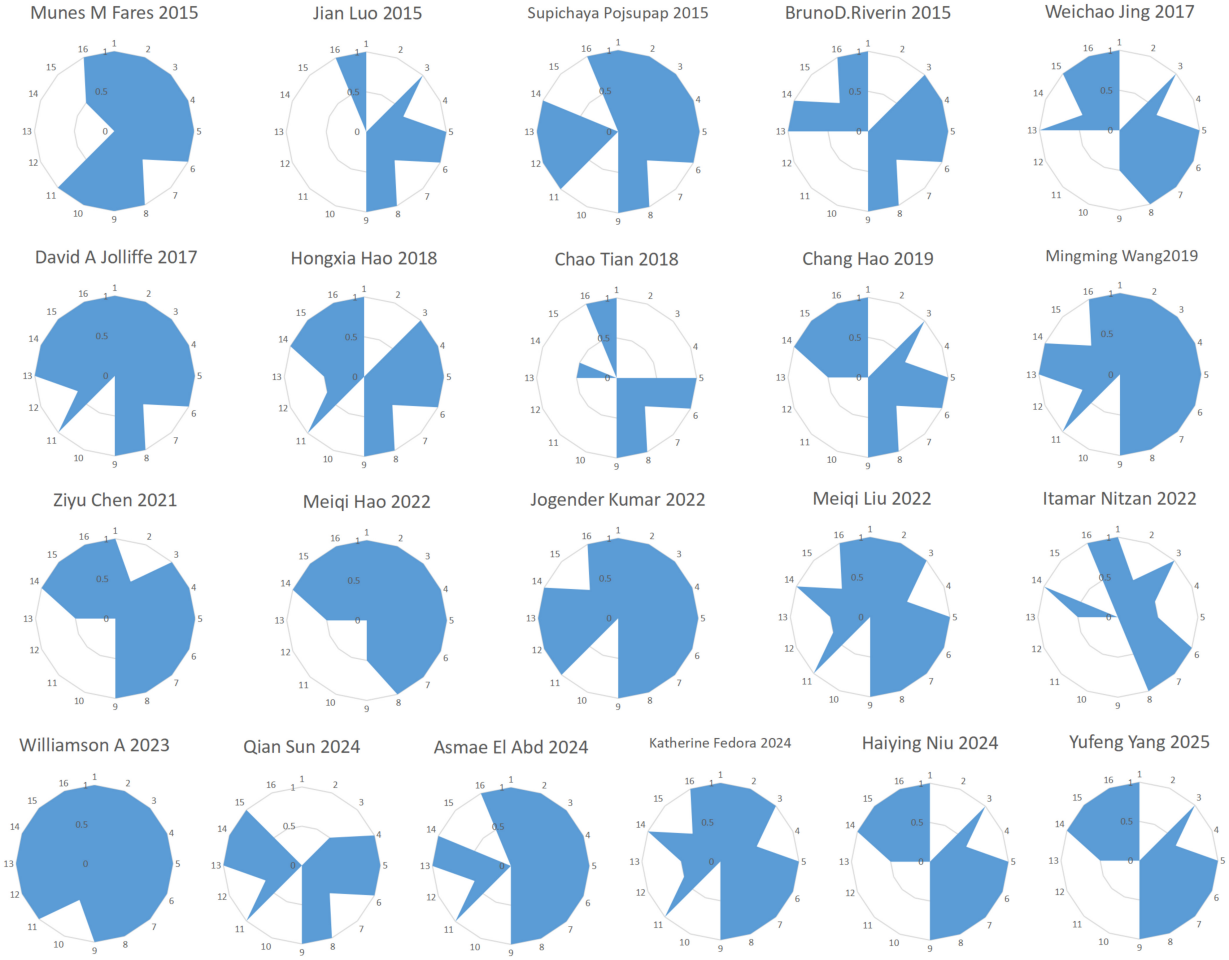
Radar chart of scores for each item of AMSTAR-2.

The methodological quality assessment of the included SRs/MAs showed that 9 studies were rated as high quality (22, 27, 31–35, 37, 40), while 12 studies were rated as low quality (23–26, 28–30, 36, 38, 39, 41, 42). For the key items, all 21 studies reported items 11 and 7 completely. Subsequent compliance rates were as follows: item 4 (20/21, 95.2%), item 9 (20/21, 95.2%), item 13 (20/21, 95.2%), item 15 (16/21, 76.2%), and item 2 (12/21, 57.1%). For non-critical items, complete reports were provided for items 1, 3, 5, 6, and 8 (21/21, 100%), while compliance varied for the remaining items: item 14 (20/21, 95.2%), item 16 (20/21, 95.2%), item 12 (10/21, 47.6%), and item 10 (2/21, 9.5%). A detailed methodological quality assessment of the included studies is presented in Figure 2.

### Results of the reporting quality assessment

**Figure 3:**
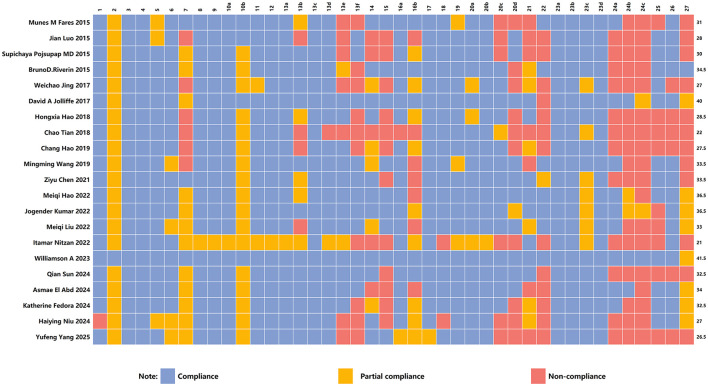
Cartesian heatmap of the scores of each item in PRISMA 2020.

The PRISMA 2020 checklist, with a maximum attainable score of 42 points, offers a structured framework for evaluating the completeness and transparency of systematic reviews and meta-analyses. Its criteria encompass all key sections of a manuscript, including the abstract, introduction, methods, results, and discussion. The included 21 studies scored between 21 and 41.5 (mean ≈ 31.26) on the PRISMA 2020 assessment. Among them, nine were classified as high-quality (25, 27, 31–35, 37, 39), 10 as moderate-quality (22–24, 26, 28, 30, 40–42), and the remaining two as low-quality in reporting (29, 36). Among the 42 checklist items, six items (15, 22, 24a, 24b, 24c, 27) exhibited particularly poor reporting compliance (completion rate ≤ 50% across all 21 studies). These deficient items primarily pertained to: determination/assessment, certainty of evidence assessment, registration and protocols, and availability of data, code, and other materials. Detailed reporting patterns are shown in Figure 3.

### Results of the evidence quality assessment

We evaluated the evidence quality of 108 composite outcome measures from the included studies using a grading approach. The assessment results showed that 10 outcomes (9.3%) were rated as high-quality evidence, 31 outcomes (28.7%) as moderate-quality evidence, 31 outcomes (28.7%) as low-quality evidence, and 36 outcomes (33.3%) as very low-quality evidence. The complete GRADE evidence profiles for all outcomes are presented in Table 2.

**Table 2:** Evidence quality assessment.

The included studies	Endpoint measure	Downgrading factor					Effect size	95% CI	I^2^/%	P	Evidence quality
		RB	IC	ID	IP	PB					
Fares et al. (22)	FEV1%	–1^①^	0	0	–1^④^	0	MD = 0.54	[-5.28, 4.19]	54	>0.05	Low
	Vitamin D levels	–1^①^	–^②^	0	–1^④^	0	MD = 6.56	[–0.64, 13.77]	97	0.07	Very low
Luo et al. (23)	Rate of asthma exacerbations	–1^①^	–^②^	0	–1^④^	0	RR = 0.66	[0.32, 1.37]	81	0.26	Very low
	FEV1%	–1^①^	–^②^	0	–1^④^	0	MD = –0.02	[–0.15, 0.11]	0	0.77	Very low
	FeNO	–^②^	0	0	–1^④^	0	MD = –0.02	[–0.16, 0.12]	0	0.78	Low
	ACT score	–^②^	0	0	–1^④^	0	MD = –0.05	[–0.17, 0.06]	0	0.36	Low
	Serum 25–hydroxyvitamin D levels	–1^①^	0	0	0	0	MD = 0.51	[0.35, 0.68]	0	<0.001	Moderate
	Rate of adverse events	–1^①^	0	0	–1^④^	0	MD = 1.16	[0.74, 1.81]	0	0.52	Low
Pojsupap et al. (24)	Asthma exacerbations	–1^①^	0	0	0	0	RR = 0.41	[0.27, 0.63]	0	<0.0001	Moderate
Pojsupap et al. (25)	Asthma exacerbations	–1^①^	0	0	–1^④^	0	RR = 0.41	[0.27, 0.63]	0	<0.0001	Low
	Serum 25-hydroxyvitamin D concentration	–1^①^	–^②^	0	0	0	MD = 19.66	[5.96, 33.37]	94	0.005	Low
	FEV1%	–1^①^	0	0	–1^④^	0	WMD = 0.00	[–3.17, 3.18]	0	0.99	Low
Pojsupap et al. (26)	Total effective rate	–1^①^	0	0	–1^④^	–1^⑤^	OR = 3.37	[1.73, 6.58]	0	0.0004	Very low
	ACT score	–1^①^	–^②^	0	–1^④^	0	OR = 1.18	[0.07, 10.05]	93	0.91	Very low
	Frequency of asthma attacks within one year	–1^①^	–^②^	0	–1^④^	0	WMD = –4.26	[–9.52, 0.99]	99	0.11	Very low
	Serum 25-hydroxyvitamin D3 (25-(OH)D3) level	–1^①^	0	0	0	0	WMD = 9.90	[5.60, 14.20]	0	<0.00001	Moderate
Jolliffe et al. (27)	Incidence of asthma exacerbation requiring treatment with systemic corticosteroid	0	0	0	0	0	RR = 0.74	[0.56, 0.97]	0	0.03	High
	Incidence of asthma exacerbations resulting in emergency department attendance or hospital admission, or both	0	0	0	–1^④^	0	OR = 0.46	[0.24, 0.91]	N/A	0.03	Moderate
	Proportion of participants with at least one asthma exacerbation requiring treatment with systemic corticosteroids	0	0	0	–1^④^	0	OR = 0.75	[0.51, 1.09]	N/N	0.13	Moderate
	Time to first asthma exacerbation requiring treatment with systemic corticosteroids	0	0	0	–1^④^	0	HR = 0.78	[0.55, 1.10]	N/A	0.16	Moderate
	Incidence of serious adverse events of any cause	0	0	0	–1^④^	0	OR = 0.87	[0.46, 1.63]	N/N	0.66	Moderate
Hao (28)	Number of acute exacerbations of asthma	–1^①^	–^②^	0	–1^④^	0	OR = 0.53	[0.28, 0.99]	65	0.05	Very low
	Acute exacerbation of asthma requiring systemic corticosteroids therapy	–1^①^	0	0	0	0	RR = 0.64	[0.46, 0.90]	0	0.01	Moderate
	Asthma exacerbations requiring ED visit or hospitalization or both	–1^①^	0	0	0	0	OR = 0.39	[0.19, 0.78]	0	0.008	Moderate
	FEV1%	–1^①^	0	0	0	0	SMD = 0.24	[0.06, 0.42]	12	0.01	Moderate
	Serum 25-hydroxyvitamin D levels	–1^①^	–^②^	0	0	0	SMD = 0.75	[0.46, 1.05]	73	<0.00001	Low
	Adverse events	–1^①^	0	0	–1^④^	0	RR = 1.02	[0.69, 1.52]	0	0.91	Low
	Fatal asthma exacerbation	–1^①^	0	0	–1^④^	0	RD = 0.00	[–0.01, 0.01]	0	1.00	Low
Tian et al. (29)	FEV1%	–1^①^	–^②^	0	–1^④^	0	MD = 0.54	[–5.28, 4.19]	54	0.83	Very low
	Number of asthma acute exacerbations	–1^①^	–^②^	0	–1^④^	0	RR = 0.63	[0.24, 1.70]	59	0.36	Very low
Hao (30)	Asthmatic attacks	–1^①^	0	0	0	0	OR = 0.27	[0.17, 0.45]	0	<0.00001	Moderate
	Serum 25-hydroxyvitamin D level	–1^①^	–^②^	0	–1^④^	0	MD = 11.92	[–3.50, 27.33]	99	0.13	Very low
	FEV1%	–1^①^	–^②^	0	–1^④^	0	MD = 16.48	[–5.56, 38.53]	91	0.14	Very low
	FEV1	–1^①^	–^②^	0	–1^④^	0	MD = –0.14	[–0.38, 0.11]	87	0.28	Very low
Wang et al. (31)	Rate of asthma exacerbation	–1^①^	0	0	–1^④^	–1^⑤^	RR = 0.73	[0.58, 0.92]	5	0.007	Very low
	FEV1%	–1^①^	–^②^	0	–1^④^	0	MD = 0.67	[–3.83, 5.16]	86	0.77	Very low
	ACT scores	–1^①^	–^②^	0	–1^④^	0	MD = 0.67	[–2.61, 4.22]	86	0.64	Very low
	FeNO	–1^①^	0	0	–1^④^	0	MD = 1.86	[–4.59, 8.32]	0	0.57	Low
	IL-10	–1^①^	–^②^	0	–1^④^	0	MD = 0.46	[–0.44, 1.36]	86	0.32	Very low
	Adverse events	–1^①^	0	0	–1^④^	0	RR = 0.87	[0.41, 1.81]	0	0.71	Low
Chen et al. (32)	Rate of asthma exacerbation	0	0	0	0	0	RR = 0.70	[0.59, 0.83]	0	<0.001	High
	Serum 25(OH)D levels	0	–^②^	0	0	0	SMD = 1.07	[0.77, 1.38]	85.4	<0.001	Moderate
	FEV1%	0	0	0	–1^④^	0	SMD = –0.02	[–0.13, 0.09]	21.4	0.687	Moderate
	ACT scores	–1^①^	–^②^	0	–1^④^	0	SMD = 0.04	[–0.19, 0.27]	68.7	0.744	Very low
	Adverse events	0	0	0	–1^④^	0	RR = 1.06	[0.89, 1.25]	0	0.522	Moderate
Hao et al. (33)	Serum Vitamin D Levels	–1^①^	0	0	–1^④^	0	MD = 13.51	[4.24, 22.79]	98	0.004	Low
	CACT Scores	0	0	0	–1^④^	0	MD = 0.15	[–0.43, 0.74]	0	0.61	Moderate
	Asthma exacerbation	–1^①^	0	–1^③^	–1^④^	0	RR = 0.92	[0.68, 1.25]	1	0.60	Very low
	Hospitalizations for asthma exacerbation	–1^①^	0	0	–1^④^	0	RR = 1.20	[0.48, 2.96]	0	0.70	Low
	Acute care visits	–1^①^	0	0	–1^④^	0	RR = 1.13	[0.77, 1.65]	7	0.53	Low
	Steroid use	–1^①^	–^②^	0	–1^④^	0	RR = 1.03	[0.41, 2.57]	56	0.95	Very low
	FeNO	–1^①^	–^②^	0	–1^④^	0	MD = –3.95	[–22.87, 14.97]	68	0.68	Very low
	FEV1%	–1^①^	0	0	–1^④^	0	MD = –4.77	[–9.35, –0.19]	0	0.04	Low
	Percentage of predicted forced vital capacity, FVC%	–1^①^	0	0	–1^④^	0	MD = –5.01	[–9.99, –0.02]	0	0.05	Low
Kumar et al. (34)	Asthma exacerbations requiring rescue systemic corticosteroids	0	0	0	–1^④^	0	RR = 1.13	[0.86, 1.48]	0	0.38	Moderate
	Asthma exacerbations of any severity	–1^①^	–^②^	–1^③^	–1^④^	0	RR = 0.84	[0.65, 1.09]	58	0.007	Very low
	Number of children requiring emergency/unscheduled visits	–1^①^	0	0	–1^④^	0	RR = 0.97	[0.89, 1.07]	0	0.4	Low
	Number of children requiring hospitalizations for asthma exacerbation	0	0	0	–1^④^	0	RR = 1.38	[0.52, 3.66]	0	0.8	Moderate
	Number of children with well-controlled asthma	–1^①^	0	0	–1^④^	0	RR = 1.00	[0.97, 1.04]	0	0.9	Low
	FEV1%	0	–^②^	0	–1^④^	0	MD = –2.64	[–7.04, 1.77]	62	0.05	Low
	FeNO	–1^①^	–^②^	0	–1^④^	0	MD = –2.87	[–24.66, 18.91]	>75	N/A	Very low
	Vitamin D levels post-intervention	0	–^②^	0	0	0	MD = 10.86	[6.3, 15.05]	>50	N/A	Moderate
	Number of children with serious adverse events	–1^①^	0	0	–1^④^	0	RR = 1.30	[0.55, 3.07]	0	0.9	Low
Liu et al. (35)	Rate of asthma exacerbations	–1^①^	–^②^	–1^③^	0	0	RR = 0.60	[0.41, 0.88]	64	<0.01	Very low
	FEV1%	–1^①^	–^②^	0	–1^④^	0	SMD = 0.04	[–0.35, 0.43]	78	<0.01	Very low
	ACT score	–1^①^	0	0	0	0	SMD = 0.04	[–0.13, 0.21]	0	0.87	Moderate
	FeNO	–1^①^	0	0	–1^④^	0	SMD = –0.01	[–0.22, 0.20]	23	0.27	Low
Williamson et al. (37)	Proportion of participants with one or more exacerbations treated with systemic corticosteroids	0	0	0	0	0	OR = 1.04	[0.81, 1.34]	0	0.75	High
	Rate of exacerbations treated with systemic corticosteroids	0	0	0	0	0	RR = 0.86	[0.62, 1.19]	60	0.36	High
	Time to first exacerbation treated with systemic corticosteroids	0	0	0	0	0	HR = 0.82	[0.59, 1.15]	22	0.26	High
	Proportion of participants with one or more exacerbations requiring emergency department visit or hospitalisation, or both	0	0	0	–1^④^	0	OR= 0.56	[0.26, 1.21]	33	0.14	Moderate
	End-study (cACT) or (ACT) score	0	–^②^	0	0	0	MD = 0.23	[–0.26, 0.73]	29	0.36	Moderate
	End-study % (FEV1)	0	0	0	0	0	MD = 0.20	[–1.24, 1.63]	25	0.79	High
	Proportion of participants with one or more serious adverse events due to any cause	0	0	0	0	0	OR = 0.89	[0.56, 1.41]	0	0.62	High
	Proportion of participants with fatal asthma exacerbation	0	0	0	–1^④^	0	RD = 0.00	[–0.01, 0.01]	0	1.00	Moderate
	Proportion of participants with one or more exacerbation as defined in primary trials	0	0	0	0	0	OR = 0.77	[0.51, 1.17]	57	0.22	High
	End-study % eosinophils, lower airway	0	–^②^	0	0	0	MD = –0.38	[–1.92, 1.15]	43	0.62	Moderate
	End-study log10 total IgE, IU/ml	0	0	0	0	0	MD = 0.07	[–0.13, 0.26]	0	0.51	High
	End-study % (FVC)	0	–^②^	0	0	0	MD = 1.84	[–3.60, 7.29]	73	0.51	Moderate
	End-study peak expiratory flow rate (PEFR) (L/min)	0	–^②^	0	0	0	MD = 4.84	[–8.95, 18.62]	79	0.49	Moderate
	Proportion of participants withdrawing from trial	0	0	0	0	0	OR = 1.05	[0.77, 1.43]	0	0.76	High
Sun (38)	FEV1%	–1^①^	–^②^	0	–1^④^	0	MD=0.51	[–1.58, 2.61]	49	0.63	Very low
	Asthma Control Test/Children-Asthma Control Test	–1^①^	0	0	0	0	SMD=0.17	[0.03, 0.31]	6	0.02	Moderate
	Asthma Exacerbation	–1^①^	0	0	0	0	RR=0.81	[0.69, 0.96]	32	0.02	Moderate
	Change in serum 25-hydroxyvitamin D from baseline	–1^①^	–^②^	0	0	0	MD=10.29	[3.62, 16.95]	99	0.002	Low
	Fractional exhaled nitric oxide	–1^①^	–^②^	0	–1^④^	0	MD=-2.52	[–8.77, 3.74]	60	0.43	Very low
	Adverse events	–1^①^	0	0	–1^④^	0	RR=1.33	[0.65, 2.70]	0	0.43	Low
El Abd et al. (39)	IgE level	0	0	0	–1^④^	0	MD = 0.06	[–0.13, 0.26]	0	0.52	Moderate
	Blood eosinophil count	–1^①^	0	0	–1^④^	0	MD = –0.02	[–0.11, 0.07]	0	0.69	Low
	FeNO	0	0	0	–1^④^	0	MD = –4.10	[–10.95, 2.75]	16	0.24	Moderate
	IL-10 level	0	–^②^	0	–1^④^	0	MD = 18.85	[1.11, 36.59]	100	0.04	Low
Fedora et al. (40)	Incidence of asthma exacerbations	–1^①^	–^②^	0	–1^④^	0	RR = 0.62	[0.44, 0.87]	61	0.006	Very low
	Serum 25-hydroxyvitamin D (25(OH)D) level	0	–^②^	0	0	–1^⑤^	SMD = 1.95	[1.18, 2.72]	94	<0.00001	Low
	FEV1%	0	–^②^	0	–1^④^	–1^⑤^	SMD = –0.23	[–0.46, –0.01]	0	0.04	Very low
Niu et al. (41)	Incidence of asthma exacerbations	–1^①^	–^②^	0	–1^④^	0	OR = 0.54	[0.29, 0.98]	65	0.04	Very low
	Incidence of asthma exacerbations requiring systemic corticosteroids	0	0	0	0	–1^⑤^	RR = 0.65	[0.46, 0.90]	0	0.01	Moderate
	Incidence of asthma exacerbations requiring emergency department visit or hospitalization, or both	0	0	0	0	–1^⑤^	RR = 0.39	[0.19, 0.78]	0	0.008	Moderate
	FEV1%	–1^①^	–^②^	0	–1^④^	0	SMD = 0.24	[0.06, 0.42]	12	0.010	Very low
	Serum 25-hydroxyvitamin D (25(OH)D) level	–1^①^	–^②^	0	–1^④^	0	MD = 26.2	[21.4, 31.0]	73	<0.00001	Very low
	Incidence of adverse events	0	0	0	–1^④^	–1^⑤^	RR = 1.02	[0.69, 1.50]	0	0.92	Low
	Incidence of fatal asthma exacerbations	–1^①^	0	0	–1^④^	–1^⑤^	RD = 0.00	[–0.02, 0.02]	0	1.00	Very low
Yang and Zhang (42)	Total treatment effective rate	–1^①^	0	0	0	–1^⑤^	RR = 1.17	[1.11, 1.24]	0	<0.00001	Low
	FVC	–1^①^	–^②^	0	0	–1^⑤^	MD = 0.52	[0.32, 0.71]	88	<0.00001	Very low
	FEV1%	–1^①^	–^②^	0	0	–1^⑤^	MD = 0.50	[0.38, 0.61]	83	<0.00001	Very low
	Lung function index: peak expiratory flow	–1^①^	–^②^	0	–1^④^	–1^⑤^	MD = 0.79	[0.35, 1.22]	95	0.0004	Very low
	IgA level	–1^①^	–^②^	0	0	–1^⑤^	MD = 0.56	[0.41, 0.71]	93	<0.00001	Very low
	IgG level	–1^①^	0	0	0	–1^⑤^	MD = 4.33	[3.70, 4.96]	0	<0.00001	Low
	IgM level	–1^①^	0	0	0	–1^⑤^	MD = 0.67	[0.62, 0.72]	0	<0.00001	Low

^①^Methodological quality of included studies was low, with biases in randomization, allocation concealment, and blinding; ^②^The heterogeneity was large and low confidence interval overlap; ^③^The population was not broadly representative; ^④^Small sample size, 95% confidence intervals include null values; ^⑤^Few studies were included, the funnel plot was not symmetrical, Egger's test found that publication bias or results were positive and there was no publication bias evaluation.

### Quantitative analysis

To improve the interpretability of the findings, quantitative results were further summarized using a clinically oriented narrative synthesis. Outcomes were grouped into five domains: symptom control, asthma exacerbation, lung function, airway inflammation, and safety. Potential effect modifiers, including age, baseline vitamin D status, dosing strategy, treatment duration, and asthma severity, were considered where such information was available from the included SRs/MAs. Because individual participant data were unavailable for most reviews, these subgroup patterns were interpreted narratively rather than through formal individual-level subgroup meta-analysis.

### Asthma control test (ACT)

**Figure 4:**
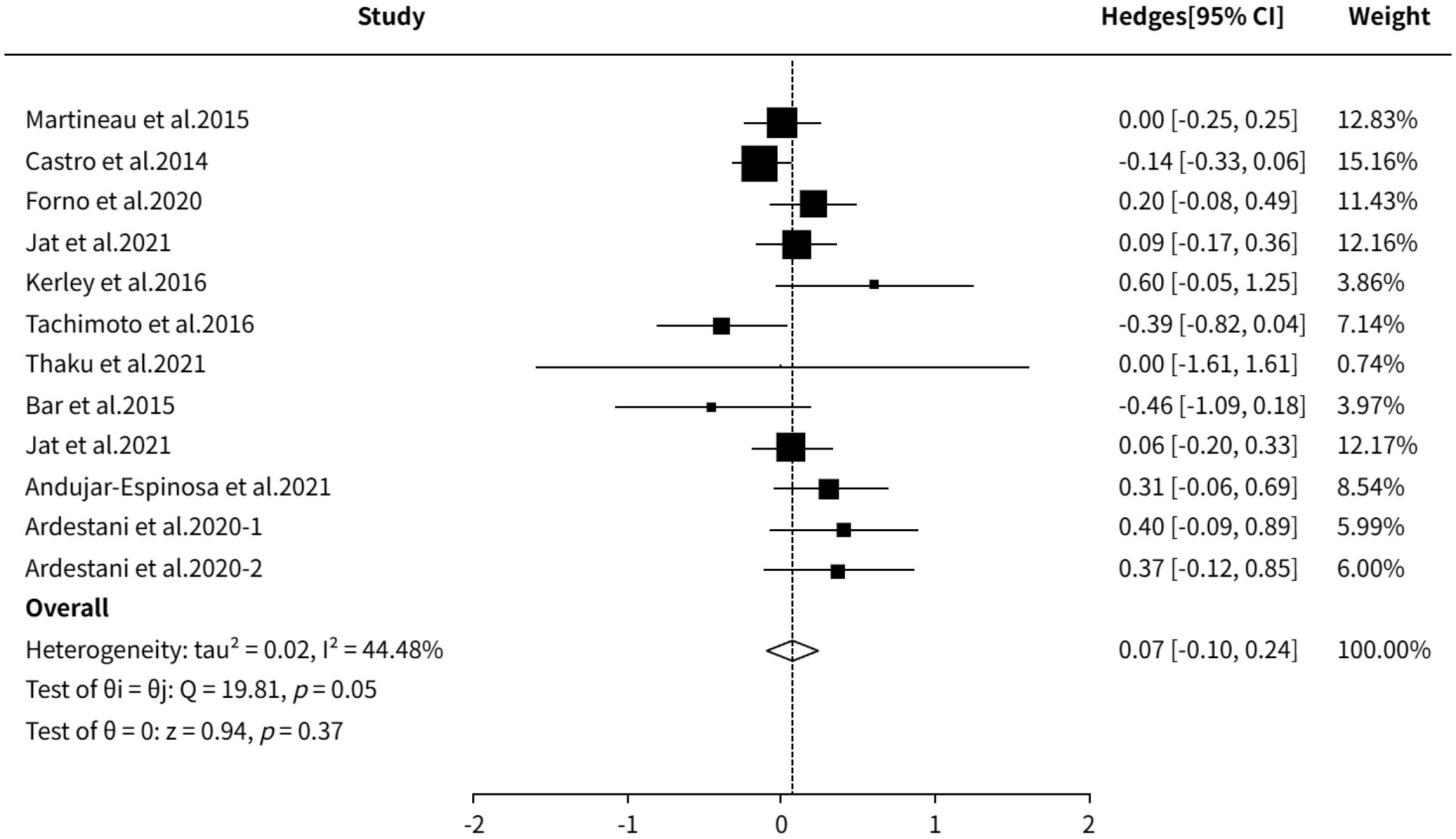
Meta-analysis of ACT scores.

9 SRs/MAs (23, 26, 31–33, 35–38) included in the analysis reported quantitative data on changes in ACT scores. A random-effects meta-analysis indicated that vitamin D supplementation as an adjunct to conventional therapy showed no significant difference compared with the control group in improving ACT scores (SMD = 0.07, 95% CI [−0.10, 0.24], *P* = 0.37). Moderate heterogeneity was observed in the study (*I*^2^ = 44.48%). Sensitivity analysis (by sequentially excluding individual studies) and cumulative meta-analysis showed that the combined effect size was stable and robust, with no directional change in the results, indicating that the current conclusions were minimally influenced by individual studies. Visual inspection of the funnel plot did not reveal significant signs of publication bias. Overall, existing evidence suggests that the effect of vitamin D supplementation on symptom control (as measured by ACT scores) in asthma patients remains unclear, and future studies should further investigate the potential sources of heterogeneity. See Figure 4 for detailed results.

### Adverse events

**Figure 5:**
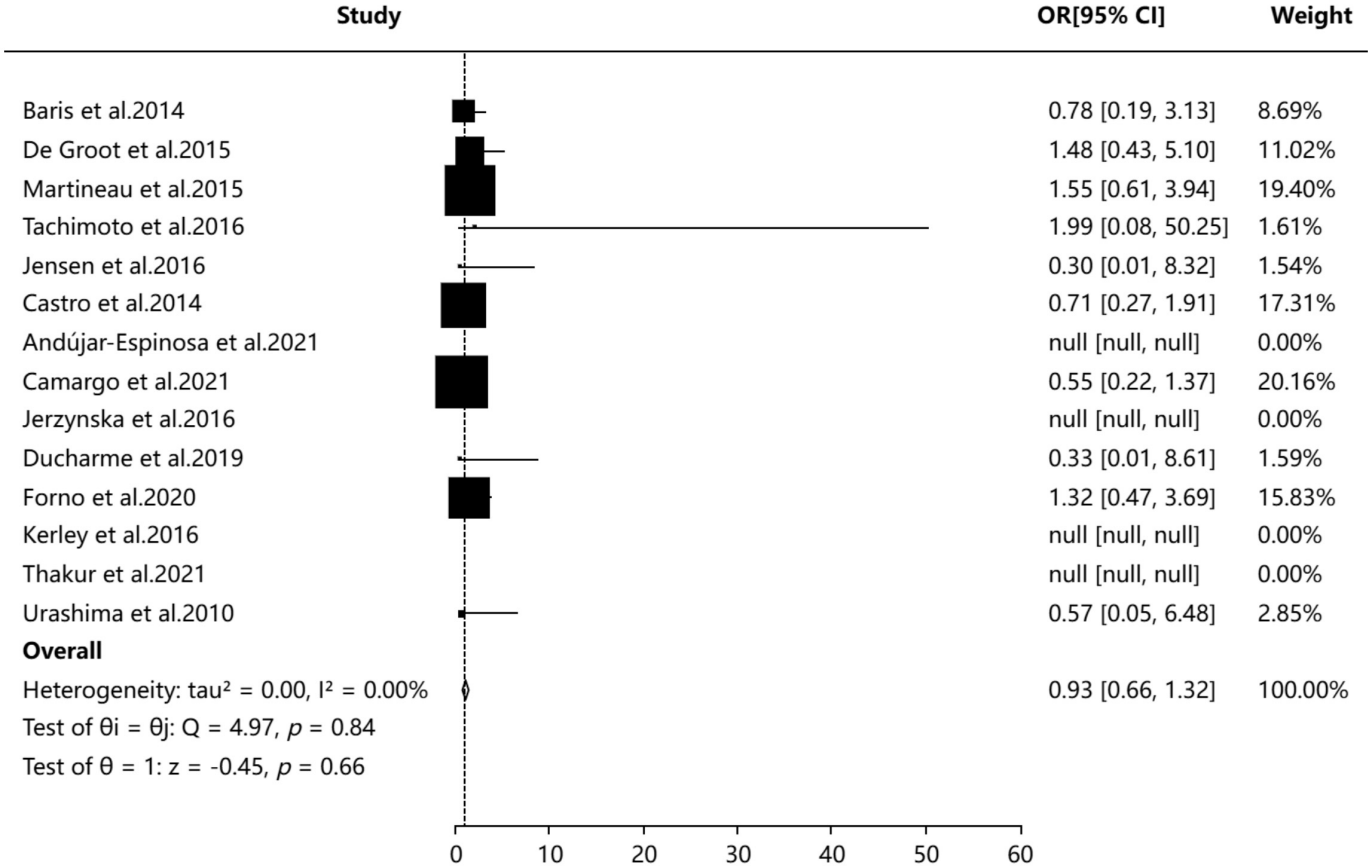
Meta-analysis of adverse event scores.

9 SRs/MAs (23, 27, 28, 31, 32, 34, 37, 38, 41) that reported quantitative data on adverse event scores were included in the analysis. A random-effects meta-analysis revealed that vitamin D supplementation as an add-on to conventional therapy showed no significant difference in the incidence of adverse events compared with the control group (OR = 0.93, 95% CI [0.66, 1.32], *P* = 0.66). No significant heterogeneity was observed among studies (*I*^2^ = 0.00%). Sensitivity analysis (sequential exclusion of individual studies) and cumulative meta-analysis demonstrated that the pooled effect size remained stable and consistent in direction, indicating good robustness of the current conclusion. Visual inspection of the funnel plot revealed no obvious signs of publication bias. Taken together, current evidence suggests that vitamin D supplementation on top of conventional asthma treatment does not significantly increase the risk of adverse events. See Figure 5 for detailed results.

### Asthma exacerbation

**Figure 6:**
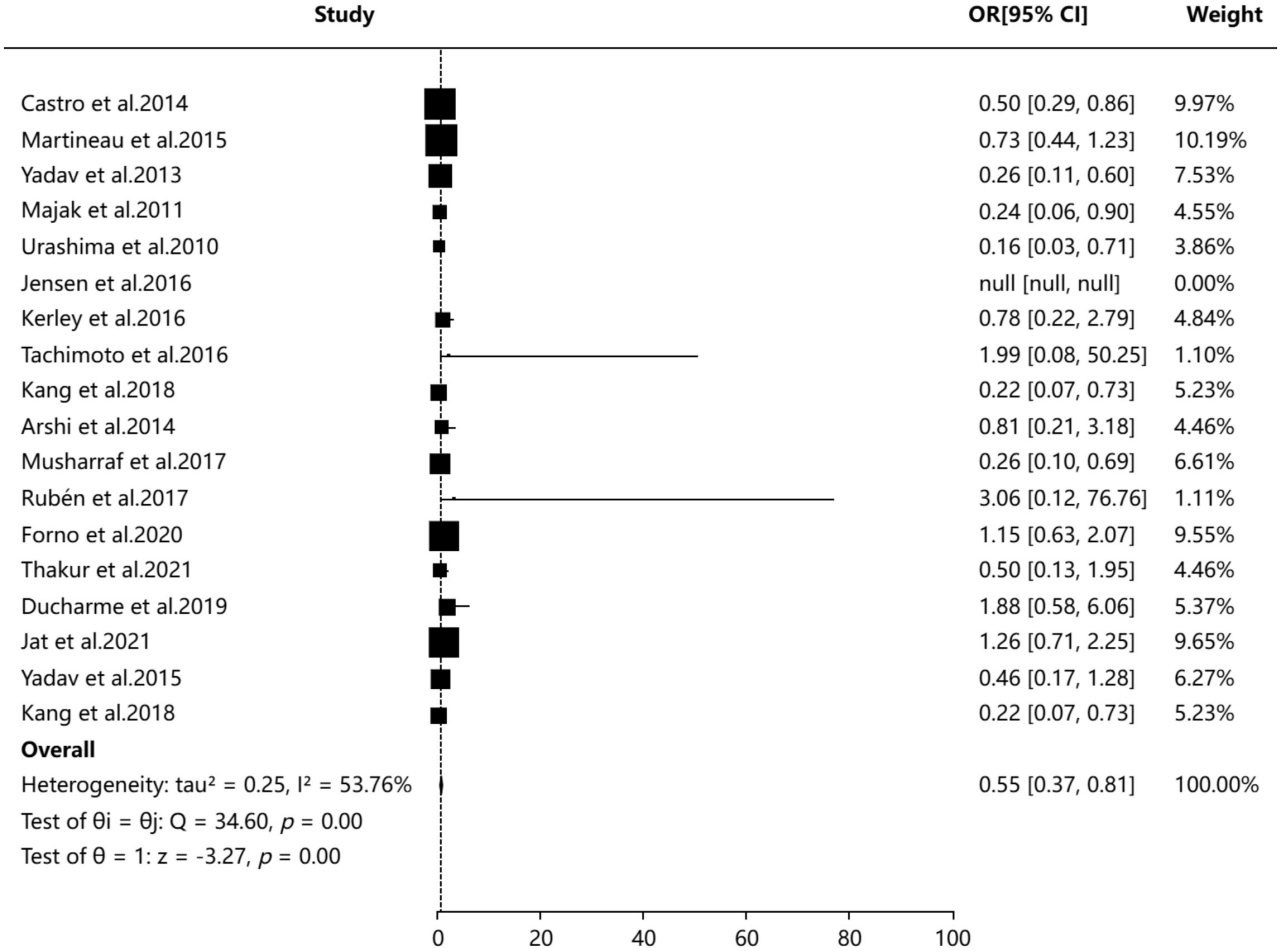
Meta-analysis of asthma exacerbation scores.

15 SRs/MAs (23–25, 27–38, 40, 41) included in the analysis provided quantitative data on asthma exacerbation scores. A random-effects meta-analysis showed that vitamin D supplementation on top of conventional therapy significantly reduced the risk of asthma exacerbation compared with the control group (OR = 0.55, 95% CI [0.37, 0.81], *P* < 0.0001). Moderate heterogeneity was observed in the study (*I*^2^ = 53.76%). Sensitivity analysis (by excluding individual studies one at a time) showed that the pooled effect size was robust, with odds ratios (OR) ranging from 0.51–0.59 and confidence intervals (CI) consistently excluding 1. Cumulative meta-analysis indicated that the effect estimate stabilized as the number of studies increased. Visual inspection of the funnel plot revealed no obvious asymmetry, suggesting a low likelihood of publication bias. Overall, although the pooled results suggested that vitamin D supplementation may be associated with a lower risk of asthma exacerbation, the certainty of evidence from the included studies was mostly low to very low, which limits confidence in this conclusion. Therefore, this finding should be interpreted cautiously and requires confirmation in future high-quality studies. Exploratory subgroup information suggested that this association may be more evident in participants with vitamin D insufficiency or deficiency, pediatric populations, and studies with shorter follow-up duration. Nevertheless, these subgroup findings were not consistently reported across SRs/MAs, and definitions of vitamin D deficiency, exacerbation severity, and follow-up duration varied substantially. Therefore, these observations should be interpreted as exploratory and hypothesis-generating rather than confirmatory. See Figure 6 for detailed results.

### Fractional exhaled nitric oxide (FENO)

**Figure 7:**
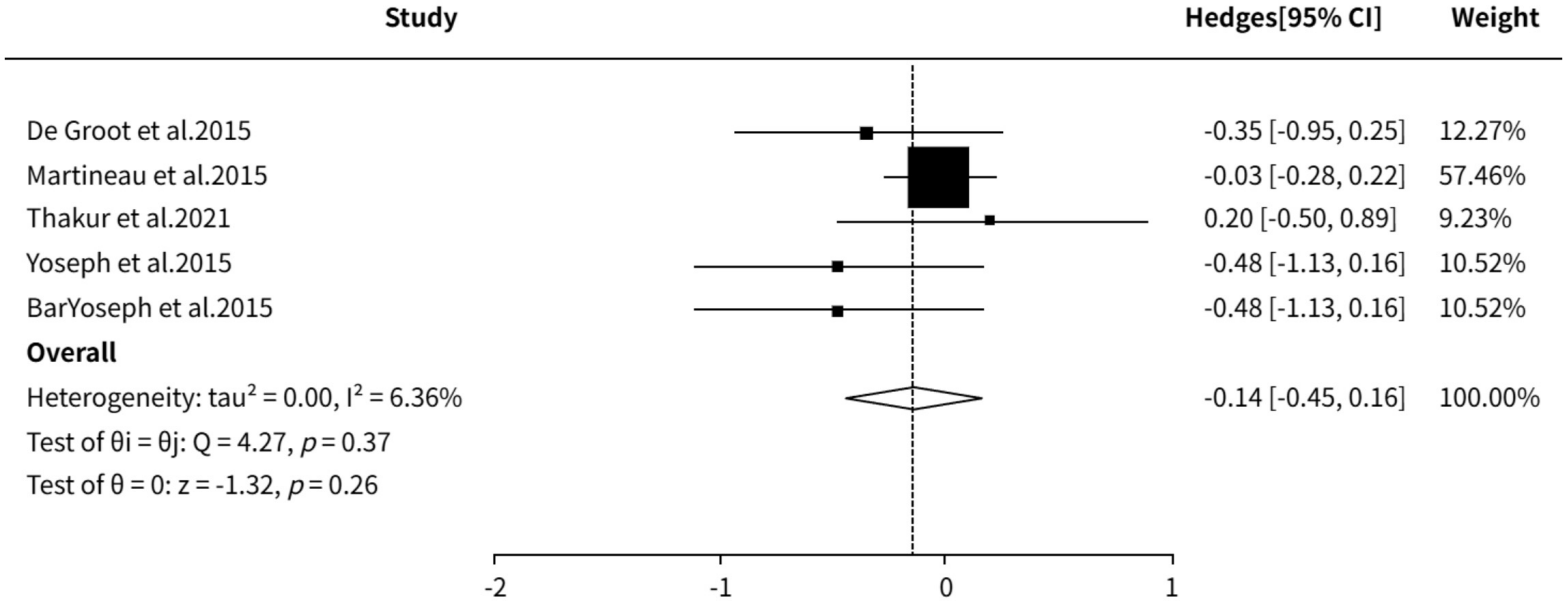
Meta-analysis of FENO scores.

8 SRs/MAs (23, 31, 33–36, 38, 39) were included in the analysis, providing quantitative data on FeNO levels. A random-effects meta-analysis revealed that vitamin D supplementation, compared with the control group on top of conventional treatment, showed no significant difference in reducing exhaled nitric oxide (FeNO) levels (SMD = −0.14, 95% CI [−0.45, 0.16], *P* = 0.26). The heterogeneity among studies was low (*I*^2^ = 6.36%). Sensitivity analysis (by sequentially excluding individual studies) and cumulative meta-analysis both demonstrated that the pooled effect size remained stable and consistent in direction, indicating that the current conclusion is relatively robust. Visual inspection of the funnel plot revealed no obvious signs of publication bias. Based on the available evidence, vitamin D supplementation did not significantly reduce FeNO levels in asthma patients. See Figure 7 for detailed results.

### Forced expiratory volume in 1 second (% predicted) (FEV1%)

**Figure 8:**
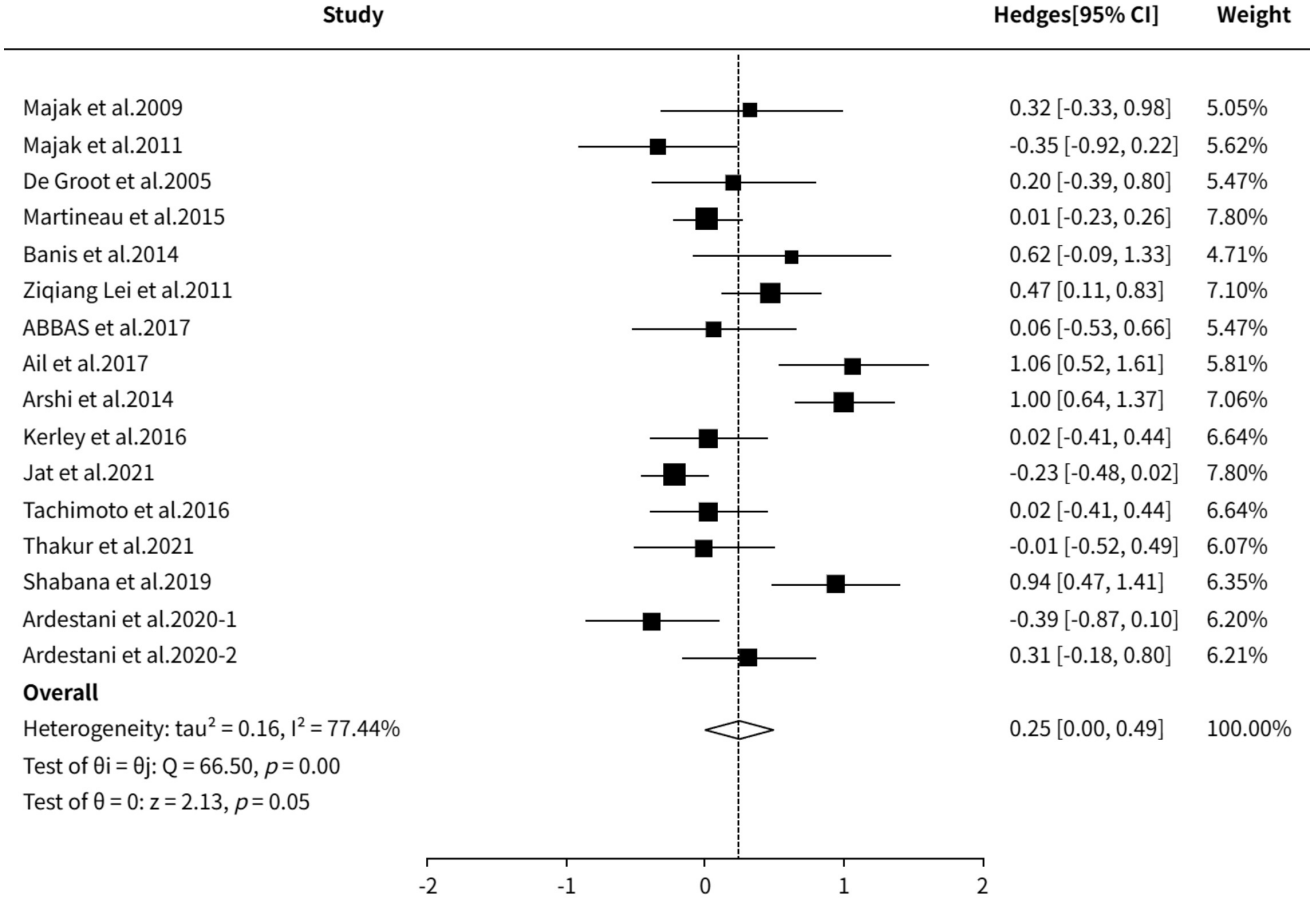
Meta-analysis of FEV1% scores.

17 SRs/MAs (22, 23, 25, 28–38, 40–42) included in the analysis reported quantitative data on FEV1% scores. A random-effects meta-analysis revealed that vitamin D supplementation as an adjunct to conventional treatment showed a marginally statistically significant improvement in FEV1% compared with the control group (SMD = 0.25, 95% CI [0.00, 0.49], *P* = 0.05), suggesting a mild positive trend in lung function associated with vitamin D supplementation. However, a high degree of heterogeneity was observed in the analysis (*I*^2^ = 77.44%). Sensitivity analysis (by removing individual studies one at a time) showed that the pooled effect size remained relatively robust, ranging from 0.18 to 0.29. Cumulative meta-analysis indicated that the effect estimate gradually converged and stabilized as studies accumulated. Funnel plot analysis suggested a potential risk of publication bias. In summary, current evidence indicates that the effect of vitamin D supplementation on improving FEV−1% remains inconclusive, and the quality of evidence is limited by high heterogeneity and potential biases. Future studies are needed to further explore the exact sources of heterogeneity. The substantial heterogeneity observed for FEV1% may be partly attributable to differences across the included SRs/MAs in age, baseline lung function, baseline vitamin D status, dosing strategy, treatment duration, and measurement time points. However, because individual participant data were unavailable, these potential effect modifiers could not be formally tested. Therefore, this interpretation should be considered exploratory. See Figure 8 for detailed results.

### Qualitative analysis

#### Vitamin D levels (serum 25(OH)D)

Current evidence indicates that vitamin D supplementation can safely and effectively increase serum 25(OH)D levels in children with asthma (25, 28, 32); however, its clinical benefits as an adjunct therapy remain uncertain (22, 25). Although observational studies suggest that low vitamin D levels are associated with reduced lung function, increased acute exacerbations, and diminished responsiveness to corticosteroids (23, 41), and despite its theoretical anti-inflammatory and immunomodulatory potential, interventional studies have not consistently demonstrated that supplementation leads to significant improvements in lung function, symptom control, or acute exacerbation rates (23, 25). This may be due to the fact that in most studies, the baseline vitamin D levels of participants were already within the normal range, limiting the potential for further elevation (22, 23). Additionally, some trials used insufficient doses, failing to raise serum concentrations to adequate levels necessary to influence airway pathophysiology. Serum 25(OH)D levels are influenced by multiple factors such as skin pigmentation, season, and latitude (30), and significant heterogeneity exists across studies in terms of deficiency thresholds, measurement methods, and design (32, 34), leading to inconsistent effect estimates. However, monitoring and correcting vitamin D deficiency may positively impact disease control by mitigating small airway obstruction and regulating immune responses, among other mechanisms (26, 38). Notably, age-appropriate recommended dosages may be more effective than ultra-high doses in reducing acute exacerbations, suggesting the existence of an optimal dosage window (40).

#### Forced vital capacity (FVC)

Current research suggests that vitamin D supplementation alone may have limited or even potentially adverse effects on improving lung function (FVC) in children with asthma, particularly among those with higher baseline levels where the scope for further enhancement is more constrained (33). A systematic review further confirms that vitamin D supplementation did not yield statistically or clinically significant improvements in the predicted percentage of FVC at endpoint (37). However, when used as an adjunct intervention alongside inhaled corticosteroids (such as fluticasone propionate), vitamin D may enhance lung volume and respiratory muscle function by synergistically improving airway patency and respiratory mechanics (42).

### Inflammation and immune biomarkers

Although current theories suggest that vitamin D may play a positive role in asthma management by modulating immune cell function and inflammatory pathways, biomarker-based meta-analyses have failed to consistently demonstrate its significant effect (31). Specifically, vitamin D supplementation did not significantly alter local airway inflammatory markers (such as IL-4 and IL-5 in exhaled breath condensate) or systemic markers such as lower airway eosinophil percentage and serum total IgE levels, suggesting that it may not exert a clear regulatory effect on type 2 inflammatory pathways (36, 37). However, some studies have found that vitamin D intervention can significantly increase serum levels of the anti-inflammatory cytokine IL-10 and may selectively modulate certain Th1/Th2-related cytokines, indicating that it might exert potential immunomodulatory effects by enhancing anti-inflammatory immune responses (37, 39). Overall, the existing evidence does not consistently support a clear and broad regulatory effect of vitamin D on asthma-related inflammatory biomarkers. Inconsistent findings may stem from factors such as study design, population heterogeneity, and timing of biomarker assessment (31, 37).

## Discussion

### Efficacy and safety of vitamin D in the treatment of asthma

This umbrella review provides a comprehensive synthesis of published systematic reviews and meta-analyses on vitamin D supplementation for asthma. Overall, vitamin D supplementation was associated with a lower risk of asthma exacerbations and did not appear to increase adverse events. However, evidence for improvements in daily symptom control, lung function, and inflammatory outcomes was limited or inconsistent. These findings suggest that vitamin D may have a more specific role in reducing exacerbation risk than in improving routine clinical or physiological measures of asthma control. Nevertheless, this interpretation should remain cautious because many outcomes were supported by low or very low certainty evidence, and substantial overlap existed among the primary studies included in the reviewed systematic reviews. The potential effect of vitamin D on asthma exacerbations is biologically plausible. Previous studies have suggested that vitamin D may influence asthma-related outcomes through immune regulation and respiratory protection (43, 44), enhancement of innate airway defense (45–48), modulation of airway inflammation and oxidative stress (49–51), regulation of Th17/Treg-related immune balance (45), improvement of glucocorticoid responsiveness and other immune-related responses (52–55), and potential effects on airway remodeling (56, 57). These mechanisms may help explain why vitamin D supplementation was more consistently associated with reduced exacerbation risk than with improvements in daily symptom control, lung function, or inflammatory biomarkers. However, the inconsistent clinical findings suggest that these biological effects may not translate into broad benefits for all patients with asthma. Differences in baseline vitamin D status, age, asthma phenotype, disease severity, dose, and treatment duration may partly explain the variation in findings across reviews.

It is worth noting that, despite the statistically significant findings for asthma exacerbation outcomes, the GRADE assessment indicated that most of the relevant evidence was of low or very low certainty, primarily due to limitations such as risk of bias in the original studies, heterogeneity across the included studies, and imprecision of effect estimates. Therefore, the current findings should be regarded as exploratory or hypothesis-generating evidence and are insufficient to support strong recommendations in clinical practice.

#### Heterogeneity and effect modifiers

Heterogeneity remained an important limitation of the current evidence. The included systematic reviews and meta-analyses differed in eligibility criteria, study populations, baseline vitamin D status, intervention dose, treatment duration, outcome definitions, and follow-up time. Although sensitivity analyses suggested that the main pooled estimates were generally stable, the sources of heterogeneity could not be fully explained. This was partly because most included reviews reported aggregate-level rather than individual participant data, which limited our ability to formally examine interactions among potential effect modifiers. Therefore, findings related to age, baseline vitamin D deficiency, asthma severity, dosing strategy, and treatment duration should be interpreted as exploratory rather than confirmatory. Future studies should use individual participant data meta-analysis or well-designed randomized trials to identify patients most likely to benefit from vitamin D supplementation.

In terms of safety, current evidence indicates that vitamin D supplementation at conventional doses is generally well tolerated and shows no significant difference compared with placebo in the incidence of serious adverse events, hypercalcemia, or kidney stones (58). Serum calcium and 25(OH)D levels generally remained within the safe range after supplementation, with no reported cases of toxicity (59). Although experimental studies have reported no obvious adverse effects even at higher doses (49, 56), the clinical interpretation of safety should still consider differences in dose, follow-up duration, and adverse-event reporting across studies. While physiological supplementation is relatively low-cost and safe, there is currently no consensus supporting its use as a routine immunomodulatory intervention for all patients with asthma (45). Some clinical studies have also shown that correcting vitamin D deficiency does not necessarily lead to direct improvement in asthma control (59, 60). Therefore, vitamin D supplementation may be more appropriate for selected clinical scenarios, such as patients with vitamin D deficiency or those at higher risk of exacerbations, rather than for widespread use in daily symptom control. From a public health perspective, its potential value may be greater if future studies confirm reductions in exacerbations, healthcare utilization, or related socioeconomic burden (61). Future research should use individual participant data meta-analysis or well-designed randomized trials to identify optimal target populations, dosing regimens, and treatment strategies (43, 45), and to further explore whether vitamin D analogs can improve efficacy and safety (52).

### Evaluation of literature quality

The methodological and reporting assessments provided important context for interpreting the current evidence. According to the ROBIS assessment, most included systematic reviews and meta-analyses were judged to be at low risk of bias, suggesting that the overall review process was generally reliable. However, several limitations were still identified in study identification and selection, data collection, and study appraisal. The AMSTAR-2 assessment also showed that although some reviews were methodologically robust, important shortcomings remained, particularly in prospective protocol registration, reporting of funding sources and conflicts of interest, and consideration of how risk of bias in primary studies might affect the interpretation of pooled findings.

The PRISMA 2020 assessment further indicated that reporting completeness varied across the included reviews, especially regarding protocol availability, certainty of evidence assessment, and access to data, code, or other supporting materials. Consistent with these reporting and methodological limitations, the GRADE assessment showed that many outcome-specific bodies of evidence were rated as low or very low certainty, mainly because of risk of bias, inconsistency, and imprecision. These findings suggest that future systematic reviews and meta-analyses on vitamin D supplementation for asthma should prospectively register protocols, follow PRISMA 2020 reporting standards, transparently report funding and conflicts of interest, and explicitly incorporate risk of bias and certainty of evidence into the interpretation of clinical conclusions.

### Limitations

This study has several limitations: (1) Differences in search strategies, inclusion criteria, and data analysis methods across the included SRs/MAs may have introduced selection bias and affected the accuracy of evidence synthesis. (2) Primary study overlap was observed among the included reviews (CCA = 10.5%), which may have led to indirect double-counting and influenced the pooled estimates or the apparent strength of evidence, despite the use of overlap-handling procedures and cautious interpretation. (3) The certainty of evidence for many outcomes was limited by methodological weaknesses, risk of bias, heterogeneity, and imprecision, which restricts the clinical generalizability of the findings. Therefore, the current conclusions should be regarded mainly as hypothesis-generating. (4) The sources of heterogeneity could not be fully identified because most included reviews reported aggregate-level rather than individual participant data. Potential effect modifiers, such as age, baseline 25(OH)D status, asthma severity, dose, and treatment duration, could only be summarized narratively and should not be interpreted as confirmatory subgroup effects.

## Conclusions

This umbrella review suggests that vitamin D supplementation may reduce asthma exacerbations and appears to be safe as an adjunctive treatment. However, current evidence does not consistently support improvements in daily symptom control, lung function, or inflammatory outcomes. Because many outcomes were supported by low or very low certainty evidence and primary study overlap was present, these findings should be interpreted cautiously and do not support universal vitamin D supplementation for all patients with asthma. Future high-quality randomized trials and individual participant data meta-analyses are needed to identify responsive subgroups and clarify optimal dose, duration, and biomarker-guided supplementation strategies.

## Data Availability

The original contributions presented in the study are included in the article/supplementary material, further inquiries can be directed to the corresponding authors.
