# Supplementary material for: Efficacy and safety of vitamin D in the treatment of asthma: an overview of systematic reviews and meta-analyses
Source: Front Med (Lausanne). 2026 May 26;13:1783005. doi: 10.3389/fmed.2026.1783005 (PMC13246613; doi:10.3389/fmed.2026.1783005)
Supplement: Supplementary file 1 [file Table_1.docx]

Research Process and Research Data

Contents

[1.Search Query and Search Logic 2](#_Toc24073)

[2.ROBIS 5](#_Toc18260)

[3.AMSTAR-2 16](#_Toc7613)

[4.PRISMA 2020 41](#_Toc27087)

[5.GRADE 115](#_Toc17699)

[6.Citation Overlap Matrix and the Corrected Covered Area (CCA). 133](#_Toc11321)

[7.Literature Screening： 135](#_Toc9394)

**1.Search Query and Search Logic**

PubMed

(((Asthmas[MeSH Terms]) OR ((((((((((((Asthma, Bronchial[Title/Abstract]) OR (Bronchial Asthma[Title/Abstract])) OR (Allergic Asthma[Title/Abstract])) OR (Exercise-Induced Bronchospasm[Title/Abstract])) OR (Occupational Asthma[Title/Abstract])) OR (Severe Asthma[Title/Abstract])) OR (Refractory Asthma[Title/Abstract])) OR (Childhood-Onset Asthma[Title/Abstract])) OR (Pediatric Asthma[Title/Abstract])) OR (Adult-Onset Asthma[Title/Abstract])) OR (Cough-Variant Asthma[Title/Abstract])) OR (Eosinophilic Asthma[Title/Abstract]))) AND ((Vitamin D[MeSH Terms]) OR ((((((((Ergocalciferols[Title/Abstract]) OR (Cholecalciferol[Title/Abstract])) OR (Calcifediol[Title/Abstract])) OR (Calcitriol[Title/Abstract])) OR (Hydroxycholecalciferols[Title/Abstract])) OR (Dihydroxycholecalciferols[Title/Abstract])) OR (Vitamin D-Binding Protein[Title/Abstract])) OR (1,25-dihydroxyvitamin D[Title/Abstract])))) AND (((((Meta-Analysis[MeSH Terms]) OR (((meta-analysis[Title/Abstract]) OR (metaanalysis[Title/Abstract])) OR (meta analysis[Title/Abstract]))) OR ((review, systematic[Title/Abstract]) OR (systematic review[Title/Abstract]))) OR (Systematic Review[MeSH Terms]))).

Embase

1:'asthma'/exp

2:('asthma bronchiale' OR 'asthma pulmonale' OR 'asthma, bronchial' OR 'asthmatic' OR 'asthmatic subject' OR 'bronchial asthma' OR 'bronchus asthma' OR 'chronic asthma' OR 'lung allergy' OR 'asthma'):ti,ab,kw

3:'vitamin d'/exp

4:('vitamin D' OR 'Ergocalciferols' OR 'Cholecalciferol' OR 'Calcifediol' OR 'Calcitriol' OR 'Dihydroxycholecalciferols' OR 'Hydroxycholecalciferols' OR 'Vitamin D-Binding Protein' OR '1,25-dihydroxyvitamin D'):ti,ab,kw

5:'meta analysis'/exp

6:('analysis, meta' OR 'meta-analysis' OR 'metaanalysis' OR 'meta analysis'):ti,ab,kw

7:'systematic review'/exp

8:('review, systematic' OR 'systematic review'):ti,ab,kw

9:#1 OR #2

10:#3 OR #4

11:#5 OR #6 OR #7 OR #8

12:#9 AND #10 AND #11

Cochrane Library

1:MeSH descriptor: [Asthma] explode all trees

2:('asthma bronchiale' OR 'asthma pulmonale' OR 'asthma, bronchial' OR 'asthmatic' OR 'asthmatic subject' OR 'bronchial asthma' OR 'bronchus asthma' OR 'chronic asthma' OR 'lung allergy' OR 'asthma'):ti,ab,kw

3:MeSH descriptor: [Vitamin D] explode all trees

4:('vitamin D' OR 'Ergocalciferols' OR 'Cholecalciferol' OR 'Calcifediol' OR 'Calcitriol' OR 'Dihydroxycholecalciferols' OR 'Hydroxycholecalciferols' OR 'Vitamin D-Binding Protein'):ti,ab,kw

5:MeSH descriptor: [Meta-Analysis] explode all trees

6:('analysis, meta' OR 'meta-analysis' OR 'metaanalysis' OR 'meta analysis'):ti,ab,kw

7:MeSH descriptor: [Systematic Review] explode all trees

8:('review, systematic' OR 'systematic review'):ti,ab,kw

9:#1 OR #2

10:#3 OR #4

11:#5 OR #6 OR #7 OR #8

12:#9 AND #10 AND #11

Web of Science

1:TS=(asthma) OR TS=(Asthmas) OR TS=(Asthma, Bronchial) OR TS=(Bronchial Asthma) OR TS=(asthma bronchiale) OR TS=(asthma pulmonale) OR TS=(asthma, bronchial) OR TS=(asthmatic) OR TS=(asthmatic subject) OR TS=(bronchial asthma) OR TS=(bronchus asthma) OR TS=(lung allergy) OR TS=(asthma) OR TS=(chronic asthma)

2:TS=(vitamin d) OR TS=(vitamin D) OR TS=(Ergocalciferols) OR TS=(Cholecalciferol) OR TS=(Calcifediol) OR TS=(Calcitriol) OR TS=(Dihydroxycholecalciferols) OR TS=(Hydroxycholecalciferols) OR TS=(Vitamin D-Binding Protein) OR TS=(1,25-dihydroxyvitamin D)

3:TS=(meta analysis) OR TS=(analysis, meta) OR TS=(meta-analysis) OR TS=(metaanalysis') OR TS=(meta analysis) OR TS=(systematic review) OR TS=(review, systematic) OR TS=(systematic review)

4:#1 AND #2 AND #3

CNKI

(SU=维生素 OR SU=维生素d OR SU='25-羟维生素' OR SU=25羟维生素 OR SU=羟基维生素d OR SU='25-羟维生素d' OR SU=羟维生素 OR SU=羟维生素d OR SU=羟基维生素) AND (SU=哮喘 OR SU=哮喘病 OR SU=哮喘患者 OR SU=支气管哮喘 OR SU=哮喘控制 OR SU=哮喘发作) AND (SU=meta OR SU=meta分析 OR SU='meta-analysis' OR SU='meta-分析' OR SU=meta分析法 OR SU=系统评价 OR SU=系统评价方法 OR SU=系统评价研究 OR SU=系统评价和meta分析 OR SU='系统评价/meta分析' OR SU=系统评价与meta分析)

VIP

(M=维生素 OR M=维生素d OR M='25-羟维生素' OR M=25羟维生素 OR M=羟基维生素d OR M='25-羟维生素d' OR M=羟维生素 OR M=羟维生素d OR M=羟基维生素) AND (M=哮喘 OR M=哮喘病 OR M=哮喘患者 OR M=支气管哮喘 OR M=哮喘控制 OR M=哮喘发作) AND (M=meta OR M=meta分析 OR M='meta-analysis' OR M='meta-分析' OR M=meta分析法 OR M=系统评价 OR M=系统评价方法 OR M=系统评价研究 OR M=系统评价和meta分析 OR M='系统评价/meta分析' OR M=系统评价与meta分析)

WANFANG

主题:(维生素 or 维生素d or '25-羟维生素' or 25羟维生素 or 羟基维生素d or '25-羟维生素d' or 羟维生素 or 羟维生素d or 羟基维生素) and 主题:(哮喘 or 哮喘病 or 哮喘患者 or 支气管哮喘 or 哮喘控制 or 哮喘发作) and 主题:(meta or meta分析 or 'meta-analysis' or 'meta-分析' or meta分析法 or 系统评价 or 系统评价方法 or 系统评价研究 or 系统评价和meta分析 or '系统评价/meta分析' or 系统评价与meta分析)

CBM

1:"维生素D"[不加权:扩展]

2:"维生素D"[中文标题:智能] OR "骨化醇类"[中文标题:智能] OR "胆骨化醇"[中文标题:智能] OR "羟基胆骨化醇类"[中文标题:智能] OR "麦角固醇"[中文标题:智能] OR "二羟胆钙化醇类"[中文标题:智能] OR "骨化二醇"[中文标题:智能]

3:"哮喘"[不加权:扩展]

4:"哮喘"[中文标题:智能] OR "哮喘持续状态"[中文标题:智能] OR "气道重塑"[中文标题:智能]

5:"Meta分析"[不加权:扩展]

6:"系统评价(主题)"[不加权:扩展]

7:"Meta分析"[中文标题:智能] OR "网络Meta分析"[中文标题:智能] OR "系统评价"[中文标题:智能]

8:(#2) OR (#1)

9:(#4) OR (#3)

10:(#7) OR (#6) OR (#5)

11:(#10) AND (#9) AND (#8)

2.ROBIS

P: Pass. F: Fail. L: Low risk of bias. H: High risk of bias. Un: Unclear risk of bias.

A:The conclusions of researcher Ph.D. Yongxiu Liu.

B:The conclusions of researcher Ph.D. Yuguo Li.

C:In case of a difference of opinions, it shall be adjudicated by Professor Lei Gao.

D:Conclusive conclusion.

Study 22：Fares, Munes M et al. “Vitamin D supplementation in children with asthma: a systematic review and meta-analysis.” BMC research notes vol. 8 23. 3 Feb. 2015, doi:10.1186/s13104-014-0961-3.PMID: 25643669 PMCID: PMC4328422

| Phase | | A | B | C | D |
| --- | --- | --- | --- | --- | --- |
| Phase 1: Assessing Relevance | | P | P | - | P |
| Phase 2: Identifying Concerns with Review Process | Study Eligibility Criteria | L | L | - | L |
|  | Identification and Selection of Studies | L | L | - | L |
|  | Data Collection and Study Appraisal | L | L | - | L |
|  | Synthesis and Findings | L | L | - | L |
| Phase 3: Judging Risk of Bias | | L | L | - | L |

ROBIS

P: Pass. F: Fail. L: Low risk of bias. H: High risk of bias. Un: Unclear risk of bias.

A:The conclusions of researcher Ph.D. Yongxiu Liu.

B:The conclusions of researcher Ph.D. Yuguo Li

C:In case of a difference of opinions, it shall be adjudicated by Professor Lei Gao

D:Conclusive conclusion.

Study 23：Luo, Jian et al. “Can Vitamin D Supplementation in Addition to Asthma Controllers Improve Clinical Outcomes in Patients With Asthma?: A Meta-Analysis.” Medicine vol. 94,50 (2015): e2185. doi:10.1097/MD.0000000000002185.PMID: 26683927 PMCID: PMC5058899

| Phase | | A | B | C | D |
| --- | --- | --- | --- | --- | --- |
| Phase 1: Assessing Relevance | | P | P | - | P |
| Phase 2: Identifying Concerns with Review Process | Study Eligibility Criteria | L | L | - | L |
|  | Identification and Selection of Studies | L | UN | UN | UN |
|  | Data Collection and Study Appraisal | L | L | - | L |
|  | Synthesis and Findings | L | L | - | L |
| Phase 3: Judging Risk of Bias | | L | L | - | L |

ROBIS

P: Pass. F: Fail. L: Low risk of bias. H: High risk of bias. Un: Unclear risk of bias.

A:The conclusions of researcher Ph.D. Yongxiu Liu.

B:The conclusions of researcher Ph.D. Yuguo Li.

C:In case of a difference of opinions, it shall be adjudicated by Professor Lei Gao.

D:Conclusive conclusion.

Study 24：Pojsupap, Supichaya et al. “Efficacy of high-dose vitamin D in pediatric asthma: a systematic review and meta-analysis.” The Journal of asthma : official journal of the Association for the Care of Asthma vol. 52,4 (2015): 382-90. doi:10.3109/02770903.2014.980509.PMID: 25365192

| Phase | | A | B | C | D |
| --- | --- | --- | --- | --- | --- |
| Phase 1: Assessing Relevance | | P | P | - | P |
| Phase 2: Identifying Concerns with Review Process | Study Eligibility Criteria | L | L | - | L |
|  | Identification and Selection of Studies | L | UN | L | L |
|  | Data Collection and Study Appraisal | L | L | - | L |
|  | Synthesis and Findings | L | L | - | L |
| Phase 3: Judging Risk of Bias | | L | L | - | L |

ROBIS

P: Pass. F: Fail. L: Low risk of bias. H: High risk of bias. Un: Unclear risk of bias.

A:The conclusions of researcher Ph.D. Yongxiu Liu.

B:The conclusions of researcher Ph.D.Yuguo Li.

C:In case of a difference of opinions, it shall be adjudicated by Professor Lei Gao.

D:Conclusive conclusion.

Study 25：Riverin, Bruno D et al. “Vitamin D Supplementation for Childhood Asthma: A Systematic Review and Meta-Analysis.” PloS one vol. 10,8 e0136841. 31 Aug. 2015, doi:10.1371/journal.pone.0136841.PMID: 26322509 PMCID: PMC4556456

| Phase | | A | B | C | D |
| --- | --- | --- | --- | --- | --- |
| Phase 1: Assessing Relevance | | P | P | - | P |
| Phase 2: Identifying Concerns with Review Process | Study Eligibility Criteria | L | L | - | L |
|  | Identification and Selection of Studies | L | UN | L | L |
|  | Data Collection and Study Appraisal | L | L | - | L |
|  | Synthesis and Findings | L | L | - | L |
| Phase 3: Judging Risk of Bias | | L | L | - | L |

ROBIS

P: Pass. F: Fail. L: Low risk of bias. H: High risk of bias. Un: Unclear risk of bias.

A:The conclusions of researcher Ph.D. Yongxiu Liu.

B:The conclusions of researcher Ph.D.Yuguo Li.

C:In case of a difference of opinions, it shall be adjudicated by Professor Lei Gao.

D:Conclusive conclusion.

Study 26：景伟超,刘璐佳,关洋洋,等.维生素D辅助治疗儿童哮喘Meta分析[J].世界中西医结合杂志,2017,12(10):1341-1344+1354.DOI:10.13935/j.cnki.sjzx.171003.

| Phase | | A | B | C | D |
| --- | --- | --- | --- | --- | --- |
| Phase 1: Assessing Relevance | | P | P | - | P |
| Phase 2: Identifying Concerns with Review Process | Study Eligibility Criteria | L | L | - | L |
|  | Identification and Selection of Studies | UN | H | H | H |
|  | Data Collection and Study Appraisal | H | H | - | H |
|  | Synthesis and Findings | L | UN | L | L |
| Phase 3: Judging Risk of Bias | | H | H | - | H |

ROBIS

P: Pass. F: Fail. L: Low risk of bias. H: High risk of bias. Un: Unclear risk of bias.

A:The conclusions of researcher Ph.D. Yongxiu Liu.

B:The conclusions of researcher Ph.D. Yuguo Li.

C:In case of a difference of opinions, it shall be adjudicated by Professor Lei Gao.

D:Conclusive conclusion.

Study 27：Jolliffe, David A et al. “Vitamin D supplementation to prevent asthma exacerbations: a systematic review and meta-analysis of individual participant data.” The Lancet. Respiratory medicine vol. 5,11 (2017): 881-890. doi:10.1016/S2213-2600(17)30306-5.PMID: 28986128 PMCID: PMC5693329

| Phase | | A | B | C | D |
| --- | --- | --- | --- | --- | --- |
| Phase 1: Assessing Relevance | | P | P | - | P |
| Phase 2: Identifying Concerns with Review Process | Study Eligibility Criteria | L | L | - | L |
|  | Identification and Selection of Studies | L | L | - | L |
|  | Data Collection and Study Appraisal | L | L | - | L |
|  | Synthesis and Findings | L | L | - | L |
| Phase 3: Judging Risk of Bias | | L | L | - | L |

ROBIS

P: Pass. F: Fail. L: Low risk of bias. H: High risk of bias. Un: Unclear risk of bias.

A:The conclusions of researcher Ph.D. Yongxiu Liu.

B:The conclusions of researcher Ph.D. Yuguo Li.

C:In case of a difference of opinions, it shall be adjudicated by Professor Lei Gao.

D:Conclusive conclusion.

Study 28：郝宏霞.维生素D在缓解期哮喘患者治疗中的有效性和安全性meta分析[D].山西医科大学,2018.

| Phase | | A | B | C | D |
| --- | --- | --- | --- | --- | --- |
| Phase 1: Assessing Relevance | | P | P | - | P |
| Phase 2: Identifying Concerns with Review Process | Study Eligibility Criteria | L | L | - | L |
|  | Identification and Selection of Studies | L | L | - | L |
|  | Data Collection and Study Appraisal | L | L | - | L |
|  | Synthesis and Findings | L | L | - | L |
| Phase 3: Judging Risk of Bias | | L | L | - | L |

ROBIS

P: Pass. F: Fail. L: Low risk of bias. H: High risk of bias. Un: Unclear risk of bias.

A:The conclusions of researcher Ph.D. Yongxiu Liu.

B:The conclusions of researcher Ph.D. Yuguo Li.

C:In case of a difference of opinions, it shall be adjudicated by Professor Lei Gao.

D:Conclusive conclusion.

Study 29：田超,史强,赵紫楠,等.维生素D补充剂对儿童支气管哮喘获益相关性的系统评价和meta分析[J].临床药物治疗杂志,2018,16(04):66-70.

| Phase | | A | B | C | D |
| --- | --- | --- | --- | --- | --- |
| Phase 1: Assessing Relevance | | P | P | - | P |
| Phase 2: Identifying Concerns with Review Process | Study Eligibility Criteria | L | L | - | L |
|  | Identification and Selection of Studies | L | UN | L | L |
|  | Data Collection and Study Appraisal | L | L | - | L |
|  | Synthesis and Findings | L | UN | L | L |
| Phase 3: Judging Risk of Bias | | L | UN | L | L |

ROBIS

P: Pass. F: Fail. L: Low risk of bias. H: High risk of bias. Un: Unclear risk of bias.

A:The conclusions of researcher Ph.D. Yongxiu Liu.

B:The conclusions of researcher Ph.D. Yuguo Li.

C:In case of a difference of opinions, it shall be adjudicated by Professor Lei Gao.

D:Conclusive conclusion.

Study 30：郝畅.维生素D补充与儿童哮喘的系统评价及meta分析[D].重庆医科大学,2019.

| Phase | | A | B | C | D |
| --- | --- | --- | --- | --- | --- |
| Phase 1: Assessing Relevance | | P | P | - | P |
| Phase 2: Identifying Concerns with Review Process | Study Eligibility Criteria | L | L | - | L |
|  | Identification and Selection of Studies | UN | L | UN | UN |
|  | Data Collection and Study Appraisal | L | L | - | L |
|  | Synthesis and Findings | L | UN | UN | UN |
| Phase 3: Judging Risk of Bias | | L | L | - | L |

ROBIS

P: Pass. F: Fail. L: Low risk of bias. H: High risk of bias. Un: Unclear risk of bias.

A:The conclusions of researcher Ph.D. Yongxiu Liu.

B:The conclusions of researcher Ph.D. Yuguo Li.

C:In case of a difference of opinions, it shall be adjudicated by Professor Lei Gao.

D:Conclusive conclusion.

Study 31：Wang, Mingming et al. “Association between vitamin D status and asthma control: A meta-analysis of randomized trials.”Respiratory medicine vol. 150 (2019): 85-94. doi:10.1016/j.rmed.2019.02.016.PMID: 30961957

| Phase | | A | B | C | D |
| --- | --- | --- | --- | --- | --- |
| Phase 1: Assessing Relevance | | P | P | - | P |
| Phase 2: Identifying Concerns with Review Process | Study Eligibility Criteria | L | L | - | L |
|  | Identification and Selection of Studies | L | L | - | L |
|  | Data Collection and Study Appraisal | L | L | - | L |
|  | Synthesis and Findings | L | L | - | L |
| Phase 3: Judging Risk of Bias | | L | L | - | L |

ROBIS

P: Pass. F: Fail. L: Low risk of bias. H: High risk of bias. Un: Unclear risk of bias.

A:The conclusions of researcher Ph.D. Yongxiu Liu.

B:The conclusions of researcher Ph.D.Yuguo Li.

C:In case of a difference of opinions, it shall be adjudicated by Professor Lei Gao.

D:Conclusive conclusion.

Study 32：Chen, Ziyu et al. “Vitamin D can safely reduce asthma exacerbations among corticosteroid-using children and adults with asthma: a systematic review and meta-analysis of randomized controlled trials.” Nutrition research (New York, N.Y.) vol. 92 (2021): 49-61. doi:10.1016/j.nutres.2021.05.010.PMID: 34274554

| Phase | | A | B | C | D |
| --- | --- | --- | --- | --- | --- |
| Phase 1: Assessing Relevance | | P | P | - | P |
| Phase 2: Identifying Concerns with Review Process | Study Eligibility Criteria | L | L | - | L |
|  | Identification and Selection of Studies | L | L | - | L |
|  | Data Collection and Study Appraisal | L | L | - | L |
|  | Synthesis and Findings | L | L | - | L |
| Phase 3: Judging Risk of Bias | | L | L | - | L |

ROBIS

P: Pass. F: Fail. L: Low risk of bias. H: High risk of bias. Un: Unclear risk of bias.

A:The conclusions of researcher Ph.D. Yongxiu Liu.

B:The conclusions of researcher Ph.D. Yuguo Li.

C:In case of a difference of opinions, it shall be adjudicated by Professor Lei Gao.

D:Conclusive conclusion.

Study 33：Hao, Meiqi et al. “The Effect of Vitamin D Supplementation in Children With Asthma: A Meta-Analysis.”Frontiers in pediatrics vol. 10 840617. 29 Jun. 2022, doi:10.3389/fped.2022.840617.PMID: 35844729 PMCID: PMC9277022

| Phase | | A | B | C | D |
| --- | --- | --- | --- | --- | --- |
| Phase 1: Assessing Relevance | | P | P | - | P |
| Phase 2: Identifying Concerns with Review Process | Study Eligibility Criteria | L | L | - | L |
|  | Identification and Selection of Studies | L | L | - | L |
|  | Data Collection and Study Appraisal | L | L | - | L |
|  | Synthesis and Findings | L | UN | L | L |
| Phase 3: Judging Risk of Bias | | L | UN | L | L |

ROBIS

P: Pass. F: Fail. L: Low risk of bias. H: High risk of bias. Un: Unclear risk of bias.

A:The conclusions of researcher Ph.D. Yongxiu Liu.

B:The conclusions of researcher Ph.D. Yuguo Li.

C:In case of a difference of opinions, it shall be adjudicated by Professor Lei Gao.

D:Conclusive conclusion.

Study 34：Kumar, Jogender et al. “Vitamin D supplementation in childhood asthma: a systematic review and meta-analysis of randomised controlled trials.” ERJ open research vol. 8,1 00662-2021. 7 Feb. 2021, doi:10.1183/23120541.00662-2021.PMID: 35141325 PMCID: PMC8819253

| Phase | | A | B | C | D |
| --- | --- | --- | --- | --- | --- |
| Phase 1: Assessing Relevance | | P | P | - | P |
| Phase 2: Identifying Concerns with Review Process | Study Eligibility Criteria | L | L | - | L |
|  | Identification and Selection of Studies | L | L | - | L |
|  | Data Collection and Study Appraisal | L | L | - | L |
|  | Synthesis and Findings | L | L | - | L |
| Phase 3: Judging Risk of Bias | | L | L | - | L |

ROBIS

P: Pass. F: Fail. L: Low risk of bias. H: High risk of bias. Un: Unclear risk of bias.

A:The conclusions of researcher Ph.D. Yongxiu Liu.

B:The conclusions of researcher Ph.D. Yuguo Li.

C:In case of a difference of opinions, it shall be adjudicated by Professor Lei Gao.

D:Conclusive conclusion.

Study 35：Liu, Meiqi et al. “A Meta-Analysis on Vitamin D Supplementation and Asthma Treatment.” Frontiers in nutrition vol. 9 860628. 6 Jul. 2022, doi:10.3389/fnut.2022.860628.PMID: 35873428 PMCID: PMC9300755

| Phase | | A | B | C | D |
| --- | --- | --- | --- | --- | --- |
| Phase 1: Assessing Relevance | | P | P | - | P |
| Phase 2: Identifying Concerns with Review Process | Study Eligibility Criteria | L | L | - | L |
|  | Identification and Selection of Studies | L | UN | L | L |
|  | Data Collection and Study Appraisal | L | L | - | L |
|  | Synthesis and Findings | L | UN | L | L |
| Phase 3: Judging Risk of Bias | | L | UN | L | L |

ROBIS

P: Pass. F: Fail. L: Low risk of bias. H: High risk of bias. Un: Unclear risk of bias.

A:The conclusions of researcher Ph.D. Yongxiu Liu.

B:The conclusions of researcher Ph.D.Yuguo Li.

C:In case of a difference of opinions, it shall be adjudicated by Professor Lei Gao.

D:Conclusive conclusion.

Study 36：Nitzan, Itamar et al. “Vitamin D and Asthma: a Systematic Review of Clinical Trials.” Current nutrition reports vol. 11,2 (2022): 311-317. doi:10.1007/s13668-022-00411-6.PMID: 35347665

| Phase | | A | B | C | D |
| --- | --- | --- | --- | --- | --- |
| Phase 1: Assessing Relevance | | P | P | - | P |
| Phase 2: Identifying Concerns with Review Process | Study Eligibility Criteria | L | L | - | L |
|  | Identification and Selection of Studies | L | L | - | L |
|  | Data Collection and Study Appraisal | UN | UN | - | UN |
|  | Synthesis and Findings | L | UN | L | L |
| Phase 3: Judging Risk of Bias | | L | UN | UN | UN |

ROBIS

P: Pass. F: Fail. L: Low risk of bias. H: High risk of bias. Un: Unclear risk of bias.

A:The conclusions of researcher Ph.D. Yongxiu Liu.

B:The conclusions of researcher Ph.D. Yuguo Li.

C:In case of a difference of opinions, it shall be adjudicated by Professor Lei Gao.

D:Conclusive conclusion.

Study 37：Williamson, Anne et al. “Vitamin D for the management of asthma.” The Cochrane database of systematic reviews vol. 2,2 CD011511. 6 Feb. 2023, doi:10.1002/14651858.CD011511.pub3.PMID: 36744416 PMCID: PMC9899558

| Phase | | A | B | C | D |
| --- | --- | --- | --- | --- | --- |
| Phase 1: Assessing Relevance | | P | P | - | P |
| Phase 2: Identifying Concerns with Review Process | Study Eligibility Criteria | L | L | - | L |
|  | Identification and Selection of Studies | L | L | - | L |
|  | Data Collection and Study Appraisal | L | L | - | L |
|  | Synthesis and Findings | L | L | - | L |
| Phase 3: Judging Risk of Bias | | L | L | - | L |

ROBIS

P: Pass. F: Fail. L: Low risk of bias. H: High risk of bias. Un: Unclear risk of bias.

A:The conclusions of researcher Ph.D. Yongxiu Liu.

B:The conclusions of researcher Ph.D. Yuguo Li.

C:In case of a difference of opinions, it shall be adjudicated by Professor Lei Gao.

D:Conclusive conclusion.

Study 38：孙倩.补充维生素D及其类似物对支气管哮喘的影响：一项随机对照试验的荟萃分析[D].南昌大学,2024.DOI:10.27232/d.cnki.gnchu.2024.003676.

| Phase | | A | B | C | D |
| --- | --- | --- | --- | --- | --- |
| Phase 1: Assessing Relevance | | P | P | - | P |
| Phase 2: Identifying Concerns with Review Process | Study Eligibility Criteria | L | L | - | L |
|  | Identification and Selection of Studies | L | L | - | L |
|  | Data Collection and Study Appraisal | L | UN | L | L |
|  | Synthesis and Findings | L | L | - | L |
| Phase 3: Judging Risk of Bias | | L | UN | L | L |

ROBIS

P: Pass. F: Fail. L: Low risk of bias. H: High risk of bias. Un: Unclear risk of bias.

A:The conclusions of researcher Ph.D. Yongxiu Liu.

B:The conclusions of researcher Ph.D. Yuguo Li.

C:In case of a difference of opinions, it shall be adjudicated by Professor Lei Gao.

D:Conclusive conclusion.

Study 39：El Abd, Asmae et al. “The effects of vitamin D supplementation on inflammatory biomarkers in patients with asthma: a systematic review and meta-analysis of randomized controlled trials.”Frontiers in immunology vol. 15 1335968. 13 Mar. 2024, doi:10.3389/fimmu.2024.1335968.PMID: 38545098 PMCID: PMC10965564

| Phase | | A | B | C | D |
| --- | --- | --- | --- | --- | --- |
| Phase 1: Assessing Relevance | | P | P | - | P |
| Phase 2: Identifying Concerns with Review Process | Study Eligibility Criteria | L | L | - | L |
|  | Identification and Selection of Studies | L | L | - | L |
|  | Data Collection and Study Appraisal | L | L | - | L |
|  | Synthesis and Findings | L | UN | L | L |
| Phase 3: Judging Risk of Bias | | L | L | - | L |

ROBIS

P: Pass. F: Fail. L: Low risk of bias. H: High risk of bias. Un: Unclear risk of bias.

A:The conclusions of researcher Ph.D.Yongxiu Liu.

B:The conclusions of researcher Ph.D. Yuguo Li.

C:In case of a difference of opinions, it shall be adjudicated by Professor Lei Gao.

D:Conclusive conclusion.

Study 40：Fedora, Katherine et al. “Vitamin D supplementation decrease asthma exacerbations in children: a systematic review and meta-analysis of randomized controlled trials.” Annals of medicine vol. 56,1 (2024): 2400313. doi:10.1080/07853890.2024.2400313.PMID:39421966.PMCID: PMC11492411

| Phase | | A | B | C | D |
| --- | --- | --- | --- | --- | --- |
| Phase 1: Assessing Relevance | | P | P | - | P |
| Phase 2: Identifying Concerns with Review Process | Study Eligibility Criteria | L | L | - | L |
|  | Identification and Selection of Studies | UN | UN | - | UN |
|  | Data Collection and Study Appraisal | L | L | - | L |
|  | Synthesis and Findings | L | UN | L | L |
| Phase 3: Judging Risk of Bias | | L | UN | L | L |

ROBIS

P: Pass. F: Fail. L: Low risk of bias. H: High risk of bias. Un: Unclear risk of bias.

A:The conclusions of researcher Ph.D.Yongxiu Liu.

B:The conclusions of researcher Ph.D. Yuguo Li.

C:In case of a difference of opinions, it shall be adjudicated by Professor Lei Gao.

D:Conclusive conclusion.

Study 41：Niu, Haiying et al. “Asthmatic patients with vitamin D deficiency: Can vitamin D supplementation make a difference.” Technology and health care : official journal of the European Society for Engineering and Medicine vol. 32,6 (2024): 3985-4008. doi:10.3233/THC-231462.PMID: 39031398.PMCID: PMC11612934

| Phase | | A | B | C | D |
| --- | --- | --- | --- | --- | --- |
| Phase 1: Assessing Relevance | | P | P | - | P |
| Phase 2: Identifying Concerns with Review Process | Study Eligibility Criteria | L | L | - | L |
|  | Identification and Selection of Studies | L | L | - | L |
|  | Data Collection and Study Appraisal | L | L | - | L |
|  | Synthesis and Findings | L | L | - | L |
| Phase 3: Judging Risk of Bias | | L | L | - | L |

ROBIS

P: Pass. F: Fail. L: Low risk of bias. H: High risk of bias. Un: Unclear risk of bias.

A:The conclusions of researcher Ph.D.Yongxiu Liu.

B:The conclusions of researcher Ph.D. Yuguo Li.

C:In case of a difference of opinions, it shall be adjudicated by Professor Lei Gao.

D:Conclusive conclusion.

Study 42：杨玉丰,张慧中. 维生素D联合丙酸氟替卡松治疗儿童哮喘有效性的Meta分析[J]. 今日健康,2025(12):105-108. DOI:10.3969/j.issn.1671-5160.2025.12.044.

| Phase | | A | B | C | D |
| --- | --- | --- | --- | --- | --- |
| Phase 1: Assessing Relevance | | P | P | - | P |
| Phase 2: Identifying Concerns with Review Process | Study Eligibility Criteria | L | L | - | L |
|  | Identification and Selection of Studies | UN | UN | - | UN |
|  | Data Collection and Study Appraisal | L | H | UN | UN |
|  | Synthesis and Findings | L | H | L | L |
| Phase 3: Judging Risk of Bias | | L | H | H | H |

3.AMSTAR-2

Y: Yes; N: No; PY: Partial Yes.

A:The conclusions of researcher Ph.D.Yongxiu Liu.

B:The conclusions of researcher Ph.D. Yuguo Li..

C:In case of a difference of opinions, it shall be adjudicated by Professor Lei Gao.

D:Conclusive conclusion.

Study 22：Fares, Munes M et al. “Vitamin D supplementation in children with asthma: a systematic review and meta-analysis.” BMC research notes vol. 8 23. 3 Feb. 2015, doi:10.1186/s13104-014-0961-3.PMID: 25643669 PMCID: PMC4328422

| Entry | | A | B | C | D |
| --- | --- | --- | --- | --- | --- |
| 1 | Did the research questions and inclusion criteria for the review include the components of PICO? | Y | Y | - | Y |
| 2 | Did the report of the review contain an explicit statement that the review methods were established prior to the conduct of the review and did the report justify any significantdeviations from the protocol? | Y | Y | - | Y |
| 3 | Did the review authors explain their selection of the study designs for inclusion in the review? | Y | Y | - | Y |
| 4 | Did the review authors use a comprehensive literature search strategy? | Y | Y | - | Y |
| 5 | Did the review authors perform study selection in duplicate? | Y | Y | - | Y |
| 6 | Did the review authors perform data extraction in duplicate? | Y | Y | - | Y |
| 7 | Did the review authors provide a list of excluded studies and justify the exclusions? | PY | PY | - | PY |
| 8 | Did the review authors describe the included studies in adequate detail? | Y | Y | - | Y |
| 9 | Did the review authors use a satisfactory technique for assessing the risk of bias (RoB) in individual studies that were included in the review? | Y | PY | Y | Y |
| 10 | Did the review authors report on the sources of funding for the studies included in the review? | PY | N | Y | Y |
| 11 | If meta-analysis was performed, did the review authors use appropriate methods for statistical combination of results? | Y | PY | Y | Y |
| 12 | If meta-analysis was performed, did the review authors assess the potential impact of RoB in individual studies on the results of the meta-analysis or other evidence synthesis? | N | N | - | N |
| 13 | Did the review authors account for RoB in primary studies when interpreting/discussing the results of the review? | Y | Y | - | Y |
| 14 | Did the review authors provide a satisfactory explanation for, and discussion of, any heterogeneity observed in the results of the review? | PY | PY | - | PY |
| 15 | If they performed quantitative synthesis did the review authors carry out an adequate investigation of publication bias (small study bias) and discuss its likely impact on the results of the review? | N | N | - | N |
| 16 | Did the review authors report any potential sources of conflict of interest, including any funding they received for conducting the review? | Y | Y | - | Y |

AMSTAR-2

Y: Yes; N: No; PY: Partial Yes.

A:The conclusions of researcher Ph.D.Yongxiu Liu.

B:The conclusions of researcher Ph.D. Yuguo Li..

C:In case of a difference of opinions, it shall be adjudicated by Professor Lei Gao.

D:Conclusive conclusion.

Study 23：Luo, Jian et al. “Can Vitamin D Supplementation in Addition to Asthma Controllers Improve Clinical Outcomes in Patients With Asthma?: A Meta-Analysis.” Medicine vol. 94,50 (2015): e2185. doi:10.1097/MD.0000000000002185.PMID: 26683927 PMCID: PMC5058899

| Entry | | A | B | C | D |
| --- | --- | --- | --- | --- | --- |
| 1 | Did the research questions and inclusion criteria for the review include the components of PICO? | Y | Y | - | Y |
| 2 | Did the report of the review contain an explicit statement that the review methods were established prior to the conduct of the review and did the report justify any significantdeviations from the protocol? | PY | N | N | N |
| 3 | Did the review authors explain their selection of the study designs for inclusion in the review? | Y | Y | - | Y |
| 4 | Did the review authors use a comprehensive literature search strategy? | Y | PY | PY | PY |
| 5 | Did the review authors perform study selection in duplicate? | Y | Y | - | Y |
| 6 | Did the review authors perform data extraction in duplicate? | Y | Y | - | Y |
| 7 | Did the review authors provide a list of excluded studies and justify the exclusions? | PY | PY | - | PY |
| 8 | Did the review authors describe the included studies in adequate detail? | Y | Y | - | Y |
| 9 | Did the review authors use a satisfactory technique for assessing the risk of bias (RoB) in individual studies that were included in the review? | Y | PY | Y | Y |
| 10 | Did the review authors report on the sources of funding for the studies included in the review? | N | N | - | N |
| 11 | If meta-analysis was performed, did the review authors use appropriate methods for statistical combination of results? | Y | Y | - | Y |
| 12 | If meta-analysis was performed, did the review authors assess the potential impact of RoB in individual studies on the results of the meta-analysis or other evidence synthesis? | N | N | - | N |
| 13 | Did the review authors account for RoB in primary studies when interpreting/discussing the results of the review? | PY | Y | N | N |
| 14 | Did the review authors provide a satisfactory explanation for, and discussion of, any heterogeneity observed in the results of the review? | PY | PY | - | PY |
| 15 | If they performed quantitative synthesis did the review authors carry out an adequate investigation of publication bias (small study bias) and discuss its likely impact on the results of the review? | N | N | - | N |
| 16 | Did the review authors report any potential sources of conflict of interest, including any funding they received for conducting the review? | Y | Y | - | Y |

AMSTAR-2

Y: Yes; N: No; PY: Partial Yes.

A:The conclusions of researcher Ph.D.Yongxiu Liu.

B:The conclusions of researcher Ph.D. Yuguo Li.

C:In case of a difference of opinions, it shall be adjudicated by Professor Lei Gao.

D:Conclusive conclusion.

Study 24：Pojsupap, Supichaya et al. “Efficacy of high-dose vitamin D in pediatric asthma: a systematic review and meta-analysis.” The Journal of asthma : official journal of the Association for the Care of Asthma vol. 52,4 (2015): 382-90. doi:10.3109/02770903.2014.980509.PMID: 25365192

| Entry | | A | B | C | D |
| --- | --- | --- | --- | --- | --- |
| 1 | Did the research questions and inclusion criteria for the review include the components of PICO? | Y | Y | - | Y |
| 2 | Did the report of the review contain an explicit statement that the review methods were established prior to the conduct of the review and did the report justify any significantdeviations from the protocol? | Y | N | Y | Y |
| 3 | Did the review authors explain their selection of the study designs for inclusion in the review? | Y | Y | - | Y |
| 4 | Did the review authors use a comprehensive literature search strategy? | Y | Y | - | Y |
| 5 | Did the review authors perform study selection in duplicate? | Y | Y | - | Y |
| 6 | Did the review authors perform data extraction in duplicate? | Y | Y | - | Y |
| 7 | Did the review authors provide a list of excluded studies and justify the exclusions? | PY | PY | - | PY |
| 8 | Did the review authors describe the included studies in adequate detail? | Y | Y | - | Y |
| 9 | Did the review authors use a satisfactory technique for assessing the risk of bias (RoB) in individual studies that were included in the review? | Y | Y | - | Y |
| 10 | Did the review authors report on the sources of funding for the studies included in the review? | N | N | - | N |
| 11 | If meta-analysis was performed, did the review authors use appropriate methods for statistical combination of results? | Y | Y | - | Y |
| 12 | If meta-analysis was performed, did the review authors assess the potential impact of RoB in individual studies on the results of the meta-analysis or other evidence synthesis? | N | PY | Y | Y |
| 13 | Did the review authors account for RoB in primary studies when interpreting/discussing the results of the review? | PY | Y | Y | Y |
| 14 | Did the review authors provide a satisfactory explanation for, and discussion of, any heterogeneity observed in the results of the review? | Y | Y | - | Y |
| 15 | If they performed quantitative synthesis did the review authors carry out an adequate investigation of publication bias (small study bias) and discuss its likely impact on the results of the review? | N | N | - | N |
| 16 | Did the review authors report any potential sources of conflict of interest, including any funding they received for conducting the review? | Y | Y | - | Y |

AMSTAR-2

Y: Yes; N: No; PY: Partial Yes.

A:The conclusions of researcher Ph.D.Yongxiu Liu.

B:The conclusions of researcher Ph.D. Yuguo Li.

C:In case of a difference of opinions, it shall be adjudicated by Professor Lei Gao.

D:Conclusive conclusion.

Study 25：Riverin, Bruno D et al. “Vitamin D Supplementation for Childhood Asthma: A Systematic Review and Meta-Analysis.” PloS one vol. 10,8 e0136841. 31 Aug. 2015, doi:10.1371/journal.pone.0136841.PMID: 26322509 PMCID: PMC4556456

| Entry | | A | B | C | D |
| --- | --- | --- | --- | --- | --- |
| 1 | Did the research questions and inclusion criteria for the review include the components of PICO? | Y | Y | - | Y |
| 2 | Did the report of the review contain an explicit statement that the review methods were established prior to the conduct of the review and did the report justify any significantdeviations from the protocol? | PY | N | N | N |
| 3 | Did the review authors explain their selection of the study designs for inclusion in the review? | Y | Y | - | Y |
| 4 | Did the review authors use a comprehensive literature search strategy? | Y | Y | - | Y |
| 5 | Did the review authors perform study selection in duplicate? | Y | Y | - | Y |
| 6 | Did the review authors perform data extraction in duplicate? | Y | Y | - | Y |
| 7 | Did the review authors provide a list of excluded studies and justify the exclusions? | PY | PY | - | PY |
| 8 | Did the review authors describe the included studies in adequate detail? | Y | Y | - | Y |
| 9 | Did the review authors use a satisfactory technique for assessing the risk of bias (RoB) in individual studies that were included in the review? | Y | Y | - | Y |
| 10 | Did the review authors report on the sources of funding for the studies included in the review? | N | N | - | N |
| 11 | If meta-analysis was performed, did the review authors use appropriate methods for statistical combination of results? | Y | Y | - | Y |
| 12 | If meta-analysis was performed, did the review authors assess the potential impact of RoB in individual studies on the results of the meta-analysis or other evidence synthesis? | N | PY | N | N |
| 13 | Did the review authors account for RoB in primary studies when interpreting/discussing the results of the review? | Y | Y | - | Y |
| 14 | Did the review authors provide a satisfactory explanation for, and discussion of, any heterogeneity observed in the results of the review? | Y | Y | - | Y |
| 15 | If they performed quantitative synthesis did the review authors carry out an adequate investigation of publication bias (small study bias) and discuss its likely impact on the results of the review? | PY | PY | - | PY |
| 16 | Did the review authors report any potential sources of conflict of interest, including any funding they received for conducting the review? | Y | Y | - | Y |

AMSTAR-2

Y: Yes; N: No; PY: Partial Yes.

A:The conclusions of researcher Ph.D.Yongxiu Liu.

B:The conclusions of researcher Ph.D. Yuguo Li.

C:In case of a difference of opinions, it shall be adjudicated by Professor Lei Gao.

D:Conclusive conclusion.

AMSTAR-2

Y: Yes; N: No; PY: Partial Yes.

A:The conclusions of researcher Ph.D.Yongxiu Liu.

B:The conclusions of researcher Ph.D. Yuguo Li..

C:In case of a difference of opinions, it shall be adjudicated by ProfessorLei Gao.

D:Conclusive conclusion.

Study 26：景伟超,刘璐佳,关洋洋,等.维生素D辅助治疗儿童哮喘Meta分析[J].世界中西医结合杂志,2017,12(10):1341-1344+1354.DOI:10.13935/j.cnki.sjzx.171003.

| Entry | | A | B | C | D |
| --- | --- | --- | --- | --- | --- |
| 1 | Did the research questions and inclusion criteria for the review include the components of PICO? | Y | Y | - | Y |
| 2 | Did the report of the review contain an explicit statement that the review methods were established prior to the conduct of the review and did the report justify any significantdeviations from the protocol? | PY | N | N | N |
| 3 | Did the review authors explain their selection of the study designs for inclusion in the review? | Y | Y | - | Y |
| 4 | Did the review authors use a comprehensive literature search strategy? | PY | PY | - | PY |
| 5 | Did the review authors perform study selection in duplicate? | Y | Y | - | Y |
| 6 | Did the review authors perform data extraction in duplicate? | Y | Y | - | Y |
| 7 | Did the review authors provide a list of excluded studies and justify the exclusions? | PY | N | Y | Y |
| 8 | Did the review authors describe the included studies in adequate detail? | PY | Y | Y | Y |
| 9 | Did the review authors use a satisfactory technique for assessing the risk of bias (RoB) in individual studies that were included in the review? | PY | N | PY | PY |
| 10 | Did the review authors report on the sources of funding for the studies included in the review? | N | N | - | N |
| 11 | If meta-analysis was performed, did the review authors use appropriate methods for statistical combination of results? | Y | PY | Y | Y |
| 12 | If meta-analysis was performed, did the review authors assess the potential impact of RoB in individual studies on the results of the meta-analysis or other evidence synthesis? | N | N | - | N |
| 13 | Did the review authors account for RoB in primary studies when interpreting/discussing the results of the review? | N | PY | Y | Y |
| 14 | Did the review authors provide a satisfactory explanation for, and discussion of, any heterogeneity observed in the results of the review? | N | Y | PY | PY |
| 15 | If they performed quantitative synthesis did the review authors carry out an adequate investigation of publication bias (small study bias) and discuss its likely impact on the results of the review? | PY | Y | Y | Y |
| 16 | Did the review authors report any potential sources of conflict of interest, including any funding they received for conducting the review? | Y | Y | - | Y |

AMSTAR-2

Y: Yes; N: No; PY: Partial Yes.

A:The conclusions of researcher Ph.D.Yongxiu Liu.

B:The conclusions of researcher Ph.D. Yuguo Li.

C:In case of a difference of opinions, it shall be adjudicated by Professor Lei Gao.

D:Conclusive conclusion.

Study 27：Jolliffe, David A et al. “Vitamin D supplementation to prevent asthma exacerbations: a systematic review and meta-analysis of individual participant data.” The Lancet. Respiratory medicine vol. 5,11 (2017): 881-890. doi:10.1016/S2213-2600(17)30306-5.PMID: 28986128 PMCID: PMC5693329

| Entry | | A | B | C | D |
| --- | --- | --- | --- | --- | --- |
| 1 | Did the research questions and inclusion criteria for the review include the components of PICO? | Y | Y | - | Y |
| 2 | Did the report of the review contain an explicit statement that the review methods were established prior to the conduct of the review and did the report justify any significantdeviations from the protocol? | Y | Y | - | Y |
| 3 | Did the review authors explain their selection of the study designs for inclusion in the review? | Y | Y | - | Y |
| 4 | Did the review authors use a comprehensive literature search strategy? | Y | Y | - | Y |
| 5 | Did the review authors perform study selection in duplicate? | Y | Y | - | Y |
| 6 | Did the review authors perform data extraction in duplicate? | Y | Y | - | Y |
| 7 | Did the review authors provide a list of excluded studies and justify the exclusions? | PY | N | PY | PY |
| 8 | Did the review authors describe the included studies in adequate detail? | Y | Y | - | Y |
| 9 | Did the review authors use a satisfactory technique for assessing the risk of bias (RoB) in individual studies that were included in the review? | Y | Y | - | Y |
| 10 | Did the review authors report on the sources of funding for the studies included in the review? | N | N | - | N |
| 11 | If meta-analysis was performed, did the review authors use appropriate methods for statistical combination of results? | Y | Y | - | Y |
| 12 | If meta-analysis was performed, did the review authors assess the potential impact of RoB in individual studies on the results of the meta-analysis or other evidence synthesis? | PY | PY | - | PY |
| 13 | Did the review authors account for RoB in primary studies when interpreting/discussing the results of the review? | Y | Y | - | Y |
| 14 | Did the review authors provide a satisfactory explanation for, and discussion of, any heterogeneity observed in the results of the review? | Y | Y | - | Y |
| 15 | If they performed quantitative synthesis did the review authors carry out an adequate investigation of publication bias (small study bias) and discuss its likely impact on the results of the review? | PY | Y | Y | Y |
| 16 | Did the review authors report any potential sources of conflict of interest, including any funding they received for conducting the review? | Y | Y | - | Y |

AMSTAR-2

Y: Yes; N: No; PY: Partial Yes.

A:The conclusions of researcher Ph.D.Yongxiu Liu.

B:The conclusions of researcher Ph.D. Yuguo Li..

C:In case of a difference of opinions, it shall be adjudicated by Professor Lei Gao.

D:Conclusive conclusion.

Study 28：郝宏霞.维生素D在缓解期哮喘患者治疗中的有效性和安全性meta分析[D].山西医科大学,2018.

| Entry | | A | B | C | D |
| --- | --- | --- | --- | --- | --- |
| 1 | Did the research questions and inclusion criteria for the review include the components of PICO? | Y | Y | - | Y |
| 2 | Did the report of the review contain an explicit statement that the review methods were established prior to the conduct of the review and did the report justify any significantdeviations from the protocol? | PY | N | N- | N |
| 3 | Did the review authors explain their selection of the study designs for inclusion in the review? | Y | Y | - | Y |
| 4 | Did the review authors use a comprehensive literature search strategy? | Y | PY | Y | Y |
| 5 | Did the review authors perform study selection in duplicate? | Y | Y | - | Y |
| 6 | Did the review authors perform data extraction in duplicate? | Y | Y | - | Y |
| 7 | Did the review authors provide a list of excluded studies and justify the exclusions? | PY | PY | - | PY |
| 8 | Did the review authors describe the included studies in adequate detail? | Y | Y | - | Y |
| 9 | Did the review authors use a satisfactory technique for assessing the risk of bias (RoB) in individual studies that were included in the review? | Y | Y | - | Y |
| 10 | Did the review authors report on the sources of funding for the studies included in the review? | N | N | - | N |
| 11 | If meta-analysis was performed, did the review authors use appropriate methods for statistical combination of results? | Y | Y | - | Y |
| 12 | If meta-analysis was performed, did the review authors assess the potential impact of RoB in individual studies on the results of the meta-analysis or other evidence synthesis? | N | PY | PY | PY |
| 13 | Did the review authors account for RoB in primary studies when interpreting/discussing the results of the review? | N | Y | PY | PY |
| 14 | Did the review authors provide a satisfactory explanation for, and discussion of, any heterogeneity observed in the results of the review? | PY | Y | Y | Y |
| 15 | If they performed quantitative synthesis did the review authors carry out an adequate investigation of publication bias (small study bias) and discuss its likely impact on the results of the review? | PY | Y | Y | Y |
| 16 | Did the review authors report any potential sources of conflict of interest, including any funding they received for conducting the review? | Y | Y | - | Y |

AMSTAR-2

Y: Yes; N: No; PY: Partial Yes.

A:The conclusions of researcher Ph.D.Yongxiu Liu.

B:The conclusions of researcher Ph.D. Yuguo Li..

C:In case of a difference of opinions, it shall be adjudicated by Professor Lei Gao.

D:Conclusive conclusion.

Study 29：田超,史强,赵紫楠,等.维生素D补充剂对儿童支气管哮喘获益相关性的系统评价和meta分析[J].临床药物治疗杂志,2018,16(04):66-70.

| Entry | | A | B | C | D |
| --- | --- | --- | --- | --- | --- |
| 1 | Did the research questions and inclusion criteria for the review include the components of PICO? | Y | Y | - | Y |
| 2 | Did the report of the review contain an explicit statement that the review methods were established prior to the conduct of the review and did the report justify any significantdeviations from the protocol? | PY | N | N | N |
| 3 | Did the review authors explain their selection of the study designs for inclusion in the review? | Y | Y | - | Y |
| 4 | Did the review authors use a comprehensive literature search strategy? | Y | PY | N | N |
| 5 | Did the review authors perform study selection in duplicate? | Y | Y | - | Y |
| 6 | Did the review authors perform data extraction in duplicate? | Y | Y | - | Y |
| 7 | Did the review authors provide a list of excluded studies and justify the exclusions? | PY | PY | - | PY |
| 8 | Did the review authors describe the included studies in adequate detail? | Y | Y | - | Y |
| 9 | Did the review authors use a satisfactory technique for assessing the risk of bias (RoB) in individual studies that were included in the review? | Y | Y | - | Y |
| 10 | Did the review authors report on the sources of funding for the studies included in the review? | N | N | - | N |
| 11 | If meta-analysis was performed, did the review authors use appropriate methods for statistical combination of results? | Y | PY | Y | Y |
| 12 | If meta-analysis was performed, did the review authors assess the potential impact of RoB in individual studies on the results of the meta-analysis or other evidence synthesis? | N | N | - | N |
| 13 | Did the review authors account for RoB in primary studies when interpreting/discussing the results of the review? | PY | Y | PY | PY |
| 14 | Did the review authors provide a satisfactory explanation for, and discussion of, any heterogeneity observed in the results of the review? | N | PY | PY | PY |
| 15 | If they performed quantitative synthesis did the review authors carry out an adequate investigation of publication bias (small study bias) and discuss its likely impact on the results of the review? | N | N | - | N |
| 16 | Did the review authors report any potential sources of conflict of interest, including any funding they received for conducting the review? | Y | Y | - | Y |

AMSTAR-2

Y: Yes; N: No; PY: Partial Yes.

A:The conclusions of researcher Ph.D.Yongxiu Liu.

B:The conclusions of researcher Ph.D. Yuguo Li..

C:In case of a difference of opinions, it shall be adjudicated by Professor Lei Gao.

D:Conclusive conclusion.

Study 30：郝畅.维生素D补充与儿童哮喘的系统评价及meta分析[D].重庆医科大学,2019.

| Entry | | A | B | C | D |
| --- | --- | --- | --- | --- | --- |
| 1 | Did the research questions and inclusion criteria for the review include the components of PICO? | Y | PY | Y | Y |
| 2 | Did the report of the review contain an explicit statement that the review methods were established prior to the conduct of the review and did the report justify any significantdeviations from the protocol? | PY | N | N | N |
| 3 | Did the review authors explain their selection of the study designs for inclusion in the review? | Y | Y | - | Y |
| 4 | Did the review authors use a comprehensive literature search strategy? | Y | PY | PY | PY |
| 5 | Did the review authors perform study selection in duplicate? | Y | Y | - | Y |
| 6 | Did the review authors perform data extraction in duplicate? | Y | Y | - | Y |
| 7 | Did the review authors provide a list of excluded studies and justify the exclusions? | PY | N | PY | PY |
| 8 | Did the review authors describe the included studies in adequate detail? | Y | Y | - | Y |
| 9 | Did the review authors use a satisfactory technique for assessing the risk of bias (RoB) in individual studies that were included in the review? | Y | Y | - | Y |
| 10 | Did the review authors report on the sources of funding for the studies included in the review? | PY | N | N | N |
| 11 | If meta-analysis was performed, did the review authors use appropriate methods for statistical combination of results? | Y | PY | Y | Y |
| 12 | If meta-analysis was performed, did the review authors assess the potential impact of RoB in individual studies on the results of the meta-analysis or other evidence synthesis? | N | N | - | N |
| 13 | Did the review authors account for RoB in primary studies when interpreting/discussing the results of the review? | PY | PY | - | PY |
| 14 | Did the review authors provide a satisfactory explanation for, and discussion of, any heterogeneity observed in the results of the review? | N | PY | Y | Y |
| 15 | If they performed quantitative synthesis did the review authors carry out an adequate investigation of publication bias (small study bias) and discuss its likely impact on the results of the review? | N | PY | Y | Y |
| 16 | Did the review authors report any potential sources of conflict of interest, including any funding they received for conducting the review? | Y | N | N | N |

AMSTAR-2

Y: Yes; N: No; PY: Partial Yes.

A:The conclusions of researcher Ph.D.Yongxiu Liu.

B:The conclusions of researcher Ph.D. Yuguo Li..

C:In case of a difference of opinions, it shall be adjudicated by Professor Lei Gao.

D:Conclusive conclusion.

Study 31：Wang, Mingming et al. “Association between vitamin D status and asthma control: A meta-analysis of randomized trials.”Respiratory medicine vol. 150 (2019): 85-94. doi:10.1016/j.rmed.2019.02.016.PMID: 30961957

| Entry | | A | B | C | D |
| --- | --- | --- | --- | --- | --- |
| 1 | Did the research questions and inclusion criteria for the review include the components of PICO? | Y | Y | - | Y |
| 2 | Did the report of the review contain an explicit statement that the review methods were established prior to the conduct of the review and did the report justify any significantdeviations from the protocol? | Y | Y | - | Y |
| 3 | Did the review authors explain their selection of the study designs for inclusion in the review? | Y | Y | - | Y |
| 4 | Did the review authors use a comprehensive literature search strategy? | Y | PY | Y | Y |
| 5 | Did the review authors perform study selection in duplicate? | Y | Y | - | Y |
| 6 | Did the review authors perform data extraction in duplicate? | Y | Y | - | Y |
| 7 | Did the review authors provide a list of excluded studies and justify the exclusions? | PY | N | Y | Y |
| 8 | Did the review authors describe the included studies in adequate detail? | Y | Y | - | Y |
| 9 | Did the review authors use a satisfactory technique for assessing the risk of bias (RoB) in individual studies that were included in the review? | Y | PY | Y | Y |
| 10 | Did the review authors report on the sources of funding for the studies included in the review? | N | N | - | N |
| 11 | If meta-analysis was performed, did the review authors use appropriate methods for statistical combination of results? | Y | Y | - | Y |
| 12 | If meta-analysis was performed, did the review authors assess the potential impact of RoB in individual studies on the results of the meta-analysis or other evidence synthesis? | PY | PY | - | PY |
| 13 | Did the review authors account for RoB in primary studies when interpreting/discussing the results of the review? | Y | PY | Y | Y |
| 14 | Did the review authors provide a satisfactory explanation for, and discussion of, any heterogeneity observed in the results of the review? | PY | Y | Y | Y |
| 15 | If they performed quantitative synthesis did the review authors carry out an adequate investigation of publication bias (small study bias) and discuss its likely impact on the results of the review? | N | PY | PY | PY |
| 16 | Did the review authors report any potential sources of conflict of interest, including any funding they received for conducting the review? | Y | Y | - | Y |

AMSTAR-2

Y: Yes; N: No; PY: Partial Yes.

A:The conclusions of researcher Ph.D.Yongxiu Liu.

B:The conclusions of researcher Ph.D. Yuguo Li..

C:In case of a difference of opinions, it shall be adjudicated by Professor Lei Gao.

D:Conclusive conclusion.

Study 32：Chen, Ziyu et al. “Vitamin D can safely reduce asthma exacerbations among corticosteroid-using children and adults with asthma: a systematic review and meta-analysis of randomized controlled trials.” Nutrition research (New York, N.Y.) vol. 92 (2021): 49-61. doi:10.1016/j.nutres.2021.05.010.PMID: 34274554

| Entry | | A | B | C | D |
| --- | --- | --- | --- | --- | --- |
| 1 | Did the research questions and inclusion criteria for the review include the components of PICO? | Y | Y | - | Y |
| 2 | Did the report of the review contain an explicit statement that the review methods were established prior to the conduct of the review and did the report justify any significantdeviations from the protocol? | PY | PY | - | PY |
| 3 | Did the review authors explain their selection of the study designs for inclusion in the review? | Y | Y | - | Y |
| 4 | Did the review authors use a comprehensive literature search strategy? | Y | PY | Y | Y |
| 5 | Did the review authors perform study selection in duplicate? | Y | Y | - | Y |
| 6 | Did the review authors perform data extraction in duplicate? | Y | Y | - | Y |
| 7 | Did the review authors provide a list of excluded studies and justify the exclusions? | PY | N | Y | Y |
| 8 | Did the review authors describe the included studies in adequate detail? | Y | Y | - | Y |
| 9 | Did the review authors use a satisfactory technique for assessing the risk of bias (RoB) in individual studies that were included in the review? | Y | Y | - | Y |
| 10 | Did the review authors report on the sources of funding for the studies included in the review? | N | N | - | N |
| 11 | If meta-analysis was performed, did the review authors use appropriate methods for statistical combination of results? | Y | Y | - | Y |
| 12 | If meta-analysis was performed, did the review authors assess the potential impact of RoB in individual studies on the results of the meta-analysis or other evidence synthesis? | N | PY | N | N |
| 13 | Did the review authors account for RoB in primary studies when interpreting/discussing the results of the review? | PY | PY | - | PY |
| 14 | Did the review authors provide a satisfactory explanation for, and discussion of, any heterogeneity observed in the results of the review? | Y | Y | - | Y |
| 15 | If they performed quantitative synthesis did the review authors carry out an adequate investigation of publication bias (small study bias) and discuss its likely impact on the results of the review? | Y | Y | - | Y |
| 16 | Did the review authors report any potential sources of conflict of interest, including any funding they received for conducting the review? | Y | Y | - | Y |

AMSTAR-2

Y: Yes; N: No; PY: Partial Yes.

A:The conclusions of researcher Ph.D.Yongxiu Liu.

B:The conclusions of researcher Ph.D. Yuguo Li..

C:In case of a difference of opinions, it shall be adjudicated by Professor Lei Gao.

D:Conclusive conclusion.

Study 33：Hao, Meiqi et al. “The Effect of Vitamin D Supplementation in Children With Asthma: A Meta-Analysis.”Frontiers in pediatrics vol. 10 840617. 29 Jun. 2022, doi:10.3389/fped.2022.840617.PMID: 35844729 PMCID: PMC9277022

| Entry | | A | B | C | D |
| --- | --- | --- | --- | --- | --- |
| 1 | Did the research questions and inclusion criteria for the review include the components of PICO? | Y | Y | - | Y |
| 2 | Did the report of the review contain an explicit statement that the review methods were established prior to the conduct of the review and did the report justify any significantdeviations from the protocol? | Y | Y | - | Y |
| 3 | Did the review authors explain their selection of the study designs for inclusion in the review? | Y | Y | - | Y |
| 4 | Did the review authors use a comprehensive literature search strategy? | Y | PY | Y | Y |
| 5 | Did the review authors perform study selection in duplicate? | Y | Y | - | Y |
| 6 | Did the review authors perform data extraction in duplicate? | Y | Y | - | Y |
| 7 | Did the review authors provide a list of excluded studies and justify the exclusions? | PY | N | Y | Y |
| 8 | Did the review authors describe the included studies in adequate detail? | Y | Y | - | Y |
| 9 | Did the review authors use a satisfactory technique for assessing the risk of bias (RoB) in individual studies that were included in the review? | Y | PY | PY | PY |
| 10 | Did the review authors report on the sources of funding for the studies included in the review? | N | N | - | N |
| 11 | If meta-analysis was performed, did the review authors use appropriate methods for statistical combination of results? | Y | Y | - | Y |
| 12 | If meta-analysis was performed, did the review authors assess the potential impact of RoB in individual studies on the results of the meta-analysis or other evidence synthesis? | N | PY | N | N |
| 13 | Did the review authors account for RoB in primary studies when interpreting/discussing the results of the review? | PY | PY | - | PY |
| 14 | Did the review authors provide a satisfactory explanation for, and discussion of, any heterogeneity observed in the results of the review? | Y | Y | - | Y |
| 15 | If they performed quantitative synthesis did the review authors carry out an adequate investigation of publication bias (small study bias) and discuss its likely impact on the results of the review? | Y | Y | - | Y |
| 16 | Did the review authors report any potential sources of conflict of interest, including any funding they received for conducting the review? | Y | Y | - | Y |

AMSTAR-2

Y: Yes; N: No; PY: Partial Yes.

A:The conclusions of researcher Ph.D.Yongxiu Liu.

B:The conclusions of researcher Ph.D. Yuguo Li..

C:In case of a difference of opinions, it shall be adjudicated by Professor Lei Gao.

D:Conclusive conclusion.

Study 34：Kumar, Jogender et al. “Vitamin D supplementation in childhood asthma: a systematic review and meta-analysis of randomised controlled trials.” ERJ open research vol. 8,1 00662-2021. 7 Feb. 2021, doi:10.1183/23120541.00662-2021.PMID: 35141325 PMCID: PMC8819253

| Entry | | A | B | C | D |
| --- | --- | --- | --- | --- | --- |
| 1 | Did the research questions and inclusion criteria for the review include the components of PICO? | Y | Y | - | Y |
| 2 | Did the report of the review contain an explicit statement that the review methods were established prior to the conduct of the review and did the report justify any significantdeviations from the protocol? | Y | Y | - | Y |
| 3 | Did the review authors explain their selection of the study designs for inclusion in the review? | Y | Y | - | Y |
| 4 | Did the review authors use a comprehensive literature search strategy? | Y | Y | - | Y |
| 5 | Did the review authors perform study selection in duplicate? | Y | Y | - | Y |
| 6 | Did the review authors perform data extraction in duplicate? | Y | Y | - | Y |
| 7 | Did the review authors provide a list of excluded studies and justify the exclusions? | PY | Y | Y | Y |
| 8 | Did the review authors describe the included studies in adequate detail? | Y | Y | - | Y |
| 9 | Did the review authors use a satisfactory technique for assessing the risk of bias (RoB) in individual studies that were included in the review? | Y | Y | - | Y |
| 10 | Did the review authors report on the sources of funding for the studies included in the review? | N | PY | N | N |
| 11 | If meta-analysis was performed, did the review authors use appropriate methods for statistical combination of results? | Y | Y | - | Y |
| 12 | If meta-analysis was performed, did the review authors assess the potential impact of RoB in individual studies on the results of the meta-analysis or other evidence synthesis? | PY | N | Y | Y |
| 13 | Did the review authors account for RoB in primary studies when interpreting/discussing the results of the review? | Y | Y | - | Y |
| 14 | Did the review authors provide a satisfactory explanation for, and discussion of, any heterogeneity observed in the results of the review? | Y | Y | - | Y |
| 15 | If they performed quantitative synthesis did the review authors carry out an adequate investigation of publication bias (small study bias) and discuss its likely impact on the results of the review? | PY | Y | PY | PY |
| 16 | Did the review authors report any potential sources of conflict of interest, including any funding they received for conducting the review? | Y | Y | - | Y |

AMSTAR-2

Y: Yes; N: No; PY: Partial Yes.

A:The conclusions of researcher Ph.D.Yongxiu Liu.

B:The conclusions of researcher Ph.D. Yuguo Li..

C:In case of a difference of opinions, it shall be adjudicated by Professor Lei Gao.

D:Conclusive conclusion.

Study 35：

Liu, Meiqi et al. “A Meta-Analysis on Vitamin D Supplementation and Asthma Treatment.” Frontiers in nutrition vol. 9 860628. 6 Jul. 2022, doi:10.3389/fnut.2022.860628.PMID: 35873428 PMCID: PMC9300755

| Entry | | A | B | C | D |
| --- | --- | --- | --- | --- | --- |
| 1 | Did the research questions and inclusion criteria for the review include the components of PICO? | Y | Y | - | Y |
| 2 | Did the report of the review contain an explicit statement that the review methods were established prior to the conduct of the review and did the report justify any significantdeviations from the protocol? | Y | PY | Y | Y |
| 3 | Did the review authors explain their selection of the study designs for inclusion in the review? | Y | Y | - | Y |
| 4 | Did the review authors use a comprehensive literature search strategy? | PY | PY | - | PY |
| 5 | Did the review authors perform study selection in duplicate? | Y | Y | - | Y |
| 6 | Did the review authors perform data extraction in duplicate? | PY | Y | Y | Y |
| 7 | Did the review authors provide a list of excluded studies and justify the exclusions? | PY | N | Y | Y |
| 8 | Did the review authors describe the included studies in adequate detail? | Y | Y | - | Y |
| 9 | Did the review authors use a satisfactory technique for assessing the risk of bias (RoB) in individual studies that were included in the review? | Y | PY | Y | Y |
| 10 | Did the review authors report on the sources of funding for the studies included in the review? | N | N | - | N |
| 11 | If meta-analysis was performed, did the review authors use appropriate methods for statistical combination of results? | Y | Y | - | Y |
| 12 | If meta-analysis was performed, did the review authors assess the potential impact of RoB in individual studies on the results of the meta-analysis or other evidence synthesis? | N | PY | PY | PY |
| 13 | Did the review authors account for RoB in primary studies when interpreting/discussing the results of the review? | PY | PY | - | PY |
| 14 | Did the review authors provide a satisfactory explanation for, and discussion of, any heterogeneity observed in the results of the review? | Y | Y | - | Y |
| 15 | If they performed quantitative synthesis did the review authors carry out an adequate investigation of publication bias (small study bias) and discuss its likely impact on the results of the review? | N | PY | PY | PY |
| 16 | Did the review authors report any potential sources of conflict of interest, including any funding they received for conducting the review? | Y | Y | - | Y |

AMSTAR-2

Y: Yes; N: No; PY: Partial Yes.

A:The conclusions of researcher Ph.D.Yongxiu Liu.

B:The conclusions of researcher Ph.D. Yuguo Li..

C:In case of a difference of opinions, it shall be adjudicated by Professor Lei Gao.

D:Conclusive conclusion.

Study 36：Nitzan, Itamar et al. “Vitamin D and Asthma: a Systematic Review of Clinical Trials.” Current nutrition reports vol. 11,2 (2022): 311-317. doi:10.1007/s13668-022-00411-6.PMID: 35347665

| Entry | | A | B | C | D |
| --- | --- | --- | --- | --- | --- |
| 1 | Did the research questions and inclusion criteria for the review include the components of PICO? | Y | Y | - | Y |
| 2 | Did the report of the review contain an explicit statement that the review methods were established prior to the conduct of the review and did the report justify any significantdeviations from the protocol? | PY | PY | - | PY |
| 3 | Did the review authors explain their selection of the study designs for inclusion in the review? | Y | Y | - | Y |
| 4 | Did the review authors use a comprehensive literature search strategy? | Y | PY | PY | PY |
| 5 | Did the review authors perform study selection in duplicate? | PY | PY | - | PY |
| 6 | Did the review authors perform data extraction in duplicate? | Y | Y | - | Y |
| 7 | Did the review authors provide a list of excluded studies and justify the exclusions? | PY | N | Y | Y |
| 8 | Did the review authors describe the included studies in adequate detail? | Y | Y | - | Y |
| 9 | Did the review authors use a satisfactory technique for assessing the risk of bias (RoB) in individual studies that were included in the review? | N | PY | N | N |
| 10 | Did the review authors report on the sources of funding for the studies included in the review? | N | N | - | N |
| 11 | If meta-analysis was performed, did the review authors use appropriate methods for statistical combination of results? | PY | PY | - | PY |
| 12 | If meta-analysis was performed, did the review authors assess the potential impact of RoB in individual studies on the results of the meta-analysis or other evidence synthesis? | N | N | - | N |
| 13 | Did the review authors account for RoB in primary studies when interpreting/discussing the results of the review? | PY | PY | - | PY |
| 14 | Did the review authors provide a satisfactory explanation for, and discussion of, any heterogeneity observed in the results of the review? | Y | Y | - | Y |
| 15 | If they performed quantitative synthesis did the review authors carry out an adequate investigation of publication bias (small study bias) and discuss its likely impact on the results of the review? | N | PY | N | N |
| 16 | Did the review authors report any potential sources of conflict of interest, including any funding they received for conducting the review? | Y | Y | - | Y |

AMSTAR-2

Y: Yes; N: No; PY: Partial Yes.

A:The conclusions of researcher Ph.D.Yongxiu Liu.

B:The conclusions of researcher Ph.D. Yuguo Li..

C:In case of a difference of opinions, it shall be adjudicated by Professor Lei Gao.

D:Conclusive conclusion.

Study 37：Williamson, Anne et al. “Vitamin D for the management of asthma.” The Cochrane database of systematic reviews vol. 2,2 CD011511. 6 Feb. 2023, doi:10.1002/14651858.CD011511.pub3.PMID: 36744416 PMCID: PMC9899558

| Entry | | A | B | C | D |
| --- | --- | --- | --- | --- | --- |
| 1 | Did the research questions and inclusion criteria for the review include the components of PICO? | Y | Y | - | Y |
| 2 | Did the report of the review contain an explicit statement that the review methods were established prior to the conduct of the review and did the report justify any significantdeviations from the protocol? | Y | Y | - | Y |
| 3 | Did the review authors explain their selection of the study designs for inclusion in the review? | Y | Y | - | Y |
| 4 | Did the review authors use a comprehensive literature search strategy? | Y | Y | - | Y |
| 5 | Did the review authors perform study selection in duplicate? | Y | Y | - | Y |
| 6 | Did the review authors perform data extraction in duplicate? | Y | Y | - | Y |
| 7 | Did the review authors provide a list of excluded studies and justify the exclusions? | Y | Y | - | Y |
| 8 | Did the review authors describe the included studies in adequate detail? | Y | Y | - | Y |
| 9 | Did the review authors use a satisfactory technique for assessing the risk of bias (RoB) in individual studies that were included in the review? | Y | Y | - | Y |
| 10 | Did the review authors report on the sources of funding for the studies included in the review? | PY | PY | - | PY |
| 11 | If meta-analysis was performed, did the review authors use appropriate methods for statistical combination of results? | Y | Y | - | Y |
| 12 | If meta-analysis was performed, did the review authors assess the potential impact of RoB in individual studies on the results of the meta-analysis or other evidence synthesis? | Y | Y | - | Y |
| 13 | Did the review authors account for RoB in primary studies when interpreting/discussing the results of the review? | Y | Y | - | Y |
| 14 | Did the review authors provide a satisfactory explanation for, and discussion of, any heterogeneity observed in the results of the review? | Y | Y | - | Y |
| 15 | If they performed quantitative synthesis did the review authors carry out an adequate investigation of publication bias (small study bias) and discuss its likely impact on the results of the review? | Y | PY | Y | Y |
| 16 | Did the review authors report any potential sources of conflict of interest, including any funding they received for conducting the review? | Y | Y | - | Y |

AMSTAR-2

Y: Yes; N: No; PY: Partial Yes.

A:The conclusions of researcher Ph.D.Yongxiu Liu.

B:The conclusions of researcher Ph.D. Yuguo Li..

C:In case of a difference of opinions, it shall be adjudicated by Professor Lei Gao.

D:Conclusive conclusion.

Study 38：孙倩.补充维生素D及其类似物对支气管哮喘的影响：一项随机对照试验的荟萃分析[D].南昌大学,2024.DOI:10.27232/d.cnki.gnchu.2024.003676.

| Entry | | A | B | C | D |
| --- | --- | --- | --- | --- | --- |
| 1 | Did the research questions and inclusion criteria for the review include the components of PICO? | Y | Y | - | Y |
| 2 | Did the report of the review contain an explicit statement that the review methods were established prior to the conduct of the review and did the report justify any significantdeviations from the protocol? | PY | N | N | N |
| 3 | Did the review authors explain their selection of the study designs for inclusion in the review? | Y | PY | PY | PY |
| 4 | Did the review authors use a comprehensive literature search strategy? | Y | Y | - | Y |
| 5 | Did the review authors perform study selection in duplicate? | Y | Y | - | Y |
| 6 | Did the review authors perform data extraction in duplicate? | Y | Y | - | Y |
| 7 | Did the review authors provide a list of excluded studies and justify the exclusions? | PY | PY | - | PY |
| 8 | Did the review authors describe the included studies in adequate detail? | Y | Y | - | Y |
| 9 | Did the review authors use a satisfactory technique for assessing the risk of bias (RoB) in individual studies that were included in the review? | Y | Y | - | Y |
| 10 | Did the review authors report on the sources of funding for the studies included in the review? | N | N | - | N |
| 11 | If meta-analysis was performed, did the review authors use appropriate methods for statistical combination of results? | Y | Y | - | Y |
| 12 | If meta-analysis was performed, did the review authors assess the potential impact of RoB in individual studies on the results of the meta-analysis or other evidence synthesis? | PY | PY | - | PY |
| 13 | Did the review authors account for RoB in primary studies when interpreting/discussing the results of the review? | PY | Y | Y | Y |
| 14 | Did the review authors provide a satisfactory explanation for, and discussion of, any heterogeneity observed in the results of the review? | Y | Y | - | Y |
| 15 | If they performed quantitative synthesis did the review authors carry out an adequate investigation of publication bias (small study bias) and discuss its likely impact on the results of the review? | PY | Y | Y | Y |
| 16 | Did the review authors report any potential sources of conflict of interest, including any funding they received for conducting the review? | N | N | - | N |

AMSTAR-2

Y: Yes; N: No; PY: Partial Yes.

A:The conclusions of researcher Ph.D.Yongxiu Liu.

B:The conclusions of researcher Ph.D. Yuguo Li..

C:In case of a difference of opinions, it shall be adjudicated by Professor Lei Gao.

D:Conclusive conclusion.

Study 39：El Abd, Asmae et al. “The effects of vitamin D supplementation on inflammatory biomarkers in patients with asthma: a systematic review and meta-analysis of randomized controlled trials.”Frontiers in immunology vol. 15 1335968. 13 Mar. 2024, doi:10.3389/fimmu.2024.1335968.PMID: 38545098 PMCID: PMC10965564

| Entry | | A | B | C | D |
| --- | --- | --- | --- | --- | --- |
| 1 | Did the research questions and inclusion criteria for the review include the components of PICO? | Y | Y | - | Y |
| 2 | Did the report of the review contain an explicit statement that the review methods were established prior to the conduct of the review and did the report justify any significantdeviations from the protocol? | Y | Y | - | Y |
| 3 | Did the review authors explain their selection of the study designs for inclusion in the review? | Y | Y | - | Y |
| 4 | Did the review authors use a comprehensive literature search strategy? | Y | Y | - | Y |
| 5 | Did the review authors perform study selection in duplicate? | Y | Y | - | Y |
| 6 | Did the review authors perform data extraction in duplicate? | Y | Y | - | Y |
| 7 | Did the review authors provide a list of excluded studies and justify the exclusions? | PY | Y | Y | Y |
| 8 | Did the review authors describe the included studies in adequate detail? | Y | Y | - | Y |
| 9 | Did the review authors use a satisfactory technique for assessing the risk of bias (RoB) in individual studies that were included in the review? | Y | Y | - | Y |
| 10 | Did the review authors report on the sources of funding for the studies included in the review? | N | N | - | N |
| 11 | If meta-analysis was performed, did the review authors use appropriate methods for statistical combination of results? | Y | Y | - | Y |
| 12 | If meta-analysis was performed, did the review authors assess the potential impact of RoB in individual studies on the results of the meta-analysis or other evidence synthesis? | Y | PY | PY | PY |
| 13 | Did the review authors account for RoB in primary studies when interpreting/discussing the results of the review? | Y | Y | - | Y |
| 14 | Did the review authors provide a satisfactory explanation for, and discussion of, any heterogeneity observed in the results of the review? | Y | PY | Y | Y |
| 15 | If they performed quantitative synthesis did the review authors carry out an adequate investigation of publication bias (small study bias) and discuss its likely impact on the results of the review? | N | N | - | N |
| 16 | Did the review authors report any potential sources of conflict of interest, including any funding they received for conducting the review? | Y | Y | - | Y |

AMSTAR-2

Y: Yes; N: No; PY: Partial Yes.

A:The conclusions of researcher Ph.D.Yongxiu Liu.

B:The conclusions of researcher Ph.D. Yuguo Li..

C:In case of a difference of opinions, it shall be adjudicated by Professor Lei Gao.

D:Conclusive conclusion.

Study 40：Fedora, Katherine et al. “Vitamin D supplementation decrease asthma exacerbations in children: a systematic review and meta-analysis of randomized controlled trials.” Annals of medicine vol. 56,1 (2024): 2400313. doi:10.1080/07853890.2024.2400313.PMID:39421966.PMCID: PMC11492411

| Entry | | A | B | C | D |
| --- | --- | --- | --- | --- | --- |
| 1 | Did the research questions and inclusion criteria for the review include the components of PICO? | Y | Y | - | Y |
| 2 | Did the report of the review contain an explicit statement that the review methods were established prior to the conduct of the review and did the report justify any significantdeviations from the protocol? | Y | Y | - | Y |
| 3 | Did the review authors explain their selection of the study designs for inclusion in the review? | Y | Y | - | Y |
| 4 | Did the review authors use a comprehensive literature search strategy? | PY | PY | - | PY |
| 5 | Did the review authors perform study selection in duplicate? | Y | Y | - | Y |
| 6 | Did the review authors perform data extraction in duplicate? | Y | Y | - | Y |
| 7 | Did the review authors provide a list of excluded studies and justify the exclusions? | PY | Y | Y | Y |
| 8 | Did the review authors describe the included studies in adequate detail? | Y | Y | - | Y |
| 9 | Did the review authors use a satisfactory technique for assessing the risk of bias (RoB) in individual studies that were included in the review? | Y | Y | - | Y |
| 10 | Did the review authors report on the sources of funding for the studies included in the review? | N | N | - | N |
| 11 | If meta-analysis was performed, did the review authors use appropriate methods for statistical combination of results? | Y | Y | - | Y |
| 12 | If meta-analysis was performed, did the review authors assess the potential impact of RoB in individual studies on the results of the meta-analysis or other evidence synthesis? | PY | PY | - | PY |
| 13 | Did the review authors account for RoB in primary studies when interpreting/discussing the results of the review? | PY | Y | PY | PY |
| 14 | Did the review authors provide a satisfactory explanation for, and discussion of, any heterogeneity observed in the results of the review? | Y | Y | - | Y |
| 15 | If they performed quantitative synthesis did the review authors carry out an adequate investigation of publication bias (small study bias) and discuss its likely impact on the results of the review? | PY | PY | - | PY |
| 16 | Did the review authors report any potential sources of conflict of interest, including any funding they received for conducting the review? | Y | Y | - | Y |

AMSTAR-2

Y: Yes; N: No; PY: Partial Yes.

A:The conclusions of researcher Ph.D.Yongxiu Liu.

B:The conclusions of researcher Ph.D. Yuguo Li..

C:In case of a difference of opinions, it shall be adjudicated by Professor Lei Gao.

D:Conclusive conclusion.

Study 41：Niu, Haiying et al. “Asthmatic patients with vitamin D deficiency: Can vitamin D supplementation make a difference.” Technology and health care : official journal of the European Society for Engineering and Medicine vol. 32,6 (2024): 3985-4008. doi:10.3233/THC-231462.PMID: 39031398.PMCID: PMC11612934

| Entry | | A | B | C | D |
| --- | --- | --- | --- | --- | --- |
| 1 | Did the research questions and inclusion criteria for the review include the components of PICO? | Y | Y | - | Y |
| 2 | Did the report of the review contain an explicit statement that the review methods were established prior to the conduct of the review and did the report justify any significantdeviations from the protocol? | PY | N | N | N |
| 3 | Did the review authors explain their selection of the study designs for inclusion in the review? | Y | Y | - | Y |
| 4 | Did the review authors use a comprehensive literature search strategy? | Y | PY | Y | Y |
| 5 | Did the review authors perform study selection in duplicate? | Y | Y | - | Y |
| 6 | Did the review authors perform data extraction in duplicate? | Y | Y | - | Y |
| 7 | Did the review authors provide a list of excluded studies and justify the exclusions? | PY | Y | Y | Y |
| 8 | Did the review authors describe the included studies in adequate detail? | Y | Y | - | Y |
| 9 | Did the review authors use a satisfactory technique for assessing the risk of bias (RoB) in individual studies that were included in the review? | Y | Y | - | Y |
| 10 | Did the review authors report on the sources of funding for the studies included in the review? | N | N | - | N |
| 11 | If meta-analysis was performed, did the review authors use appropriate methods for statistical combination of results? | Y | Y | - | Y |
| 12 | If meta-analysis was performed, did the review authors assess the potential impact of RoB in individual studies on the results of the meta-analysis or other evidence synthesis? | N | PY | N | N |
| 13 | Did the review authors account for RoB in primary studies when interpreting/discussing the results of the review? | PY | Y | PY | PY |
| 14 | Did the review authors provide a satisfactory explanation for, and discussion of, any heterogeneity observed in the results of the review? | PY | Y | Y | Y |
| 15 | If they performed quantitative synthesis did the review authors carry out an adequate investigation of publication bias (small study bias) and discuss its likely impact on the results of the review? | Y | PY | Y | Y |
| 16 | Did the review authors report any potential sources of conflict of interest, including any funding they received for conducting the review? | Y | Y | - | Y |

AMSTAR-2

Y: Yes; N: No; PY: Partial Yes.

A:The conclusions of researcher Ph.D.Yongxiu Liu.

B:The conclusions of researcher Ph.D. Yuguo Li..

C:In case of a difference of opinions, it shall be adjudicated by Professor Lei Gao.

D:Conclusive conclusion.

Study 42：杨玉丰,张慧中. 维生素D联合丙酸氟替卡松治疗儿童哮喘有效性的Meta分析[J]. 今日健康,2025(12):105-108. DOI:10.3969/j.issn.1671-5160.2025.12.044.

| Entry | | A | B | C | D |
| --- | --- | --- | --- | --- | --- |
| 1 | Did the research questions and inclusion criteria for the review include the components of PICO? | Y | Y | - | Y |
| 2 | Did the report of the review contain an explicit statement that the review methods were established prior to the conduct of the review and did the report justify any significantdeviations from the protocol? | PY | N | N | N |
| 3 | Did the review authors explain their selection of the study designs for inclusion in the review? | Y | Y | - | Y |
| 4 | Did the review authors use a comprehensive literature search strategy? | PY | PY | - | PY |
| 5 | Did the review authors perform study selection in duplicate? | Y | Y | - | Y |
| 6 | Did the review authors perform data extraction in duplicate? | Y | Y | - | Y |
| 7 | Did the review authors provide a list of excluded studies and justify the exclusions? | PY | Y | Y | Y |
| 8 | Did the review authors describe the included studies in adequate detail? | PY | Y | Y | Y |
| 9 | Did the review authors use a satisfactory technique for assessing the risk of bias (RoB) in individual studies that were included in the review? | Y | Y | - | Y |
| 10 | Did the review authors report on the sources of funding for the studies included in the review? | N | N | - | N |
| 11 | If meta-analysis was performed, did the review authors use appropriate methods for statistical combination of results? | Y | Y | - | Y |
| 12 | If meta-analysis was performed, did the review authors assess the potential impact of RoB in individual studies on the results of the meta-analysis or other evidence synthesis? | N | PY | N | N |
| 13 | Did the review authors account for RoB in primary studies when interpreting/discussing the results of the review? | PY | Y | PY | PY |
| 14 | Did the review authors provide a satisfactory explanation for, and discussion of, any heterogeneity observed in the results of the review? | N | Y | N | N |
| 15 | If they performed quantitative synthesis did the review authors carry out an adequate investigation of publication bias (small study bias) and discuss its likely impact on the results of the review? | N | N | - | N |
| 16 | Did the review authors report any potential sources of conflict of interest, including any funding they received for conducting the review? | N | Y | N | N |

4.PRISMA 2020

Y: Yes; N: No; PY: Partial Yes.

A:The conclusions of researcher Ph.D.Yongxiu Liu.

B:The conclusions of researcher Ph.D. Yuguo Li..

C:In case of a difference of opinions, it shall be adjudicated by Professor Lei Gao.

D:Conclusive conclusion.

Study 22：Fares, Munes M et al. “Vitamin D supplementation in children with asthma: a systematic review and meta-analysis.” BMC research notes vol. 8 23. 3 Feb. 2015, doi:10.1186/s13104-014-0961-3.PMID: 25643669 PMCID: PMC4328422

| Section and topic | Item # | Checklist item | A | B | C | D |
| --- | --- | --- | --- | --- | --- | --- |
| **Title** | | | | | | |
| Title | 1 | Identify the report as a systematic review. | Y | Y | - | Y |
| **Abstract** | | | | | | |
| Abstract | 2 | See the PRISMA 2020 for Abstracts checklist (table 2). | PY | PY | - | PY |
| **Introduction** | | | | | | |
| Rationale | 3 | Describe the rationale for the review in the context of existing knowledge | Y | Y | - | Y |
| Objectives | 4 | Provide an explicit statement of the objective(s) or question(s) the review addresses. | Y | Y | - | Y |
| **Methods** | | | | | | |
| Eligibility criteria | 5 | Specify the inclusion and exclusion criteria for the review and how studies were grouped for the syntheses. | PY | PY | - | PY |
| Information sources | 6 | Specify all databases, registers, websites, organisations, reference lists and other sources searched or consulted to identify studies. Specify the date when each source was last searched or consulted. | PY | Y | Y | Y |
| Search strategy | 7 | Present the full search strategies for all databases, registers and websites, including any filters and limits used | PY | N | Y | Y |
| Selection process | 8 | Specify the methods used to decide whether a study met the inclusion criteria of the review, including how many reviewers screened each record and each report retrieved, whether they worked independently, and if applicable, details of automation tools  used in the process. | Y | Y | - | Y |
| Data collection  process | 9 | Specify the methods used to collect data from reports, including how many reviewers collected data from each report, whether they worked independently, any processes for obtaining or confirming data from study investigators, and if applicable, details of automation tools used in the process. | Y | Y | - | Y |
| Data items | 10a | List and define all outcomes for which data were sought. Specify whether all results that were compatible with each outcome domain in each study were sought (e.g. for all measures, time points, analyses), and if not, the methods used to decide which  results to collect. | Y | PY | Y | Y |
|  | 10b | List and define all other variables for which data were sought (e.g. participant and intervention characteristics, funding sources). Describe any assumptions made about any missing or unclear information. | PY | N | Y | Y |
| Study risk of bias  assessment | 11 | Specify the methods used to assess risk of bias in the included studies, including details of the tool(s) used, how many reviewers assessed each study and whether they worked independently, and if applicable, details of automation tools used in the process. | Y | PY | Y | Y |
| Effect measures | 12 | Specify for each outcome the effect measure(s) (e.g. risk ratio, mean difference) used in the synthesis or presentation of results. | Y | Y | - | Y |
| Synthesis methods | 13a | Describe the processes used to decide which studies were eligible for each synthesis (e.g. tabulating the study intervention characteristics and comparing against the planned groups for each synthesis (item #5)). | N | PY | Y | Y |
|  | 13b | Describe any methods required to prepare the data for presentation or synthesis, such as handling of missing summary statistics, or data conversions. | PY | PY | - | PY |
|  | 13c | Describe any methods used to tabulate or visually display results of individual studies and syntheses. | Y | N | Y | Y |
|  | 13d | Describe any methods used to synthesise results and provide a rationale for the choice(s). If meta-analysis was performed, describe the model(s), method(s) to identify the presence and extent of statistical heterogeneity, and software package(s) used. | Y | Y | - | Y |
|  | 13e | Describe any methods used to explore possible causes of heterogeneity among study results (e.g. subgroup analysis, meta regression). | N | N | - | N |
|  | 13f | Describe any sensitivity analyses conducted to assess robustness of the synthesised results. | N | N | - | N |
| Reporting bias  assessment | 14 | Describe any methods used to assess risk of bias due to missing results in a synthesis (arising from reporting biases). | N | N | - | Y |
| Certainty assessment | 15 | Describe any methods used to assess certainty (or confidence) in the body of evidence for an outcome. | Y | Y | - | Y |
| **Results** | | | | | | |
| Study selection | 16a | Describe the results of the search and selection process, from the number of records identified in the search to the number of studies included in the review, ideally using a flow diagram (see fig 1). | Y | Y | - | Y |
|  | 16b | Cite studies that might appear to meet the inclusion criteria, but which were excluded, and explain why they were excluded. | PY | N | Y | Y |
| Study characteristics | 17 | Cite each included study and present its characteristics. | Y | Y | - | Y |
| Risk of bias in studies | 18 | Present assessments of risk of bias for each included study. | Y | Y | - | Y |
| Results of individual  studies | 19 | For all outcomes, present, for each study: (a) summary statistics for each group (where appropriate) and (b) an effect estimate and its precision (e.g. confidence/credible interval), ideally using structured tables or plots. | PY | PY | - | PY |
| Results of syntheses | 20a | For each synthesis, briefly summarise the characteristics and risk of bias among contributing studies. | Y | Y | - | Y |
|  | 20b | Present results of all statistical syntheses conducted. If meta-analysis was done, present for each the summary estimate and its precision (e.g. confidence/credible interval) and measures of statistical heterogeneity. If comparing groups, describe the direction of the effect. | Y | Y | - | Y |
|  | 20c | Present results of all investigations of possible causes of heterogeneity among study results. | N | N | - | N |
|  | 20d | Present results of all sensitivity analyses conducted to assess the robustness of the synthesised results. | N | N | - | N |
| Reporting biases | 21 | Present assessments of risk of bias due to missing results (arising from reporting biases) for each synthesis assessed. | N | N | - | N |
| Certainty of evidence | 22 | Present assessments of certainty (or confidence) in the body of evidence for each outcome assessed. | Y | Y | - | Y |
| **Discussion** | | | | | | |
| Discussion | 23a | Provide a general interpretation of the results in the context of other evidence. | Y | Y | - | Y |
|  | 23b | Discuss any limitations of the evidence included in the review. | Y | Y | - | Y |
|  | 23c | Discuss any limitations of the review processes used. | Y | PY | Y | Y |
|  | 23d | Discuss implications of the results for practice, policy, and future research. | Y | Y | - | Y |
| **Other information** | | | | | | |
| Registration and  protocol | 24a | Provide registration information for the review, including register name and registration number, or state that the review was not registered. | Y | Y | - | Y |
|  | 24b | Indicate where the review protocol can be accessed, or state that a protocol was not prepared. | N | N | - | N |
|  | 24c | Describe and explain any amendments to information provided at registration or in the protocol. | Y | N | N | N |
| Support | 25 | Describe sources of financial or non-financial support for the review, and the role of the funders or sponsors in the review. | PY | N | N | N |
| Competing interests | 26 | Declare any competing interests of review authors. | Y | Y | - | Y |
| Availability of data,  code, and other  materials | 27 | Report which of the following are publicly available and where they can be found: template data collection forms; data extracted from included studies; data used for all analyses; analytic code; any other materials used in the review. | N | N | - | N |

PRISMA 2020

Y: Yes; N: No; PY: Partial Yes.

A:The conclusions of researcher Ph.D.Yongxiu Liu.

B:The conclusions of researcher Ph.D. Yuguo Li..

C:In case of a difference of opinions, it shall be adjudicated by Professor Lei Gao.

D:Conclusive conclusion.

Study 23：Luo, Jian et al. “Can Vitamin D Supplementation in Addition to Asthma Controllers Improve Clinical Outcomes in Patients With Asthma?: A Meta-Analysis.” Medicine vol. 94,50 (2015): e2185. doi:10.1097/MD.0000000000002185.PMID: 26683927 PMCID: PMC5058899

| Section and topic | Item # | Checklist item | A | B | C | D |
| --- | --- | --- | --- | --- | --- | --- |
| **Title** | | | | | | |
| Title | 1 | Identify the report as a systematic review. | Y | Y | - | Y |
| **Abstract** | | | | | | |
| Abstract | 2 | See the PRISMA 2020 for Abstracts checklist (table 2). | PY | PY | - | PY |
| **Introduction** | | | | | | |
| Rationale | 3 | Describe the rationale for the review in the context of existing knowledge | Y | Y | - | Y |
| Objectives | 4 | Provide an explicit statement of the objective(s) or question(s) the review addresses. | Y | Y | - | Y |
| **Methods** | | | | | | |
| Eligibility criteria | 5 | Specify the inclusion and exclusion criteria for the review and how studies were grouped for the syntheses. | PY | PY | - | PY |
| Information sources | 6 | Specify all databases, registers, websites, organisations, reference lists and other sources searched or consulted to identify studies. Specify the date when each source was last searched or consulted. | Y | Y | - | Y |
| Search strategy | 7 | Present the full search strategies for all databases, registers and websites, including any filters and limits used | N | N | - | N |
| Selection process | 8 | Specify the methods used to decide whether a study met the inclusion criteria of the review, including how many reviewers screened each record and each report retrieved, whether they worked independently, and if applicable, details of automation tools  used in the process. | Y | Y | - | Y |
| Data collection  process | 9 | Specify the methods used to collect data from reports, including how many reviewers collected data from each report, whether they worked independently, any processes for obtaining or confirming data from study investigators, and if applicable, details of automation tools used in the process. | Y | Y | - | Y |
| Data items | 10a | List and define all outcomes for which data were sought. Specify whether all results that were compatible with each outcome domain in each study were sought (e.g. for all measures, time points, analyses), and if not, the methods used to decide which  results to collect. | Y | PY | Y | Y |
|  | 10b | List and define all other variables for which data were sought (e.g. participant and intervention characteristics, funding sources). Describe any assumptions made about any missing or unclear information. | PY | N | Y | Y |
| Study risk of bias  assessment | 11 | Specify the methods used to assess risk of bias in the included studies, including details of the tool(s) used, how many reviewers assessed each study and whether they worked independently, and if applicable, details of automation tools used in the process. | Y | PY | Y | Y |
| Effect measures | 12 | Specify for each outcome the effect measure(s) (e.g. risk ratio, mean difference) used in the synthesis or presentation of results. | Y | Y | - | Y |
| Synthesis methods | 13a | Describe the processes used to decide which studies were eligible for each synthesis (e.g. tabulating the study intervention characteristics and comparing against the planned groups for each synthesis (item #5)). | N | PY | Y | Y |
|  | 13b | Describe any methods required to prepare the data for presentation or synthesis, such as handling of missing summary statistics, or data conversions. | N | PY | N | N |
|  | 13c | Describe any methods used to tabulate or visually display results of individual studies and syntheses. | Y | N | Y | Y |
|  | 13d | Describe any methods used to synthesise results and provide a rationale for the choice(s). If meta-analysis was performed, describe the model(s), method(s) to identify the presence and extent of statistical heterogeneity, and software package(s) used. | Y | Y | - | Y |
|  | 13e | Describe any methods used to explore possible causes of heterogeneity among study results (e.g. subgroup analysis, meta regression). | N | N | - | N |
|  | 13f | Describe any sensitivity analyses conducted to assess robustness of the synthesised results. | N | PY | Y | Y |
| Reporting bias  assessment | 14 | Describe any methods used to assess risk of bias due to missing results in a synthesis (arising from reporting biases). | N | N | - | N |
| Certainty assessment | 15 | Describe any methods used to assess certainty (or confidence) in the body of evidence for an outcome. | N | N | - | N |
| **Results** | | | | | | |
| Study selection | 16a | Describe the results of the search and selection process, from the number of records identified in the search to the number of studies included in the review, ideally using a flow diagram (see fig 1). | Y | Y | - | Y |
|  | 16b | Cite studies that might appear to meet the inclusion criteria, but which were excluded, and explain why they were excluded. | PY | N | N | N |
| Study characteristics | 17 | Cite each included study and present its characteristics. | Y | Y | - | Y |
| Risk of bias in studies | 18 | Present assessments of risk of bias for each included study. | Y | Y | - | Y |
| Results of individual  studies | 19 | For all outcomes, present, for each study: (a) summary statistics for each group (where appropriate) and (b) an effect estimate and its precision (e.g. confidence/credible interval), ideally using structured tables or plots. | Y | Y | - | Y |
| Results of syntheses | 20a | For each synthesis, briefly summarise the characteristics and risk of bias among contributing studies. | Y | Y | - | Y |
|  | 20b | Present results of all statistical syntheses conducted. If meta-analysis was done, present for each the summary estimate and its precision (e.g. confidence/credible interval) and measures of statistical heterogeneity. If comparing groups, describe the direction of the effect. | Y | Y | - | Y |
|  | 20c | Present results of all investigations of possible causes of heterogeneity among study results. | N | N | - | N |
|  | 20d | Present results of all sensitivity analyses conducted to assess the robustness of the synthesised results. | N | PY | Y | Y |
| Reporting biases | 21 | Present assessments of risk of bias due to missing results (arising from reporting biases) for each synthesis assessed. | N | N | - | N |
| Certainty of evidence | 22 | Present assessments of certainty (or confidence) in the body of evidence for each outcome assessed. | N | N | - | N |
| **Discussion** | | | | | | |
| Discussion | 23a | Provide a general interpretation of the results in the context of other evidence. | Y | Y | - | Y |
|  | 23b | Discuss any limitations of the evidence included in the review. | Y | Y | - | Y |
|  | 23c | Discuss any limitations of the review processes used. | Y | PY | Y | Y |
|  | 23d | Discuss implications of the results for practice, policy, and future research. | Y | Y | - | Y |
| **Other information** | | | | | | |
| Registration and  protocol | 24a | Provide registration information for the review, including register name and registration number, or state that the review was not registered. | N | N | - | N |
|  | 24b | Indicate where the review protocol can be accessed, or state that a protocol was not prepared. | N | N | - | N |
|  | 24c | Describe and explain any amendments to information provided at registration or in the protocol. | Y | N | N | N |
| Support | 25 | Describe sources of financial or non-financial support for the review, and the role of the funders or sponsors in the review. | Y | N | Y | Y |
| Competing interests | 26 | Declare any competing interests of review authors. | Y | Y | - | Y |
| Availability of data,  code, and other  materials | 27 | Report which of the following are publicly available and where they can be found: template data collection forms; data extracted from included studies; data used for all analyses; analytic code; any other materials used in the review. | N | N | - | N |

PRISMA 2020

Y: Yes; N: No; PY: Partial Yes.

A:The conclusions of researcher Ph.D.Yongxiu Liu.

B:The conclusions of researcher Ph.D. Yuguo Li..

C:In case of a difference of opinions, it shall be adjudicated by Professor Lei Gao.

D:Conclusive conclusion.

Study 24：Pojsupap, Supichaya et al. “Efficacy of high-dose vitamin D in pediatric asthma: a systematic review and meta-analysis.” The Journal of asthma : official journal of the Association for the Care of Asthma vol. 52,4 (2015): 382-90. doi:10.3109/02770903.2014.980509.PMID: 25365192

| Section and topic | Item # | Checklist item | A | B | C | D |
| --- | --- | --- | --- | --- | --- | --- |
| **Title** | | | | | | |
| Title | 1 | Identify the report as a systematic review. | Y | Y | - | Y |
| **Abstract** | | | | | | |
| Abstract | 2 | See the PRISMA 2020 for Abstracts checklist (table 2). | PY | PY | - | PY |
| **Introduction** | | | | | | |
| Rationale | 3 | Describe the rationale for the review in the context of existing knowledge | Y | Y | - | Y |
| Objectives | 4 | Provide an explicit statement of the objective(s) or question(s) the review addresses. | Y | Y | - | Y |
| **Methods** | | | | | | |
| Eligibility criteria | 5 | Specify the inclusion and exclusion criteria for the review and how studies were grouped for the syntheses. | PY | Y | Y | Y |
| Information sources | 6 | Specify all databases, registers, websites, organisations, reference lists and other sources searched or consulted to identify studies. Specify the date when each source was last searched or consulted. | Y | Y | - | Y |
| Search strategy | 7 | Present the full search strategies for all databases, registers and websites, including any filters and limits used | PY | PY | - | PY |
| Selection process | 8 | Specify the methods used to decide whether a study met the inclusion criteria of the review, including how many reviewers screened each record and each report retrieved, whether they worked independently, and if applicable, details of automation tools  used in the process. | Y | Y | - | Y |
| Data collection  process | 9 | Specify the methods used to collect data from reports, including how many reviewers collected data from each report, whether they worked independently, any processes for obtaining or confirming data from study investigators, and if applicable, details of automation tools used in the process. | Y | Y | - | Y |
| Data items | 10a | List and define all outcomes for which data were sought. Specify whether all results that were compatible with each outcome domain in each study were sought (e.g. for all measures, time points, analyses), and if not, the methods used to decide which  results to collect. | Y | Y | - | Y |
|  | 10b | List and define all other variables for which data were sought (e.g. participant and intervention characteristics, funding sources). Describe any assumptions made about any missing or unclear information. | PY | PY | - | PY |
| Study risk of bias  assessment | 11 | Specify the methods used to assess risk of bias in the included studies, including details of the tool(s) used, how many reviewers assessed each study and whether they worked independently, and if applicable, details of automation tools used in the process. | Y | Y | - | Y |
| Effect measures | 12 | Specify for each outcome the effect measure(s) (e.g. risk ratio, mean difference) used in the synthesis or presentation of results. | Y | Y | - | Y |
| Synthesis methods | 13a | Describe the processes used to decide which studies were eligible for each synthesis (e.g. tabulating the study intervention characteristics and comparing against the planned groups for each synthesis (item #5)). | N | Y | Y | Y |
|  | 13b | Describe any methods required to prepare the data for presentation or synthesis, such as handling of missing summary statistics, or data conversions. | PY | Y | Y | Y |
|  | 13c | Describe any methods used to tabulate or visually display results of individual studies and syntheses. | Y | Y | - | Y |
|  | 13d | Describe any methods used to synthesise results and provide a rationale for the choice(s). If meta-analysis was performed, describe the model(s), method(s) to identify the presence and extent of statistical heterogeneity, and software package(s) used. | Y | Y | - | Y |
|  | 13e | Describe any methods used to explore possible causes of heterogeneity among study results (e.g. subgroup analysis, meta regression). | N | N | - | N |
|  | 13f | Describe any sensitivity analyses conducted to assess robustness of the synthesised results. | PY | Y | Y | Y |
| Reporting bias  assessment | 14 | Describe any methods used to assess risk of bias due to missing results in a synthesis (arising from reporting biases). | N | N | - | N |
| Certainty assessment | 15 | Describe any methods used to assess certainty (or confidence) in the body of evidence for an outcome. | N | N | - | N |
| **Results** | | | | | | |
| Study selection | 16a | Describe the results of the search and selection process, from the number of records identified in the search to the number of studies included in the review, ideally using a flow diagram (see fig 1). | Y | Y | - | Y |
|  | 16b | Cite studies that might appear to meet the inclusion criteria, but which were excluded, and explain why they were excluded. | PY | PY | - | PY |
| Study characteristics | 17 | Cite each included study and present its characteristics. | Y | Y | - | Y |
| Risk of bias in studies | 18 | Present assessments of risk of bias for each included study. | Y | Y | - | Y |
| Results of individual  studies | 19 | For all outcomes, present, for each study: (a) summary statistics for each group (where appropriate) and (b) an effect estimate and its precision (e.g. confidence/credible interval), ideally using structured tables or plots. | Y | Y | - | Y |
| Results of syntheses | 20a | For each synthesis, briefly summarise the characteristics and risk of bias among contributing studies. | Y | Y | - | Y |
|  | 20b | Present results of all statistical syntheses conducted. If meta-analysis was done, present for each the summary estimate and its precision (e.g. confidence/credible interval) and measures of statistical heterogeneity. If comparing groups, describe the direction of the effect. | Y | Y | - | Y |
|  | 20c | Present results of all investigations of possible causes of heterogeneity among study results. | N | N | - | N |
|  | 20d | Present results of all sensitivity analyses conducted to assess the robustness of the synthesised results. | Y | Y | - | Y |
| Reporting biases | 21 | Present assessments of risk of bias due to missing results (arising from reporting biases) for each synthesis assessed. | N | N | - | N |
| Certainty of evidence | 22 | Present assessments of certainty (or confidence) in the body of evidence for each outcome assessed. | N | N | - | N |
| **Discussion** | | | | | | |
| Discussion | 23a | Provide a general interpretation of the results in the context of other evidence. | Y | Y | - | Y |
|  | 23b | Discuss any limitations of the evidence included in the review. | Y | Y | - | Y |
|  | 23c | Discuss any limitations of the review processes used. | Y | Y | - | Y |
|  | 23d | Discuss implications of the results for practice, policy, and future research. | Y | Y | - | Y |
| **Other information** | | | | | | |
| Registration and  protocol | 24a | Provide registration information for the review, including register name and registration number, or state that the review was not registered. | N | N | - | N |
|  | 24b | Indicate where the review protocol can be accessed, or state that a protocol was not prepared. | PY | N | N | N |
|  | 24c | Describe and explain any amendments to information provided at registration or in the protocol. | Y | N | N | N |
| Support | 25 | Describe sources of financial or non-financial support for the review, and the role of the funders or sponsors in the review. | Y | PY | Y | Y |
| Competing interests | 26 | Declare any competing interests of review authors. | Y | Y | - | Y |
| Availability of data,  code, and other  materials | 27 | Report which of the following are publicly available and where they can be found: template data collection forms; data extracted from included studies; data used for all analyses; analytic code; any other materials used in the review. | PY | N | N | N |

PRISMA 2020

Y: Yes; N: No; PY: Partial Yes.

A:The conclusions of researcher Ph.D.Yongxiu Liu.

B:The conclusions of researcher Ph.D. Yuguo Li.

C:In case of a difference of opinions, it shall be adjudicated by Professor Lei Gao.

D:Conclusive conclusion.

Study 25：Riverin, Bruno D et al. “Vitamin D Supplementation for Childhood Asthma: A Systematic Review and Meta-Analysis.” PloS one vol. 10,8 e0136841. 31 Aug. 2015, doi:10.1371/journal.pone.0136841.PMID: 26322509 PMCID: PMC4556456

| Section and topic | Item # | Checklist item | A | B | C | D |
| --- | --- | --- | --- | --- | --- | --- |
| **Title** | | | | | | |
| Title | 1 | Identify the report as a systematic review. | Y | Y | - | Y |
| **Abstract** | | | | | | |
| Abstract | 2 | See the PRISMA 2020 for Abstracts checklist (table 2). | PY | PY | - | PY |
| **Introduction** | | | | | | |
| Rationale | 3 | Describe the rationale for the review in the context of existing knowledge | Y | Y | - | Y |
| Objectives | 4 | Provide an explicit statement of the objective(s) or question(s) the review addresses. | Y | Y | - | Y |
| **Methods** | | | | | | |
| Eligibility criteria | 5 | Specify the inclusion and exclusion criteria for the review and how studies were grouped for the syntheses. | PY | Y | Y | Y |
| Information sources | 6 | Specify all databases, registers, websites, organisations, reference lists and other sources searched or consulted to identify studies. Specify the date when each source was last searched or consulted. | Y | Y | - | Y |
| Search strategy | 7 | Present the full search strategies for all databases, registers and websites, including any filters and limits used | PY | PY | - | PY |
| Selection process | 8 | Specify the methods used to decide whether a study met the inclusion criteria of the review, including how many reviewers screened each record and each report retrieved, whether they worked independently, and if applicable, details of automation tools  used in the process. | Y | PY | Y | Y |
| Data collection  process | 9 | Specify the methods used to collect data from reports, including how many reviewers collected data from each report, whether they worked independently, any processes for obtaining or confirming data from study investigators, and if applicable, details of automation tools used in the process. | Y | PY | Y | Y |
| Data items | 10a | List and define all outcomes for which data were sought. Specify whether all results that were compatible with each outcome domain in each study were sought (e.g. for all measures, time points, analyses), and if not, the methods used to decide which  results to collect. | Y | Y | - | Y |
|  | 10b | List and define all other variables for which data were sought (e.g. participant and intervention characteristics, funding sources). Describe any assumptions made about any missing or unclear information. | PY | PY | - | PY |
| Study risk of bias  assessment | 11 | Specify the methods used to assess risk of bias in the included studies, including details of the tool(s) used, how many reviewers assessed each study and whether they worked independently, and if applicable, details of automation tools used in the process. | Y | PY | Y | Y |
| Effect measures | 12 | Specify for each outcome the effect measure(s) (e.g. risk ratio, mean difference) used in the synthesis or presentation of results. | Y | Y | - | Y |
| Synthesis methods | 13a | Describe the processes used to decide which studies were eligible for each synthesis (e.g. tabulating the study intervention characteristics and comparing against the planned groups for each synthesis (item #5)). | N | Y | Y | Y |
|  | 13b | Describe any methods required to prepare the data for presentation or synthesis, such as handling of missing summary statistics, or data conversions. | PY | Y | Y | Y |
|  | 13c | Describe any methods used to tabulate or visually display results of individual studies and syntheses. | Y | Y | - | Y |
|  | 13d | Describe any methods used to synthesise results and provide a rationale for the choice(s). If meta-analysis was performed, describe the model(s), method(s) to identify the presence and extent of statistical heterogeneity, and software package(s) used. | Y | Y | - | Y |
|  | 13e | Describe any methods used to explore possible causes of heterogeneity among study results (e.g. subgroup analysis, meta regression). | PY | PY | - | PY |
|  | 13f | Describe any sensitivity analyses conducted to assess robustness of the synthesised results. | N | N | - | N |
| Reporting bias  assessment | 14 | Describe any methods used to assess risk of bias due to missing results in a synthesis (arising from reporting biases). | PY | Y | Y | Y |
| Certainty assessment | 15 | Describe any methods used to assess certainty (or confidence) in the body of evidence for an outcome. | Y | Y | - | Y |
| **Results** | | | | | | |
| Study selection | 16a | Describe the results of the search and selection process, from the number of records identified in the search to the number of studies included in the review, ideally using a flow diagram (see fig 1). | Y | Y | - | Y |
|  | 16b | Cite studies that might appear to meet the inclusion criteria, but which were excluded, and explain why they were excluded. | PY | N | Y | Y |
| Study characteristics | 17 | Cite each included study and present its characteristics. | Y | Y | - | Y |
| Risk of bias in studies | 18 | Present assessments of risk of bias for each included study. | Y | PY | Y | Y |
| Results of individual  studies | 19 | For all outcomes, present, for each study: (a) summary statistics for each group (where appropriate) and (b) an effect estimate and its precision (e.g. confidence/credible interval), ideally using structured tables or plots. | Y | Y | - | Y |
| Results of syntheses | 20a | For each synthesis, briefly summarise the characteristics and risk of bias among contributing studies. | Y | PY | Y | Y |
|  | 20b | Present results of all statistical syntheses conducted. If meta-analysis was done, present for each the summary estimate and its precision (e.g. confidence/credible interval) and measures of statistical heterogeneity. If comparing groups, describe the direction of the effect. | Y | Y | - | Y |
|  | 20c | Present results of all investigations of possible causes of heterogeneity among study results. | N | PY | Y | Y |
|  | 20d | Present results of all sensitivity analyses conducted to assess the robustness of the synthesised results. | N | N | - | N |
| Reporting biases | 21 | Present assessments of risk of bias due to missing results (arising from reporting biases) for each synthesis assessed. | PY | PY | - | PY |
| Certainty of evidence | 22 | Present assessments of certainty (or confidence) in the body of evidence for each outcome assessed. | Y | Y | - | Y |
| **Discussion** | | | | | | |
| Discussion | 23a | Provide a general interpretation of the results in the context of other evidence. | Y | Y | - | Y |
|  | 23b | Discuss any limitations of the evidence included in the review. | Y | Y | - | Y |
|  | 23c | Discuss any limitations of the review processes used. | Y | PY | Y | Y |
|  | 23d | Discuss implications of the results for practice, policy, and future research. | Y | Y | - | Y |
| **Other information** | | | | | | |
| Registration and  protocol | 24a | Provide registration information for the review, including register name and registration number, or state that the review was not registered. | N | N | - | N |
|  | 24b | Indicate where the review protocol can be accessed, or state that a protocol was not prepared. | N | N | - | N |
|  | 24c | Describe and explain any amendments to information provided at registration or in the protocol. | Y | N | N | N |
| Support | 25 | Describe sources of financial or non-financial support for the review, and the role of the funders or sponsors in the review. | Y | Y | - | Y |
| Competing interests | 26 | Declare any competing interests of review authors. | Y | Y | - | Y |
| Availability of data,  code, and other  materials | 27 | Report which of the following are publicly available and where they can be found: template data collection forms; data extracted from included studies; data used for all analyses; analytic code; any other materials used in the review. | Y | PY | Y | Y |

PRISMA 2020

Y: Yes; N: No; PY: Partial Yes.

A:The conclusions of researcher Ph.D.Yongxiu Liu.

B:The conclusions of researcher Ph.D. Yuguo Li.

C:In case of a difference of opinions, it shall be adjudicated by Professor Lei Gao.

D:Conclusive conclusion.

Study 26：景伟超,刘璐佳,关洋洋,等.维生素D辅助治疗儿童哮喘Meta分析[J].世界中西医结合杂志,2017,12(10):1341-1344+1354.DOI:10.13935/j.cnki.sjzx.171003.

| Section and topic | Item # | Checklist item | A | B | C | D |
| --- | --- | --- | --- | --- | --- | --- |
| **Title** | | | | | | |
| Title | 1 | Identify the report as a systematic review. | Y | Y | - | Y |
| **Abstract** | | | | | | |
| Abstract | 2 | See the PRISMA 2020 for Abstracts checklist (table 2). | PY | PY | - | PY |
| **Introduction** | | | | | | |
| Rationale | 3 | Describe the rationale for the review in the context of existing knowledge | Y | Y | - | Y |
| Objectives | 4 | Provide an explicit statement of the objective(s) or question(s) the review addresses. | Y | Y | - | Y |
| **Methods** | | | | | | |
| Eligibility criteria | 5 | Specify the inclusion and exclusion criteria for the review and how studies were grouped for the syntheses. | PY | Y | Y | Y |
| Information sources | 6 | Specify all databases, registers, websites, organisations, reference lists and other sources searched or consulted to identify studies. Specify the date when each source was last searched or consulted. | Y | PY | Y | Y |
| Search strategy | 7 | Present the full search strategies for all databases, registers and websites, including any filters and limits used | Y | N | N | N |
| Selection process | 8 | Specify the methods used to decide whether a study met the inclusion criteria of the review, including how many reviewers screened each record and each report retrieved, whether they worked independently, and if applicable, details of automation tools  used in the process. | Y | PY | Y | Y |
| Data collection  process | 9 | Specify the methods used to collect data from reports, including how many reviewers collected data from each report, whether they worked independently, any processes for obtaining or confirming data from study investigators, and if applicable, details of automation tools used in the process. | Y | PY | Y | Y |
| Data items | 10a | List and define all outcomes for which data were sought. Specify whether all results that were compatible with each outcome domain in each study were sought (e.g. for all measures, time points, analyses), and if not, the methods used to decide which  results to collect. | Y | Y | - | Y |
|  | 10b | List and define all other variables for which data were sought (e.g. participant and intervention characteristics, funding sources). Describe any assumptions made about any missing or unclear information. | PY | PY | - | PY |
| Study risk of bias  assessment | 11 | Specify the methods used to assess risk of bias in the included studies, including details of the tool(s) used, how many reviewers assessed each study and whether they worked independently, and if applicable, details of automation tools used in the process. | PY | PY | - | PY |
| Effect measures | 12 | Specify for each outcome the effect measure(s) (e.g. risk ratio, mean difference) used in the synthesis or presentation of results. | Y | Y | - | Y |
| Synthesis methods | 13a | Describe the processes used to decide which studies were eligible for each synthesis (e.g. tabulating the study intervention characteristics and comparing against the planned groups for each synthesis (item #5)). | N | Y | Y | Y |
|  | 13b | Describe any methods required to prepare the data for presentation or synthesis, such as handling of missing summary statistics, or data conversions. | N | Y | Y | Y |
|  | 13c | Describe any methods used to tabulate or visually display results of individual studies and syntheses. | Y | Y | - | Y |
|  | 13d | Describe any methods used to synthesise results and provide a rationale for the choice(s). If meta-analysis was performed, describe the model(s), method(s) to identify the presence and extent of statistical heterogeneity, and software package(s) used. | Y | PY | Y | Y |
|  | 13e | Describe any methods used to explore possible causes of heterogeneity among study results (e.g. subgroup analysis, meta regression). | N | N | - | N |
|  | 13f | Describe any sensitivity analyses conducted to assess robustness of the synthesised results. | N | N | - | N |
| Reporting bias  assessment | 14 | Describe any methods used to assess risk of bias due to missing results in a synthesis (arising from reporting biases). | PY | PY | - | PY |
| Certainty assessment | 15 | Describe any methods used to assess certainty (or confidence) in the body of evidence for an outcome. | N | N | - | N |
| **Results** | | | | | | |
| Study selection | 16a | Describe the results of the search and selection process, from the number of records identified in the search to the number of studies included in the review, ideally using a flow diagram (see fig 1). | Y | Y | - | Y |
|  | 16b | Cite studies that might appear to meet the inclusion criteria, but which were excluded, and explain why they were excluded. | PY | N | PY | PY |
| Study characteristics | 17 | Cite each included study and present its characteristics. | Y | Y | - | Y |
| Risk of bias in studies | 18 | Present assessments of risk of bias for each included study. | N | PY | Y | Y |
| Results of individual  studies | 19 | For all outcomes, present, for each study: (a) summary statistics for each group (where appropriate) and (b) an effect estimate and its precision (e.g. confidence/credible interval), ideally using structured tables or plots. | Y | Y | - | Y |
| Results of syntheses | 20a | For each synthesis, briefly summarise the characteristics and risk of bias among contributing studies. | PY | PY | - | PY |
|  | 20b | Present results of all statistical syntheses conducted. If meta-analysis was done, present for each the summary estimate and its precision (e.g. confidence/credible interval) and measures of statistical heterogeneity. If comparing groups, describe the direction of the effect. | Y | Y | - | Y |
|  | 20c | Present results of all investigations of possible causes of heterogeneity among study results. | N | PY | Y | Y |
|  | 20d | Present results of all sensitivity analyses conducted to assess the robustness of the synthesised results. | N | N | - | N |
| Reporting biases | 21 | Present assessments of risk of bias due to missing results (arising from reporting biases) for each synthesis assessed. | PY | PY | - | PY |
| Certainty of evidence | 22 | Present assessments of certainty (or confidence) in the body of evidence for each outcome assessed. | N | N | - | N |
| **Discussion** | | | | | | |
| Discussion | 23a | Provide a general interpretation of the results in the context of other evidence. | Y | Y | - | Y |
|  | 23b | Discuss any limitations of the evidence included in the review. | Y | Y | - | Y |
|  | 23c | Discuss any limitations of the review processes used. | PY | PY | - | PY |
|  | 23d | Discuss implications of the results for practice, policy, and future research. | Y | Y | - | Y |
| **Other information** | | | | | | |
| Registration and  protocol | 24a | Provide registration information for the review, including register name and registration number, or state that the review was not registered. | N | N | - | N |
|  | 24b | Indicate where the review protocol can be accessed, or state that a protocol was not prepared. | N | N | - | N |
|  | 24c | Describe and explain any amendments to information provided at registration or in the protocol. | Y | N | N | N |
| Support | 25 | Describe sources of financial or non-financial support for the review, and the role of the funders or sponsors in the review. | Y | PY | Y | Y |
| Competing interests | 26 | Declare any competing interests of review authors. | Y | N | N | N |
| Availability of data,  code, and other  materials | 27 | Report which of the following are publicly available and where they can be found: template data collection forms; data extracted from included studies; data used for all analyses; analytic code; any other materials used in the review. | N | PY | N | N |

PRISMA 2020

Y: Yes; N: No; PY: Partial Yes.

A:The conclusions of researcher Ph.D.Yongxiu Liu.

B:The conclusions of researcher Ph.D. Yuguo Li..

C:In case of a difference of opinions, it shall be adjudicated by Professor Lei Gao.

D:Conclusive conclusion.

Study 27：Jolliffe, David A et al. “Vitamin D supplementation to prevent asthma exacerbations: a systematic review and meta-analysis of individual participant data.” The Lancet. Respiratory medicine vol. 5,11 (2017): 881-890. doi:10.1016/S2213-2600(17)30306-5.PMID: 28986128 PMCID: PMC5693329

| Section and topic | Item # | Checklist item | A | B | C | D |
| --- | --- | --- | --- | --- | --- | --- |
| **Title** | | | | | | |
| Title | 1 | Identify the report as a systematic review. | Y | Y | - | Y |
| **Abstract** | | | | | | |
| Abstract | 2 | See the PRISMA 2020 for Abstracts checklist (table 2). | PY | PY | - | PY |
| **Introduction** | | | | | | |
| Rationale | 3 | Describe the rationale for the review in the context of existing knowledge | Y | Y | - | Y |
| Objectives | 4 | Provide an explicit statement of the objective(s) or question(s) the review addresses. | Y | Y | - | Y |
| **Methods** | | | | | | |
| Eligibility criteria | 5 | Specify the inclusion and exclusion criteria for the review and how studies were grouped for the syntheses. | Y | Y | - | Y |
| Information sources | 6 | Specify all databases, registers, websites, organisations, reference lists and other sources searched or consulted to identify studies. Specify the date when each source was last searched or consulted. | Y | PY | Y | Y |
| Search strategy | 7 | Present the full search strategies for all databases, registers and websites, including any filters and limits used | PY | PY | - | PY |
| Selection process | 8 | Specify the methods used to decide whether a study met the inclusion criteria of the review, including how many reviewers screened each record and each report retrieved, whether they worked independently, and if applicable, details of automation tools  used in the process. | Y | PY | Y | Y |
| Data collection  process | 9 | Specify the methods used to collect data from reports, including how many reviewers collected data from each report, whether they worked independently, any processes for obtaining or confirming data from study investigators, and if applicable, details of automation tools used in the process. | Y | Y | - | Y |
| Data items | 10a | List and define all outcomes for which data were sought. Specify whether all results that were compatible with each outcome domain in each study were sought (e.g. for all measures, time points, analyses), and if not, the methods used to decide which  results to collect. | Y | Y | - | Y |
|  | 10b | List and define all other variables for which data were sought (e.g. participant and intervention characteristics, funding sources). Describe any assumptions made about any missing or unclear information. | Y | Y | - | Y |
| Study risk of bias  assessment | 11 | Specify the methods used to assess risk of bias in the included studies, including details of the tool(s) used, how many reviewers assessed each study and whether they worked independently, and if applicable, details of automation tools used in the process. | Y | Y | - | Y |
| Effect measures | 12 | Specify for each outcome the effect measure(s) (e.g. risk ratio, mean difference) used in the synthesis or presentation of results. | Y | Y | - | Y |
| Synthesis methods | 13a | Describe the processes used to decide which studies were eligible for each synthesis (e.g. tabulating the study intervention characteristics and comparing against the planned groups for each synthesis (item #5)). | Y | Y | - | Y |
|  | 13b | Describe any methods required to prepare the data for presentation or synthesis, such as handling of missing summary statistics, or data conversions. | Y | Y | - | Y |
|  | 13c | Describe any methods used to tabulate or visually display results of individual studies and syntheses. | Y | Y | - | Y |
|  | 13d | Describe any methods used to synthesise results and provide a rationale for the choice(s). If meta-analysis was performed, describe the model(s), method(s) to identify the presence and extent of statistical heterogeneity, and software package(s) used. | Y | Y | - | Y |
|  | 13e | Describe any methods used to explore possible causes of heterogeneity among study results (e.g. subgroup analysis, meta regression). | Y | Y | - | Y |
|  | 13f | Describe any sensitivity analyses conducted to assess robustness of the synthesised results. | Y | PY | Y | Y |
| Reporting bias  assessment | 14 | Describe any methods used to assess risk of bias due to missing results in a synthesis (arising from reporting biases). | Y | PY | Y | Y |
| Certainty assessment | 15 | Describe any methods used to assess certainty (or confidence) in the body of evidence for an outcome. | Y | Y | - | Y |
| **Results** | | | | | | |
| Study selection | 16a | Describe the results of the search and selection process, from the number of records identified in the search to the number of studies included in the review, ideally using a flow diagram (see fig 1). | Y | Y | - | Y |
|  | 16b | Cite studies that might appear to meet the inclusion criteria, but which were excluded, and explain why they were excluded. | Y | PY | Y | Y |
| Study characteristics | 17 | Cite each included study and present its characteristics. | Y | Y | - | Y |
| Risk of bias in studies | 18 | Present assessments of risk of bias for each included study. | Y | Y | - | Y |
| Results of individual  studies | 19 | For all outcomes, present, for each study: (a) summary statistics for each group (where appropriate) and (b) an effect estimate and its precision (e.g. confidence/credible interval), ideally using structured tables or plots. | Y | Y | - | Y |
| Results of syntheses | 20a | For each synthesis, briefly summarise the characteristics and risk of bias among contributing studies. | Y | Y | - | Y |
|  | 20b | Present results of all statistical syntheses conducted. If meta-analysis was done, present for each the summary estimate and its precision (e.g. confidence/credible interval) and measures of statistical heterogeneity. If comparing groups, describe the direction of the effect. | Y | Y | - | Y |
|  | 20c | Present results of all investigations of possible causes of heterogeneity among study results. | Y | Y | - | Y |
|  | 20d | Present results of all sensitivity analyses conducted to assess the robustness of the synthesised results. | Y | PY | Y | Y |
| Reporting biases | 21 | Present assessments of risk of bias due to missing results (arising from reporting biases) for each synthesis assessed. | Y | PY | Y | Y |
| Certainty of evidence | 22 | Present assessments of certainty (or confidence) in the body of evidence for each outcome assessed. | Y | Y | - | Y |
| **Discussion** | | | | | | |
| Discussion | 23a | Provide a general interpretation of the results in the context of other evidence. | Y | Y | - | Y |
|  | 23b | Discuss any limitations of the evidence included in the review. | Y | Y | - | Y |
|  | 23c | Discuss any limitations of the review processes used. | Y | PY | Y | Y |
|  | 23d | Discuss implications of the results for practice, policy, and future research. | Y | Y | - | Y |
| **Other information** | | | | | | |
| Registration and  protocol | 24a | Provide registration information for the review, including register name and registration number, or state that the review was not registered. | Y | Y | - | Y |
|  | 24b | Indicate where the review protocol can be accessed, or state that a protocol was not prepared. | Y | PY | Y | Y |
|  | 24c | Describe and explain any amendments to information provided at registration or in the protocol. | Y | PY | PY | PY |
| Support | 25 | Describe sources of financial or non-financial support for the review, and the role of the funders or sponsors in the review. | Y | Y | - | Y |
| Competing interests | 26 | Declare any competing interests of review authors. | Y | Y | - | Y |
| Availability of data,  code, and other  materials | 27 | Report which of the following are publicly available and where they can be found: template data collection forms; data extracted from included studies; data used for all analyses; analytic code; any other materials used in the review. | PY | PY | - | PY |

PRISMA 2020

Y: Yes; N: No; PY: Partial Yes.

A:The conclusions of researcher Ph.D.Yongxiu Liu.

B:The conclusions of researcher Ph.D. Yuguo Li..

C:In case of a difference of opinions, it shall be adjudicated by Professor Lei Gao.

D:Conclusive conclusion.

Study 28：郝宏霞.维生素D在缓解期哮喘患者治疗中的有效性和安全性meta分析[D].山西医科大学,2018.

| Section and topic | Item # | Checklist item | A | B | C | D |
| --- | --- | --- | --- | --- | --- | --- |
| **Title** | | | | | | |
| Title | 1 | Identify the report as a systematic review. | Y | Y | - | Y |
| **Abstract** | | | | | | |
| Abstract | 2 | See the PRISMA 2020 for Abstracts checklist (table 2). | PY | PY | - | PY |
| **Introduction** | | | | | | |
| Rationale | 3 | Describe the rationale for the review in the context of existing knowledge | Y | Y | - | Y |
| Objectives | 4 | Provide an explicit statement of the objective(s) or question(s) the review addresses. | Y | Y | - | Y |
| **Methods** | | | | | | |
| Eligibility criteria | 5 | Specify the inclusion and exclusion criteria for the review and how studies were grouped for the syntheses. | PY | Y | Y | Y |
| Information sources | 6 | Specify all databases, registers, websites, organisations, reference lists and other sources searched or consulted to identify studies. Specify the date when each source was last searched or consulted. | Y | PY | Y | Y |
| Search strategy | 7 | Present the full search strategies for all databases, registers and websites, including any filters and limits used | N | N | - | N |
| Selection process | 8 | Specify the methods used to decide whether a study met the inclusion criteria of the review, including how many reviewers screened each record and each report retrieved, whether they worked independently, and if applicable, details of automation tools  used in the process. | Y | PY | Y | Y |
| Data collection  process | 9 | Specify the methods used to collect data from reports, including how many reviewers collected data from each report, whether they worked independently, any processes for obtaining or confirming data from study investigators, and if applicable, details of automation tools used in the process. | Y | PY | Y | Y |
| Data items | 10a | List and define all outcomes for which data were sought. Specify whether all results that were compatible with each outcome domain in each study were sought (e.g. for all measures, time points, analyses), and if not, the methods used to decide which  results to collect. | Y | Y | - | Y |
|  | 10b | List and define all other variables for which data were sought (e.g. participant and intervention characteristics, funding sources). Describe any assumptions made about any missing or unclear information. | PY | PY | - | PY |
| Study risk of bias  assessment | 11 | Specify the methods used to assess risk of bias in the included studies, including details of the tool(s) used, how many reviewers assessed each study and whether they worked independently, and if applicable, details of automation tools used in the process. | Y | PY | Y | Y |
| Effect measures | 12 | Specify for each outcome the effect measure(s) (e.g. risk ratio, mean difference) used in the synthesis or presentation of results. | Y | Y | - | Y |
| Synthesis methods | 13a | Describe the processes used to decide which studies were eligible for each synthesis (e.g. tabulating the study intervention characteristics and comparing against the planned groups for each synthesis (item #5)). | N | Y | Y | Y |
|  | 13b | Describe any methods required to prepare the data for presentation or synthesis, such as handling of missing summary statistics, or data conversions. | PY | Y | PY | PY |
|  | 13c | Describe any methods used to tabulate or visually display results of individual studies and syntheses. | Y | Y | - | Y |
|  | 13d | Describe any methods used to synthesise results and provide a rationale for the choice(s). If meta-analysis was performed, describe the model(s), method(s) to identify the presence and extent of statistical heterogeneity, and software package(s) used. | Y | PY | Y | Y |
|  | 13e | Describe any methods used to explore possible causes of heterogeneity among study results (e.g. subgroup analysis, meta regression). | PY | N | Y | Y |
|  | 13f | Describe any sensitivity analyses conducted to assess robustness of the synthesised results. | N | N | - | N |
| Reporting bias  assessment | 14 | Describe any methods used to assess risk of bias due to missing results in a synthesis (arising from reporting biases). | Y | PY | Y | Y |
| Certainty assessment | 15 | Describe any methods used to assess certainty (or confidence) in the body of evidence for an outcome. | N | N | - | N |
| **Results** | | | | | | |
| Study selection | 16a | Describe the results of the search and selection process, from the number of records identified in the search to the number of studies included in the review, ideally using a flow diagram (see fig 1). | Y | Y | - | Y |
|  | 16b | Cite studies that might appear to meet the inclusion criteria, but which were excluded, and explain why they were excluded. | PY | N | PY | PY |
| Study characteristics | 17 | Cite each included study and present its characteristics. | Y | Y | - | Y |
| Risk of bias in studies | 18 | Present assessments of risk of bias for each included study. | Y | PY | Y | Y |
| Results of individual  studies | 19 | For all outcomes, present, for each study: (a) summary statistics for each group (where appropriate) and (b) an effect estimate and its precision (e.g. confidence/credible interval), ideally using structured tables or plots. | Y | Y | - | Y |
| Results of syntheses | 20a | For each synthesis, briefly summarise the characteristics and risk of bias among contributing studies. | Y | PY | PY | PY |
|  | 20b | Present results of all statistical syntheses conducted. If meta-analysis was done, present for each the summary estimate and its precision (e.g. confidence/credible interval) and measures of statistical heterogeneity. If comparing groups, describe the direction of the effect. | Y | Y | - | Y |
|  | 20c | Present results of all investigations of possible causes of heterogeneity among study results. | N | PY | Y | Y |
|  | 20d | Present results of all sensitivity analyses conducted to assess the robustness of the synthesised results. | N | N | - | N |
| Reporting biases | 21 | Present assessments of risk of bias due to missing results (arising from reporting biases) for each synthesis assessed. | Y | PY | Y | Y |
| Certainty of evidence | 22 | Present assessments of certainty (or confidence) in the body of evidence for each outcome assessed. | N | N | - | N |
| **Discussion** | | | | | | |
| Discussion | 23a | Provide a general interpretation of the results in the context of other evidence. | Y | Y | - | Y |
|  | 23b | Discuss any limitations of the evidence included in the review. | Y | Y | - | Y |
|  | 23c | Discuss any limitations of the review processes used. | Y | PY | Y | Y |
|  | 23d | Discuss implications of the results for practice, policy, and future research. | Y | Y | - | Y |
| **Other information** | | | | | | |
| Registration and  protocol | 24a | Provide registration information for the review, including register name and registration number, or state that the review was not registered. | N | N | - | N |
|  | 24b | Indicate where the review protocol can be accessed, or state that a protocol was not prepared. | N | N | - | N |
|  | 24c | Describe and explain any amendments to information provided at registration or in the protocol. | Y | N | N | N |
| Support | 25 | Describe sources of financial or non-financial support for the review, and the role of the funders or sponsors in the review. | N | PY | N | N |
| Competing interests | 26 | Declare any competing interests of review authors. | Y | N | N | N |
| Availability of data,  code, and other  materials | 27 | Report which of the following are publicly available and where they can be found: template data collection forms; data extracted from included studies; data used for all analyses; analytic code; any other materials used in the review. | N | PY | N | N |

PRISMA 2020

Y: Yes; N: No; PY: Partial Yes.

A:The conclusions of researcher Ph.D.Yongxiu Liu.

B:The conclusions of researcher Ph.D. Yuguo Li..

C:In case of a difference of opinions, it shall be adjudicated by Professor Lei Gao.

D:Conclusive conclusion.

Study 29：田超,史强,赵紫楠,等.维生素D补充剂对儿童支气管哮喘获益相关性的系统评价和meta分析[J].临床药物治疗杂志,2018,16(04):66-70.

| Section and topic | Item # | Checklist item | A | B | C | D |
| --- | --- | --- | --- | --- | --- | --- |
| **Title** | | | | | | |
| Title | 1 | Identify the report as a systematic review. | Y | Y | - | Y |
| **Abstract** | | | | | | |
| Abstract | 2 | See the PRISMA 2020 for Abstracts checklist (table 2). | PY | PY | - | PY |
| **Introduction** | | | | | | |
| Rationale | 3 | Describe the rationale for the review in the context of existing knowledge | Y | Y | - | Y |
| Objectives | 4 | Provide an explicit statement of the objective(s) or question(s) the review addresses. | Y | Y | - | Y |
| **Methods** | | | | | | |
| Eligibility criteria | 5 | Specify the inclusion and exclusion criteria for the review and how studies were grouped for the syntheses. | PY | Y | Y | Y |
| Information sources | 6 | Specify all databases, registers, websites, organisations, reference lists and other sources searched or consulted to identify studies. Specify the date when each source was last searched or consulted. | Y | PY | Y | Y |
| Search strategy | 7 | Present the full search strategies for all databases, registers and websites, including any filters and limits used | N | N | - | N |
| Selection process | 8 | Specify the methods used to decide whether a study met the inclusion criteria of the review, including how many reviewers screened each record and each report retrieved, whether they worked independently, and if applicable, details of automation tools  used in the process. | Y | PY | Y | Y |
| Data collection  process | 9 | Specify the methods used to collect data from reports, including how many reviewers collected data from each report, whether they worked independently, any processes for obtaining or confirming data from study investigators, and if applicable, details of automation tools used in the process. | Y | PY | Y | Y |
| Data items | 10a | List and define all outcomes for which data were sought. Specify whether all results that were compatible with each outcome domain in each study were sought (e.g. for all measures, time points, analyses), and if not, the methods used to decide which  results to collect. | Y | Y | - | Y |
|  | 10b | List and define all other variables for which data were sought (e.g. participant and intervention characteristics, funding sources). Describe any assumptions made about any missing or unclear information. | PY | PY | - | PY |
| Study risk of bias  assessment | 11 | Specify the methods used to assess risk of bias in the included studies, including details of the tool(s) used, how many reviewers assessed each study and whether they worked independently, and if applicable, details of automation tools used in the process. | Y | PY | Y | Y |
| Effect measures | 12 | Specify for each outcome the effect measure(s) (e.g. risk ratio, mean difference) used in the synthesis or presentation of results. | Y | Y | - | Y |
| Synthesis methods | 13a | Describe the processes used to decide which studies were eligible for each synthesis (e.g. tabulating the study intervention characteristics and comparing against the planned groups for each synthesis (item #5)). | N | Y | PY | Y |
|  | 13b | Describe any methods required to prepare the data for presentation or synthesis, such as handling of missing summary statistics, or data conversions. | N | Y | N | N |
|  | 13c | Describe any methods used to tabulate or visually display results of individual studies and syntheses. | Y | Y | - | Y |
|  | 13d | Describe any methods used to synthesise results and provide a rationale for the choice(s). If meta-analysis was performed, describe the model(s), method(s) to identify the presence and extent of statistical heterogeneity, and software package(s) used. | Y | PY | N | N |
|  | 13e | Describe any methods used to explore possible causes of heterogeneity among study results (e.g. subgroup analysis, meta regression). | N | N | - | N |
|  | 13f | Describe any sensitivity analyses conducted to assess robustness of the synthesised results. | N | N | - | N |
| Reporting bias  assessment | 14 | Describe any methods used to assess risk of bias due to missing results in a synthesis (arising from reporting biases). | N | PY | N | N |
| Certainty assessment | 15 | Describe any methods used to assess certainty (or confidence) in the body of evidence for an outcome. | N | N | - | N |
| **Results** | | | | | | |
| Study selection | 16a | Describe the results of the search and selection process, from the number of records identified in the search to the number of studies included in the review, ideally using a flow diagram (see fig 1). | N | Y | N | N |
|  | 16b | Cite studies that might appear to meet the inclusion criteria, but which were excluded, and explain why they were excluded. | N | PY | N | N |
| Study characteristics | 17 | Cite each included study and present its characteristics. | Y | Y | - | Y |
| Risk of bias in studies | 18 | Present assessments of risk of bias for each included study. | Y | Y | - | Y |
| Results of individual  studies | 19 | For all outcomes, present, for each study: (a) summary statistics for each group (where appropriate) and (b) an effect estimate and its precision (e.g. confidence/credible interval), ideally using structured tables or plots. | Y | Y | - | Y |
| Results of syntheses | 20a | For each synthesis, briefly summarise the characteristics and risk of bias among contributing studies. | Y | Y | - | Y |
|  | 20b | Present results of all statistical syntheses conducted. If meta-analysis was done, present for each the summary estimate and its precision (e.g. confidence/credible interval) and measures of statistical heterogeneity. If comparing groups, describe the direction of the effect. | Y | Y | - | Y |
|  | 20c | Present results of all investigations of possible causes of heterogeneity among study results. | N | PY | PY | PY |
|  | 20d | Present results of all sensitivity analyses conducted to assess the robustness of the synthesised results. | N | N | - | N |
| Reporting biases | 21 | Present assessments of risk of bias due to missing results (arising from reporting biases) for each synthesis assessed. | N | PY | N | N |
| Certainty of evidence | 22 | Present assessments of certainty (or confidence) in the body of evidence for each outcome assessed. | N | N | - | N |
| **Discussion** | | | | | | |
| Discussion | 23a | Provide a general interpretation of the results in the context of other evidence. | Y | Y | - | Y |
|  | 23b | Discuss any limitations of the evidence included in the review. | Y | Y | - | Y |
|  | 23c | Discuss any limitations of the review processes used. | Y | PY | PY | PY |
|  | 23d | Discuss implications of the results for practice, policy, and future research. | Y | Y | - | Y |
| **Other information** | | | | | | |
| Registration and  protocol | 24a | Provide registration information for the review, including register name and registration number, or state that the review was not registered. | N | N | - | N |
|  | 24b | Indicate where the review protocol can be accessed, or state that a protocol was not prepared. | N | N | - | N |
|  | 24c | Describe and explain any amendments to information provided at registration or in the protocol. | Y | N | N | N |
| Support | 25 | Describe sources of financial or non-financial support for the review, and the role of the funders or sponsors in the review. | N | N | - | N |
| Competing interests | 26 | Declare any competing interests of review authors. | Y | PY | N | N |
| Availability of data,  code, and other  materials | 27 | Report which of the following are publicly available and where they can be found: template data collection forms; data extracted from included studies; data used for all analyses; analytic code; any other materials used in the review. | N | PY | N | N |

PRISMA 2020

Y: Yes; N: No; PY: Partial Yes.

A:The conclusions of researcher Ph.D.Yongxiu Liu.

B:The conclusions of researcher Ph.D. Yuguo Li..

C:In case of a difference of opinions, it shall be adjudicated by Professor Lei Gao.

D:Conclusive conclusion.

Study 30：郝畅.维生素D补充与儿童哮喘的系统评价及meta分析[D].重庆医科大学,2019.

| Section and topic | Item # | Checklist item | A | B | C | D |
| --- | --- | --- | --- | --- | --- | --- |
| **Title** | | | | | | |
| Title | 1 | Identify the report as a systematic review. | Y | Y | - | Y |
| **Abstract** | | | | | | |
| Abstract | 2 | See the PRISMA 2020 for Abstracts checklist (table 2). | PY | PY | - | PY |
| **Introduction** | | | | | | |
| Rationale | 3 | Describe the rationale for the review in the context of existing knowledge | Y | Y | - | Y |
| Objectives | 4 | Provide an explicit statement of the objective(s) or question(s) the review addresses. | Y | Y | - | Y |
| **Methods** | | | | | | |
| Eligibility criteria | 5 | Specify the inclusion and exclusion criteria for the review and how studies were grouped for the syntheses. | PY | Y | Y | Y |
| Information sources | 6 | Specify all databases, registers, websites, organisations, reference lists and other sources searched or consulted to identify studies. Specify the date when each source was last searched or consulted. | Y | PY | Y | Y |
| Search strategy | 7 | Present the full search strategies for all databases, registers and websites, including any filters and limits used | N | N | - | N |
| Selection process | 8 | Specify the methods used to decide whether a study met the inclusion criteria of the review, including how many reviewers screened each record and each report retrieved, whether they worked independently, and if applicable, details of automation tools  used in the process. | Y | PY | Y | Y |
| Data collection  process | 9 | Specify the methods used to collect data from reports, including how many reviewers collected data from each report, whether they worked independently, any processes for obtaining or confirming data from study investigators, and if applicable, details of automation tools used in the process. | Y | PY | Y | Y |
| Data items | 10a | List and define all outcomes for which data were sought. Specify whether all results that were compatible with each outcome domain in each study were sought (e.g. for all measures, time points, analyses), and if not, the methods used to decide which  results to collect. | Y | Y | - | Y |
|  | 10b | List and define all other variables for which data were sought (e.g. participant and intervention characteristics, funding sources). Describe any assumptions made about any missing or unclear information. | PY | PY | - | PY |
| Study risk of bias  assessment | 11 | Specify the methods used to assess risk of bias in the included studies, including details of the tool(s) used, how many reviewers assessed each study and whether they worked independently, and if applicable, details of automation tools used in the process. | Y | PY | Y | Y |
| Effect measures | 12 | Specify for each outcome the effect measure(s) (e.g. risk ratio, mean difference) used in the synthesis or presentation of results. | Y | Y | - | Y |
| Synthesis methods | 13a | Describe the processes used to decide which studies were eligible for each synthesis (e.g. tabulating the study intervention characteristics and comparing against the planned groups for each synthesis (item #5)). | Y | Y | - | Y |
|  | 13b | Describe any methods required to prepare the data for presentation or synthesis, such as handling of missing summary statistics, or data conversions. | PY | Y | N | N |
|  | 13c | Describe any methods used to tabulate or visually display results of individual studies and syntheses. | Y | Y | - | Y |
|  | 13d | Describe any methods used to synthesise results and provide a rationale for the choice(s). If meta-analysis was performed, describe the model(s), method(s) to identify the presence and extent of statistical heterogeneity, and software package(s) used. | Y | PY | Y | Y |
|  | 13e | Describe any methods used to explore possible causes of heterogeneity among study results (e.g. subgroup analysis, meta regression). | PY | Y | Y | Y |
|  | 13f | Describe any sensitivity analyses conducted to assess robustness of the synthesised results. | N | N | - | N |
| Reporting bias  assessment | 14 | Describe any methods used to assess risk of bias due to missing results in a synthesis (arising from reporting biases). | PY | PY | - | PY |
| Certainty assessment | 15 | Describe any methods used to assess certainty (or confidence) in the body of evidence for an outcome. | N | N | - | N |
| **Results** | | | | | | |
| Study selection | 16a | Describe the results of the search and selection process, from the number of records identified in the search to the number of studies included in the review, ideally using a flow diagram (see fig 1). | Y | Y | - | Y |
|  | 16b | Cite studies that might appear to meet the inclusion criteria, but which were excluded, and explain why they were excluded. | PY | PY | - | PY |
| Study characteristics | 17 | Cite each included study and present its characteristics. | Y | Y | - | Y |
| Risk of bias in studies | 18 | Present assessments of risk of bias for each included study. | Y | Y | - | Y |
| Results of individual  studies | 19 | For all outcomes, present, for each study: (a) summary statistics for each group (where appropriate) and (b) an effect estimate and its precision (e.g. confidence/credible interval), ideally using structured tables or plots. | Y | Y | - | Y |
| Results of syntheses | 20a | For each synthesis, briefly summarise the characteristics and risk of bias among contributing studies. | Y | Y | - | Y |
|  | 20b | Present results of all statistical syntheses conducted. If meta-analysis was done, present for each the summary estimate and its precision (e.g. confidence/credible interval) and measures of statistical heterogeneity. If comparing groups, describe the direction of the effect. | Y | Y | - | Y |
|  | 20c | Present results of all investigations of possible causes of heterogeneity among study results. | PY | Y | Y | Y |
|  | 20d | Present results of all sensitivity analyses conducted to assess the robustness of the synthesised results. | N | N | - | N |
| Reporting biases | 21 | Present assessments of risk of bias due to missing results (arising from reporting biases) for each synthesis assessed. | PY | PY | - | PY |
| Certainty of evidence | 22 | Present assessments of certainty (or confidence) in the body of evidence for each outcome assessed. | N | N | - | N |
| **Discussion** | | | | | | |
| Discussion | 23a | Provide a general interpretation of the results in the context of other evidence. | Y | Y | - | Y |
|  | 23b | Discuss any limitations of the evidence included in the review. | Y | Y | - | Y |
|  | 23c | Discuss any limitations of the review processes used. | Y | PY | Y | Y |
|  | 23d | Discuss implications of the results for practice, policy, and future research. | Y | Y | - | Y |
| **Other information** | | | | | | |
| Registration and  protocol | 24a | Provide registration information for the review, including register name and registration number, or state that the review was not registered. | N | N | - | N |
|  | 24b | Indicate where the review protocol can be accessed, or state that a protocol was not prepared. | N | N | - | N |
|  | 24c | Describe and explain any amendments to information provided at registration or in the protocol. | Y | N | N | N |
| Support | 25 | Describe sources of financial or non-financial support for the review, and the role of the funders or sponsors in the review. | N | N | - | N |
| Competing interests | 26 | Declare any competing interests of review authors. | Y | PY | N | N |
| Availability of data,  code, and other  materials | 27 | Report which of the following are publicly available and where they can be found: template data collection forms; data extracted from included studies; data used for all analyses; analytic code; any other materials used in the review. | N | PY | N | N |

PRISMA 2020

Y: Yes; N: No; PY: Partial Yes.

A:The conclusions of researcher Ph.D.Yongxiu Liu.

B:The conclusions of researcher Ph.D. Yuguo Li..

C:In case of a difference of opinions, it shall be adjudicated by Professor Lei Gao.

D:Conclusive conclusion.

Study 31：Wang, Mingming et al. “Association between vitamin D status and asthma control: A meta-analysis of randomized trials.”Respiratory medicine vol. 150 (2019): 85-94. doi:10.1016/j.rmed.2019.02.016.PMID: 30961957

| Section and topic | Item # | Checklist item | A | B | C | D |
| --- | --- | --- | --- | --- | --- | --- |
| **Title** | | | | | | |
| Title | 1 | Identify the report as a systematic review. | Y | PY | Y | Y |
| **Abstract** | | | | | | |
| Abstract | 2 | See the PRISMA 2020 for Abstracts checklist (table 2). | PY | PY | - | PY |
| **Introduction** | | | | | | |
| Rationale | 3 | Describe the rationale for the review in the context of existing knowledge | Y | Y | - | Y |
| Objectives | 4 | Provide an explicit statement of the objective(s) or question(s) the review addresses. | Y | Y | - | Y |
| **Methods** | | | | | | |
| Eligibility criteria | 5 | Specify the inclusion and exclusion criteria for the review and how studies were grouped for the syntheses. | Y | Y | - | Y |
| Information sources | 6 | Specify all databases, registers, websites, organisations, reference lists and other sources searched or consulted to identify studies. Specify the date when each source was last searched or consulted. | PY | PY | - | PY |
| Search strategy | 7 | Present the full search strategies for all databases, registers and websites, including any filters and limits used | PY | N | N | N |
| Selection process | 8 | Specify the methods used to decide whether a study met the inclusion criteria of the review, including how many reviewers screened each record and each report retrieved, whether they worked independently, and if applicable, details of automation tools  used in the process. | Y | PY | Y | Y |
| Data collection  process | 9 | Specify the methods used to collect data from reports, including how many reviewers collected data from each report, whether they worked independently, any processes for obtaining or confirming data from study investigators, and if applicable, details of automation tools used in the process. | Y | PY | Y | Y |
| Data items | 10a | List and define all outcomes for which data were sought. Specify whether all results that were compatible with each outcome domain in each study were sought (e.g. for all measures, time points, analyses), and if not, the methods used to decide which  results to collect. | Y | PY | Y | Y |
|  | 10b | List and define all other variables for which data were sought (e.g. participant and intervention characteristics, funding sources). Describe any assumptions made about any missing or unclear information. | PY | PY | - | PY |
| Study risk of bias  assessment | 11 | Specify the methods used to assess risk of bias in the included studies, including details of the tool(s) used, how many reviewers assessed each study and whether they worked independently, and if applicable, details of automation tools used in the process. | Y | Y | - | Y |
| Effect measures | 12 | Specify for each outcome the effect measure(s) (e.g. risk ratio, mean difference) used in the synthesis or presentation of results. | Y | Y | - | Y |
| Synthesis methods | 13a | Describe the processes used to decide which studies were eligible for each synthesis (e.g. tabulating the study intervention characteristics and comparing against the planned groups for each synthesis (item #5)). | Y | PY | Y | Y |
|  | 13b | Describe any methods required to prepare the data for presentation or synthesis, such as handling of missing summary statistics, or data conversions. | PY | N | Y | Y |
|  | 13c | Describe any methods used to tabulate or visually display results of individual studies and syntheses. | Y | PY | Y | Y |
|  | 13d | Describe any methods used to synthesise results and provide a rationale for the choice(s). If meta-analysis was performed, describe the model(s), method(s) to identify the presence and extent of statistical heterogeneity, and software package(s) used. | Y | Y | - | Y |
|  | 13e | Describe any methods used to explore possible causes of heterogeneity among study results (e.g. subgroup analysis, meta regression). | Y | Y | - | Y |
|  | 13f | Describe any sensitivity analyses conducted to assess robustness of the synthesised results. | PY | Y | Y | Y |
| Reporting bias  assessment | 14 | Describe any methods used to assess risk of bias due to missing results in a synthesis (arising from reporting biases). | Y | N | PY | PY |
| Certainty assessment | 15 | Describe any methods used to assess certainty (or confidence) in the body of evidence for an outcome. | Y | Y | - | Y |
| **Results** | | | | | | |
| Study selection | 16a | Describe the results of the search and selection process, from the number of records identified in the search to the number of studies included in the review, ideally using a flow diagram (see fig 1). | Y | Y | - | Y |
|  | 16b | Cite studies that might appear to meet the inclusion criteria, but which were excluded, and explain why they were excluded. | N | N | - | N |
| Study characteristics | 17 | Cite each included study and present its characteristics. | Y | Y | - | Y |
| Risk of bias in studies | 18 | Present assessments of risk of bias for each included study. | Y | Y | - | Y |
| Results of individual  studies | 19 | For all outcomes, present, for each study: (a) summary statistics for each group (where appropriate) and (b) an effect estimate and its precision (e.g. confidence/credible interval), ideally using structured tables or plots. | PY | PY | - | PY |
| Results of syntheses | 20a | For each synthesis, briefly summarise the characteristics and risk of bias among contributing studies. | Y | PY | Y | Y |
|  | 20b | Present results of all statistical syntheses conducted. If meta-analysis was done, present for each the summary estimate and its precision (e.g. confidence/credible interval) and measures of statistical heterogeneity. If comparing groups, describe the direction of the effect. | Y | Y | - | Y |
|  | 20c | Present results of all investigations of possible causes of heterogeneity among study results. | Y | Y | - | Y |
|  | 20d | Present results of all sensitivity analyses conducted to assess the robustness of the synthesised results. | Y | Y | - | Y |
| Reporting biases | 21 | Present assessments of risk of bias due to missing results (arising from reporting biases) for each synthesis assessed. | N | N | - | N |
| Certainty of evidence | 22 | Present assessments of certainty (or confidence) in the body of evidence for each outcome assessed. | Y | Y | - | Y |
| **Discussion** | | | | | | |
| Discussion | 23a | Provide a general interpretation of the results in the context of other evidence. | Y | Y | - | Y |
|  | 23b | Discuss any limitations of the evidence included in the review. | Y | Y | - | Y |
|  | 23c | Discuss any limitations of the review processes used. | Y | PY | Y | Y |
|  | 23d | Discuss implications of the results for practice, policy, and future research. | Y | Y | - | Y |
| **Other information** | | | | | | |
| Registration and  protocol | 24a | Provide registration information for the review, including register name and registration number, or state that the review was not registered. | Y | Y | - | Y |
|  | 24b | Indicate where the review protocol can be accessed, or state that a protocol was not prepared. | N | N | - | N |
|  | 24c | Describe and explain any amendments to information provided at registration or in the protocol. | Y | N | N | N |
| Support | 25 | Describe sources of financial or non-financial support for the review, and the role of the funders or sponsors in the review. | Y | Y | - | Y |
| Competing interests | 26 | Declare any competing interests of review authors. | Y | Y | - | Y |
| Availability of data,  code, and other  materials | 27 | Report which of the following are publicly available and where they can be found: template data collection forms; data extracted from included studies; data used for all analyses; analytic code; any other materials used in the review. | N | N | - | N |

PRISMA 2020

Y: Yes; N: No; PY: Partial Yes.

A:The conclusions of researcher Ph.D.Yongxiu Liu.

B:The conclusions of researcher Ph.D. Yuguo Li..

C:In case of a difference of opinions, it shall be adjudicated by Professor Lei Gao.

D:Conclusive conclusion.

Study 32：Chen, Ziyu et al. “Vitamin D can safely reduce asthma exacerbations among corticosteroid-using children and adults with asthma: a systematic review and meta-analysis of randomized controlled trials.” Nutrition research (New York, N.Y.) vol. 92 (2021): 49-61. doi:10.1016/j.nutres.2021.05.010.PMID: 34274554

| Section and topic | Item # | Checklist item | A | B | C | D |
| --- | --- | --- | --- | --- | --- | --- |
| **Title** | | | | | | |
| Title | 1 | Identify the report as a systematic review. | Y | PY | Y | Y |
| **Abstract** | | | | | | |
| Abstract | 2 | See the PRISMA 2020 for Abstracts checklist (table 2). | PY | PY | - | PY |
| **Introduction** | | | | | | |
| Rationale | 3 | Describe the rationale for the review in the context of existing knowledge | Y | Y | - | Y |
| Objectives | 4 | Provide an explicit statement of the objective(s) or question(s) the review addresses. | Y | Y | - | Y |
| **Methods** | | | | | | |
| Eligibility criteria | 5 | Specify the inclusion and exclusion criteria for the review and how studies were grouped for the syntheses. | Y | Y | - | Y |
| Information sources | 6 | Specify all databases, registers, websites, organisations, reference lists and other sources searched or consulted to identify studies. Specify the date when each source was last searched or consulted. | Y | PY | Y | Y |
| Search strategy | 7 | Present the full search strategies for all databases, registers and websites, including any filters and limits used | PY | N | Y | Y |
| Selection process | 8 | Specify the methods used to decide whether a study met the inclusion criteria of the review, including how many reviewers screened each record and each report retrieved, whether they worked independently, and if applicable, details of automation tools  used in the process. | Y | Y | - | Y |
| Data collection  process | 9 | Specify the methods used to collect data from reports, including how many reviewers collected data from each report, whether they worked independently, any processes for obtaining or confirming data from study investigators, and if applicable, details of automation tools used in the process. | Y | PY | Y | Y |
| Data items | 10a | List and define all outcomes for which data were sought. Specify whether all results that were compatible with each outcome domain in each study were sought (e.g. for all measures, time points, analyses), and if not, the methods used to decide which  results to collect. | Y | PY | Y | Y |
|  | 10b | List and define all other variables for which data were sought (e.g. participant and intervention characteristics, funding sources). Describe any assumptions made about any missing or unclear information. | PY | PY | - | PY |
| Study risk of bias  assessment | 11 | Specify the methods used to assess risk of bias in the included studies, including details of the tool(s) used, how many reviewers assessed each study and whether they worked independently, and if applicable, details of automation tools used in the process. | Y | Y | - | Y |
| Effect measures | 12 | Specify for each outcome the effect measure(s) (e.g. risk ratio, mean difference) used in the synthesis or presentation of results. | Y | Y | - | Y |
| Synthesis methods | 13a | Describe the processes used to decide which studies were eligible for each synthesis (e.g. tabulating the study intervention characteristics and comparing against the planned groups for each synthesis (item #5)). | Y | PY | Y | Y |
|  | 13b | Describe any methods required to prepare the data for presentation or synthesis, such as handling of missing summary statistics, or data conversions. | PY | N | PY | PY |
|  | 13c | Describe any methods used to tabulate or visually display results of individual studies and syntheses. | Y | Y | - | Y |
|  | 13d | Describe any methods used to synthesise results and provide a rationale for the choice(s). If meta-analysis was performed, describe the model(s), method(s) to identify the presence and extent of statistical heterogeneity, and software package(s) used. | Y | Y | - | Y |
|  | 13e | Describe any methods used to explore possible causes of heterogeneity among study results (e.g. subgroup analysis, meta regression). | Y | Y | - | Y |
|  | 13f | Describe any sensitivity analyses conducted to assess robustness of the synthesised results. | PY | Y | Y | Y |
| Reporting bias  assessment | 14 | Describe any methods used to assess risk of bias due to missing results in a synthesis (arising from reporting biases). | Y | PY | Y | Y |
| Certainty assessment | 15 | Describe any methods used to assess certainty (or confidence) in the body of evidence for an outcome. | N | N | - | N |
| **Results** | | | | | | |
| Study selection | 16a | Describe the results of the search and selection process, from the number of records identified in the search to the number of studies included in the review, ideally using a flow diagram (see fig 1). | Y | Y | - | Y |
|  | 16b | Cite studies that might appear to meet the inclusion criteria, but which were excluded, and explain why they were excluded. | N | N | - | N |
| Study characteristics | 17 | Cite each included study and present its characteristics. | Y | Y | - | Y |
| Risk of bias in studies | 18 | Present assessments of risk of bias for each included study. | Y | Y | - | Y |
| Results of individual  studies | 19 | For all outcomes, present, for each study: (a) summary statistics for each group (where appropriate) and (b) an effect estimate and its precision (e.g. confidence/credible interval), ideally using structured tables or plots. | PY | Y | Y | Y |
| Results of syntheses | 20a | For each synthesis, briefly summarise the characteristics and risk of bias among contributing studies. | Y | PY | Y | Y |
|  | 20b | Present results of all statistical syntheses conducted. If meta-analysis was done, present for each the summary estimate and its precision (e.g. confidence/credible interval) and measures of statistical heterogeneity. If comparing groups, describe the direction of the effect. | Y | Y | - | Y |
|  | 20c | Present results of all investigations of possible causes of heterogeneity among study results. | Y | Y | - | Y |
|  | 20d | Present results of all sensitivity analyses conducted to assess the robustness of the synthesised results. | PY | Y | Y | Y |
| Reporting biases | 21 | Present assessments of risk of bias due to missing results (arising from reporting biases) for each synthesis assessed. | Y | PY | Y | Y |
| Certainty of evidence | 22 | Present assessments of certainty (or confidence) in the body of evidence for each outcome assessed. | N | N | - | N |
| **Discussion** | | | | | | |
| Discussion | 23a | Provide a general interpretation of the results in the context of other evidence. | Y | Y | - | Y |
|  | 23b | Discuss any limitations of the evidence included in the review. | Y | Y | - | Y |
|  | 23c | Discuss any limitations of the review processes used. | PY | PY | - | PY |
|  | 23d | Discuss implications of the results for practice, policy, and future research. | Y | Y | - | Y |
| **Other information** | | | | | | |
| Registration and  protocol | 24a | Provide registration information for the review, including register name and registration number, or state that the review was not registered. | N | N | - | N |
|  | 24b | Indicate where the review protocol can be accessed, or state that a protocol was not prepared. | N | N | - | N |
|  | 24c | Describe and explain any amendments to information provided at registration or in the protocol. | N | N | - | N |
| Support | 25 | Describe sources of financial or non-financial support for the review, and the role of the funders or sponsors in the review. | Y | Y | - | Y |
| Competing interests | 26 | Declare any competing interests of review authors. | Y | Y | - | Y |
| Availability of data,  code, and other  materials | 27 | Report which of the following are publicly available and where they can be found: template data collection forms; data extracted from included studies; data used for all analyses; analytic code; any other materials used in the review. | N | N | - | N |

PRISMA 2020

Y: Yes; N: No; PY: Partial Yes.

A:The conclusions of researcher Ph.D.Yongxiu Liu.

B:The conclusions of researcher Ph.D. Yuguo Li..

C:In case of a difference of opinions, it shall be adjudicated by Professor Lei Gao.

D:Conclusive conclusion.

Study 33：Hao, Meiqi et al. “The Effect of Vitamin D Supplementation in Children With Asthma: A Meta-Analysis.”Frontiers in pediatrics vol. 10 840617. 29 Jun. 2022, doi:10.3389/fped.2022.840617.PMID: 35844729 PMCID: PMC9277022

| Section and topic | Item # | Checklist item | A | B | C | D |
| --- | --- | --- | --- | --- | --- | --- |
| **Title** | | | | | | |
| Title | 1 | Identify the report as a systematic review. | Y | Y | - | Y |
| **Abstract** | | | | | | |
| Abstract | 2 | See the PRISMA 2020 for Abstracts checklist (table 2). | PY | PY | - | PY |
| **Introduction** | | | | | | |
| Rationale | 3 | Describe the rationale for the review in the context of existing knowledge | Y | Y | - | Y |
| Objectives | 4 | Provide an explicit statement of the objective(s) or question(s) the review addresses. | Y | Y | - | Y |
| **Methods** | | | | | | |
| Eligibility criteria | 5 | Specify the inclusion and exclusion criteria for the review and how studies were grouped for the syntheses. | Y | Y | - | Y |
| Information sources | 6 | Specify all databases, registers, websites, organisations, reference lists and other sources searched or consulted to identify studies. Specify the date when each source was last searched or consulted. | Y | PY | Y | Y |
| Search strategy | 7 | Present the full search strategies for all databases, registers and websites, including any filters and limits used | PY | N | PY | PY |
| Selection process | 8 | Specify the methods used to decide whether a study met the inclusion criteria of the review, including how many reviewers screened each record and each report retrieved, whether they worked independently, and if applicable, details of automation tools  used in the process. | Y | Y | - | Y |
| Data collection  process | 9 | Specify the methods used to collect data from reports, including how many reviewers collected data from each report, whether they worked independently, any processes for obtaining or confirming data from study investigators, and if applicable, details of automation tools used in the process. | Y | PY | Y | Y |
| Data items | 10a | List and define all outcomes for which data were sought. Specify whether all results that were compatible with each outcome domain in each study were sought (e.g. for all measures, time points, analyses), and if not, the methods used to decide which  results to collect. | Y | PY | Y | Y |
|  | 10b | List and define all other variables for which data were sought (e.g. participant and intervention characteristics, funding sources). Describe any assumptions made about any missing or unclear information. | PY | PY | - | PY |
| Study risk of bias  assessment | 11 | Specify the methods used to assess risk of bias in the included studies, including details of the tool(s) used, how many reviewers assessed each study and whether they worked independently, and if applicable, details of automation tools used in the process. | Y | Y | - | Y |
| Effect measures | 12 | Specify for each outcome the effect measure(s) (e.g. risk ratio, mean difference) used in the synthesis or presentation of results. | Y | Y | - | Y |
| Synthesis methods | 13a | Describe the processes used to decide which studies were eligible for each synthesis (e.g. tabulating the study intervention characteristics and comparing against the planned groups for each synthesis (item #5)). | Y | PY | Y | Y |
|  | 13b | Describe any methods required to prepare the data for presentation or synthesis, such as handling of missing summary statistics, or data conversions. | PY | N | PY | PY |
|  | 13c | Describe any methods used to tabulate or visually display results of individual studies and syntheses. | Y | Y | - | Y |
|  | 13d | Describe any methods used to synthesise results and provide a rationale for the choice(s). If meta-analysis was performed, describe the model(s), method(s) to identify the presence and extent of statistical heterogeneity, and software package(s) used. | Y | Y | - | Y |
|  | 13e | Describe any methods used to explore possible causes of heterogeneity among study results (e.g. subgroup analysis, meta regression). | PY | Y | Y | Y |
|  | 13f | Describe any sensitivity analyses conducted to assess robustness of the synthesised results. | Y | Y | - | Y |
| Reporting bias  assessment | 14 | Describe any methods used to assess risk of bias due to missing results in a synthesis (arising from reporting biases). | Y | Y | - | Y |
| Certainty assessment | 15 | Describe any methods used to assess certainty (or confidence) in the body of evidence for an outcome. | Y | Y | - | Y |
| **Results** | | | | | | |
| Study selection | 16a | Describe the results of the search and selection process, from the number of records identified in the search to the number of studies included in the review, ideally using a flow diagram (see fig 1). | Y | Y | - | Y |
|  | 16b | Cite studies that might appear to meet the inclusion criteria, but which were excluded, and explain why they were excluded. | N | N | - | N |
| Study characteristics | 17 | Cite each included study and present its characteristics. | Y | Y | - | Y |
| Risk of bias in studies | 18 | Present assessments of risk of bias for each included study. | Y | Y | - | Y |
| Results of individual  studies | 19 | For all outcomes, present, for each study: (a) summary statistics for each group (where appropriate) and (b) an effect estimate and its precision (e.g. confidence/credible interval), ideally using structured tables or plots. | PY | Y | Y | Y |
| Results of syntheses | 20a | For each synthesis, briefly summarise the characteristics and risk of bias among contributing studies. | Y | PY | Y | Y |
|  | 20b | Present results of all statistical syntheses conducted. If meta-analysis was done, present for each the summary estimate and its precision (e.g. confidence/credible interval) and measures of statistical heterogeneity. If comparing groups, describe the direction of the effect. | Y | Y | - | Y |
|  | 20c | Present results of all investigations of possible causes of heterogeneity among study results. | PY | Y | Y | Y |
|  | 20d | Present results of all sensitivity analyses conducted to assess the robustness of the synthesised results. | Y | PY | Y | Y |
| Reporting biases | 21 | Present assessments of risk of bias due to missing results (arising from reporting biases) for each synthesis assessed. | Y | Y | - | Y |
| Certainty of evidence | 22 | Present assessments of certainty (or confidence) in the body of evidence for each outcome assessed. | Y | Y | - | Y |
| **Discussion** | | | | | | |
| Discussion | 23a | Provide a general interpretation of the results in the context of other evidence. | Y | Y | - | Y |
|  | 23b | Discuss any limitations of the evidence included in the review. | Y | Y | - | Y |
|  | 23c | Discuss any limitations of the review processes used. | PY | PY | - | PY |
|  | 23d | Discuss implications of the results for practice, policy, and future research. | Y | Y | - | Y |
| **Other information** | | | | | | |
| Registration and  protocol | 24a | Provide registration information for the review, including register name and registration number, or state that the review was not registered. | Y | Y | - | Y |
|  | 24b | Indicate where the review protocol can be accessed, or state that a protocol was not prepared. | PY | N | PY | PY |
|  | 24c | Describe and explain any amendments to information provided at registration or in the protocol. | Y | N | N | N |
| Support | 25 | Describe sources of financial or non-financial support for the review, and the role of the funders or sponsors in the review. | Y | Y | - | Y |
| Competing interests | 26 | Declare any competing interests of review authors. | Y | Y | - | Y |
| Availability of data,  code, and other  materials | 27 | Report which of the following are publicly available and where they can be found: template data collection forms; data extracted from included studies; data used for all analyses; analytic code; any other materials used in the review. | PY | PY | - | PY |

PRISMA 2020

Y: Yes; N: No; PY: Partial Yes.

A:The conclusions of researcher Ph.D.Yongxiu Liu.

B:The conclusions of researcher Ph.D. Yuguo Li.

C:In case of a difference of opinions, it shall be adjudicated by Professor Lei Gao.

D:Conclusive conclusion.

Study 34：Kumar, Jogender et al. “Vitamin D supplementation in childhood asthma: a systematic review and meta-analysis of randomised controlled trials.” ERJ open research vol. 8,1 00662-2021. 7 Feb. 2021, doi:10.1183/23120541.00662-2021.PMID: 35141325 PMCID: PMC8819253

| Section and topic | Item # | Checklist item | A | B | C | D |
| --- | --- | --- | --- | --- | --- | --- |
| **Title** | | | | | | |
| Title | 1 | Identify the report as a systematic review. | Y | Y | - | Y |
| **Abstract** | | | | | | |
| Abstract | 2 | See the PRISMA 2020 for Abstracts checklist (table 2). | PY | PY | - | PY |
| **Introduction** | | | | | | |
| Rationale | 3 | Describe the rationale for the review in the context of existing knowledge | Y | Y | - | Y |
| Objectives | 4 | Provide an explicit statement of the objective(s) or question(s) the review addresses. | Y | Y | - | Y |
| **Methods** | | | | | | |
| Eligibility criteria | 5 | Specify the inclusion and exclusion criteria for the review and how studies were grouped for the syntheses. | Y | Y | - | Y |
| Information sources | 6 | Specify all databases, registers, websites, organisations, reference lists and other sources searched or consulted to identify studies. Specify the date when each source was last searched or consulted. | Y | PY | Y | Y |
| Search strategy | 7 | Present the full search strategies for all databases, registers and websites, including any filters and limits used | PY | PY | - | PY |
| Selection process | 8 | Specify the methods used to decide whether a study met the inclusion criteria of the review, including how many reviewers screened each record and each report retrieved, whether they worked independently, and if applicable, details of automation tools  used in the process. | Y | Y | - | Y |
| Data collection  process | 9 | Specify the methods used to collect data from reports, including how many reviewers collected data from each report, whether they worked independently, any processes for obtaining or confirming data from study investigators, and if applicable, details of automation tools used in the process. | Y | Y | - | Y |
| Data items | 10a | List and define all outcomes for which data were sought. Specify whether all results that were compatible with each outcome domain in each study were sought (e.g. for all measures, time points, analyses), and if not, the methods used to decide which  results to collect. | Y | Y | - | Y |
|  | 10b | List and define all other variables for which data were sought (e.g. participant and intervention characteristics, funding sources). Describe any assumptions made about any missing or unclear information. | PY | PY | - | PY |
| Study risk of bias  assessment | 11 | Specify the methods used to assess risk of bias in the included studies, including details of the tool(s) used, how many reviewers assessed each study and whether they worked independently, and if applicable, details of automation tools used in the process. | Y | Y | - | Y |
| Effect measures | 12 | Specify for each outcome the effect measure(s) (e.g. risk ratio, mean difference) used in the synthesis or presentation of results. | Y | Y | - | Y |
| Synthesis methods | 13a | Describe the processes used to decide which studies were eligible for each synthesis (e.g. tabulating the study intervention characteristics and comparing against the planned groups for each synthesis (item #5)). | Y | PY | Y | Y |
|  | 13b | Describe any methods required to prepare the data for presentation or synthesis, such as handling of missing summary statistics, or data conversions. | Y | PY | Y | Y |
|  | 13c | Describe any methods used to tabulate or visually display results of individual studies and syntheses. | Y | Y | - | Y |
|  | 13d | Describe any methods used to synthesise results and provide a rationale for the choice(s). If meta-analysis was performed, describe the model(s), method(s) to identify the presence and extent of statistical heterogeneity, and software package(s) used. | Y | Y | - | Y |
|  | 13e | Describe any methods used to explore possible causes of heterogeneity among study results (e.g. subgroup analysis, meta regression). | Y | Y | - | Y |
|  | 13f | Describe any sensitivity analyses conducted to assess robustness of the synthesised results. | PY | N | Y | Y |
| Reporting bias  assessment | 14 | Describe any methods used to assess risk of bias due to missing results in a synthesis (arising from reporting biases). | PY | Y | - | Y |
| Certainty assessment | 15 | Describe any methods used to assess certainty (or confidence) in the body of evidence for an outcome. | Y | Y | - | Y |
| **Results** | | | | | | |
| Study selection | 16a | Describe the results of the search and selection process, from the number of records identified in the search to the number of studies included in the review, ideally using a flow diagram (see fig 1). | Y | Y | - | Y |
|  | 16b | Cite studies that might appear to meet the inclusion criteria, but which were excluded, and explain why they were excluded. | N | PY | PY | PY |
| Study characteristics | 17 | Cite each included study and present its characteristics. | Y | Y | - | Y |
| Risk of bias in studies | 18 | Present assessments of risk of bias for each included study. | Y | Y | - | Y |
| Results of individual  studies | 19 | For all outcomes, present, for each study: (a) summary statistics for each group (where appropriate) and (b) an effect estimate and its precision (e.g. confidence/credible interval), ideally using structured tables or plots. | PY | Y | Y | Y |
| Results of syntheses | 20a | For each synthesis, briefly summarise the characteristics and risk of bias among contributing studies. | Y | PY | Y | Y |
|  | 20b | Present results of all statistical syntheses conducted. If meta-analysis was done, present for each the summary estimate and its precision (e.g. confidence/credible interval) and measures of statistical heterogeneity. If comparing groups, describe the direction of the effect. | Y | Y | - | Y |
|  | 20c | Present results of all investigations of possible causes of heterogeneity among study results. | Y | Y | - | Y |
|  | 20d | Present results of all sensitivity analyses conducted to assess the robustness of the synthesised results. | PY | PY | - | PY |
| Reporting biases | 21 | Present assessments of risk of bias due to missing results (arising from reporting biases) for each synthesis assessed. | PY | Y | Y | Y |
| Certainty of evidence | 22 | Present assessments of certainty (or confidence) in the body of evidence for each outcome assessed. | Y | Y | - | Y |
| **Discussion** | | | | | | |
| Discussion | 23a | Provide a general interpretation of the results in the context of other evidence. | Y | Y | - | Y |
|  | 23b | Discuss any limitations of the evidence included in the review. | Y | Y | - | Y |
|  | 23c | Discuss any limitations of the review processes used. | PY | PY | - | PY |
|  | 23d | Discuss implications of the results for practice, policy, and future research. | Y | Y | - | Y |
| **Other information** | | | | | | |
| Registration and  protocol | 24a | Provide registration information for the review, including register name and registration number, or state that the review was not registered. | Y | Y | - | Y |
|  | 24b | Indicate where the review protocol can be accessed, or state that a protocol was not prepared. | PY | PY | - | PY |
|  | 24c | Describe and explain any amendments to information provided at registration or in the protocol. | PY | N | PY | PY |
| Support | 25 | Describe sources of financial or non-financial support for the review, and the role of the funders or sponsors in the review. | N | N | - | N |
| Competing interests | 26 | Declare any competing interests of review authors. | Y | Y | - | Y |
| Availability of data,  code, and other  materials | 27 | Report which of the following are publicly available and where they can be found: template data collection forms; data extracted from included studies; data used for all analyses; analytic code; any other materials used in the review. | PY | Y | PY | PY |

PRISMA 2020

Y: Yes; N: No; PY: Partial Yes.

A:The conclusions of researcher Ph.D.Yongxiu Liu.

B:The conclusions of researcher Ph.D. Yuguo Li.

C:In case of a difference of opinions, it shall be adjudicated by Professor Lei Gao.

D:Conclusive conclusion.

Study 35：Liu, Meiqi et al. “A Meta-Analysis on Vitamin D Supplementation and Asthma Treatment.” Frontiers in nutrition vol. 9 860628. 6 Jul. 2022, doi:10.3389/fnut.2022.860628.PMID: 35873428 PMCID: PMC9300755

| Section and topic | Item # | Checklist item | A | B | C | D |
| --- | --- | --- | --- | --- | --- | --- |
| **Title** | | | | | | |
| Title | 1 | Identify the report as a systematic review. | Y | Y | - | Y |
| **Abstract** | | | | | | |
| Abstract | 2 | See the PRISMA 2020 for Abstracts checklist (table 2). | PY | PY | - | PY |
| **Introduction** | | | | | | |
| Rationale | 3 | Describe the rationale for the review in the context of existing knowledge | Y | Y | - | Y |
| Objectives | 4 | Provide an explicit statement of the objective(s) or question(s) the review addresses. | Y | Y | - | Y |
| **Methods** | | | | | | |
| Eligibility criteria | 5 | Specify the inclusion and exclusion criteria for the review and how studies were grouped for the syntheses. | Y | Y | - | Y |
| Information sources | 6 | Specify all databases, registers, websites, organisations, reference lists and other sources searched or consulted to identify studies. Specify the date when each source was last searched or consulted. | PY | PY | - | PY |
| Search strategy | 7 | Present the full search strategies for all databases, registers and websites, including any filters and limits used | PY | PY | - | PY |
| Selection process | 8 | Specify the methods used to decide whether a study met the inclusion criteria of the review, including how many reviewers screened each record and each report retrieved, whether they worked independently, and if applicable, details of automation tools  used in the process. | Y | Y | - | Y |
| Data collection  process | 9 | Specify the methods used to collect data from reports, including how many reviewers collected data from each report, whether they worked independently, any processes for obtaining or confirming data from study investigators, and if applicable, details of automation tools used in the process. | Y | PY | Y | Y |
| Data items | 10a | List and define all outcomes for which data were sought. Specify whether all results that were compatible with each outcome domain in each study were sought (e.g. for all measures, time points, analyses), and if not, the methods used to decide which  results to collect. | Y | PY | Y | Y |
|  | 10b | List and define all other variables for which data were sought (e.g. participant and intervention characteristics, funding sources). Describe any assumptions made about any missing or unclear information. | PY | PY | - | PY |
| Study risk of bias  assessment | 11 | Specify the methods used to assess risk of bias in the included studies, including details of the tool(s) used, how many reviewers assessed each study and whether they worked independently, and if applicable, details of automation tools used in the process. | Y | Y | - | Y |
| Effect measures | 12 | Specify for each outcome the effect measure(s) (e.g. risk ratio, mean difference) used in the synthesis or presentation of results. | Y | Y | - | Y |
| Synthesis methods | 13a | Describe the processes used to decide which studies were eligible for each synthesis (e.g. tabulating the study intervention characteristics and comparing against the planned groups for each synthesis (item #5)). | Y | PY | Y | Y |
|  | 13b | Describe any methods required to prepare the data for presentation or synthesis, such as handling of missing summary statistics, or data conversions. | N | N | - | N |
|  | 13c | Describe any methods used to tabulate or visually display results of individual studies and syntheses. | Y | Y | - | Y |
|  | 13d | Describe any methods used to synthesise results and provide a rationale for the choice(s). If meta-analysis was performed, describe the model(s), method(s) to identify the presence and extent of statistical heterogeneity, and software package(s) used. | Y | Y | - | Y |
|  | 13e | Describe any methods used to explore possible causes of heterogeneity among study results (e.g. subgroup analysis, meta regression). | PY | Y | Y | Y |
|  | 13f | Describe any sensitivity analyses conducted to assess robustness of the synthesised results. | Y | N | Y | Y |
| Reporting bias  assessment | 14 | Describe any methods used to assess risk of bias due to missing results in a synthesis (arising from reporting biases). | N | PY | PY | PY |
| Certainty assessment | 15 | Describe any methods used to assess certainty (or confidence) in the body of evidence for an outcome. | Y | Y | - | Y |
| **Results** | | | | | | |
| Study selection | 16a | Describe the results of the search and selection process, from the number of records identified in the search to the number of studies included in the review, ideally using a flow diagram (see fig 1). | Y | Y | - | Y |
|  | 16b | Cite studies that might appear to meet the inclusion criteria, but which were excluded, and explain why they were excluded. | N | N | - | N |
| Study characteristics | 17 | Cite each included study and present its characteristics. | Y | Y | - | Y |
| Risk of bias in studies | 18 | Present assessments of risk of bias for each included study. | Y | Y | - | Y |
| Results of individual  studies | 19 | For all outcomes, present, for each study: (a) summary statistics for each group (where appropriate) and (b) an effect estimate and its precision (e.g. confidence/credible interval), ideally using structured tables or plots. | PY | Y | Y | Y |
| Results of syntheses | 20a | For each synthesis, briefly summarise the characteristics and risk of bias among contributing studies. | Y | PY | Y | Y |
|  | 20b | Present results of all statistical syntheses conducted. If meta-analysis was done, present for each the summary estimate and its precision (e.g. confidence/credible interval) and measures of statistical heterogeneity. If comparing groups, describe the direction of the effect. | Y | Y | - | Y |
|  | 20c | Present results of all investigations of possible causes of heterogeneity among study results. | PY | Y | Y | Y |
|  | 20d | Present results of all sensitivity analyses conducted to assess the robustness of the synthesised results. | Y | PY | Y | Y |
| Reporting biases | 21 | Present assessments of risk of bias due to missing results (arising from reporting biases) for each synthesis assessed. | N | PY | PY | PY |
| Certainty of evidence | 22 | Present assessments of certainty (or confidence) in the body of evidence for each outcome assessed. | Y | Y | - | Y |
| **Discussion** | | | | | | |
| Discussion | 23a | Provide a general interpretation of the results in the context of other evidence. | Y | Y | - | Y |
|  | 23b | Discuss any limitations of the evidence included in the review. | Y | Y | - | Y |
|  | 23c | Discuss any limitations of the review processes used. | PY | PY | - | PY |
|  | 23d | Discuss implications of the results for practice, policy, and future research. | Y | Y | - | Y |
| **Other information** | | | | | | |
| Registration and  protocol | 24a | Provide registration information for the review, including register name and registration number, or state that the review was not registered. | Y | Y | - | Y |
|  | 24b | Indicate where the review protocol can be accessed, or state that a protocol was not prepared. | N | N | - | N |
|  | 24c | Describe and explain any amendments to information provided at registration or in the protocol. | PY | N | N | N |
| Support | 25 | Describe sources of financial or non-financial support for the review, and the role of the funders or sponsors in the review. | N | N | - | N |
| Competing interests | 26 | Declare any competing interests of review authors. | Y | Y | - | Y |
| Availability of data,  code, and other  materials | 27 | Report which of the following are publicly available and where they can be found: template data collection forms; data extracted from included studies; data used for all analyses; analytic code; any other materials used in the review. | PY | PY | - | PY |

PRISMA 2020

Y: Yes; N: No; PY: Partial Yes.

A:The conclusions of researcher Ph.D.Yongxiu Liu.

B:The conclusions of researcher Ph.D. Yuguo Li.

C:In case of a difference of opinions, it shall be adjudicated by Professor Lei Gao.

D:Conclusive conclusion.

Study 36：Nitzan, Itamar et al. “Vitamin D and Asthma: a Systematic Review of Clinical Trials.” Current nutrition reports vol. 11,2 (2022): 311-317. doi:10.1007/s13668-022-00411-6.PMID: 35347665

| Section and topic | Item # | Checklist item | A | B | C | D |
| --- | --- | --- | --- | --- | --- | --- |
| **Title** | | | | | | |
| Title | 1 | Identify the report as a systematic review. | Y | PY | Y | Y |
| **Abstract** | | | | | | |
| Abstract | 2 | See the PRISMA 2020 for Abstracts checklist (table 2). | PY | PY | - | PY |
| **Introduction** | | | | | | |
| Rationale | 3 | Describe the rationale for the review in the context of existing knowledge | Y | Y | - | Y |
| Objectives | 4 | Provide an explicit statement of the objective(s) or question(s) the review addresses. | Y | Y | - | Y |
| **Methods** | | | | | | |
| Eligibility criteria | 5 | Specify the inclusion and exclusion criteria for the review and how studies were grouped for the syntheses. | PY | Y | Y | Y |
| Information sources | 6 | Specify all databases, registers, websites, organisations, reference lists and other sources searched or consulted to identify studies. Specify the date when each source was last searched or consulted. | Y | PY | Y | Y |
| Search strategy | 7 | Present the full search strategies for all databases, registers and websites, including any filters and limits used | PY | PY | - | PY |
| Selection process | 8 | Specify the methods used to decide whether a study met the inclusion criteria of the review, including how many reviewers screened each record and each report retrieved, whether they worked independently, and if applicable, details of automation tools  used in the process. | PY | PY | - | PY |
| Data collection  process | 9 | Specify the methods used to collect data from reports, including how many reviewers collected data from each report, whether they worked independently, any processes for obtaining or confirming data from study investigators, and if applicable, details of automation tools used in the process. | PY | PY | - | PY |
| Data items | 10a | List and define all outcomes for which data were sought. Specify whether all results that were compatible with each outcome domain in each study were sought (e.g. for all measures, time points, analyses), and if not, the methods used to decide which  results to collect. | PY | PY | - | PY |
|  | 10b | List and define all other variables for which data were sought (e.g. participant and intervention characteristics, funding sources). Describe any assumptions made about any missing or unclear information. | PY | PY | - | PY |
| Study risk of bias  assessment | 11 | Specify the methods used to assess risk of bias in the included studies, including details of the tool(s) used, how many reviewers assessed each study and whether they worked independently, and if applicable, details of automation tools used in the process. | N | N | - | N |
| Effect measures | 12 | Specify for each outcome the effect measure(s) (e.g. risk ratio, mean difference) used in the synthesis or presentation of results. | PY | Y | PY | PY |
| Synthesis methods | 13a | Describe the processes used to decide which studies were eligible for each synthesis (e.g. tabulating the study intervention characteristics and comparing against the planned groups for each synthesis (item #5)). | PY | PY | - | PY |
|  | 13b | Describe any methods required to prepare the data for presentation or synthesis, such as handling of missing summary statistics, or data conversions. | PY | PY | - | PY |
|  | 13c | Describe any methods used to tabulate or visually display results of individual studies and syntheses. | PY | Y | Y | Y |
|  | 13d | Describe any methods used to synthesise results and provide a rationale for the choice(s). If meta-analysis was performed, describe the model(s), method(s) to identify the presence and extent of statistical heterogeneity, and software package(s) used. | N | PY | PY | PY |
|  | 13e | Describe any methods used to explore possible causes of heterogeneity among study results (e.g. subgroup analysis, meta regression). | N | Y | PY | PY |
|  | 13f | Describe any sensitivity analyses conducted to assess robustness of the synthesised results. | N | N | - | N |
| Reporting bias  assessment | 14 | Describe any methods used to assess risk of bias due to missing results in a synthesis (arising from reporting biases). | N | N | - | N |
| Certainty assessment | 15 | Describe any methods used to assess certainty (or confidence) in the body of evidence for an outcome. | N | N | - | N |
| **Results** | | | | | | |
| Study selection | 16a | Describe the results of the search and selection process, from the number of records identified in the search to the number of studies included in the review, ideally using a flow diagram (see fig 1). | Y | Y | - | Y |
|  | 16b | Cite studies that might appear to meet the inclusion criteria, but which were excluded, and explain why they were excluded. | N | PY | PY | PY |
| Study characteristics | 17 | Cite each included study and present its characteristics. | PY | Y | Y | Y |
| Risk of bias in studies | 18 | Present assessments of risk of bias for each included study. | N | N | - | N |
| Results of individual  studies | 19 | For all outcomes, present, for each study: (a) summary statistics for each group (where appropriate) and (b) an effect estimate and its precision (e.g. confidence/credible interval), ideally using structured tables or plots. | NY | Y | PY | PY |
| Results of syntheses | 20a | For each synthesis, briefly summarise the characteristics and risk of bias among contributing studies. | PY | PY | PY | PY |
|  | 20b | Present results of all statistical syntheses conducted. If meta-analysis was done, present for each the summary estimate and its precision (e.g. confidence/credible interval) and measures of statistical heterogeneity. If comparing groups, describe the direction of the effect. | N | Y | PY | PY |
|  | 20c | Present results of all investigations of possible causes of heterogeneity among study results. | N | Y | N | N |
|  | 20d | Present results of all sensitivity analyses conducted to assess the robustness of the synthesised results. | N | N | - | N |
| Reporting biases | 21 | Present assessments of risk of bias due to missing results (arising from reporting biases) for each synthesis assessed. | N | N | - | Y |
| Certainty of evidence | 22 | Present assessments of certainty (or confidence) in the body of evidence for each outcome assessed. | N | N | - | N |
| **Discussion** | | | | | | |
| Discussion | 23a | Provide a general interpretation of the results in the context of other evidence. | Y | Y | - | Y |
|  | 23b | Discuss any limitations of the evidence included in the review. | Y | Y | - | Y |
|  | 23c | Discuss any limitations of the review processes used. | PY | PY | - | PY |
|  | 23d | Discuss implications of the results for practice, policy, and future research. | Y | Y | - | Y |
| **Other information** | | | | | | |
| Registration and  protocol | 24a | Provide registration information for the review, including register name and registration number, or state that the review was not registered. | N | N | - | N |
|  | 24b | Indicate where the review protocol can be accessed, or state that a protocol was not prepared. | N | N | - | N |
|  | 24c | Describe and explain any amendments to information provided at registration or in the protocol. | Y | N | N | N |
| Support | 25 | Describe sources of financial or non-financial support for the review, and the role of the funders or sponsors in the review. | N | N | - | N |
| Competing interests | 26 | Declare any competing interests of review authors. | Y | Y | - | Y |
| Availability of data,  code, and other  materials | 27 | Report which of the following are publicly available and where they can be found: template data collection forms; data extracted from included studies; data used for all analyses; analytic code; any other materials used in the review. | N | PY | N | N |

PRISMA 2020

Y: Yes; N: No; PY: Partial Yes.

A:The conclusions of researcher Ph.D.Yongxiu Liu.

B:The conclusions of researcher Ph.D. Yuguo Li.

C:In case of a difference of opinions, it shall be adjudicated by Professor Lei Gao.

D:Conclusive conclusion.

Study 37：Williamson, Anne et al. “Vitamin D for the management of asthma.” The Cochrane database of systematic reviews vol. 2,2 CD011511. 6 Feb. 2023, doi:10.1002/14651858.CD011511.pub3.PMID: 36744416 PMCID: PMC9899558

| Section and topic | Item # | Checklist item | A | B | C | D |
| --- | --- | --- | --- | --- | --- | --- |
| **Title** | | | | | | |
| Title | 1 | Identify the report as a systematic review. | Y | Y | - | Y |
| **Abstract** | | | | | | |
| Abstract | 2 | See the PRISMA 2020 for Abstracts checklist (table 2). | PY | Y | - | Y |
| **Introduction** | | | | | | |
| Rationale | 3 | Describe the rationale for the review in the context of existing knowledge | Y | Y | - | Y |
| Objectives | 4 | Provide an explicit statement of the objective(s) or question(s) the review addresses. | Y | Y | - | Y |
| **Methods** | | | | | | |
| Eligibility criteria | 5 | Specify the inclusion and exclusion criteria for the review and how studies were grouped for the syntheses. | Y | Y | - | Y |
| Information sources | 6 | Specify all databases, registers, websites, organisations, reference lists and other sources searched or consulted to identify studies. Specify the date when each source was last searched or consulted. | Y | Y | - | Y |
| Search strategy | 7 | Present the full search strategies for all databases, registers and websites, including any filters and limits used | Y | Y | - | Y |
| Selection process | 8 | Specify the methods used to decide whether a study met the inclusion criteria of the review, including how many reviewers screened each record and each report retrieved, whether they worked independently, and if applicable, details of automation tools  used in the process. | Y | Y | - | Y |
| Data collection  process | 9 | Specify the methods used to collect data from reports, including how many reviewers collected data from each report, whether they worked independently, any processes for obtaining or confirming data from study investigators, and if applicable, details of automation tools used in the process. | Y | Y | - | Y |
| Data items | 10a | List and define all outcomes for which data were sought. Specify whether all results that were compatible with each outcome domain in each study were sought (e.g. for all measures, time points, analyses), and if not, the methods used to decide which  results to collect. | Y | Y | - | Y |
|  | 10b | List and define all other variables for which data were sought (e.g. participant and intervention characteristics, funding sources). Describe any assumptions made about any missing or unclear information. | Y | Y | - | Y |
| Study risk of bias  assessment | 11 | Specify the methods used to assess risk of bias in the included studies, including details of the tool(s) used, how many reviewers assessed each study and whether they worked independently, and if applicable, details of automation tools used in the process. | Y | Y | - | Y |
| Effect measures | 12 | Specify for each outcome the effect measure(s) (e.g. risk ratio, mean difference) used in the synthesis or presentation of results. | Y | Y | - | Y |
| Synthesis methods | 13a | Describe the processes used to decide which studies were eligible for each synthesis (e.g. tabulating the study intervention characteristics and comparing against the planned groups for each synthesis (item #5)). | Y | Y | - | Y |
|  | 13b | Describe any methods required to prepare the data for presentation or synthesis, such as handling of missing summary statistics, or data conversions. | Y | Y | - | Y |
|  | 13c | Describe any methods used to tabulate or visually display results of individual studies and syntheses. | Y | Y | - | Y |
|  | 13d | Describe any methods used to synthesise results and provide a rationale for the choice(s). If meta-analysis was performed, describe the model(s), method(s) to identify the presence and extent of statistical heterogeneity, and software package(s) used. | Y | Y | - | Y |
|  | 13e | Describe any methods used to explore possible causes of heterogeneity among study results (e.g. subgroup analysis, meta regression). | Y | Y | - | Y |
|  | 13f | Describe any sensitivity analyses conducted to assess robustness of the synthesised results. | Y | Y | - | Y |
| Reporting bias  assessment | 14 | Describe any methods used to assess risk of bias due to missing results in a synthesis (arising from reporting biases). | Y | Y | - | Y |
| Certainty assessment | 15 | Describe any methods used to assess certainty (or confidence) in the body of evidence for an outcome. | Y | Y | - | Y |
| **Results** | | | | | | |
| Study selection | 16a | Describe the results of the search and selection process, from the number of records identified in the search to the number of studies included in the review, ideally using a flow diagram (see fig 1). | Y | Y | - | Y |
|  | 16b | Cite studies that might appear to meet the inclusion criteria, but which were excluded, and explain why they were excluded. | Y | Y | - | Y |
| Study characteristics | 17 | Cite each included study and present its characteristics. | Y | Y | - | Y |
| Risk of bias in studies | 18 | Present assessments of risk of bias for each included study. | Y | Y | - | Y |
| Results of individual  studies | 19 | For all outcomes, present, for each study: (a) summary statistics for each group (where appropriate) and (b) an effect estimate and its precision (e.g. confidence/credible interval), ideally using structured tables or plots. | Y | Y | - | Y |
| Results of syntheses | 20a | For each synthesis, briefly summarise the characteristics and risk of bias among contributing studies. | Y | Y | - | Y |
|  | 20b | Present results of all statistical syntheses conducted. If meta-analysis was done, present for each the summary estimate and its precision (e.g. confidence/credible interval) and measures of statistical heterogeneity. If comparing groups, describe the direction of the effect. | Y | Y | - | Y |
|  | 20c | Present results of all investigations of possible causes of heterogeneity among study results. | Y | Y | - | Y |
|  | 20d | Present results of all sensitivity analyses conducted to assess the robustness of the synthesised results. | Y | Y | - | Y |
| Reporting biases | 21 | Present assessments of risk of bias due to missing results (arising from reporting biases) for each synthesis assessed. | Y | Y | - | Y |
| Certainty of evidence | 22 | Present assessments of certainty (or confidence) in the body of evidence for each outcome assessed. | Y | Y | - | Y |
| **Discussion** | | | | | | |
| Discussion | 23a | Provide a general interpretation of the results in the context of other evidence. | Y | Y | - | Y |
|  | 23b | Discuss any limitations of the evidence included in the review. | Y | Y | - | Y |
|  | 23c | Discuss any limitations of the review processes used. | Y | Y | - | Y |
|  | 23d | Discuss implications of the results for practice, policy, and future research. | Y | Y | - | Y |
| **Other information** | | | | | | |
| Registration and  protocol | 24a | Provide registration information for the review, including register name and registration number, or state that the review was not registered. | Y | Y | - | Y |
|  | 24b | Indicate where the review protocol can be accessed, or state that a protocol was not prepared. | Y | Y | - | Y |
|  | 24c | Describe and explain any amendments to information provided at registration or in the protocol. | Y | Y | - | Y |
| Support | 25 | Describe sources of financial or non-financial support for the review, and the role of the funders or sponsors in the review. | Y | Y | - | Y |
| Competing interests | 26 | Declare any competing interests of review authors. | Y | Y | - | Y |
| Availability of data,  code, and other  materials | 27 | Report which of the following are publicly available and where they can be found: template data collection forms; data extracted from included studies; data used for all analyses; analytic code; any other materials used in the review. | PY | PY | - | PY |

PRISMA 2020

Y: Yes; N: No; PY: Partial Yes.

A:The conclusions of researcher Ph.D.Yongxiu Liu.

B:The conclusions of researcher Ph.D. Yuguo Li.

C:In case of a difference of opinions, it shall be adjudicated by Professor Lei Gao.

D:Conclusive conclusion.

Study 38：孙倩.补充维生素D及其类似物对支气管哮喘的影响：一项随机对照试验的荟萃分析[D].南昌大学,2024.DOI:10.27232/d.cnki.gnchu.2024.003676.

| Section and topic | Item # | Checklist item | A | B | C | D |
| --- | --- | --- | --- | --- | --- | --- |
| **Title** | | | | | | |
| Title | 1 | Identify the report as a systematic review. | Y | Y | - | Y |
| **Abstract** | | | | | | |
| Abstract | 2 | See the PRISMA 2020 for Abstracts checklist (table 2). | PY | PY | - | PY |
| **Introduction** | | | | | | |
| Rationale | 3 | Describe the rationale for the review in the context of existing knowledge | Y | Y | - | Y |
| Objectives | 4 | Provide an explicit statement of the objective(s) or question(s) the review addresses. | Y | Y | - | Y |
| **Methods** | | | | | | |
| Eligibility criteria | 5 | Specify the inclusion and exclusion criteria for the review and how studies were grouped for the syntheses. | Y | Y | - | Y |
| Information sources | 6 | Specify all databases, registers, websites, organisations, reference lists and other sources searched or consulted to identify studies. Specify the date when each source was last searched or consulted. | Y | Y | - | Y |
| Search strategy | 7 | Present the full search strategies for all databases, registers and websites, including any filters and limits used | PY | PY | - | PY |
| Selection process | 8 | Specify the methods used to decide whether a study met the inclusion criteria of the review, including how many reviewers screened each record and each report retrieved, whether they worked independently, and if applicable, details of automation tools  used in the process. | Y | Y | - | Y |
| Data collection  process | 9 | Specify the methods used to collect data from reports, including how many reviewers collected data from each report, whether they worked independently, any processes for obtaining or confirming data from study investigators, and if applicable, details of automation tools used in the process. | Y | PY | Y | Y |
| Data items | 10a | List and define all outcomes for which data were sought. Specify whether all results that were compatible with each outcome domain in each study were sought (e.g. for all measures, time points, analyses), and if not, the methods used to decide which  results to collect. | Y | PY | Y | Y |
|  | 10b | List and define all other variables for which data were sought (e.g. participant and intervention characteristics, funding sources). Describe any assumptions made about any missing or unclear information. | PY | PY | - | PY |
| Study risk of bias  assessment | 11 | Specify the methods used to assess risk of bias in the included studies, including details of the tool(s) used, how many reviewers assessed each study and whether they worked independently, and if applicable, details of automation tools used in the process. | Y | Y | - | Y |
| Effect measures | 12 | Specify for each outcome the effect measure(s) (e.g. risk ratio, mean difference) used in the synthesis or presentation of results. | Y | Y | - | Y |
| Synthesis methods | 13a | Describe the processes used to decide which studies were eligible for each synthesis (e.g. tabulating the study intervention characteristics and comparing against the planned groups for each synthesis (item #5)). | Y | Y | - | Y |
|  | 13b | Describe any methods required to prepare the data for presentation or synthesis, such as handling of missing summary statistics, or data conversions. | Y | PY | Y | Y |
|  | 13c | Describe any methods used to tabulate or visually display results of individual studies and syntheses. | Y | PY | Y | Y |
|  | 13d | Describe any methods used to synthesise results and provide a rationale for the choice(s). If meta-analysis was performed, describe the model(s), method(s) to identify the presence and extent of statistical heterogeneity, and software package(s) used. | Y | Y | - | Y |
|  | 13e | Describe any methods used to explore possible causes of heterogeneity among study results (e.g. subgroup analysis, meta regression). | Y | Y | - | Y |
|  | 13f | Describe any sensitivity analyses conducted to assess robustness of the synthesised results. | PY | N | Y | Y |
| Reporting bias  assessment | 14 | Describe any methods used to assess risk of bias due to missing results in a synthesis (arising from reporting biases). | Y | Y | - | Y |
| Certainty assessment | 15 | Describe any methods used to assess certainty (or confidence) in the body of evidence for an outcome. | N | Y | N | N |
| **Results** | | | | | | |
| Study selection | 16a | Describe the results of the search and selection process, from the number of records identified in the search to the number of studies included in the review, ideally using a flow diagram (see fig 1). | Y | Y | - | Y |
|  | 16b | Cite studies that might appear to meet the inclusion criteria, but which were excluded, and explain why they were excluded. | N | N | - | N |
| Study characteristics | 17 | Cite each included study and present its characteristics. | Y | Y | - | Y |
| Risk of bias in studies | 18 | Present assessments of risk of bias for each included study. | Y | Y | - | Y |
| Results of individual  studies | 19 | For all outcomes, present, for each study: (a) summary statistics for each group (where appropriate) and (b) an effect estimate and its precision (e.g. confidence/credible interval), ideally using structured tables or plots. | Y | Y | - | Y |
| Results of syntheses | 20a | For each synthesis, briefly summarise the characteristics and risk of bias among contributing studies. | Y | Y | - | Y |
|  | 20b | Present results of all statistical syntheses conducted. If meta-analysis was done, present for each the summary estimate and its precision (e.g. confidence/credible interval) and measures of statistical heterogeneity. If comparing groups, describe the direction of the effect. | Y | Y | - | Y |
|  | 20c | Present results of all investigations of possible causes of heterogeneity among study results. | Y | Y | - | Y |
|  | 20d | Present results of all sensitivity analyses conducted to assess the robustness of the synthesised results. | Y | PY | Y | Y |
| Reporting biases | 21 | Present assessments of risk of bias due to missing results (arising from reporting biases) for each synthesis assessed. | Y | PY | Y | Y |
| Certainty of evidence | 22 | Present assessments of certainty (or confidence) in the body of evidence for each outcome assessed. | N | N | - | N |
| **Discussion** | | | | | | |
| Discussion | 23a | Provide a general interpretation of the results in the context of other evidence. | Y | Y | - | Y |
|  | 23b | Discuss any limitations of the evidence included in the review. | Y | Y | - | Y |
|  | 23c | Discuss any limitations of the review processes used. | Y | PY | Y | Y |
|  | 23d | Discuss implications of the results for practice, policy, and future research. | Y | Y | - | Y |
| **Other information** | | | | | | |
| Registration and  protocol | 24a | Provide registration information for the review, including register name and registration number, or state that the review was not registered. | N | N | - | N |
|  | 24b | Indicate where the review protocol can be accessed, or state that a protocol was not prepared. | N | N | - | N |
|  | 24c | Describe and explain any amendments to information provided at registration or in the protocol. | N | N | - | N |
| Support | 25 | Describe sources of financial or non-financial support for the review, and the role of the funders or sponsors in the review. | N | N | - | N |
| Competing interests | 26 | Declare any competing interests of review authors. | Y | N | N | N |
| Availability of data,  code, and other  materials | 27 | Report which of the following are publicly available and where they can be found: template data collection forms; data extracted from included studies; data used for all analyses; analytic code; any other materials used in the review. | N | N | - | N |

PRISMA 2020

Y: Yes; N: No; PY: Partial Yes.

A:The conclusions of researcher Ph.D.Yongxiu Liu.

B:The conclusions of researcher Ph.D. Yuguo Li.

C:In case of a difference of opinions, it shall be adjudicated by Professor Lei Gao.

D:Conclusive conclusion.

Study 39：El Abd, Asmae et al. “The effects of vitamin D supplementation on inflammatory biomarkers in patients with asthma: a systematic review and meta-analysis of randomized controlled trials.”Frontiers in immunology vol. 15 1335968. 13 Mar. 2024, doi:10.3389/fimmu.2024.1335968.PMID: 38545098 PMCID: PMC10965564

| Section and topic | Item # | Checklist item | A | B | C | D |
| --- | --- | --- | --- | --- | --- | --- |
| **Title** | | | | | | |
| Title | 1 | Identify the report as a systematic review. | Y | Y | - | Y |
| **Abstract** | | | | | | |
| Abstract | 2 | See the PRISMA 2020 for Abstracts checklist (table 2). | PY | PY | - | PY |
| **Introduction** | | | | | | |
| Rationale | 3 | Describe the rationale for the review in the context of existing knowledge | Y | Y | - | Y |
| Objectives | 4 | Provide an explicit statement of the objective(s) or question(s) the review addresses. | Y | Y | - | Y |
| **Methods** | | | | | | |
| Eligibility criteria | 5 | Specify the inclusion and exclusion criteria for the review and how studies were grouped for the syntheses. | Y | Y | - | Y |
| Information sources | 6 | Specify all databases, registers, websites, organisations, reference lists and other sources searched or consulted to identify studies. Specify the date when each source was last searched or consulted. | Y | Y | - | Y |
| Search strategy | 7 | Present the full search strategies for all databases, registers and websites, including any filters and limits used | PY | PY | - | PY |
| Selection process | 8 | Specify the methods used to decide whether a study met the inclusion criteria of the review, including how many reviewers screened each record and each report retrieved, whether they worked independently, and if applicable, details of automation tools  used in the process. | Y | Y | - | Y |
| Data collection  process | 9 | Specify the methods used to collect data from reports, including how many reviewers collected data from each report, whether they worked independently, any processes for obtaining or confirming data from study investigators, and if applicable, details of automation tools used in the process. | Y | Y | - | Y |
| Data items | 10a | List and define all outcomes for which data were sought. Specify whether all results that were compatible with each outcome domain in each study were sought (e.g. for all measures, time points, analyses), and if not, the methods used to decide which  results to collect. | Y | Y | - | Y |
|  | 10b | List and define all other variables for which data were sought (e.g. participant and intervention characteristics, funding sources). Describe any assumptions made about any missing or unclear information. | PY | PY | - | PY |
| Study risk of bias  assessment | 11 | Specify the methods used to assess risk of bias in the included studies, including details of the tool(s) used, how many reviewers assessed each study and whether they worked independently, and if applicable, details of automation tools used in the process. | Y | Y | - | Y |
| Effect measures | 12 | Specify for each outcome the effect measure(s) (e.g. risk ratio, mean difference) used in the synthesis or presentation of results. | Y | Y | - | Y |
| Synthesis methods | 13a | Describe the processes used to decide which studies were eligible for each synthesis (e.g. tabulating the study intervention characteristics and comparing against the planned groups for each synthesis (item #5)). | Y | Y | - | Y |
|  | 13b | Describe any methods required to prepare the data for presentation or synthesis, such as handling of missing summary statistics, or data conversions. | Y | Y | - | Y |
|  | 13c | Describe any methods used to tabulate or visually display results of individual studies and syntheses. | Y | Y | - | Y |
|  | 13d | Describe any methods used to synthesise results and provide a rationale for the choice(s). If meta-analysis was performed, describe the model(s), method(s) to identify the presence and extent of statistical heterogeneity, and software package(s) used. | Y | Y | - | Y |
|  | 13e | Describe any methods used to explore possible causes of heterogeneity among study results (e.g. subgroup analysis, meta regression). | Y | Y | - | Y |
|  | 13f | Describe any sensitivity analyses conducted to assess robustness of the synthesised results. | Y | Y | - | Y |
| Reporting bias  assessment | 14 | Describe any methods used to assess risk of bias due to missing results in a synthesis (arising from reporting biases). | N | N | - | N |
| Certainty assessment | 15 | Describe any methods used to assess certainty (or confidence) in the body of evidence for an outcome. | N | N | - | N |
| **Results** | | | | | | |
| Study selection | 16a | Describe the results of the search and selection process, from the number of records identified in the search to the number of studies included in the review, ideally using a flow diagram (see fig 1). | Y | Y | - | Y |
|  | 16b | Cite studies that might appear to meet the inclusion criteria, but which were excluded, and explain why they were excluded. | N | PY | N | N |
| Study characteristics | 17 | Cite each included study and present its characteristics. | Y | Y | - | Y |
| Risk of bias in studies | 18 | Present assessments of risk of bias for each included study. | Y | Y | - | Y |
| Results of individual  studies | 19 | For all outcomes, present, for each study: (a) summary statistics for each group (where appropriate) and (b) an effect estimate and its precision (e.g. confidence/credible interval), ideally using structured tables or plots. | Y | Y | - | Y |
| Results of syntheses | 20a | For each synthesis, briefly summarise the characteristics and risk of bias among contributing studies. | Y | Y | - | Y |
|  | 20b | Present results of all statistical syntheses conducted. If meta-analysis was done, present for each the summary estimate and its precision (e.g. confidence/credible interval) and measures of statistical heterogeneity. If comparing groups, describe the direction of the effect. | Y | Y | - | Y |
|  | 20c | Present results of all investigations of possible causes of heterogeneity among study results. | Y | Y | - | Y |
|  | 20d | Present results of all sensitivity analyses conducted to assess the robustness of the synthesised results. | Y | Y | - | Y |
| Reporting biases | 21 | Present assessments of risk of bias due to missing results (arising from reporting biases) for each synthesis assessed. | N | N | - | N |
| Certainty of evidence | 22 | Present assessments of certainty (or confidence) in the body of evidence for each outcome assessed. | N | N | - | N |
| **Discussion** | | | | | | |
| Discussion | 23a | Provide a general interpretation of the results in the context of other evidence. | Y | Y | - | Y |
|  | 23b | Discuss any limitations of the evidence included in the review. | Y | Y | - | Y |
|  | 23c | Discuss any limitations of the review processes used. | Y | Y | - | Y |
|  | 23d | Discuss implications of the results for practice, policy, and future research. | Y | Y | - | Y |
| **Other information** | | | | | | |
| Registration and  protocol | 24a | Provide registration information for the review, including register name and registration number, or state that the review was not registered. | Y | Y | - | Y |
|  | 24b | Indicate where the review protocol can be accessed, or state that a protocol was not prepared. | N | PY | Y | Y |
|  | 24c | Describe and explain any amendments to information provided at registration or in the protocol. | N | N | - | N |
| Support | 25 | Describe sources of financial or non-financial support for the review, and the role of the funders or sponsors in the review. | Y | Y | - | Y |
| Competing interests | 26 | Declare any competing interests of review authors. | Y | Y | - | Y |
| Availability of data,  code, and other  materials | 27 | Report which of the following are publicly available and where they can be found: template data collection forms; data extracted from included studies; data used for all analyses; analytic code; any other materials used in the review. | PY | PY | - | PY |

PRISMA 2020

Y: Yes; N: No; PY: Partial Yes.

A:The conclusions of researcher Ph.D.Yongxiu Liu.

B:The conclusions of researcher Ph.D. Yuguo Li.

C:In case of a difference of opinions, it shall be adjudicated by Professor Lei Gao.

D:Conclusive conclusion.

Study 40：Fedora, Katherine et al. “Vitamin D supplementation decrease asthma exacerbations in children: a systematic review and meta-analysis of randomized controlled trials.” Annals of medicine vol. 56,1 (2024): 2400313. doi:10.1080/07853890.2024.2400313.PMID: 39421966.PMCID: PMC11492411

| Section and topic | Item # | Checklist item | A | B | C | D |
| --- | --- | --- | --- | --- | --- | --- |
| **Title** | | | | | | |
| Title | 1 | Identify the report as a systematic review. | Y | Y | - | Y |
| **Abstract** | | | | | | |
| Abstract | 2 | See the PRISMA 2020 for Abstracts checklist (table 2). | PY | PY | - | PY |
| **Introduction** | | | | | | |
| Rationale | 3 | Describe the rationale for the review in the context of existing knowledge | Y | Y | - | Y |
| Objectives | 4 | Provide an explicit statement of the objective(s) or question(s) the review addresses. | Y | Y | - | Y |
| **Methods** | | | | | | |
| Eligibility criteria | 5 | Specify the inclusion and exclusion criteria for the review and how studies were grouped for the syntheses. | Y | Y | - | Y |
| Information sources | 6 | Specify all databases, registers, websites, organisations, reference lists and other sources searched or consulted to identify studies. Specify the date when each source was last searched or consulted. | Y | PY | Y | Y |
| Search strategy | 7 | Present the full search strategies for all databases, registers and websites, including any filters and limits used | PY | PY | - | PY |
| Selection process | 8 | Specify the methods used to decide whether a study met the inclusion criteria of the review, including how many reviewers screened each record and each report retrieved, whether they worked independently, and if applicable, details of automation tools  used in the process. | Y | Y | - | Y |
| Data collection  process | 9 | Specify the methods used to collect data from reports, including how many reviewers collected data from each report, whether they worked independently, any processes for obtaining or confirming data from study investigators, and if applicable, details of automation tools used in the process. | Y | Y | - | Y |
| Data items | 10a | List and define all outcomes for which data were sought. Specify whether all results that were compatible with each outcome domain in each study were sought (e.g. for all measures, time points, analyses), and if not, the methods used to decide which  results to collect. | Y | Y | - | Y |
|  | 10b | List and define all other variables for which data were sought (e.g. participant and intervention characteristics, funding sources). Describe any assumptions made about any missing or unclear information. | PY | PY | - | PY |
| Study risk of bias  assessment | 11 | Specify the methods used to assess risk of bias in the included studies, including details of the tool(s) used, how many reviewers assessed each study and whether they worked independently, and if applicable, details of automation tools used in the process. | Y | Y | - | Y |
| Effect measures | 12 | Specify for each outcome the effect measure(s) (e.g. risk ratio, mean difference) used in the synthesis or presentation of results. | Y | Y | - | Y |
| Synthesis methods | 13a | Describe the processes used to decide which studies were eligible for each synthesis (e.g. tabulating the study intervention characteristics and comparing against the planned groups for each synthesis (item #5)). | Y | Y | - | Y |
|  | 13b | Describe any methods required to prepare the data for presentation or synthesis, such as handling of missing summary statistics, or data conversions. | Y | Y | - | Y |
|  | 13c | Describe any methods used to tabulate or visually display results of individual studies and syntheses. | Y | Y | - | Y |
|  | 13d | Describe any methods used to synthesise results and provide a rationale for the choice(s). If meta-analysis was performed, describe the model(s), method(s) to identify the presence and extent of statistical heterogeneity, and software package(s) used. | Y | Y | - | Y |
|  | 13e | Describe any methods used to explore possible causes of heterogeneity among study results (e.g. subgroup analysis, meta regression). | Y | Y | - | Y |
|  | 13f | Describe any sensitivity analyses conducted to assess robustness of the synthesised results. | N | N | - | N |
| Reporting bias  assessment | 14 | Describe any methods used to assess risk of bias due to missing results in a synthesis (arising from reporting biases). | PY | PY | - | PY |
| Certainty assessment | 15 | Describe any methods used to assess certainty (or confidence) in the body of evidence for an outcome. | N | N | - | N |
| **Results** | | | | | | |
| Study selection | 16a | Describe the results of the search and selection process, from the number of records identified in the search to the number of studies included in the review, ideally using a flow diagram (see fig 1). | Y | Y | - | Y |
|  | 16b | Cite studies that might appear to meet the inclusion criteria, but which were excluded, and explain why they were excluded. | N | PY | PY | PY |
| Study characteristics | 17 | Cite each included study and present its characteristics. | Y | Y | - | Y |
| Risk of bias in studies | 18 | Present assessments of risk of bias for each included study. | Y | Y | - | Y |
| Results of individual  studies | 19 | For all outcomes, present, for each study: (a) summary statistics for each group (where appropriate) and (b) an effect estimate and its precision (e.g. confidence/credible interval), ideally using structured tables or plots. | Y | Y | - | Y |
| Results of syntheses | 20a | For each synthesis, briefly summarise the characteristics and risk of bias among contributing studies. | Y | Y | - | Y |
|  | 20b | Present results of all statistical syntheses conducted. If meta-analysis was done, present for each the summary estimate and its precision (e.g. confidence/credible interval) and measures of statistical heterogeneity. If comparing groups, describe the direction of the effect. | Y | Y | - | Y |
|  | 20c | Present results of all investigations of possible causes of heterogeneity among study results. | Y | Y | - | Y |
|  | 20d | Present results of all sensitivity analyses conducted to assess the robustness of the synthesised results. | N | N | - | N |
| Reporting biases | 21 | Present assessments of risk of bias due to missing results (arising from reporting biases) for each synthesis assessed. | PY | PY | - | PY |
| Certainty of evidence | 22 | Present assessments of certainty (or confidence) in the body of evidence for each outcome assessed. | N | N | - | N |
| **Discussion** | | | | | | |
| Discussion | 23a | Provide a general interpretation of the results in the context of other evidence. | Y | Y | - | Y |
|  | 23b | Discuss any limitations of the evidence included in the review. | Y | Y | - | Y |
|  | 23c | Discuss any limitations of the review processes used. | Y | Y | - | Y |
|  | 23d | Discuss implications of the results for practice, policy, and future research. | Y | Y | - | Y |
| **Other information** | | | | | | |
| Registration and  protocol | 24a | Provide registration information for the review, including register name and registration number, or state that the review was not registered. | Y | Y | - | Y |
|  | 24b | Indicate where the review protocol can be accessed, or state that a protocol was not prepared. | N | N | - | N |
|  | 24c | Describe and explain any amendments to information provided at registration or in the protocol. | N | N | - | N |
| Support | 25 | Describe sources of financial or non-financial support for the review, and the role of the funders or sponsors in the review. | Y | Y | - | Y |
| Competing interests | 26 | Declare any competing interests of review authors. | Y | Y | - | Y |
| Availability of data,  code, and other  materials | 27 | Report which of the following are publicly available and where they can be found: template data collection forms; data extracted from included studies; data used for all analyses; analytic code; any other materials used in the review. | PY | PY | - | PY |

PRISMA 2020

Y: Yes; N: No; PY: Partial Yes.

A:The conclusions of researcher Ph.D.Yongxiu Liu.

B:The conclusions of researcher Ph.D. Yuguo Li.

C:In case of a difference of opinions, it shall be adjudicated by Professor Lei Gao.

D:Conclusive conclusion.

Study 41：Niu, Haiying et al. “Asthmatic patients with vitamin D deficiency: Can vitamin D supplementation make a difference.” Technology and health care : official journal of the European Society for Engineering and Medicine vol. 32,6 (2024): 3985-4008. doi:10.3233/THC-231462.PMID: 39031398.PMCID: PMC11612934

| Section and topic | Item # | Checklist item | A | B | C | D |
| --- | --- | --- | --- | --- | --- | --- |
| **Title** | | | | | | |
| Title | 1 | Identify the report as a systematic review. | PY | Y | N | N |
| **Abstract** | | | | | | |
| Abstract | 2 | See the PRISMA 2020 for Abstracts checklist (table 2). | PY | PY | - | PY |
| **Introduction** | | | | | | |
| Rationale | 3 | Describe the rationale for the review in the context of existing knowledge | Y | Y | - | Y |
| Objectives | 4 | Provide an explicit statement of the objective(s) or question(s) the review addresses. | Y | Y | - | Y |
| **Methods** | | | | | | |
| Eligibility criteria | 5 | Specify the inclusion and exclusion criteria for the review and how studies were grouped for the syntheses. | Y | PY | PY | PY |
| Information sources | 6 | Specify all databases, registers, websites, organisations, reference lists and other sources searched or consulted to identify studies. Specify the date when each source was last searched or consulted. | Y | PY | PY | PY |
| Search strategy | 7 | Present the full search strategies for all databases, registers and websites, including any filters and limits used | PY | PY | - | PY |
| Selection process | 8 | Specify the methods used to decide whether a study met the inclusion criteria of the review, including how many reviewers screened each record and each report retrieved, whether they worked independently, and if applicable, details of automation tools  used in the process. | Y | Y | - | Y |
| Data collection  process | 9 | Specify the methods used to collect data from reports, including how many reviewers collected data from each report, whether they worked independently, any processes for obtaining or confirming data from study investigators, and if applicable, details of automation tools used in the process. | Y | Y | - | Y |
| Data items | 10a | List and define all outcomes for which data were sought. Specify whether all results that were compatible with each outcome domain in each study were sought (e.g. for all measures, time points, analyses), and if not, the methods used to decide which  results to collect. | Y | Y | - | Y |
|  | 10b | List and define all other variables for which data were sought (e.g. participant and intervention characteristics, funding sources). Describe any assumptions made about any missing or unclear information. | PY | PY | - | PY |
| Study risk of bias  assessment | 11 | Specify the methods used to assess risk of bias in the included studies, including details of the tool(s) used, how many reviewers assessed each study and whether they worked independently, and if applicable, details of automation tools used in the process. | Y | Y | - | Y |
| Effect measures | 12 | Specify for each outcome the effect measure(s) (e.g. risk ratio, mean difference) used in the synthesis or presentation of results. | Y | Y | - | Y |
| Synthesis methods | 13a | Describe the processes used to decide which studies were eligible for each synthesis (e.g. tabulating the study intervention characteristics and comparing against the planned groups for each synthesis (item #5)). | Y | Y | - | Y |
|  | 13b | Describe any methods required to prepare the data for presentation or synthesis, such as handling of missing summary statistics, or data conversions. | Y | Y | - | Y |
|  | 13c | Describe any methods used to tabulate or visually display results of individual studies and syntheses. | Y | Y | - | Y |
|  | 13d | Describe any methods used to synthesise results and provide a rationale for the choice(s). If meta-analysis was performed, describe the model(s), method(s) to identify the presence and extent of statistical heterogeneity, and software package(s) used. | Y | Y | - | Y |
|  | 13e | Describe any methods used to explore possible causes of heterogeneity among study results (e.g. subgroup analysis, meta regression). | N | PY | N | N |
|  | 13f | Describe any sensitivity analyses conducted to assess robustness of the synthesised results. | N | PY | N | N |
| Reporting bias  assessment | 14 | Describe any methods used to assess risk of bias due to missing results in a synthesis (arising from reporting biases). | Y | PY | Y | Y |
| Certainty assessment | 15 | Describe any methods used to assess certainty (or confidence) in the body of evidence for an outcome. | N | N | - | N |
| **Results** | | | | | | |
| Study selection | 16a | Describe the results of the search and selection process, from the number of records identified in the search to the number of studies included in the review, ideally using a flow diagram (see fig 1). | Y | Y | - | Y |
|  | 16b | Cite studies that might appear to meet the inclusion criteria, but which were excluded, and explain why they were excluded. | N | PY | PY | PY |
| Study characteristics | 17 | Cite each included study and present its characteristics. | Y | Y | - | Y |
| Risk of bias in studies | 18 | Present assessments of risk of bias for each included study. | PY | Y | N | N |
| Results of individual  studies | 19 | For all outcomes, present, for each study: (a) summary statistics for each group (where appropriate) and (b) an effect estimate and its precision (e.g. confidence/credible interval), ideally using structured tables or plots. | Y | Y | - | Y |
| Results of syntheses | 20a | For each synthesis, briefly summarise the characteristics and risk of bias among contributing studies. | Y | Y | - | Y |
|  | 20b | Present results of all statistical syntheses conducted. If meta-analysis was done, present for each the summary estimate and its precision (e.g. confidence/credible interval) and measures of statistical heterogeneity. If comparing groups, describe the direction of the effect. | Y | Y | - | Y |
|  | 20c | Present results of all investigations of possible causes of heterogeneity among study results. | N | PY | N | N |
|  | 20d | Present results of all sensitivity analyses conducted to assess the robustness of the synthesised results. | N | PY | N | N |
| Reporting biases | 21 | Present assessments of risk of bias due to missing results (arising from reporting biases) for each synthesis assessed. | Y | PY | PY | PY |
| Certainty of evidence | 22 | Present assessments of certainty (or confidence) in the body of evidence for each outcome assessed. | N | N | - | N |
| **Discussion** | | | | | | |
| Discussion | 23a | Provide a general interpretation of the results in the context of other evidence. | Y | Y | - | Y |
|  | 23b | Discuss any limitations of the evidence included in the review. | Y | Y | - | Y |
|  | 23c | Discuss any limitations of the review processes used. | Y | Y | - | Y |
|  | 23d | Discuss implications of the results for practice, policy, and future research. | Y | Y | - | Y |
| **Other information** | | | | | | |
| Registration and  protocol | 24a | Provide registration information for the review, including register name and registration number, or state that the review was not registered. | N | N | - | N |
|  | 24b | Indicate where the review protocol can be accessed, or state that a protocol was not prepared. | N | N | - | N |
|  | 24c | Describe and explain any amendments to information provided at registration or in the protocol. | N | N | - | N |
| Support | 25 | Describe sources of financial or non-financial support for the review, and the role of the funders or sponsors in the review. | Y | Y | - | Y |
| Competing interests | 26 | Declare any competing interests of review authors. | Y | Y | - | Y |
| Availability of data,  code, and other  materials | 27 | Report which of the following are publicly available and where they can be found: template data collection forms; data extracted from included studies; data used for all analyses; analytic code; any other materials used in the review. | PY | PY | - | PY |

PRISMA 2020

Y: Yes; N: No; PY: Partial Yes.

A:The conclusions of researcher Ph.D.Yongxiu Liu.

B:The conclusions of researcher Ph.D. Yuguo Li.

C:In case of a difference of opinions, it shall be adjudicated by Professor Lei Gao.

D:Conclusive conclusion.

Study 42：杨玉丰,张慧中. 维生素D联合丙酸氟替卡松治疗儿童哮喘有效性的Meta分析[J]. 今日健康,2025(12):105-108. DOI:10.3969/j.issn.1671-5160.2025.12.044.

| Section and topic | Item # | Checklist item | A | B | C | D |
| --- | --- | --- | --- | --- | --- | --- |
| **Title** | | | | | | |
| Title | 1 | Identify the report as a systematic review. | PY | Y | Y | Y |
| **Abstract** | | | | | | |
| Abstract | 2 | See the PRISMA 2020 for Abstracts checklist (table 2). | PY | PY | - | PY |
| **Introduction** | | | | | | |
| Rationale | 3 | Describe the rationale for the review in the context of existing knowledge | Y | Y | - | Y |
| Objectives | 4 | Provide an explicit statement of the objective(s) or question(s) the review addresses. | Y | Y | - | Y |
| **Methods** | | | | | | |
| Eligibility criteria | 5 | Specify the inclusion and exclusion criteria for the review and how studies were grouped for the syntheses. | Y | Y | - | Y |
| Information sources | 6 | Specify all databases, registers, websites, organisations, reference lists and other sources searched or consulted to identify studies. Specify the date when each source was last searched or consulted. | PY | PY | - | PY |
| Search strategy | 7 | Present the full search strategies for all databases, registers and websites, including any filters and limits used | PY | PY | - | PY |
| Selection process | 8 | Specify the methods used to decide whether a study met the inclusion criteria of the review, including how many reviewers screened each record and each report retrieved, whether they worked independently, and if applicable, details of automation tools  used in the process. | Y | Y | - | Y |
| Data collection  process | 9 | Specify the methods used to collect data from reports, including how many reviewers collected data from each report, whether they worked independently, any processes for obtaining or confirming data from study investigators, and if applicable, details of automation tools used in the process. | Y | Y | - | Y |
| Data items | 10a | List and define all outcomes for which data were sought. Specify whether all results that were compatible with each outcome domain in each study were sought (e.g. for all measures, time points, analyses), and if not, the methods used to decide which  results to collect. | Y | Y | - | Y |
|  | 10b | List and define all other variables for which data were sought (e.g. participant and intervention characteristics, funding sources). Describe any assumptions made about any missing or unclear information. | PY | PY | - | PY |
| Study risk of bias  assessment | 11 | Specify the methods used to assess risk of bias in the included studies, including details of the tool(s) used, how many reviewers assessed each study and whether they worked independently, and if applicable, details of automation tools used in the process. | Y | Y | - | Y |
| Effect measures | 12 | Specify for each outcome the effect measure(s) (e.g. risk ratio, mean difference) used in the synthesis or presentation of results. | Y | Y | - | Y |
| Synthesis methods | 13a | Describe the processes used to decide which studies were eligible for each synthesis (e.g. tabulating the study intervention characteristics and comparing against the planned groups for each synthesis (item #5)). | Y | Y | - | Y |
|  | 13b | Describe any methods required to prepare the data for presentation or synthesis, such as handling of missing summary statistics, or data conversions. | Y | Y | - | Y |
|  | 13c | Describe any methods used to tabulate or visually display results of individual studies and syntheses. | Y | Y | - | Y |
|  | 13d | Describe any methods used to synthesise results and provide a rationale for the choice(s). If meta-analysis was performed, describe the model(s), method(s) to identify the presence and extent of statistical heterogeneity, and software package(s) used. | Y | Y | - | Y |
|  | 13e | Describe any methods used to explore possible causes of heterogeneity among study results (e.g. subgroup analysis, meta regression). | N | N | - | N |
|  | 13f | Describe any sensitivity analyses conducted to assess robustness of the synthesised results. | N | N | - | N |
| Reporting bias  assessment | 14 | Describe any methods used to assess risk of bias due to missing results in a synthesis (arising from reporting biases). | N | Y | N | Y |
| Certainty assessment | 15 | Describe any methods used to assess certainty (or confidence) in the body of evidence for an outcome. | N | Y | N | Y |
| **Results** | | | | | | |
| Study selection | 16a | Describe the results of the search and selection process, from the number of records identified in the search to the number of studies included in the review, ideally using a flow diagram (see fig 1). | PY | Y | PY | PY |
|  | 16b | Cite studies that might appear to meet the inclusion criteria, but which were excluded, and explain why they were excluded. | N | PY | PY | PY |
| Study characteristics | 17 | Cite each included study and present its characteristics. | PY | Y | PY | PY |
| Risk of bias in studies | 18 | Present assessments of risk of bias for each included study. | PY | Y | Y | Y |
| Results of individual  studies | 19 | For all outcomes, present, for each study: (a) summary statistics for each group (where appropriate) and (b) an effect estimate and its precision (e.g. confidence/credible interval), ideally using structured tables or plots. | Y | Y | - | Y |
| Results of syntheses | 20a | For each synthesis, briefly summarise the characteristics and risk of bias among contributing studies. | Y | Y | - | Y |
|  | 20b | Present results of all statistical syntheses conducted. If meta-analysis was done, present for each the summary estimate and its precision (e.g. confidence/credible interval) and measures of statistical heterogeneity. If comparing groups, describe the direction of the effect. | Y | Y | - | Y |
|  | 20c | Present results of all investigations of possible causes of heterogeneity among study results. | N | N | - | N |
|  | 20d | Present results of all sensitivity analyses conducted to assess the robustness of the synthesised results. | N | N | - | N |
| Reporting biases | 21 | Present assessments of risk of bias due to missing results (arising from reporting biases) for each synthesis assessed. | N | N | - | N |
| Certainty of evidence | 22 | Present assessments of certainty (or confidence) in the body of evidence for each outcome assessed. | N | N | - | N |
| **Discussion** | | | | | | |
| Discussion | 23a | Provide a general interpretation of the results in the context of other evidence. | Y | Y | - | Y |
|  | 23b | Discuss any limitations of the evidence included in the review. | Y | Y | - | Y |
|  | 23c | Discuss any limitations of the review processes used. | Y | Y | - | Y |
|  | 23d | Discuss implications of the results for practice, policy, and future research. | Y | Y | - | Y |
| **Other information** | | | | | | |
| Registration and  protocol | 24a | Provide registration information for the review, including register name and registration number, or state that the review was not registered. | N | N | - | N |
|  | 24b | Indicate where the review protocol can be accessed, or state that a protocol was not prepared. | N | N | - | N |
|  | 24c | Describe and explain any amendments to information provided at registration or in the protocol. | N | N | - | N |
| Support | 25 | Describe sources of financial or non-financial support for the review, and the role of the funders or sponsors in the review. | N | Y | N | N |
| Competing interests | 26 | Declare any competing interests of review authors. | N | Y | N | N |
| Availability of data,  code, and other  materials | 27 | Report which of the following are publicly available and where they can be found: template data collection forms; data extracted from included studies; data used for all analyses; analytic code; any other materials used in the review. | N | PY | N | N |

5.GRADE

A:The conclusions of researcher Ph.D.Yongxiu Liu.

B:The conclusions of researcher Ph.D. Yuguo Li.

C:In case of a difference of opinions, it shall be adjudicated by Professor Lei Gao.

D:Conclusive conclusion.

①Methodological quality of included studies was low, with biases in randomization, allocation concealment, and blinding. ②The heterogeneity was large and low confidence interval overlap. ③The population was not broadly representative. ④Small sample size, 95% confidence intervals include null values. ⑤Few studies were included, the funnel plot was not symmetrical, Egger’s test found that publication bias or results were positive, and there was no publication bias evaluation.

Study 22：Fares, Munes M et al. “Vitamin D supplementation in children with asthma: a systematic review and meta-analysis.” BMC research notes vol. 8 23. 3 Feb. 2015, doi:10.1186/s13104-014-0961-3.PMID: 25643669 PMCID: PMC4328422

| Endpoint measure | Downgrading factor | A | B | C | D |
| --- | --- | --- | --- | --- | --- |
| FEV1 (Forced Expiratory Volume in 1 second) | Risk of bias | -1^①^ | -1^①^ | - | -1^①^ |
|  | Inconsistency | 0 | -1^②^ | 0 | 0 |
|  | Indirectness | 0 | 0 | - | 0 |
|  | Impression | -1^④^ | -1^④^ | - | -1^④^ |
|  | Publication bias | 0 | 0 | - | 0 |
| Vitamin D levels (serum 25(OH)D) | Risk of bias | -1^①^ | -1^①^ | - | -1^①^ |
|  | Inconsistency | -1^②^ | -1^②^ | - | -1^②^ |
|  | Indirectness | 0 | 0 | - | 0 |
|  | Impression | -1^④^ | -1^④^ | - | -1^④^ |
|  | Publication bias | 0 | 0 | - | 0 |

GRADE

A:The conclusions of researcher Ph.D.Yongxiu Liu.

B:The conclusions of researcher Ph.D. Yuguo Li.

C:In case of a difference of opinions, it shall be adjudicated by Professor Lei Gao.

D:Conclusive conclusion.

①Methodological quality of included studies was low, with biases in randomization, allocation concealment, and blinding. ②The heterogeneity was large and low confidence interval overlap. ③The population was not broadly representative. ④Small sample size, 95% confidence intervals include null values. ⑤Few studies were included, the funnel plot was not symmetrical, Egger’s test found that publication bias or results were positive, and there was no publication bias evaluation.

Study 23：Luo, Jian et al. “Can Vitamin D Supplementation in Addition to Asthma Controllers Improve Clinical Outcomes in Patients With Asthma?: A Meta-Analysis.” Medicine vol. 94,50 (2015): e2185. doi:10.1097/MD.0000000000002185.PMID: 26683927 PMCID: PMC5058899

| Endpoint measure | Downgrading factor | A | B | C | D |
| --- | --- | --- | --- | --- | --- |
| Asthma exacerbations | Risk of bias | -1^①^ | 0 | -1^①^ | -1^①^ |
|  | Inconsistency | -1^②^ | -1^②^ | - | -1^②^ |
|  | Indirectness | 0 | 0 | - | 0 |
|  | Impression | -1^④^ | -1^④^ | - | -1^④^ |
|  | Publication bias | 0 | 0 | - | 0 |
| FEV₁ (% of predicted value) | Risk of bias | 0 | -1^①^ | -1^①^ | -1^①^ |
|  | Inconsistency | -1^②^ | -1^②^ | - | -1^②^ |
|  | Indirectness | 0 | 0 | - | 0 |
|  | Impression | -1^④^ | -1^④^ | - | -1^④^ |
|  | Publication bias | 0 | 0 | - | 0 |
| FeNO (Fraction of exhaled nitric oxide) | Risk of bias | -1^①^ | 0 |  | -1^①^ |
|  | Inconsistency | 0 | 0 | - | 0 |
|  | Indirectness | 0 | 0 | - | 0 |
|  | Impression | -1^④^ | -1^④^ | - | -1^④^ |
|  | Publication bias | 0 | 0 | - | 0 |
| ACT (Asthma Control Test) | Risk of bias | -1^①^ | 0 | -1^①^ | -1^①^ |
|  | Inconsistency | 0 | 0 | - | 0 |
|  | Indirectness | 0 | 0 | - | 0 |
|  | Impression | -1^④^ | -1^④^ | - | -1^④^ |
|  | Publication bias | 0 | 0 | - | 0 |
| Serum 25-hydroxyvitamin D levels | Risk of bias | -1^①^ | 0 |  | -1^①^ |
|  | Inconsistency | 0 | 0 | - | 0 |
|  | Indirectness | 0 | 0 | - | 0 |
|  | Impression | 0 | 0 | - | 0 |
|  | Publication bias | 0 | 0 | - | 0 |
| Adverse events | Risk of bias | -1^①^ | 0 | -1^①^ | -1^①^ |
|  | Inconsistency | 0 | 0 | - | 0 |
|  | Indirectness | 0 | 0 | - | 0 |
|  | Impression | -1^④^ | -1^④^ | - | -1^④^ |
|  | Publication bias | 0 | 0 | - | 0 |

GRADE

A:The conclusions of researcher Ph.D.Yongxiu Liu.

B:The conclusions of researcher Ph.D. Yuguo Li.

C:In case of a difference of opinions, it shall be adjudicated by Professor Lei Gao.

D:Conclusive conclusion.

①Methodological quality of included studies was low, with biases in randomization, allocation concealment, and blinding. ②The heterogeneity was large and low confidence interval overlap. ③The population was not broadly representative. ④Small sample size, 95% confidence intervals include null values. ⑤Few studies were included, the funnel plot was not symmetrical, Egger’s test found that publication bias or results were positive, and there was no publication bias evaluation.

Study 24：Pojsupap, Supichaya et al. “Efficacy of high-dose vitamin D in pediatric asthma: a systematic review and meta-analysis.” The Journal of asthma : official journal of the Association for the Care of Asthma vol. 52,4 (2015): 382-90. doi:10.3109/02770903.2014.980509.PMID: 25365192

| Endpoint measure | Downgrading factor | A | B | C | D |
| --- | --- | --- | --- | --- | --- |
| Asthma exacerbations | Risk of bias | -1^①^ | 0 | -1^①^ | -1^①^ |
|  | Inconsistency | 0 | 0 | - | 0 |
|  | Indirectness | 0 | 0 | - | 0 |
|  | Impression | 0 | -1^④^ | 0 | 0 |
|  | Publication bias | 0 | 0 | - | 0 |

GRADE

A:The conclusions of researcher Ph.D.Yongxiu Liu.

B:The conclusions of researcher Ph.D. Yuguo Li.

C:In case of a difference of opinions, it shall be adjudicated by Professor Lei Gao.

D:Conclusive conclusion.

①Methodological quality of included studies was low, with biases in randomization, allocation concealment, and blinding. ②The heterogeneity was large and low confidence interval overlap. ③The population was not broadly representative. ④Small sample size, 95% confidence intervals include null values. ⑤Few studies were included, the funnel plot was not symmetrical, Egger’s test found that publication bias or results were positive, and there was no publication bias evaluation.

Study 25：Riverin, Bruno D et al. “Vitamin D Supplementation for Childhood Asthma: A Systematic Review and Meta-Analysis.” PloS one vol. 10,8 e0136841. 31 Aug. 2015, doi:10.1371/journal.pone.0136841.PMID: 26322509 PMCID: PMC4556456

| Endpoint measure | Downgrading factor | A | B | C | D |
| --- | --- | --- | --- | --- | --- |
| Asthma exacerbations | Risk of bias | -1^①^ | -1^①^ | - | -1^①^ |
|  | Inconsistency | 0 | 0 | - | 0 |
|  | Indirectness | 0 | 0 | - | 0 |
|  | Impression | -1^④^ | -1^④^ | - | -1^④^ |
|  | Publication bias | 0 | 0 | - | 0 |
| Serum 25-hydroxyvitamin D concentration | Risk of bias | -1^①^ | -1^①^ | - | -1^①^ |
|  | Inconsistency | -1^②^ | -1^②^ | - | -1^②^ |
|  | Indirectness | 0 | 0 | - | 0 |
|  | Impression | 0 | -1^④^ | 0 | 0 |
|  | Publication bias | 0 | 0 | - | 0 |
| Lung function (FEV1) | Risk of bias | -1^①^ | -1^①^ | - | -1^①^ |
|  | Inconsistency | 0 | 0 | - | 0 |
|  | Indirectness | 0 | 0 | - | 0 |
|  | Impression | -1^④^ | -1^④^ | - | -1^④^ |
|  | Publication bias | 0 | 0 | - | 0 |

GRADE

A:The conclusions of researcher Ph.D.Yongxiu Liu.

B:The conclusions of researcher Ph.D. Yuguo Li.

C:In case of a difference of opinions, it shall be adjudicated by Professor Lei Gao.

D:Conclusive conclusion.

①Methodological quality of included studies was low, with biases in randomization, allocation concealment, and blinding. ②The heterogeneity was large and low confidence interval overlap. ③The population was not broadly representative. ④Small sample size, 95% confidence intervals include null values. ⑤Few studies were included, the funnel plot was not symmetrical, Egger’s test found that publication bias or results were positive, and there was no publication bias evaluation.

Study 26：景伟超,刘璐佳,关洋洋,等.维生素D辅助治疗儿童哮喘Meta分析[J].世界中西医结合杂志,2017,12(10):1341-1344+1354.DOI:10.13935/j.cnki.sjzx.171003.

| Endpoint measure | Downgrading factor | A | B | C | D |
| --- | --- | --- | --- | --- | --- |
| Total effective rate | Risk of bias | -1^①^ | -1^①^ | - | -1^①^ |
|  | Inconsistency | 0 | 0 | - | 0 |
|  | Indirectness | 0 | 0 | - | 0 |
|  | Impression | 0 | -1^④^ | -1^④^ | -1^④^ |
|  | Publication bias | -1^⑤^ | -1^⑤^ | - | -1^⑤^ |
| Asthma Control Test (ACT) score | Risk of bias | -1^①^ | -1^①^ | 0 | -1^①^ |
|  | Inconsistency | -1^②^ | -1^②^ | - | -1^②^ |
|  | Indirectness | 0 | 0 | - | 0 |
|  | Impression | -1^④^ | -1^④^ | - | -1^④^ |
|  | Publication bias | 0 | 0 | - | 0 |
| Frequency of asthma attacks within one year | Risk of bias | -1^①^ | -1^①^ | - | -1^①^ |
|  | Inconsistency | -1^②^ | -1^②^ | - | -1^②^ |
|  | Indirectness | 0 | 0 | - | 0 |
|  | Impression | -1^④^ | -1^④^ | - | -1^④^ |
|  | Publication bias | 0 | 0 | - | 0 |
| Serum 25-hydroxyvitamin D₃ (25-(OH)D₃) level | Risk of bias | -1^①^ | -1^①^ | - | -1^①^ |
|  | Inconsistency | 0 | 0 | - | 0 |
|  | Indirectness | 0 | 0 | - | 0 |
|  | Impression | 0 | -1^④^ | 0 | 0 |
|  | Publication bias | 0 | 0 | - | 0 |

GRADE

A:The conclusions of researcher Ph.D.Yongxiu Liu.

B:The conclusions of researcher Ph.D. Yuguo Li.

C:In case of a difference of opinions, it shall be adjudicated by Professor Lei Gao.

D:Conclusive conclusion.

①Methodological quality of included studies was low, with biases in randomization, allocation concealment, and blinding. ②The heterogeneity was large and low confidence interval overlap. ③The population was not broadly representative. ④Small sample size, 95% confidence intervals include null values. ⑤Few studies were included, the funnel plot was not symmetrical, Egger’s test found that publication bias or results were positive, and there was no publication bias evaluation.

Study 27：Jolliffe, David A et al. “Vitamin D supplementation to prevent asthma exacerbations: a systematic review and meta-analysis of individual participant data.” The Lancet. Respiratory medicine vol. 5,11 (2017): 881-890. doi:10.1016/S2213-2600(17)30306-5.PMID: 28986128 PMCID: PMC5693329

| Endpoint measure | Downgrading factor | A | B | C | D |
| --- | --- | --- | --- | --- | --- |
| Incidence of asthma exacerbation requiring treatment with systemic corticosteroids | Risk of bias | 0 | 0 | - | 0 |
|  | Inconsistency | 0 | 0 | - | 0 |
|  | Indirectness | 0 | 0 | - | 0 |
|  | Impression | 0 | 0 | - | 0 |
|  | Publication bias | 0 | 0 | - | 0 |
| Incidence of asthma exacerbations resulting in emergency department attendance or hospital admission, or both | Risk of bias | 0 | 0 | 0 | 0 |
|  | Inconsistency | 0 | 0 | 0 | 0 |
|  | Indirectness | 0 | 0 | 0 | 0 |
|  | Impression | -1^④^ | -1^④^ | -1^④^ | -1^④^ |
|  | Publication bias | 0 | 0 | 0 | 0 |
| Proportion of participants with at least one asthma exacerbation requiring treatment with systemic corticosteroids | Risk of bias | 0 | 0 | 0 | 0 |
|  | Inconsistency | 0 | 0 | - | 0 |
|  | Indirectness | 0 | 0 | - | 0 |
|  | Impression | -1^④^ | -1^④^ | -1^④^ | -1^④^ |
|  | Publication bias | 0 | 0 | - | 0 |
| Time to first asthma exacerbation requiring treatment with systemic corticosteroids | Risk of bias | 0 | 0 | - | 0 |
|  | Inconsistency | 0 | 0 | - | 0 |
|  | Indirectness | 0 | 0 | - | 0 |
|  | Impression | -1^④^ | -1^④^ | - | -1^④^ |
|  | Publication bias | 0 | 0 | - | 0 |
| Incidence of serious adverse events of any cause | Risk of bias | 0 | 0 | - | 0 |
|  | Inconsistency | 0 | 0 | - | 0 |
|  | Indirectness | 0 | 0 | - | 0 |
|  | Impression | -1^④^ | -1^④^ | - | -1^④^ |
|  | Publication bias | 0 | 0 | - | 0 |

GRADE

A:The conclusions of researcher Ph.D.Yongxiu Liu.

B:The conclusions of researcher Ph.D. Yuguo Li.

C:In case of a difference of opinions, it shall be adjudicated by Professor Lei Gao.

D:Conclusive conclusion.

①Methodological quality of included studies was low, with biases in randomization, allocation concealment, and blinding. ②The heterogeneity was large and low confidence interval overlap. ③The population was not broadly representative. ④Small sample size, 95% confidence intervals include null values. ⑤Few studies were included, the funnel plot was not symmetrical, Egger’s test found that publication bias or results were positive, and there was no publication bias evaluation.

Study 28：郝宏霞.维生素D在缓解期哮喘患者治疗中的有效性和安全性meta分析[D].山西医科大学,2018.

| Endpoint measure | Downgrading factor | A | B | C | D |
| --- | --- | --- | --- | --- | --- |
| Number of acute exacerbations of asthma | Risk of bias | -1^①^ | -1^①^ | - | -1^①^ |
|  | Inconsistency | -1^②^ | -1^②^ | - | -1^②^ |
|  | Indirectness | 0 | 0 | - | 0 |
|  | Impression | -1^④^ | -1^④^ | - | -1^④^ |
|  | Publication bias | 0 | 0 | - | 0 |
| Acute exacerbation of asthma requiring systemic corticosteroids therapy | Risk of bias | -1^①^ | -1^①^ | - | -1^①^ |
|  | Inconsistency | 0 | 0 | - | 0 |
|  | Indirectness | 0 | 0 | - | 0 |
|  | Impression | 0 | 0 | - | 0 |
|  | Publication bias | 0 | 0 | - | 0 |
| Asthma exacerbations requiring ED visit or hospitalisation or both | Risk of bias | -1^①^ | -1^①^ | - | -1^①^ |
|  | Inconsistency | 0 | 0 | - | 0 |
|  | Indirectness | 0 | 0 | - | 0 |
|  | Impression | 0 | 0 | - | 0 |
|  | Publication bias | 0 | 0 | - | 0 |
| FEV₁ (% of predicted value) | Risk of bias | -1^①^ | -1^①^ | - | -1^①^ |
|  | Inconsistency | 0 | 0 | - | 0 |
|  | Indirectness | 0 | 0 | - | 0 |
|  | Impression | 0 | 0 | - | 0 |
|  | Publication bias | 0 | 0 | - | 0 |
| Serum 25-hydroxyvitamin D levels | Risk of bias | -1^①^ | -1^①^ | - | -1^①^ |
|  | Inconsistency | -1^②^ | -1^②^ | - | -1^②^ |
|  | Indirectness | 0 | 0 | - | 0 |
|  | Impression | 0 | 0 | - | 0 |
|  | Publication bias | 0 | 0 | - | 0 |
| Adverse events | Risk of bias | -1^①^ | -1^①^ | - | -1^①^ |
|  | Inconsistency | 0 | 0 | - | 0 |
|  | Indirectness | 0 | 0 | - | 0 |
|  | Impression | -1^④^ | -1^④^ | - | -1^④^ |
|  | Publication bias | 0 | 0 | - | 0 |
| Fatal asthma exacerbation | Risk of bias | -1^①^ | -1^①^ | - | -1^①^ |
|  | Inconsistency | 0 | 0 | - | 0 |
|  | Indirectness | 0 | 0 | - | 0 |
|  | Impression | -1^④^ | -1^④^ | - | -1^④^ |
|  | Publication bias | 0 | 0 | - | 0 |

GRADE

A:The conclusions of researcher Ph.D.Yongxiu Liu.

B:The conclusions of researcher Ph.D. Yuguo Li.

C:In case of a difference of opinions, it shall be adjudicated by Professor Lei Gao.

D:Conclusive conclusion.

①Methodological quality of included studies was low, with biases in randomization, allocation concealment, and blinding. ②The heterogeneity was large and low confidence interval overlap. ③The population was not broadly representative. ④Small sample size, 95% confidence intervals include null values. ⑤Few studies were included, the funnel plot was not symmetrical, Egger’s test found that publication bias or results were positive, and there was no publication bias evaluation.

Study 29：田超,史强,赵紫楠,等.维生素D补充剂对儿童支气管哮喘获益相关性的系统评价和meta分析[J].临床药物治疗杂志,2018,16(04):66-70.

| Endpoint measure | Downgrading factor | A | B | C | D |
| --- | --- | --- | --- | --- | --- |
| Forced expiratory volume in 1 second, % predicted (FEV₁% predicted) | Risk of bias | -1^①^ | -1^①^ | - | -1^①^ |
|  | Inconsistency | -1^②^ | -1^②^ | - | -1^②^ |
|  | Indirectness | 0 | 0 | - | 0 |
|  | Impression | -1^④^ | -1^④^ | - | -1^④^ |
|  | Publication bias | 0 | 0 | - | 0 |
| Number of asthma acute exacerbations | Risk of bias | 0 | -1^①^ | -1^①^ | -1^①^ |
|  | Inconsistency | -1^②^ | 0 | -1^②^ | -1^②^ |
|  | Indirectness | 0 | 0 | - | 0 |
|  | Impression | -1^④^ | -1^④^ | - | -1^④^ |
|  | Publication bias | 0 | 0 | - | 0 |

GRADE

A:The conclusions of researcher Ph.D.Yongxiu Liu.

B:The conclusions of researcher Ph.D. Yuguo Li.

C:In case of a difference of opinions, it shall be adjudicated by Professor Lei Gao.

D:Conclusive conclusion.

①Methodological quality of included studies was low, with biases in randomization, allocation concealment, and blinding. ②The heterogeneity was large and low confidence interval overlap. ③The population was not broadly representative. ④Small sample size, 95% confidence intervals include null values. ⑤Few studies were included, the funnel plot was not symmetrical, Egger’s test found that publication bias or results were positive, and there was no publication bias evaluation.

Study 30：郝畅.维生素D补充与儿童哮喘的系统评价及meta分析[D].重庆医科大学,2019.

| Endpoint measure | Downgrading factor | A | B | C | D |
| --- | --- | --- | --- | --- | --- |
| Asthmatic attacks | Risk of bias | -1^①^ | -1^①^ | - | -1^①^ |
|  | Inconsistency | 0 | 0 | - | 0 |
|  | Indirectness | 0 | 0 | - | 0 |
|  | Impression | 0 | 0 | - | 0 |
|  | Publication bias | 0 | 0 | - | 0 |
| Serum 25-hydroxyvitamin D level | Risk of bias | -1^①^ | -1^①^ | - | -1^①^ |
|  | Inconsistency | -1^②^ | -1^②^ | - | -1^②^ |
|  | Indirectness | 0 | 0 | - | 0 |
|  | Impression | -1^④^ | -1^④^ | - | -1^④^ |
|  | Publication bias | 0 | 0 | - | 0 |
| Forced Expiratory Volume in 1 second (% predicted) | Risk of bias | -1^①^ | -1^①^ | - | -1^①^ |
|  | Inconsistency | -1^②^ | -1^②^ | - | -1^②^ |
|  | Indirectness | 0 | 0 | - | 0 |
|  | Impression | -1^④^ | -1^④^ | - | -1^④^ |
|  | Publication bias | 0 | 0 | - | 0 |
| Forced Expiratory Volume in 1 second | Risk of bias | -1^①^ | -1^①^ | - | -1^①^ |
|  | Inconsistency | -1^②^ | -1^②^ | - | -1^②^ |
|  | Indirectness | 0 | 0 | - | 0 |
|  | Impression | -1^④^ | -1^④^ | - | -1^④^ |
|  | Publication bias | 0 | 0 | - | 0 |

GRADE

A:The conclusions of researcher Ph.D.Yongxiu Liu.

B:The conclusions of researcher Ph.D. Yuguo Li.

C:In case of a difference of opinions, it shall be adjudicated by Professor Lei Gao.

D:Conclusive conclusion.

①Methodological quality of included studies was low, with biases in randomization, allocation concealment, and blinding. ②The heterogeneity was large and low confidence interval overlap. ③The population was not broadly representative. ④Small sample size, 95% confidence intervals include null values. ⑤Few studies were included, the funnel plot was not symmetrical, Egger’s test found that publication bias or results were positive, and there was no publication bias evaluation.

Study 31：Wang, Mingming et al. “Association between vitamin D status and asthma control: A meta-analysis of randomized trials.”Respiratory medicine vol. 150 (2019): 85-94. doi:10.1016/j.rmed.2019.02.016.PMID: 30961957

| Endpoint measure | Downgrading factor | A | B | C | D |
| --- | --- | --- | --- | --- | --- |
| Rate of asthma exacerbation | Risk of bias | -1^①^ | -1^①^ | - | -1^①^ |
|  | Inconsistency | 0 | 0 | - | 0 |
|  | Indirectness | 0 | 0 | - | 0 |
|  | Impression | -1^④^ | -1^④^ | - | -1^④^ |
|  | Publication bias | -1^⑤^ | 0 | -1^⑤^ | -1^⑤^ |
| FEV₁% (predicted percentage of forced expiratory volume in first second) | Risk of bias | -1^①^ | -1^①^ | - | -1^①^ |
|  | Inconsistency | -1^②^ | -1^②^ | - | -1^②^ |
|  | Indirectness | 0 | 0 | - | 0 |
|  | Impression | -1^④^ | -1^④^ | - | -1^④^ |
|  | Publication bias | 0 | 0 | - | 0 |
| ACT scores (asthma control test scores) | Risk of bias | -1^①^ | -1^①^ | - | -1^①^ |
|  | Inconsistency | -1^②^ | -1^②^ | - | -1^②^ |
|  | Indirectness | 0 | 0 | - | 0 |
|  | Impression | -1^④^ | -1^④^ | - | -1^④^ |
|  | Publication bias | 0 | 0 | - | 0 |
| FeNO (fractional exhaled nitric oxide) | Risk of bias | -1^①^ | -1^①^ | - | -1^①^ |
|  | Inconsistency | 0 | 0 | - | 0 |
|  | Indirectness | 0 | 0 | - | 0 |
|  | Impression | -1^④^ | -1^④^ | - | -1^④^ |
|  | Publication bias | 0 | 0 | - | 0 |
| IL-10 (interleukin-10) | Risk of bias | -1^①^ | -1^①^ | - | -1^①^ |
|  | Inconsistency | -1^②^ | -1^②^ | - | -1^②^ |
|  | Indirectness | 0 | -1^③^ | 0 | 0 |
|  | Impression | -1^④^ | -1^④^ | - | -1^④^ |
|  | Publication bias | 0 | 0 | - | 0 |
| Adverse events | Risk of bias | -1^①^ | -1^①^ | - | -1^①^ |
|  | Inconsistency | 0 | 0 | - | 0 |
|  | Indirectness | 0 | 0 | - | 0 |
|  | Impression | -1^④^ | -1^④^ | - | -1^④^ |
|  | Publication bias | 0 | 0 | - | 0 |

GRADE

A:The conclusions of researcher Ph.D.Yongxiu Liu.

B:The conclusions of researcher Ph.D. Yuguo Li.

C:In case of a difference of opinions, it shall be adjudicated by Professor Lei Gao.

D:Conclusive conclusion.

①Methodological quality of included studies was low, with biases in randomization, allocation concealment, and blinding. ②The heterogeneity was large and low confidence interval overlap. ③The population was not broadly representative. ④Small sample size, 95% confidence intervals include null values. ⑤Few studies were included, the funnel plot was not symmetrical, Egger’s test found that publication bias or results were positive, and there was no publication bias evaluation.

Study 32：Chen, Ziyu et al. “Vitamin D can safely reduce asthma exacerbations among corticosteroid-using children and adults with asthma: a systematic review and meta-analysis of randomized controlled trials.” Nutrition research (New York, N.Y.) vol. 92 (2021): 49-61. doi:10.1016/j.nutres.2021.05.010.PMID: 34274554

| Endpoint measure | Downgrading factor | A | B | C | D |
| --- | --- | --- | --- | --- | --- |
| Rate of asthma exacerbation | Risk of bias | -1^①^ | 0 | 0 | 0 |
|  | Inconsistency | 0 | 0 | - | 0 |
|  | Indirectness | 0 | 0 | - | 0 |
|  | Impression | 0 | 0 | - | 0 |
|  | Publication bias | 0 | 0 | - | 0 |
| Serum 25(OH)D levels | Risk of bias | -1^①^ | 0 | 0 | 0 |
|  | Inconsistency | -1^②^ | -1^②^ | - | -1^②^ |
|  | Indirectness | 0 | 0 | - | 0 |
|  | Impression | 0 | 0 | - | 0 |
|  | Publication bias | 0 | 0 | - | 0 |
| FEV₁% (predicted percentage of forced expiratory volume in first second) | Risk of bias | -1^①^ | 0 | 0 | 0 |
|  | Inconsistency | 0 | 0 | - | 0 |
|  | Indirectness | 0 | 0 | - | 0 |
|  | Impression | -1^④^ | -1^④^ | - | -1^④^ |
|  | Publication bias | 0 | 0 | - | 0 |
| ACT scores (asthma control test scores) | Risk of bias | -1^①^ | -1^①^ | - | -1^①^ |
|  | Inconsistency | -1^②^ | -1^②^ | - | -1^②^ |
|  | Indirectness | 0 | 0 | - | 0 |
|  | Impression | -1^④^ | -1^④^ | - | -1^④^ |
|  | Publication bias | 0 | 0 | - | 0 |
| Adverse events | Risk of bias | -1^①^ | 0 | 0 | 0 |
|  | Inconsistency | 0 | 0 | - | 0 |
|  | Indirectness | 0 | 0 | - | 0 |
|  | Impression | -1^④^ | -1^④^ | - | -1^④^ |
|  | Publication bias | 0 | 0 | - | 0 |

GRADE

A:The conclusions of researcher Ph.D.Yongxiu Liu.

B:The conclusions of researcher Ph.D. Yuguo Li.

C:In case of a difference of opinions, it shall be adjudicated by Professor Lei Gao.

D:Conclusive conclusion.

①Methodological quality of included studies was low, with biases in randomization, allocation concealment, and blinding. ②The heterogeneity was large and low confidence interval overlap. ③The population was not broadly representative. ④Small sample size, 95% confidence intervals include null values. ⑤Few studies were included, the funnel plot was not symmetrical, Egger’s test found that publication bias or results were positive, and there was no publication bias evaluation.

Study 33：Hao, Meiqi et al. “The Effect of Vitamin D Supplementation in Children With Asthma: A Meta-Analysis.”Frontiers in pediatrics vol. 10 840617. 29 Jun. 2022, doi:10.3389/fped.2022.840617.PMID: 35844729 PMCID: PMC9277022

| Endpoint measure | Downgrading factor | A | B | C | D |
| --- | --- | --- | --- | --- | --- |
| Serum Vitamin D Levels | Risk of bias | 0 | -1^①^ | -1^①^ | -1^①^ |
|  | Inconsistency | 0 | -1^②^ | 0 | 0 |
|  | Indirectness | 0 | 0 | - | 0 |
|  | Impression | 0 | -1^④^ | -1^④^ | -1^④^ |
|  | Publication bias | 0 | 0 | - | 0 |
| Childhood Asthma Control Test, CACT Scores | Risk of bias | 0 | -1^①^ | 0 | 0 |
|  | Inconsistency | 0 | 0 | - | 0 |
|  | Indirectness | 0 | 0 | - | 0 |
|  | Impression | -1^④^ | -1^④^ | - | -1^④^ |
|  | Publication bias | 0 | 0 | - | 0 |
| Asthma Exacerbation | Risk of bias | -1^①^ | -1^①^ | - | -1^①^ |
|  | Inconsistency | 0 | 0 | - | 0 |
|  | Indirectness | 0 | -1^③^ | -1^③^ | -1^③^ |
|  | Impression | -1^④^ | -1^④^ | - | -1^④^ |
|  | Publication bias | 0 | 0 | - | 0 |
| Hospitalizations for Asthma Exacerbation | Risk of bias | 0 | -1^①^ | -1^①^ | -1^①^ |
|  | Inconsistency | 0 | 0 | - | 0 |
|  | Indirectness | 0 | 0 | - | 0 |
|  | Impression | -1^④^ | -1^④^ | -1^④^ | -1^④^ |
|  | Publication bias | 0 | 0 | - | 0 |
| Acute Care Visits | Risk of bias | 0 | -1^①^ | -1^①^ | -1^①^ |
|  | Inconsistency | 0 | 0 | - | 0 |
|  | Indirectness | 0 | 0 | - | 0 |
|  | Impression | -1^④^ | -1^④^ | - | -1^④^ |
|  | Publication bias | 0 | 0 | - | 0 |
| Steroid Use | Risk of bias | 0 | -1^①^ | -1^①^ | -1^①^ |
|  | Inconsistency | -1^②^ | -1^②^ | - | -1^②^ |
|  | Indirectness | 0 | 0 | - | 0 |
|  | Impression | -1^④^ | -1^④^ | - | -1^④^ |
|  | Publication bias | 0 | 0 | - | 0 |
| Fractional Exhaled Nitric Oxide, FeNO | Risk of bias | 0 | -1^①^ | -1^①^ | -1^①^ |
|  | Inconsistency | -1^②^ | -1^②^ | - | -1^②^ |
|  | Indirectness | 0 | 0 | - | 0 |
|  | Impression | -1^④^ | -1^④^ | - | -1^④^ |
|  | Publication bias | 0 | 0 | - | 0 |
| Predicted Percentage of Forced Expiratory Volume in the First Second, FEV1% | Risk of bias | 0 | -1^①^ | -1^①^ | -1^①^ |
|  | Inconsistency | 0 | 0 | - | 0 |
|  | Indirectness | 0 | 0 | - | 0 |
|  | Impression | -1^④^ | -1^④^ | - | -1^④^ |
|  | Publication bias | 0 | 0 | - | 0 |
| Percentage of Predicted Forced Vital Capacity, FVC% | Risk of bias | 0 | -1^①^ | -1^①^ | -1^①^ |
|  | Inconsistency | 0 | 0 | - | 0 |
|  | Indirectness | 0 | 0 | - | 0 |
|  | Impression | -1^④^ | -1^④^ | - | -1^④^ |
|  | Publication bias | 0 | 0 | - | 0 |

GRADE

A:The conclusions of researcher Ph.D.Yongxiu Liu.

B:The conclusions of researcher Ph.D. Yuguo Li.

C:In case of a difference of opinions, it shall be adjudicated by Professor Lei Gao.

D:Conclusive conclusion.

①Methodological quality of included studies was low, with biases in randomization, allocation concealment, and blinding. ②The heterogeneity was large and low confidence interval overlap. ③The population was not broadly representative. ④Small sample size, 95% confidence intervals include null values. ⑤Few studies were included, the funnel plot was not symmetrical, Egger’s test found that publication bias or results were positive, and there was no publication bias evaluation.

Study 34：Kumar, Jogender et al. “Vitamin D supplementation in childhood asthma: a systematic review and meta-analysis of randomised controlled trials.” ERJ open research vol. 8,1 00662-2021. 7 Feb. 2021, doi:10.1183/23120541.00662-2021.PMID: 35141325 PMCID: PMC8819253

| Endpoint measure | Downgrading factor | A | B | C | D |
| --- | --- | --- | --- | --- | --- |
| Asthma exacerbations requiring rescue systemic corticosteroids | Risk of bias | 0 | 0 | - | 0 |
|  | Inconsistency | 0 | 0 | - | 0 |
|  | Indirectness | 0 | 0 | - | 0 |
|  | Impression | -1^④^ | -1^④^ | -1^④^ | -1^④^ |
|  | Publication bias | 0 | 0 | - | 0 |
| Asthma exacerbations of any severity | Risk of bias | -1^①^ | -1^①^ | - | -1^①^ |
|  | Inconsistency | -1^②^ | -1^②^ | - | -1^②^ |
|  | Indirectness | 0 | -1^③^ | -1^③^ | -1^③^ |
|  | Impression | -1^④^ | -1^④^ | - | -1^④^ |
|  | Publication bias | 0 | 0 | - | 0 |
| Number of children requiring emergency/unscheduled visits | Risk of bias | -1^①^ | -1^①^ | - | -1^①^ |
|  | Inconsistency | 0 | 0 | - | 0 |
|  | Indirectness | 0 | 0 | - | 0 |
|  | Impression | -1^④^ | -1^④^ | - | -1^④^ |
|  | Publication bias | 0 | 0 | - | 0 |
| Number of children requiring hospitalisations for asthma exacerbation | Risk of bias | 0 | -1^①^ | 0 | 0 |
|  | Inconsistency | 0 | 0 | - | 0 |
|  | Indirectness | 0 | 0 | - | 0 |
|  | Impression | -1^④^ | -1^④^ | 0 | -1^④^ |
|  | Publication bias | 0 | 0 | - | 0 |
| Number of children with well-controlled asthma | Risk of bias | 0 | -1^①^ | -1^①^ | -1^①^ |
|  | Inconsistency | 0 | 0 | - | 0 |
|  | Indirectness | 0 | 0 | - | 0 |
|  | Impression | -1^④^ | -1^④^ | - | -1^④^ |
|  | Publication bias | 0 | 0 | - | 0 |
| Forced Expiratory Volume in 1 second, FEV1 | Risk of bias | 0 | -1^①^ | 0 | 0 |
|  | Inconsistency | -1^②^ | -1^②^ | - | -1^②^ |
|  | Indirectness | 0 | 0 | - | 0 |
|  | Impression | -1^④^ | -1^④^ | - | -1^④^ |
|  | Publication bias | 0 | 0 | - | 0 |
| Fractional Exhaled Nitric Oxide, FeNO | Risk of bias | -1^①^ | -1^①^ | - | -1^①^ |
|  | Inconsistency | -1^②^ | -1^②^ | - | -1^②^ |
|  | Indirectness | 0 | 0 | - | 0 |
|  | Impression | -1^④^ | -1^④^ | - | -1^④^ |
|  | Publication bias | 0 | -1^⑤^ | 0 | 0 |
| Vitamin D levels post-intervention | Risk of bias | 0 | 0 | - | 0 |
|  | Inconsistency | -1^②^ | -1^②^ | - | -1^②^ |
|  | Indirectness | 0 | 0 | - | 0 |
|  | Impression | 0 | 0 | - | 0 |
|  | Publication bias | 0 | 0 | - | 0 |
| Number of children with serious adverse events | Risk of bias | 0 | -1^①^ | -1^①^ | -1^①^ |
|  | Inconsistency | 0 | 0 | - | 0 |
|  | Indirectness | 0 | 0 | - | 0 |
|  | Impression | -1^④^ | -1^④^ | - | -1^④^ |
|  | Publication bias | 0 | 0 | - | 0 |

GRADE

A:The conclusions of researcher Ph.D.Yongxiu Liu.

B:The conclusions of researcher Ph.D. Yuguo Li.

C:In case of a difference of opinions, it shall be adjudicated by Professor Lei Gao.

D:Conclusive conclusion.

①Methodological quality of included studies was low, with biases in randomization, allocation concealment, and blinding. ②The heterogeneity was large and low confidence interval overlap. ③The population was not broadly representative. ④Small sample size, 95% confidence intervals include null values. ⑤Few studies were included, the funnel plot was not symmetrical, Egger’s test found that publication bias or results were positive, and there was no publication bias evaluation.

Study 35：Liu, Meiqi et al. “A Meta-Analysis on Vitamin D Supplementation and Asthma Treatment.” Frontiers in nutrition vol. 9 860628. 6 Jul. 2022, doi:10.3389/fnut.2022.860628.PMID: 35873428 PMCID: PMC9300755

| Endpoint measure | Downgrading factor | A | B | C | D |
| --- | --- | --- | --- | --- | --- |
| Rate of asthma exacerbations | Risk of bias | -1^①^ | -1^①^ | - | -1^①^ |
|  | Inconsistency | -1^②^ | -1^②^ | - | -1^②^ |
|  | Indirectness | 0 | -1^③^ | -1^③^ | -1^③^ |
|  | Impression | 0 | 0 | - | 0 |
|  | Publication bias | -1^⑤^ | 0 | 0 | 0 |
| Forced Expiratory Volume in 1 second (FEV₁) | Risk of bias | -1^①^ | -1^①^ | - | -1^①^ |
|  | Inconsistency | -1^②^ | -1^②^ | - | -1^②^ |
|  | Indirectness | 0 | 0 | - | 0 |
|  | Impression | -1^④^ | -1^④^ | - | -1^④^ |
|  | Publication bias | 0 | 0 | - | 0 |
| Asthma Control Test (ACT) score | Risk of bias | -1^①^ | -1^①^ | - | -1^①^ |
|  | Inconsistency | 0 | 0 | - | 0 |
|  | Indirectness | 0 | 0 | - | 0 |
|  | Impression | -1^④^ | 0 | 0 | 0 |
|  | Publication bias | 0 | 0 | - | 0 |
| Fractional Exhaled Nitric Oxide (FeNO) | Risk of bias | -1^①^ | -1^①^ | - | -1^①^ |
|  | Inconsistency | 0 | 0 | - | 0 |
|  | Indirectness | 0 | 0 | - | 0 |
|  | Impression | -1^④^ | -1^④^ | - | -1^④^ |
|  | Publication bias | 0 | 0 | - | 0 |

GRADE

A:The conclusions of researcher Ph.D.Yongxiu Liu.

B:The conclusions of researcher Ph.D. Yuguo Li.

C:In case of a difference of opinions, it shall be adjudicated by Professor Lei Gao.

D:Conclusive conclusion.

①Methodological quality of included studies was low, with biases in randomization, allocation concealment, and blinding. ②The heterogeneity was large and low confidence interval overlap. ③The population was not broadly representative. ④Small sample size, 95% confidence intervals include null values. ⑤Few studies were included, the funnel plot was not symmetrical, Egger’s test found that publication bias or results were positive, and there was no publication bias evaluation.

Study 37：Williamson, Anne et al. “Vitamin D for the management of asthma.” The Cochrane database of systematic reviews vol. 2,2 CD011511. 6 Feb. 2023, doi:10.1002/14651858.CD011511.pub3.PMID: 36744416 PMCID: PMC9899558

| Endpoint measure | Downgrading factor | A | B | C | D |
| --- | --- | --- | --- | --- | --- |
| Proportion of participants with one or more exacerbations treated with systemic corticosteroidsIndirectness Impression  Publication bias | Risk of bias | 0 | 0 | - | 0 |
|  | Inconsistency | 0 | 0 | - | 0 |
|  | Indirectness | 0 | 0 | - | 0 |
|  | Impression | 0 | 0 | - | 0 |
|  | Publication bias | 0 | 0 | - | 0 |
| Rate of exacerbations treated with systemic corticosteroids | Risk of bias | 0 | 0 | - | 0 |
|  | Inconsistency | 0 | 0 | - | 0 |
|  | Indirectness | 0 | 0 | - | 0 |
|  | Impression | 0 | 0 | - | 0 |
|  | Publication bias | 0 | 0 | - | 0 |
| Time to first exacerbation treated with systemic corticosteroids | Risk of bias | 0 | 0 | - | 0 |
|  | Inconsistency | 0 | 0 | - | 0 |
|  | Indirectness | 0 | 0 | - | 0 |
|  | Impression | 0 | 0 | - | 0 |
|  | Publication bias | 0 | 0 | - | 0 |
| Proportion of participants with one or more exacerbations requiring emergency department visit or hospitalisation, or both | Risk of bias | 0 | 0 | - | 0 |
|  | Inconsistency | 0 | 0 | - | 0 |
|  | Indirectness | 0 | 0 | - | 0 |
|  | Impression | -1^④^ | -1^④^ | - | -1^④^ |
|  | Publication bias | 0 | 0 | - | 0 |
| End-study childhood Asthma Control Test (cACT) or Asthma Control Test (ACT) score | Risk of bias | 0 | 0 | - | 0 |
|  | Inconsistency | 0 | -1^②^ | -1^②^ | -1^②^ |
|  | Indirectness | 0 | 0 | - | 0 |
|  | Impression | -1^④^ | 0 | 0 | 0 |
|  | Publication bias | 0 | 0 | - | 0 |
| End-study % predicted forced expiratory volume in one second (FEV1) | Risk of bias | 0 | 0 | - | 0 |
|  | Inconsistency | 0 | 0 | - | 0 |
|  | Indirectness | 0 | 0 | - | 0 |
|  | Impression | 0 | 0 | - | 0 |
|  | Publication bias | 0 | 0 | - | 0 |
| Proportion of participants with one or more serious adverse events due to any cause | Risk of bias | 0 | 0 | - | 0 |
|  | Inconsistency | 0 | 0 | - | 0 |
|  | Indirectness | 0 | 0 | - | 0 |
|  | Impression | 0 | 0 | - | 0 |
|  | Publication bias | 0 | 0 | - | 0 |
| Proportion of participants with fatal asthma exacerbation | Risk of bias | 0 | 0 | - | 0 |
|  | Inconsistency | 0 | 0 | - | 0 |
|  | Indirectness | 0 | 0 | - | 0 |
|  | Impression | -1^④^ | -1^④^ | - | -1^④^ |
|  | Publication bias | 0 | 0 | - | 0 |
| Proportion of participants with one ormore exacerbation as defined in primary trials | Risk of bias | 0 | 0 | - | 0 |
|  | Inconsistency | -1^②^ | 0 | 0 | 0 |
|  | Indirectness | -1^③^ | 0 | 0 | 0 |
|  | Impression | 0 | 0 | - | 0 |
|  | Publication bias | 0 | 0 | - | 0 |
| End-study % eosinophils, lower airway | Risk of bias | 0 | 0 | - | 0 |
|  | Inconsistency | 0 | -1^②^ | -1^②^ | -1^②^ |
|  | Indirectness | 0 | 0 | - | 0 |
|  | Impression | -1^④^ | 0 | 0 | 0 |
|  | Publication bias | 0 | 0 | - | 0 |
| End-study log10 total IgE, IU/ml | Risk of bias | 0 | 0 | - | 0 |
|  | Inconsistency | 0 | 0 | - | 0 |
|  | Indirectness | 0 | 0 | - | 0 |
|  | Impression | -1^④^ | 0 | 0 | 0 |
|  | Publication bias | 0 | 0 | - | 0 |
| End-study % predicted forced vital capacity (FVC) | Risk of bias | 0 | 0 | - | 0 |
|  | Inconsistency | -1^②^ | -1^②^ | - | -1^②^ |
|  | Indirectness | 0 | 0 | - | 0 |
|  | Impression | -1^④^ | 0 | 0 | 0 |
|  | Publication bias | 0 | 0 | - | 0 |
| End-study peak expiratory flow rate (PEFR) (L/min) | Risk of bias | 0 | 0 | - | 0 |
|  | Inconsistency | -1^②^ | -1^②^ | - | -1^②^ |
|  | Indirectness | 0 | 0 | - | 0 |
|  | Impression | -1^④^ | 0 | 0 | 0 |
|  | Publication bias | 0 | 0 | - | 0 |
| Proportion of participants withdrawing from trial | Risk of bias | 0 | 0 | - | 0 |
|  | Inconsistency | 0 | 0 | - | 0 |
|  | Indirectness | 0 | 0 | - | 0 |
|  | Impression | 0 | 0 | - | 0 |
|  | Publication bias | 0 | 0 | - | 0 |

GRADE

A:The conclusions of researcher Ph.D.Yongxiu Liu.

B:The conclusions of researcher Ph.D. Yuguo Li.

C:In case of a difference of opinions, it shall be adjudicated by Professor Lei Gao.

D:Conclusive conclusion.

①Methodological quality of included studies was low, with biases in randomization, allocation concealment, and blinding. ②The heterogeneity was large and low confidence interval overlap. ③The population was not broadly representative. ④Small sample size, 95% confidence intervals include null values. ⑤Few studies were included, the funnel plot was not symmetrical, Egger’s test found that publication bias or results were positive, and there was no publication bias evaluation.

Study 38：孙倩.补充维生素D及其类似物对支气管哮喘的影响：一项随机对照试验的荟萃分析[D].南昌大学,2024.DOI:10.27232/d.cnki.gnchu.2024.003676.

| Endpoint measure | Downgrading factor | A | B | C | D |
| --- | --- | --- | --- | --- | --- |
| Forced Expiratory Volume in 1 second (% predicted) | Risk of bias | -1^①^ | -1^①^ | - | -1^①^ |
|  | Inconsistency | -1^②^ | -1^②^ | - | -1^②^ |
|  | Indirectness | 0 | 0 | - | 0 |
|  | Impression | -1^④^ | -1^④^ | - | -1^④^ |
|  | Publication bias | 0 | 0 | - | 0 |
| Asthma Control Test / Children-Asthma Control Test | Risk of bias | -1^①^ | -1^①^ | - | -1^①^ |
|  | Inconsistency | 0 | 0 | - | 0 |
|  | Indirectness | 0 | 0 | - | 0 |
|  | Impression | 0 | 0 | - | 0 |
|  | Publication bias | 0 | 0 | - | 0 |
| Asthma Exacerbation | Risk of bias | -1^①^ | -1^①^ | - | -1^①^ |
|  | Inconsistency | 0 | 0 | - | 0 |
|  | Indirectness | 0 | 0 | - | 0 |
|  | Impression | 0 | 0 | - | 0 |
|  | Publication bias | 0 | 0 | - | 0 |
| Change in serum 25-hydroxyvitamin D from baseline | Risk of bias | -1^①^ | -1^①^ | - | -1^①^ |
|  | Inconsistency | -1^②^ | -1^②^ | - | -1^②^ |
|  | Indirectness | 0 | 0 | - | 0 |
|  | Impression | 0 | -1^④^ | 0 | 0 |
|  | Publication bias | 0 | 0 | - | 0 |
| Fractional exhaled nitric oxide | Risk of bias | -1^①^ | -1^①^ | - | -1^①^ |
|  | Inconsistency | -1^②^ | -1^②^ | - | -1^②^ |
|  | Indirectness | 0 | 0 | - | 0 |
|  | Impression | 0 | -1^④^ | -1^④^ | -1^④^ |
|  | Publication bias | 0 | 0 | - | 0 |
| Adverse events | Risk of bias | -1^①^ | -1^①^ | - | -1^①^ |
|  | Inconsistency | 0 | 0 | - | 0 |
|  | Indirectness | 0 | 0 | - | 0 |
|  | Impression | -1^④^ | -1^④^ | - | -1^④^ |
|  | Publication bias | 0 | 0 | - | 0 |

GRADE

A:The conclusions of researcher Ph.D.Yongxiu Liu.

B:The conclusions of researcher Ph.D. Yuguo Li.

C:In case of a difference of opinions, it shall be adjudicated by Professor Lei Gao.

D:Conclusive conclusion.

①Methodological quality of included studies was low, with biases in randomization, allocation concealment, and blinding. ②The heterogeneity was large and low confidence interval overlap. ③The population was not broadly representative. ④Small sample size, 95% confidence intervals include null values. ⑤Few studies were included, the funnel plot was not symmetrical, Egger’s test found that publication bias or results were positive, and there was no publication bias evaluation.

Study 39：El Abd, Asmae et al. “The effects of vitamin D supplementation on inflammatory biomarkers in patients with asthma: a systematic review and meta-analysis of randomized controlled trials.”Frontiers in immunology vol. 15 1335968. 13 Mar. 2024, doi:10.3389/fimmu.2024.1335968.PMID: 38545098 PMCID: PMC10965564

| Endpoint measure | Downgrading factor | A | B | C | D |
| --- | --- | --- | --- | --- | --- |
| Serum total Immunoglobulin E (IgE) level | Risk of bias | 0 | 0 | - | 0 |
|  | Inconsistency | 0 | 0 | - | 0 |
|  | Indirectness | 0 | 0 | - | 0 |
|  | Impression | 0 | -1^④^ | -1^④^ | -1^④^ |
|  | Publication bias | 0 | -1^⑤^ | 0 | 0 |
| Blood eosinophil count | Risk of bias | 0 | 0 | - | -1^①^ |
|  | Inconsistency | 0 | 0 | - | 0 |
|  | Indirectness | 0 | 0 | - | 0 |
|  | Impression | 0 | -1^④^ | -1^④^ | -1^④^ |
|  | Publication bias | 0 | -1^⑤^ | 0 | 0 |
| Fractional exhaled nitric oxide (FeNO) | Risk of bias | 0 | -1^①^ | 0 | 0 |
|  | Inconsistency | 0 | 0 | - | 0 |
|  | Indirectness | 0 | -1^③^ | 0 | 0 |
|  | Impression | -1^④^ | -1^④^ | - | -1^④^ |
|  | Publication bias | 0 | -1^⑤^ | 0 | 0 |
| Serum interleukin-10 (IL-10) level | Risk of bias | 0 | 0 | - | 0 |
|  | Inconsistency | -1^②^ | -1^②^ | - | -1^②^ |
|  | Indirectness | 0 | -1^③^ | 0 | 0 |
|  | Impression | -1^④^ | -1^④^ | 0 | -1^④^ |
|  | Publication bias | 0 | -1^⑤^ | 0 | 0 |

GRADE

A:The conclusions of researcher Ph.D.Yongxiu Liu.

B:The conclusions of researcher Ph.D. Yuguo Li.

C:In case of a difference of opinions, it shall be adjudicated by Professor Lei Gao.

D:Conclusive conclusion.

①Methodological quality of included studies was low, with biases in randomization, allocation concealment, and blinding. ②The heterogeneity was large and low confidence interval overlap. ③The population was not broadly representative. ④Small sample size, 95% confidence intervals include null values. ⑤Few studies were included, the funnel plot was not symmetrical, Egger’s test found that publication bias or results were positive, and there was no publication bias evaluation.

Study 40：Fedora, Katherine et al. “Vitamin D supplementation decrease asthma exacerbations in children: a systematic review and meta-analysis of randomized controlled trials.” Annals of medicine vol. 56,1 (2024): 2400313. doi:10.1080/07853890.2024.2400313.PMID: 39421966.PMCID: PMC11492411

| Endpoint measure | Downgrading factor | A | B | C | D |
| --- | --- | --- | --- | --- | --- |
| Incidence of asthma exacerbations | Risk of bias | 0 | -1^①^ | -1^①^ | -1^①^ |
|  | Inconsistency | 0 | -1^②^ | -1^②^ | -1^②^ |
|  | Indirectness | 0 | 0 | - | 0 |
|  | Impression | 0 | -1^④^ | -1^④^ | -1^④^ |
|  | Publication bias | 0 | 0 | - | 0 |
| Serum 25-hydroxyvitamin D (25(OH)D) level | Risk of bias | 0 | 0 | - | 0 |
|  | Inconsistency | -1^②^ | -1^②^ | - | -1^②^ |
|  | Indirectness | 0 | 0 | - | 0 |
|  | Impression | 0 | -1^④^ | 0 | 0 |
|  | Publication bias | 0 | -1^⑤^ | -1^⑤^ | -1^⑤^ |
| Forced expiratory volume in 1 second, % predicted (FEV₁% predicted) | Risk of bias | 0 | 0 | - | 0 |
|  | Inconsistency | 0 | -1^②^ | -1^②^ | -1^②^ |
|  | Indirectness | 0 | 0 | - | 0 |
|  | Impression | -1^④^ | -1^④^ | - | -1^④^ |
|  | Publication bias | 0 | -1^⑤^ | -1^⑤^ | -1^⑤^ |

GRADE

A:The conclusions of researcher Ph.D.Yongxiu Liu.

B:The conclusions of researcher Ph.D. Yuguo Li.

C:In case of a difference of opinions, it shall be adjudicated by Professor Lei Gao.

D:Conclusive conclusion.

①Methodological quality of included studies was low, with biases in randomization, allocation concealment, and blinding. ②The heterogeneity was large and low confidence interval overlap. ③The population was not broadly representative. ④Small sample size, 95% confidence intervals include null values. ⑤Few studies were included, the funnel plot was not symmetrical, Egger’s test found that publication bias or results were positive, and there was no publication bias evaluation.

Study 41：Niu, Haiying et al. “Asthmatic patients with vitamin D deficiency: Can vitamin D supplementation make a difference.” Technology and health care : official journal of the European Society for Engineering and Medicine vol. 32,6 (2024): 3985-4008. doi:10.3233/THC-231462.PMID: 39031398.PMCID: PMC11612934

| Endpoint measure | Downgrading factor | A | B | C | D |
| --- | --- | --- | --- | --- | --- |
| Incidence of asthma exacerbations | Risk of bias | 0 | -1^①^ | - | -1^①^ |
|  | Inconsistency | 0 | -1^②^ | - | -1^②^ |
|  | Indirectness | 0 | 0 | - | 0 |
|  | Impression | 0 | -1^④^ | - | -1^④^ |
|  | Publication bias | 0 | -1^⑤^ | - | 0 |
| Incidence of asthma exacerbations requiring systemic corticosteroids | Risk of bias | 0 | 0 | - | 0 |
|  | Inconsistency | 0 | 0 | - | 0 |
|  | Indirectness | 0 | 0 | - | 0 |
|  | Impression | 0 | -1^④^ | - | 0 |
|  | Publication bias | 0 | -1^⑤^ | -1^⑤^ | -1^⑤^ |
| Incidence of asthma exacerbations requiring emergency department visit or hospitalization, or both | Risk of bias | 0 | 0 | - | 0 |
|  | Inconsistency | 0 | 0 | - | 0 |
|  | Indirectness | 0 | 0 | - | 0 |
|  | Impression | 0 | -1^④^ | - | 0 |
|  | Publication bias | 0 | -1^⑤^ | -1^⑤^ | -1^⑤^ |
| Forced expiratory volume in 1 second, % predicted (FEV₁% predicted) | Risk of bias | 0 | 0 | - | -1^①^ |
|  | Inconsistency | 0 | -1^②^ | -1^②^ | -1^②^ |
|  | Indirectness | 0 | 0 | - | 0 |
|  | Impression | -1^④^ | -1^④^ | - | -1^④^ |
|  | Publication bias | 0 | -1^⑤^ | 0 | 0 |
| Serum 25-hydroxyvitamin D (25(OH)D) level | Risk of bias | -1^①^ | -1^①^ | - | -1^①^ |
|  | Inconsistency | -1^②^ | -1^②^ | - | -1^②^ |
|  | Indirectness | 0 | 0 | - | 0 |
|  | Impression | 0 | -1^④^ | -1^④^ | -1^④^ |
|  | Publication bias | 0 | 0 | - | 0 |
| Incidence of adverse events | Risk of bias | 0 | 0 | - | 0 |
|  | Inconsistency | 0 | 0 | - | 0 |
|  | Indirectness | 0 | 0 | - | 0 |
|  | Impression | -1^④^ | 0 | -1^④^ | -1^④^ |
|  | Publication bias | 0 | -1^⑤^ | -1^⑤^ | -1^⑤^ |
| Incidence of fatal asthma exacerbations | Risk of bias | 0 | -1^①^ | -1^①^ | -1^①^ |
|  | Inconsistency | 0 | -1^②^ | 0 | 0 |
|  | Indirectness | 0 | 0 | - | 0 |
|  | Impression | -1^④^ | -1^④^ | - | -1^④^ |
|  | Publication bias | 0 | -1^⑤^ | -1^⑤^ | -1^⑤^ |

GRADE

A:The conclusions of researcher Ph.D.Yongxiu Liu.

B:The conclusions of researcher Ph.D. Yuguo Li.

C:In case of a difference of opinions, it shall be adjudicated by Professor Lei Gao.

D:Conclusive conclusion.

①Methodological quality of included studies was low, with biases in randomization, allocation concealment, and blinding. ②The heterogeneity was large and low confidence interval overlap. ③The population was not broadly representative. ④Small sample size, 95% confidence intervals include null values. ⑤Few studies were included, the funnel plot was not symmetrical, Egger’s test found that publication bias or results were positive, and there was no publication bias evaluation.

Study 42：杨玉丰,张慧中. 维生素D联合丙酸氟替卡松治疗儿童哮喘有效性的Meta分析[J]. 今日健康,2025(12):105-108. DOI:10.3969/j.issn.1671-5160.2025.12.044.

| Endpoint measure | Downgrading factor | A | B | C | D |
| --- | --- | --- | --- | --- | --- |
| Total treatment effective rate | Risk of bias | -1^①^ | -1^①^ | - | -1^①^ |
|  | Inconsistency | 0 | 0 | - | 0 |
|  | Indirectness | 0 | 0 | - | 0 |
|  | Impression | 0 | 0 | - | 0 |
|  | Publication bias | 0 | -1^⑤^ | -1^⑤^ | -1^⑤^ |
| Lung function index: Forced Vital Capacity (FVC) | Risk of bias | -1^①^ | -1^①^ | - | -1^①^ |
|  | Inconsistency | -1^②^ | -1^②^ | - | -1^②^ |
|  | Indirectness | 0 | 0 | - | 0 |
|  | Impression | 0 | -1^④^ | 0 | 0 |
|  | Publication bias | -1^⑤^ | 0 | -1^⑤^ | -1^⑤^ |
| Lung function index: Forced Expiratory Volume in 1 second (FEV₁) | Risk of bias | -1^①^ | -1^①^ | - | -1^①^ |
|  | Inconsistency | -1^②^ | -1^②^ | - | -1^②^ |
|  | Indirectness | 0 | 0 | - | 0 |
|  | Impression | 0 | -1^④^ | 0 | 0 |
|  | Publication bias | -1^⑤^ | 0 | -1^⑤^ | -1^⑤^ |
| Lung function index: Peak Expiratory Flow (PEF) | Risk of bias | -1^①^ | -1^①^ | - | -1^①^ |
|  | Inconsistency | -1^②^ | -1^②^ | - | -1^②^ |
|  | Indirectness | 0 | 0 | - | 0 |
|  | Impression | -1^④^ | -1^④^ | - | -1^④^ |
|  | Publication bias | -1^⑤^ | 0 | -1^⑤^ | -1^⑤^ |
| Immune function index: Immunoglobulin A (IgA) level | Risk of bias | -1^①^ | -1^①^ | - | -1^①^ |
|  | Inconsistency | -1^②^ | -1^②^ | - | -1^②^ |
|  | Indirectness | 0 | -1^③^ | 0 | 0 |
|  | Impression | -1^④^ | -1^④^ | - | 0 |
|  | Publication bias | -1^⑤^ | 0 | -1^⑤^ | -1^⑤^ |
| Immune function index: Immunoglobulin G (IgG) level | Risk of bias | -1^①^ | -1^①^ | - | -1^①^ |
|  | Inconsistency | 0 | 0 | - | 0 |
|  | Indirectness | 0 | -1^③^ | 0 | 0 |
|  | Impression | -1^④^ | 0 | 0 | 0 |
|  | Publication bias | -1^⑤^ | -1^⑤^ | - | -1^⑤^ |
| Immune function index: Immunoglobulin M (IgM) level | Risk of bias | -1^①^ | -1^①^ | - | -1^①^ |
|  | Inconsistency | 0 | 0 | - | 0 |
|  | Indirectness | 0 | -1^③^ | 0 | 0 |
|  | Impression | -1^④^ | 0 | 0 | 0 |
|  | Publication bias | -1^⑤^ | -1^⑤^ | - | -1^⑤^ |

**6.Citation Overlap Matrix and the Corrected Covered Area (CCA).**

|  | 1 | 2 | 3 | 4 | 5 | 6 | 7 | 8 | 9 | 10 | 11 | 12 | 13 | 14 | 15 | 16 | 17 | 18 | 19 | 20 | 21 |
| --- | --- | --- | --- | --- | --- | --- | --- | --- | --- | --- | --- | --- | --- | --- | --- | --- | --- | --- | --- | --- | --- |
| Worth 1994 |  | √ |  |  |  |  | √ |  |  |  |  |  |  |  |  |  |  |  |  | √ |  |
| Schou 2003 | √ |  | √ | √ |  |  |  | √ | √ |  |  |  |  |  |  |  |  |  |  |  |  |
| Majak 2009 | √ | √ |  | √ |  |  | √ | √ | √ | √ | √ |  | √ |  |  | √ | √ |  | √ | √ |  |
| Urashima 2010 |  |  | √ | √ |  | √ | √ |  | √ |  |  |  | √ |  |  | √ |  |  | √ | √ |  |
| Manaseki-Holland 2010 |  |  |  |  |  |  |  |  |  |  |  |  |  |  |  |  |  |  |  |  |  |
| Kumar 2011 |  |  |  |  |  |  |  |  |  |  |  |  |  |  |  |  |  |  |  |  |  |
| 雷自强 2011 |  |  |  |  |  |  | √ |  |  |  |  |  |  |  |  |  |  |  |  | √ |  |
| Majak 2011 |  |  | √ | √ |  | √ | √ | √ | √ | √ | √ |  | √ | √ |  | √ | √ |  | √ | √ |  |
| Camargo 2012 |  |  |  |  |  |  |  |  |  |  |  |  |  |  |  |  |  |  |  |  |  |
| Choudhary 2012 |  |  |  |  |  |  |  |  |  |  |  |  |  |  |  |  |  |  |  |  |  |
| 刘飒 2012 |  |  |  |  | √ |  |  |  |  |  |  |  |  |  |  |  |  |  |  |  |  |
| 李华亭 2012 |  |  |  |  | √ |  |  |  |  |  |  |  |  |  |  |  |  |  |  |  |  |
| Lewis 2012 | √ |  | √ | √ |  |  |  | √ |  |  |  |  | √ |  |  | √ |  |  |  |  |  |
| Manaseki-Holland 2012 |  |  |  |  |  |  |  |  |  |  |  |  |  |  |  |  |  |  |  |  |  |
| Darabi 2013 |  |  |  | √ |  |  |  |  |  |  |  |  | √ |  |  |  |  |  |  |  |  |
| 李发明 2013 |  |  |  |  | √ |  |  |  |  |  |  |  |  |  |  |  |  |  |  |  |  |
| Arshi2014 |  |  |  |  |  |  |  |  |  | √ |  |  |  |  |  |  | √ |  |  |  |  |
| Baris 2014 |  | √ |  | √ |  |  | √ |  | √ |  | √ |  | √ |  |  |  |  |  |  | √ |  |
| Castro 2014 |  | √ |  |  |  | √ | √ |  |  | √ | √ |  |  | √ |  | √ | √ | √ |  | √ |  |
| 刘黎 2014 |  |  |  |  | √ |  |  |  |  |  |  |  |  |  |  |  |  |  |  |  |  |
| 张娴 2014 |  |  |  |  | √ |  |  |  |  |  |  |  |  |  |  |  |  |  |  |  |  |
| 李源 2014 |  |  |  |  | √ |  |  |  |  |  |  |  |  |  |  |  |  |  |  |  |  |
| 王立民 2014 |  |  |  |  | √ |  |  |  |  |  |  |  |  |  |  |  |  |  |  |  |  |
| Yadav and Mitta 2014 |  |  | √ | √ |  |  | √ | √ | √ |  | √ |  | √ | √ | √ | √ | √ | √ |  | √ |  |
| Bar Yoseph R 2015 |  |  |  |  |  |  |  | √ |  |  |  | √ | √ |  |  |  |  | √ | √ |  |  |
| De Groot 2015 |  | √ |  |  |  |  | √ |  |  | √ | √ |  |  | √ |  |  | √ | √ |  | √ |  |
| Martineau 2015 |  | √ |  |  |  |  | √ |  |  | √ | √ |  |  |  |  | √ | √ | √ |  | √ |  |
| Nageswari 2015 |  |  |  |  |  |  |  |  |  | √ |  |  |  |  |  |  |  |  |  |  |  |
| 刘艳芳 2015 |  |  |  |  | √ |  |  |  |  |  |  |  |  |  |  |  |  |  |  |  |  |
| 李晓东 2015 |  |  |  |  | √ |  |  |  |  |  |  |  |  |  |  |  |  |  |  |  |  |
| Tachimoto 2016 |  |  |  |  |  | √ | √ |  | √ |  |  | √ | √ |  |  | √ | √ | √ | √ |  |  |
| Kerley 2016 |  |  |  |  |  | √ |  | √ | √ | √ |  | √ | √ |  |  | √ | √ | √ |  |  |  |
| Jensen 2016 |  |  |  |  |  | √ | √ |  |  | √ |  | √ | √ |  |  | √ | √ |  |  | √ |  |
| Jerzynska 2016 |  |  |  |  |  |  |  |  |  |  |  |  |  |  |  | √ |  |  |  |  |  |
| Yoseph 2015 |  |  |  |  |  |  |  |  |  | √ |  |  |  |  |  |  | √ |  |  |  |  |
| Ali 2017 |  |  |  |  |  |  |  |  |  | √ |  |  |  |  |  |  | √ |  |  |  |  |
| Alansari 2017 |  |  |  |  |  |  |  |  |  |  |  |  | √ |  | √ |  |  |  |  |  |  |
| ABBAS 2017 |  |  |  |  |  |  |  |  |  | √ |  |  |  |  |  |  | √ | √ |  |  |  |
| 陈江 2017 |  |  |  |  |  |  |  |  |  |  |  |  |  |  |  |  | √ |  |  |  |  |
| Jiang 2017 |  |  |  |  |  |  |  |  |  |  |  |  |  |  |  | √ |  |  |  |  |  |
| Musharraf 2017 |  |  |  |  |  |  |  |  |  | √ |  |  |  | √ |  |  | √ |  |  |  |  |
| Najmuddin 2017 |  |  |  |  |  |  |  |  |  |  |  |  | √ |  |  |  |  |  |  |  |  |
| Rubén 2017 |  |  |  |  |  |  |  |  |  | √ |  |  |  |  |  |  |  |  |  |  |  |
| Kang 2018 |  |  |  |  |  |  |  |  | √ |  |  |  |  |  |  |  |  |  | √ |  |  |
| Ramos-Martínez 2018 |  |  |  |  |  |  |  |  |  |  |  |  |  |  |  | √ |  | √ |  |  |  |
| 王志华 2018 |  |  |  |  |  |  |  |  |  |  |  |  |  |  |  |  |  |  |  |  | √ |
| Aglipay 2019 |  |  |  |  |  |  |  |  |  |  |  |  |  |  | √ | √ |  |  |  |  |  |
| 李燕京2019 |  |  |  |  |  |  |  |  |  |  |  |  |  |  |  |  |  |  |  |  | √ |
| Shabana 2019 |  |  |  |  |  |  |  |  |  |  |  |  |  | √ |  |  |  | √ |  |  |  |
| Ducharme 2019 |  |  |  |  |  |  |  |  |  |  | √ | √ | √ |  |  | √ | √ |  |  |  |  |
| Dodamani 2019 |  |  |  |  |  |  |  |  |  |  | √ |  |  | √ |  |  |  | √ |  |  |  |
| 贾金华 2019 |  |  |  |  |  |  |  |  |  |  |  |  |  |  |  |  |  |  |  |  | √ |
| Swangtrakul 2019 |  |  |  |  |  |  |  |  |  |  |  |  | √ |  |  |  |  |  |  |  |  |
| 杨帆 2019 |  |  |  |  |  |  |  |  |  |  |  |  |  |  |  |  |  |  |  |  | √ |
| Ardestani 2020 a |  |  |  |  |  |  |  |  |  |  |  |  |  |  |  |  | √ |  |  |  |  |
| Ardestani 2020 b |  |  |  |  |  |  |  |  |  |  |  |  |  |  |  |  | √ |  |  |  |  |
| Forno 2020 |  |  |  |  |  |  |  |  |  |  | √ | √ | √ |  | √ | √ | √ |  | √ |  |  |
| 师晶玉 2020 |  |  |  |  |  |  |  |  |  |  |  |  |  |  |  |  |  |  |  |  | √ |
| 张潮 2020 |  |  |  |  |  |  |  |  |  |  |  |  |  |  |  |  |  |  |  |  | √ |
| 张军 2020 |  |  |  |  |  |  |  |  |  |  |  |  |  |  |  |  |  |  |  |  | √ |
| Andújar-Espinosa 2021 |  |  |  |  |  |  |  |  |  |  |  |  |  |  |  | √ | √ | √ |  |  |  |
| Camargo 2021 |  |  |  |  |  |  |  |  |  |  |  |  |  |  |  | √ |  |  |  |  |  |
| 高原 2021 |  |  |  |  |  |  |  |  |  |  |  |  |  |  |  |  |  |  |  |  | √ |
| Jat 2021 |  |  |  |  |  |  |  |  |  |  | √ | √ | √ | √ | √ | √ | √ |  | √ |  |  |
| Thakur 2021 |  |  |  |  |  |  |  |  |  |  |  | √ | √ | √ | √ | √ | √ | √ | √ |  |  |
| Andújar-Espinosa 2022 |  |  |  |  |  |  |  |  |  |  | √ |  |  |  |  |  |  |  |  |  |  |
| 林东 2022 |  |  |  |  |  |  |  |  |  |  |  |  |  |  |  |  |  |  |  |  | √ |
| Rosser 2022 |  |  |  |  |  |  |  |  |  |  |  |  |  |  |  |  |  | √ |  |  |  |
| 朱虹 2022 |  |  |  |  |  |  |  |  |  |  |  |  |  |  |  |  |  |  |  |  | √ |
| 周琦琦 2022 |  |  |  |  |  |  |  |  |  |  |  |  |  |  |  |  |  |  |  |  | √ |
| 陈丽芳 2023 |  |  |  |  |  |  |  |  |  |  |  |  |  |  |  |  |  |  |  |  | √ |

**7.Literature Screening：**

1 Asthma is not the central research focus or a primary therapeutic target

|  | 2024 | Intestinal Microbiota Transplant as a Strategy to Enhance the Resilience Capacity of the Elderly Aiming to Retain Muscular, Cognitive, and Metabolic Functions in a Stressful Environment. |
| --- | --- | --- |
| Adachi, J. D. and A. Papaioannou | 2005 | In whom and how to prevent glucocorticoid-induced osteoporosis |
| Adams, J. B., J. K. Kirby, J. C. Sorensen, E. L. Pollard and T. Audhya | 2022 | Evidence based recommendations for an optimal prenatal supplement for women in the US: vitamins and related nutrients |
| Amestoy, A., C. Baudrillard, K. Briot, A. Pizano, M. Bouvard and M. C. Lai | 2023 | Steroid hormone pathways, vitamin D and autism: a systematic review |
| Amrein, K., M. Hoffmann, E. Lobmeyr and G. Martucci | 2021 | Vitamin D in critical care: where are we now and what is next? |
| Andersen, C. L., T. K. Kristensen, M. T. Severinsen, M. B. Møller, H. Vestergaard, O. J. Bergmann, H. C. Hasselbalch and O. W. Bjerrum | 2012 | Systemic mastocytosis - A systematic review |
| Anikeeva, O., P. Bi, J. E. Hiller, P. Ryan, D. Roder and G. S. Han | 2010 | The Health Status of Migrants in Australia: A Review |
| Aryan, Z., N. Rezaei and C. A. Camargo | 2017 | Vitamin D status, aeroallergen sensitization, and allergic rhinitis: A systematic review and meta-analysis |
| Autier, P., P. Mullie, A. Macacu, M. Dragomir, M. Boniol, K. Coppens, C. Pizot and M. Boniol | 2017 | Effect of vitamin D supplementation on non-skeletal disorders: a systematic review of meta-analyses and randomised trials |
| Aziz, D. A., S. K. Fatima and H. N. Tahir | 2021 | Risk factors leading to pulmonary exacerbation in patients with cystic fibrosis: A systematic review |
| Bang, A. S., M. Hakimi, P. Tahir, T. Bhutani and K. S. Leslie | 2023 | Biologic Therapies in HIV/AIDS Patients with Inflammatory Diseases: A Systematic Review of the Literature |
| Basha, M., H. A. Majid, N. Razali and A. Yahya | 2020 | Risk of eczema, wheezing and respiratory tract infections in the first year of life: A systematic review of vitamin D concentrations during pregnancy and at birth |
| Beauchesne, A. R., K. C. Cara, D. M. Krobath, L. P. Penkert, S. P. Shertukde, D. S. Cahoon, B. Prado, R. Li, Q. Yao, J. Huang, T. Reh and M. Chung | 2022 | Vitamin D intakes and health outcomes in infants and preschool children: Summary of an evidence report |
| Ben-Eltriki, M., R. Hopefl, J. M. Wright and S. Deb | 2022 | Association between Vitamin D Status and Risk of Developing Severe COVID-19 Infection: A Meta-Analysis of Observational Studies |
| Bi, W. G. and S. Q. Wei | 2018 | Vitamin D supplementation during pregnancy and offspring mortality and morbidity: A systematic review |
| Bradley, R., J. Schloss, D. Brown, D. Celis, J. Finnell, R. Hedo, V. Honcharov, T. Pantuso, H. Peña, R. Lauche and A. Steel | 2020 | The effects of vitamin D on acute viral respiratory infections: A rapid review |
| Brustad, N., N. R. Fink, J. Stokholm, K. Bonnelykke, N. V. Folsgaard, D. Hougaard, S. Brix, J. Lasky-Su, S. T. Weiss and B. Chawes | 2022 | Associations of 25 Hydroxyvitamin D and High Sensitivity C-reactive Protein Levels in Early Life |
| Buonsanti, G. | 2011 | VITAMIN D: FROM ANTIRACHITIC FACTOR TO INDICATOR OF THE GENERAL STATE OF HEALTH |
| Challem, J. | 2006 | Medical journal watch: Context and applications |
| Chen, Y. C. S., H. Mirzakhani, H. Knihtilä, R. N. Fichorova, N. Luu, N. Laranjo, A. Jha, R. S. Kelly, S. T. Weiss, A. A. Litonjua and K. A. Lee-Sarwar | 2024 | The Association of Prenatal C-Reactive Protein and Interleukin-8 Levels with Maternal Characteristics and Preterm Birth |
| Christensen, N., J. Sondergaard, N. Fisker and H. T. Christesen | 2017 | Infant Respiratory Tract Infections or Wheeze and Maternal Vitamin D in Pregnancy A Systematic Review |
| Chu, A. W. L., M. M. Wong, D. G. Rayner, G. H. Guyatt, J. P. Díaz Martinez, R. Ceccacci, I. X. Zhao, E. McMullen, A. Srivastava, J. Wang, A. Wen, F. C. Wang, R. Brignardello-Petersen, A. Izcovich, P. Oykhman, K. E. Wheeler, J. Wang, J. M. Spergel, J. A. Singh, J. I. Silverberg, P. Y. Ong, M. O'Brien, S. A. Martin, P. A. Lio, M. L. Lind, J. LeBovidge, E. Kim, J. Huynh, M. Greenhawt, D. D. Gardner, W. T. Frazier, K. Ellison, L. Chen, K. Capozza, A. De Benedetto, M. Boguniewicz, W. Smith Begolka, R. N. Asiniwasis, L. C. Schneider and D. K. Chu | 2023 | Systemic treatments for atopic dermatitis (eczema): Systematic review and network meta-analysis of randomized trials |
| Chu, D. K., J. J. Koplin, T. Ahmed, N. Islam, C. L. Chang and A. J. Lowe | 2024 | How to Prevent Atopic Dermatitis (Eczema) in 2024: Theory and Evidence |
| Chua, J. E. and K. M. Gutierrez | 2023 | The effect of vitamin D as adjunct to allergen immunotherapy (AIT): A systematic review and meta-analysis |
| Clausen, M., K. Jonasson, T. Keil, K. Beyer and S. T. Sigurdardottir | 2018 | Fish oil in infancy protects against food allergy in IcelandResults from a birth cohort study |
| Conradi, S., U. Malzahn, F. Paul, S. Quill, L. Harms, F. T. Bergh, A. Ditzenbach, T. Georgi, P. Heuschmann and B. Rosche | 2013 | Breastfeeding is associated with lower risk for multiple sclerosis |
| Curtis, E. M., R. J. Moon, N. C. Harvey and C. Cooper | 2018 | Maternal vitamin D supplementation during pregnancy |
| Curtis, J. R. and K. G. Saag | 2007 | Prevention and treatment of glucocorticoid-induced osteoporosis |
| Davidson, Z. E., K. Z. Walker and H. Truby | 2012 | Do glucocorticosteroids alter vitamin D status? A systematic review with meta-analyses of observational studies |
| De Almeida Brasiel, P. G., F. V. Guimarães, P. M. Rodrigues, D. C. Bou-Habib and V. D. F. Carvalho | 2022 | Therapeutic Efficacy of Flavonoids in Allergies: A Systematic Review of Randomized Controlled Trials |
| Eichenfield, L. F., J. Ahluwalia, A. Waldman, J. Borok, J. Udkoff and M. Boguniewicz | 2017 | Current guidelines for the evaluation and management of atopic dermatitis: A comparison of the Joint Task Force Practice Parameter and American Academy of Dermatology guidelines |
| Elhage, K. G., A. Kranyak, J. Q. Jin, K. Haran, R. K. Spencer, P. L. Smith, M. S. Davis, M. Hakimi, T. Bhutani and W. Liao | 2024 | Mendelian Randomization Studies in Atopic Dermatitis: A Systematic Review |
| Essien, E. E. | 2019 | 硝酸盐暴露与癌症的关系：文献计量学分析与系统评价 硕士, 东南大学. |
| Feng, H., Y. Chen, X. J. Xiong, Q. Y. Xu, Z. W. Zhang, Q. H. Xi, Y. N. Wu and Y. A. Lu | 2023 | Association of nutrients intake during pregnancy with the risk of allergic disease in offspring: a meta-analysis of prospective cohort studies |
| Fisher, S. A., M. Rahimzadeh, C. Brierley, B. Gration, C. Doree, C. E. Kimber, A. P. Cajide, A. A. Lamikanra and D. J. Roberts | 2019 | The role of Vitamin D in increasing circulating T regulatory cell numbers and modulating T regulatory cell phenotypes in patients with inflammatory disease or in healthy volunteers: A systematic review |
| Freedman, R., S. K. Hunter and M. C. Hoffman | 2018 | Prenatal Primary Prevention of mental illness by micronutrient supplements in pregnancy |
| Fried, D. A., J. Rhyu, K. Odato, H. Blunt, M. R. Karagas and D. Gilbert-Diamond | 2016 | Maternal and cord blood vitamin D status and childhood infection and allergic disease: a systematic review |
| Ganmaa, D., D. Enkhmaa, E. Nasantogtokh, S. Sukhbaatar, K. E. Tumur-Ochir and J. E. Manson | 2022 | Vitamin D, respiratory infections, and chronic disease: Review of meta-analyses and randomized clinical trials |
| Garcia-Larsen, V., D. Ierodiakonou, K. Jarrold, S. Cunha, J. Chivinge, Z. Robinson, N. Geoghegan, A. Ruparelia, P. Devani, M. Trivella, J. Leonardi-Bee and R. J. Boyle | 2018 | Diet during pregnancy and infancy and risk of allergic or autoimmune disease: A systematic review and meta-analysis |
| Giorgetti, A., V. Orazietti, F. P. Busardò, F. Pirani and R. Giorgetti | 2021 | Died with or died of? Development and testing of a SARS CoV-2 significance score to assess the role of covid-19 in the deaths of affected patients |
| Głąbska, D., A. Kołota, K. Lachowicz, D. Skolmowska, M. Stachoń and D. Guzek | 2023 | Supplementation of Vitamin D and Mental Health in Adults with Respiratory System Diseases: A Systematic Review of Randomized Controlled Trials |
| Glintborg, D. and M. Andersen | 2017 | Morbidity in polycystic ovary syndrome |
| Griffith, R., J. Alsweiler, A. Moore, S. Brown, P. Middleton, E. Shepherd and C. Crowther | 2019 | Interventions to prevent women developing gestational diabetes mellitus: An overview |
| Griffith, R. J., J. Alsweiler, A. E. Moore, S. Brown, P. Middleton, E. Shepherd and C. A. Crowther | 2020 | Interventions to prevent women from developing gestational diabetes mellitus: an overview of Cochrane Reviews |
| Hansen, L. B. and S. F. Vondracek | 2004 | Prevention and treatment of nonpostmenopausal osteoporosis |
| Harvey, N., C. Holroyd, G. Ntani, K. Javaid, P. Cooper, R. Moon, Z. Cole, T. Tinati, N. Bishop, K. Godfrey, E. Dennison, J. Baird and C. Cooper | 2013 | Maternal vitamin d status in pregnancy and offspring bone health: A systematic reviewand meta-analysis |
| Harvey, N. C., C. Holroyd, G. Ntani, K. Javaid, P. Cooper, R. Moon, Z. Cole, T. Tinati, K. Godfrey, E. Dennison, N. J. Bishop, J. Baird and C. Cooper | 2014 | Vitamin D supplementation in pregnancy: A systematic review |
| Isa, Z. M., N. R. M. Nordin, M. H. Mahmud and S. Hashim | 2022 | An Update on Vitamin D Deficiency Status in Malaysia |
| Isoldi, F. C., A. Garcia, L. Vieites and L. M. Ferreira | 2024 | Keloids in male genitalia—Systematic review and illustrative case report |
| Jain, N., R. Varman, J. A. Tarbox and T. Nguyen | 2021 | Biomolecular endotype factors involved in COVID-19 airway infectivity: A systematic review |
| Jensen, K. K., J. Serup and K. K. Alsing | 2022 | Psoriasis and seasonal variation: A systematic review on reports from Northern and Central Europe—Little overall variation but distinctive subsets with improvement in summer or wintertime |
| Jeyakumar, A., P. Bhalekar and P. Shambharkar | 2024 | Effect of vitamin D supplementation on the immune response to respiratory tract infections and inflammatory conditions: A systematic review and meta-analysis |
| Jia, X. X., H. Zheng, X. M. Yan, H. Dai and Q. W. Xiang | 2022 | Effect of baseline serum vitamin D level on symptom and medication scores of subcutaneous immunotherapy in children with mite allergy |
| Jin, J. Q., K. G. Elhage, R. K. Spencer, M. S. Davis, M. Hakimi, T. Bhutani and W. Liao | 2023 | Mendelian Randomization Studies in Psoriasis and Psoriatic Arthritis: A Systematic Review |
| Jolliffe, D. A., C. A. Camargo, Jr., J. D. Sluyter, M. Aglipay, J. F. Aloia, P. Bergman, H. A. Bischoff-Ferrari, A. Borzutzky, V. Y. Bubes, C. T. Damsgaard, F. M. Ducharme, G. Dubnov-Raz, S. Esposito, D. Ganmaa, C. Gilham, A. A. Ginde, I. Golan-Tripto, E. C. Goodall, C. C. Grant, C. J. Griffiths, A. M. Hibbs, W. Janssens, A. V. Khadilkar, I. Laaksi, M. T. Lee, M. Loeb, J. L. Maguire, P. Majak, S. Manaseki-Holland, J. E. Manson, D. T. Mauger, D. R. Murdoch, A. Nakashima, R. E. Neale, H. Pham, C. Rake, J. R. Rees, J. Rosendahl, R. Scragg, D. Shah, Y. Shimizu, S. Simpson-Yap, G. T. Kumar, M. Urashima and A. R. Martineau | 2025 | Vitamin D supplementation to prevent acute respiratory infections: systematic review and meta-analysis of stratified aggregate data |
| Jolliffe, D. A., C. A. Camargo, J. D. Sluyter, M. Aglipay, J. F. Aloia, D. Ganmaa, P. Bergman, H. A. Bischoff-Ferrari, A. Borzutzky, C. T. Damsgaard, G. Dubnov-Raz, S. Esposito, C. Gilham, A. A. Ginde, I. Golan-Tripto, E. C. Goodall, C. C. Grant, C. J. Griffiths, A. M. Hibbs, W. Janssens, A. V. Khadilkar, I. Laaksi, M. T. Lee, M. Loeb, J. L. Maguire, P. Majak, D. T. Mauger, S. Manaseki-Holland, D. R. Murdoch, A. Nakashima, R. E. Neale, H. Pham, C. Rake, J. R. Rees, J. Rosendahl, R. Scragg, D. Shah, Y. Shimizu, S. Simpson-Yap, G. Trilok-Kumar, M. Urashima and A. R. Martineau | 2021 | Vitamin D supplementation to prevent acute respiratory infections: a systematic review and meta-analysis of aggregate data from randomised controlled trials |
| Jolliffe, D. A., L. Greenberg, R. L. Hooper, C. Mathyssen, R. Rafiq, R. T. de Jongh, C. A. Camargo, C. J. Griffiths, W. Janssens and A. R. Martineau | 2019 | Vitamin D to prevent exacerbations of COPD: systematic review and meta-analysis of individual participant data from randomised controlled trials |
| Jorde, I., S. Stegemann-Koniszewski, K. Papra, S. Föllner, A. Lux, J. Schreiber and E. Lücke | 2021 | Association of serum vitamin D levels with disease severity, systemic inflammation, prior lung function loss and exacerbations in a cohort of patients with chronic obstructive pulmonary disease (COPD) |
| Kaur, M., K. D. Soni and A. Trikha | 2022 | Does Vitamin D Improve All-cause Mortality in Critically Ill Adults? An Updated Systematic Review and Meta-analysis of Randomized Controlled Trials |
| Khanna, R., D. Nandy and S. Senapati | 2019 | Systematic review and meta-analysis to establish the association of common genetic variations in Vitamin D binding protein with chronic obstructive pulmonary disease |
| Kim, J. H., J. Y. Kim, J. Lee, G. H. Jeong, E. Lee, S. Lee, K. H. Lee, A. Kronbichler, B. Stubbs, M. Solmi, A. Koyanagi, S. H. Hong, E. Dragioti, L. Jacob, A. R. Brunoni, A. F. Carvalho, J. Radua, T. Thompson, L. Smith, H. Oh, L. Yang, I. Grabovac, F. Schuch, M. Fornaro, A. Stickley, T. B. Rais, G. S. de Pablo, J. I. Shin and P. Fusar-Poli | 2020 | Environmental risk factors, protective factors, and peripheral biomarkers for ADHD: an umbrella review |
| Kim, M. J., S. N. Kim, Y. W. Lee, Y. B. Choe and K. J. Ahn | 2016 | Vitamin D Status and Efficacy of Vitamin D Supplementation in Atopic Dermatitis: A Systematic Review and Meta-Analysis |
| Kim, Y. H., K. W. Kim, M. J. Kim, I. S. Sol, S. H. Yoon, H. S. Ahn, H. J. Kim, M. H. Sohn and K. E. Kim | 2016 | Vitamin D levels in allergic rhinitis: a systematic review and meta-analysis |
| Kim, Y. H., M. J. Kim, I. S. Sol, S. H. Yoon, Y. A. Park, K. W. Kim, M. H. Sohn and K. E. Kim | 2016 | Vitamin D level in allergic rhinitis: A systemic review and meta-analysis |
| Kreiner, E., J. Waage, M. Standl, S. Brix, T. H. Pers, A. C. Alves, N. M. Warrington, C. M. T. Tiesler, E. Fuertes, L. Franke, J. N. Hirschhorn, A. James, A. Simpson, J. Y. Tung, G. H. Koppelman, D. S. Postma, C. E. Pennell, M. R. Jarvelin, A. Custovic, N. Timpson, M. A. Ferreira, D. P. Strachan, J. Henderson, D. Hinds, H. Bisgaard and K. Bonnelykke | 2017 | Shared genetic variants suggest common pathways in allergy and autoimmune diseases |
| Kurmangali, Z., B. Abdykalykova, A. Kurmangali, D. Zhantagulov and M. Terzic | 2024 | The Influence of Vitamin D on Pregnancy and Outcomes: Current Knowledge and Future Perspectives |
| LaRosa, D. F. and A. J. Apter | 2004 | Assessing the risk of osteoporosis in patients with asthma and COPD |
| Li, M. X., L. J. Zhao, C. C. Hu, Y. Li, Y. Yang, X. Q. Zhang, Q. G. Li, A. G. Ma and J. Cai | 2024 | Improvement of Lung Function by Micronutrient Supplementation in Patients with COPD: A Systematic Review and Meta-Analysis |
| Li, Q., X. Xu, Y. Liu, S. Yin, Q. Hu, Q. Ji, Y. Zhong and F. Zhu | 2025 | The effects of prenatal vitamin D supplementation on respiratory and allergy-related outcomes in children: A systematic review and meta-analysis of randomized controlled trials |
| Li, Q., Q. Zhou, G. Zhang, X. Tian, Y. Li, Z. Wang, Y. Zhao, Y. Chen and Z. Luo | 2022 | Vitamin D Supplementation and Allergic Diseases during Childhood: A Systematic Review and Meta-Analysis |
| Li, X. Y., J. He, M. Yu and J. Sun | 2020 | The efficacy of vitamin D therapy for patients with COPD: a meta-analysis of randomized controlled trials |
| Linseisen, J. | 2023 | Update on extra-skeletal health effects of vitamin D - implications for recommendations |
| Lips, P., J. P. Bilezikian and R. Bouillon | 2020 | Vitamin D: Giveth to Those Who Needeth |
| Lloyd-Lavery, A., L. Solman, D. J. C. Grindlay, N. K. Rogers, K. S. Thomas and K. E. Harman | 2019 | What's new in atopic eczema? An analysis of systematic reviews published in 2016. Part 2: Epidemiology, aetiology and risk factors |
| Loghman-Adham, M. | 2003 | Medication noncompliance in patients with chronic disease: Issues in dialysis and renal transplantation |
| Lu, C., Y. Jiang, W. Deng, M. J. Lan, L. Wang, Y. Q. Tong, Z. P. Qiao, W. X. Xu, T. Li, W. W. Liu and F. M. Wang | 2025 | Early-life nutritional additives, household environment, and air pollution in relation to childhood food allergies: A multi-city mother-child study in China |
| Luo, C., Y. Sun, Z. Zeng, Y. Liu and S. Peng | 2022 | Vitamin D supplementation in pregnant women or infants for preventing allergic diseases: A systematic review and meta-analysis of randomized controlled trials |
| Luo, S., Y. Liang, T. H. T. Wong, C. M. Schooling and S. L. Au Yeung | 2022 | Identifying factors contributing to increased susceptibility to COVID-19 risk: A systematic review of Mendelian randomization studies |
| Malden, S., J. Gillespie, A. Hughes, A. Gibson, A. Martin, C. Summerbell and J. Reilly | 2019 | The relationship between obesity in early childhood and physical morbidity in childhood and adolescence: A systematic review and meta-analysis |
| Malden, S., J. Gillespie, A. Hughes, A. M. Gibson, A. Farooq, A. Martin, C. Summerbell and J. J. Reilly | 2021 | Obesity in young children and its relationship with diagnosis of asthma, vitamin D deficiency, iron deficiency, specific allergies and flat-footedness: A systematic review and meta-analysis |
| Maretzke, F., A. Bechthold, S. Egert, J. B. Ernst, D. M. van Lent, S. Pilz, J. Reichrath, G. I. Stangl, P. Stehle, D. Volkert, M. Wagner, J. Waizenegger, A. Zittermann and J. Linseisen | 2020 | Role of vitamin D in preventing and treating selected extraskeletal diseases—an umbrella review |
| Marino, R. and M. Misra | 2019 | Extra-Skeletal Effects of Vitamin D |
| Martineau, A. R., D. A. Jolliffe, L. Greenberg, J. F. Aloia, P. Bergman, G. Dubnov-Raz, S. Esposito, D. Ganmaa, A. A. Ginde, E. C. Goodall, C. C. Grant, W. Janssens, M. E. Jensen, C. P. Kerley, I. Laaksi, S. Manaseki-Holland, D. Mauger, D. R. Murdoch, R. Neale, J. R. Rees, S. Simpson, I. Stelmach, G. T. Kumar, M. Urashima, C. A. Camargo, C. J. Griffiths and R. L. Hooper | 2019 | Vitamin D supplementation to prevent acute respiratory infections: individual participant data meta-analysis |
| Martineau, A. R., D. A. Jolliffe, R. L. Hooper, L. Greenberg, J. F. Aloia, P. Bergman, G. Dubnov-Raz, S. Esposito, D. Ganmaa, A. A. Ginde, E. C. Goodall, C. C. Grant, C. J. Griffiths, W. Janssens, I. Laaksi, S. Manaseki-Holland, D. Mauger, D. R. Murdoch, R. Neale, J. R. Rees, S. Simpson, I. Stelmach, G. T. Kumar, M. Urashima and C. A. Camargo | 2017 | Vitamin D supplementation to prevent acute respiratory tract infections: systematic review and meta-analysis of individual participant data |
| Mateussi, M. V., C. O. C. Latorraca, J. P. Daou, A. L. C. Martimbianco, R. Riera, R. L. Pacheco and D. V. Pachito | 2017 | What do Cochrane systematic reviews say about interventions for vitamin D supplementation? |
| McEvoy, C. E. and D. E. Niewoehner | 1997 | Adverse effects of corticosteroid therapy for COPD: A critical review |
| Mead, E., G. Atkinson, B. Richter, M. I. Metzendorf, L. Baur, N. Finer, E. Corpeleijn, C. O'Malley and L. J. Ells | 2016 | Drug interventions for the treatment of obesity in children and adolescents |
| Mirzakhani, H., A. A. Litonjua, T. F. McElrath, G. O'Connor, A. Lee-Parritz, R. Iverson, G. Macones, R. C. Strunk, L. B. Bacharier, R. Zeiger, B. W. Hollis, D. E. Handy, A. Sharma, N. Laranjo, V. Carey, W. L. Qiu, M. Santolini, S. K. Liu, D. Chhabra, D. A. Enquobahrie, M. A. Williams, J. Loscalzo and S. T. Weiss | 2016 | Early pregnancy vitamin D status and risk of preeclampsia |
| Moroti, R., R. Petre, I. Niculescu, I. Pigulea, V. Molagic, A. Hristea and A. Porojnicu | 2012 | Vitamin D an antimicrobial weapon against acute respiratory tract infections. A systematic review (2006- March 2011) |
| Nct | 2025 | Bisphosphonate Prior to Parathyroidectomy in Primary Hyperparathyroidism |
| Nelson, H. S. | 2018 | Immunotherapy for house-dust mite allergy |
| Nielsen, A. Y., S. Hoj, S. F. Thomsen and H. Meteran | 2024 | Vitamin D Supplementation for Treating Atopic Dermatitis in Children and Adults: A Systematic Review and Meta-Analysis |
| Nosrati, A., L. Afifi, M. J. Danesh, K. Lee, D. Yan, K. Beroukhim, R. Ahn and W. Liao | 2017 | Dietary modifications in atopic dermatitis: patient-reported outcomes |
| O Shea, P. M., T. P. Griffin, M. Brennan and E. C. Mulkerrin | 2020 | COVID-19: The older adult and the importance of vitamin D sufficiency |
| Obeid, R., W. Holzgreve and K. Pietrzik | 2019 | Folate supplementation for prevention of congenital heart defects and low birth weight: an update |
| Owczarek, B., A. Ziomkiewicz and E. Łukowska-Chojnacka | 2024 | Has a High Dose of Vitamin D3 Impacted Health Conditions in Older Adults?—A Systematic Review and Meta-Analysis Focusing on Dose 100,000 IU |
| Pacheco-González, R. M., L. García-Marcos and E. Morales | 2018 | Prenatal vitamin D status and respiratory and allergic outcomes in childhood: A meta-analysis of observational studies |
| Park, H. J., J. Y. Choi, W. M. Lee and S. M. Park | 2023 | Prevalence of chronic low back pain and its associated factors in the general population of South Korea: a cross-sectional study using the National Health and Nutrition Examination Surveys |
| Park, H. W., G. Lim, Y. M. Park, M. Chang, J. S. Son and R. Lee | 2020 | Association between vitamin D level and bronchopulmonary dysplasia: A systematic review and meta-analysis |
| Patrick, M. T., R. P. Nair, K. He, P. E. Stuart, A. C. Billi, X. Zhou, J. E. Gudjonsson, J. R. Oksenberg, J. T. Elder and L. C. Tsoi | 2023 | Shared Genetic Risk Factors for Multiple Sclerosis/Psoriasis Suggest Involvement of Interleukin-17 and Janus Kinase–Signal Transducers and Activators of Transcription Signaling |
| Persico, A. M., L. Asta, F. Chehbani, S. Mirabelli, V. Parlatini, S. Cortese, C. Arango and B. Vitiello | 2025 | The pediatric psychopharmacology of autism spectrum disorder: A systematic review - Part II: The future |
| Pilz, S., A. Zittermann, R. Obeid, A. Hahn, P. Pludowski, C. Trummer, E. Lerchbaum, F. R. Pérez-López, S. N. Karras and W. März | 2018 | The Role of Vitamin D in Fertility and during Pregnancy and Lactation: A Review of Clinical Data |
| Pioggia, G., A. Tonacci, G. Tartarisco, L. Billeci, F. Muratori, L. Ruta and S. Gangemi | 2014 | Autism and lack of D3 vitamin: A systematic review |
| Popp, A. W., J. Isenegger, E. M. Buergi, U. Buergi and K. Lippuner | 2006 | Glucocorticosteroid-induced spinal osteoporosis: Scientific update on pathophysiology and treatment |
| Pullar, J., K. Wickramasinghe, A. R. Demaio, N. Roberts, K. M. Perez-Blanco, K. Noonan and N. Townsend | 2019 | The impact of maternal nutrition on offspring's risk of non-communicable diseases in adulthood: a systematic review |
| Reinehr, T., D. Schnabel, M. Wabitsch, S. Bechtold-Dalla Pozzalla, C. Bührer, B. Heidtmann, F. Jochum, T. Kauth, A. Körner, W. Mihatsch, C. Prell, S. Rudloff, B. Tittel, J. Wölfle, K. P. Zimmer and B. Koletzko | 2018 | Vitamin D supplementation beyond the second year of life: Joint statement of the Nutrition Committee of the German Society for Pediatric and Adolescent Medicine (DGKJ) and the German Society for Pediatric Endocrinology and Diabetology (DGKED) |
| Roberts, C. L., S. L. Rushworth, E. Richman and J. M. Rhodes | 2013 | Hypothesis: Increased consumption of emulsifiers as an explanation for the rising incidence of Crohn's disease |
| Ruiz, M., A. Cocores, A. Tosti, P. J. Goadsby and T. S. Monteith | 2023 | Alopecia as an emerging adverse event to CGRP monoclonal antibodies: Cases Series, evaluation of FAERS, and literature review |
| Sarkar, M., Srinivasa, I. Madabhavi and K. Kumar | 2017 | Tuberculosis associated chronic obstructive pulmonary disease |
| Scragg, R. | 2018 | Emerging evidence of thresholds for beneficial effects from vitamin D supplementation |
| Shah, V. P., T. Nayfeh, Y. Alsawaf, S. Saadi, M. Farah, Y. Zhu, M. Firwana, M. Seisa, Z. Wang, R. Scragg, M. E. Kiely, P. Lips, D. M. Mitchell, M. B. Demay, A. G. Pittas and M. H. Murad | 2024 | A Systematic Review Supporting the Endocrine Society Clinical Practice Guidelines on Vitamin D |
| Shailaja, K., M. Sneha Mavis, S. Sowndharya, A. Tharani and P. Preethi | 2024 | The Effect of Vitamin D Supplementation in Various Diseases – A Systematic Review |
| Shaker, J. L. and B. P. Lukert | 2005 | Osteoporosis associated with excess glucocorticoids |
| Siekmeier, R., G. Delgado, L. Titze, T. Grammer, S. Pilz, M. Kleber and W. März | 2017 | Role of vitamin D deficiency in chronic obstructive pulmonary disease - An overview |
| Soe, H. H. K., A. B. L. Abas, N. N. Than, H. Ni, J. Singh, A. R. B. M. Said and I. Osunkwo | 2017 | Vitamin D supplementation for sickle cell disease |
| Soe, H. H. K., A. B. L. Abas, N. N. Than, H. Ni, J. Singh, A. R. B. M. Said and I. Osunkwo | 2020 | Vitamin D supplementation for sickle cell disease |
| Sova, C., M. B. Feuling, M. Baumler, L. Gleason, J. S. Tam, H. Zafra and P. S. Goday | 2013 | Systematic Review of Nutrient Intake and Growth in Children with Multiple IgE-Mediated Food Allergies |
| Stokes, P. J. and J. Rimmer | 2016 | The relationship between serum vitamin D and chronic rhinosinusitis: A systematic review |
| TePoel, M. R. W., A. F. Saftlas and A. B. Wallis | 2011 | Association of seasonality with hypertension in pregnancy: A systematic review |
| Tiihonen, M., H. Taipale, A. Tanskanen, J. Tiihonen and S. Hartikainen | 2016 | Incidence and Duration of Cumulative Bisphosphonate Use among Community-Dwelling Persons with or without Alzheimer's Disease |
| Tuchinda, P., K. Kulthanan, L. Chularojanamontri, S. Arunkajohnsak and S. Sriussadaporn | 2018 | Relationship between vitamin D and chronic spontaneous urticaria: a systematic review |
| Walker, K. C., F. Thorsteinsdottir, H. T. Christesen, V. E. Hjortdal, B. L. Heitmann, I. O. Specht and M. N. Händel | 2023 | Vitamin D Supplementation and Vitamin D Status during Pregnancy and the Risk of Congenital Anomalies—A Systematic Review and Meta-Analysis |
| Wang, X. Y., X. R. Li, Y. W. Shen and X. Y. Wang | 2018 | The association between serum vitamin D levels and urticaria: a meta-analysis of observational studies |
| Williams, H. C. and J. C. Chalmers | 2020 | Prevention of Atopic Dermatitis |
| Willits, E. K., Z. Wang, J. Jin, B. Patel, M. Motosue, A. Bhagia, J. Almasri, P. J. Erwin, S. Kumar and A. Y. Joshi | 2017 | Vitamin D and food allergies in children: A systematic review and meta-analysis |
| Wu, T., H. Cheng, J. Zhuang, X. Liu, Z. Ouyang and R. Qian | 2024 | Risk factors for inflammatory bowel disease: an umbrella review |
| Xu, Z., E. Forno, E. Acosta-Pérez, Y. Han, F. J. Rosser, M. L. Manni, G. Canino, W. Chen and J. C. Celedon | 2021 | Transcriptomics of obesity-related asthma in nasal epithelium from children and adolescents |
| Xu, Z., E. Forno, Y. Han, M. L. Manni, W. Chen and J. C. Celedon | 2022 | Transcriptomics of Total and Allergen-Specific IgE in Nasal Epithelium from Children and Adolescents |
| Yang, W. C., R. Chitale, K. M. O’Callaghan, C. R. Sudfeld and E. R. Smith | 2025 | The Effects of Vitamin D Supplementation During Pregnancy on Maternal, Neonatal, and Infant Health: A Systematic Review and Meta-analysis |
| Yeo, B. S. Y., E. J. Guan, K. Ng, Y. S. Lim, R. T. H. Goh, X. Liu, C. Q. Phua, K. Tay, L. H. Png, S. Xu, N. W. Y. Teo and T. C. Charn | 2025 | Association of Abnormal Body Weight and Allergic Rhinitis—A Systematic Review and Meta-Analysis |
| Yue, M., Z. Xu, Y. Han, F. J. Rosser, E. Acosta-Pérez, G. Canino, E. Forno, W. Chen and J. C. Celedon | 2022 | Genome-Wide Analysis of Gene Expression in Nasal Airway Epithelium and Children's Psychosocial Distress Symptoms |
| Zahra, M. and A. Leila | 2012 | Relationship between low Vitamin D and respiratory and bone density |
| Zaidi, A. Z., S. E. Moore and S. G. Okala | 2021 | Impact of maternal nutritional supplementation during pregnancy and lactation on the infant gut or breastmilk microbiota: A systematic review |
| Zeng, R., Y. S. Li, S. Y. Shen, X. Qiu, C. L. Chang, J. J. Koplin, K. P. Perrett, S. C. Dharmage, C. J. Lodge and A. J. Lowe | 2023 | Is antenatal or early-life vitamin D associated with eczema or food allergy in childhood? A systematic review |
| Zhou, J. H., F. Yuan, T. H. Huang, L. Zhu and D. W. Wu | 2023 | Current understanding of disease control and its application in patients with chronic rhinosinusitis |
| Zippi, M., S. Fiorino, R. Budriesi, M. Micucci, I. Corazza, R. Pica, D. de Biase, C. G. Gallo and W. Hong | 2021 | Paradoxical relationship between proton pump inhibitors and COVID-19: A systematic review and meta-analysis |
| Zittermann, A. | 2022 | THE IMPACT OF VITAMIN D ON CARDIOVASCULAR DISEASE, AND BEYOND |
| Zittermann, A., C. Trummer, V. Theiler-Schwetz and S. Pilz | 2023 | Long-term supplementation with 3200 to 4000 IU of vitamin D daily and adverse events: a systematic review and meta-analysis of randomized controlled trials |
| Zou, J. and W. Peng | 2024 | Unveiling the Knowledge Frontier: A Scientometric Analysis of COPD with Sarcopenia |
| 顾敏敬. | 2019 | 维生素D辅助治疗婴幼儿毛细支气管炎的META分析 硕士, 重庆医科大学. |
| 何方婷 . | 2022 | 花椒籽油抗骨质疏松的作用及机制研究 博士, 四川大学. |
| 胡冀生. | 2024 | 孕期维生素D水平与子代过敏性疾病发生风险的Meta分析 硕士, 河北医科大学. |
| 黄金龙. | 2016 | 基于蒙定水教授“金水相生”法治疗老年高血压病的临床观察及作用机理探讨 博士, 广州中医药大学. |
| 刘丹青. | 2018 | 活性维生素D对D-半乳糖诱导的老年雄性大鼠睾丸功能影响的研究 硕士, 郑州大学. |
| 王姝, 李泉波 and 冯晓玲. | 2023 | 叶酸补充过量与生殖风险相关性的研究进展 |
| 王雪琪. | 2022 | 维生素D与儿童社区获得性肺炎相关性研究的meta分析 硕士, 内蒙古医科大学. |
| 韦桂丽. | 2018 | 菲牛蛭提取物对黑色素瘤细胞的影响及抗氧化活性和安全性评价 硕士, 广西中医药大学 |
| 种树彬. | 2008 | 复方颠倒散药物面膜的皮肤毒理学及临床应用研究 硕士, 南方医科大学. |

2 Intervention measures for the treatment group or control group that do not meet the requirements

| Abreo, A., T. Gebretsadik, C. A. Stone and T. V. Hartert | 2018 | The impact of modifiable risk factor reduction on childhood asthma development |
| --- | --- | --- |
| Aghili, S. M. M., M. Ebrahimpur, B. Arjmand, Z. Shadman, M. Pejman Sani, M. Qorbani, B. Larijani and M. Payab | 2021 | Obesity in COVID-19 era, implications for mechanisms, comorbidities, and prognosis: a review and meta-analysis |
| Baek, H., M. Han and J. H. Kim | 2023 | Association of serum vitamin D level measured in childhood with asthma incidence: retrospective multicenter cohort study using six real-world databases |
| Beckhaus, A. A., J. A. Castro-Rodriguez, M. A. Oyarzun, R. Pacheco-Gonzalez, E. Forno, J. C. Celedon and L. Garcia-Marcos | 2015 | Maternal nutrition during pregnancy and risk of asthma, wheeze and atopic diseases during childhood: Systematic review and meta-analysis |
| Beckhaus, A. A., L. Garcia-Marcos, E. Forno, R. M. Pacheco-Gonzalez, J. C. Celedõn and J. A. Castro-Rodriguez | 2015 | Maternal nutrition during pregnancy and risk of asthma, wheeze, and atopic diseases during childhood: A systematic review and meta-analysis |
| Buelo, A., S. McLean, J. Flores-Kim, S. Julious, M. Shields, J. Paton, J. Henderson, A. Bush and H. Pinnock | 2017 | Identifying the child (5-12 years) with asthma at increased risk of attacks: The at-risk child with asthma (ARC) systematic review |
| Buelo, A., S. McLean, S. Julious, J. Flores-Kim, A. Bush, J. Henderson, J. Y. Paton, A. Sheikh, M. Shields and H. Pinnock | 2018 | At-risk children with asthma (ARC): A systematic review |
| Buendía, J. A., R. Acuña-Cordero and D. G. Patiño | 2023 | The role of high carbohydrate-rich food intake and severity of asthma exacerbation in children between 2 to 6 years aged |
| Bukhari, A. A. S., A. R. K. Shaikh, W. Salman, F. A. Bhatti, W. Malik, M. Minhas, A. Muddasser and H. Khaliq | 2025 | Pathophysiological role of nerve growth factor (NGF) in asthma: insights into airway inflammation, remodeling, and neural regulation in intensive care settings |
| Byrnes, C. | 2017 | Maintaining respiratory health in resource poor populations |
| Calamelli, E., P. Bottau and M. Lanari | 2018 | Update on Interventions in Prevention and Treatment of Pediatric Asthma |
| Calhoun, K. H. | 2015 | Asthma treatments: New and emerging therapies |
| Chatzi, L., R. Garcia, T. Roumeliotaki, M. Basterrechea, H. Begiristain, C. Iñiguez, J. Vioque, M. Kogevinas, J. Sunyer, I. S. Grp and R. S. Grp | 2013 | Mediterranean diet adherence during pregnancy and risk of wheeze and eczema in the first year of life: INMA (Spain) and RHEA (Greece) mother-child cohort studies |
| Chen, Y. L., A. Checa, P. Zhang, M. N. Huang, R. S. Kelly, M. Kim, Y. C. S. Chen, K. A. Lee-Sarwar, N. Prince, K. M. Mendez, S. Begum, P. Kachroo, S. H. Chu, J. Stokholm, K. Bonnelykke, A. A. Litonjua, H. Bisgaard, S. T. Weiss, B. L. Chawes, C. E. Wheelock and J. A. Lasky-Su | 2024 | Sphingolipid classes and the interrelationship with pediatric asthma and asthma risk factors |
| Chien, M. C., C. Y. Huang, J. H. Wang, C. L. Shih and P. Wu | 2024 | Effects of vitamin D in pregnancy on maternal and offspring health-related outcomes: An umbrella review of systematic review and meta-analyses |
| Danielewicz, H. | 2014 | What the Genetic Background of Individuals with Asthma and Obesity Can Reveal: Is β2-Adrenergic Receptor Gene Polymorphism Important? |
| Ferrante, G., G. Piacentini, M. Piazza, A. L. Boner and J. A. Bellanti | 2024 | Addressing global health disparities in the management of RSV infection in infants and children: Strategies for preventing bronchiolitis and post-bronchiolitis recurrent wheezing |
| Forno, E., O. M. Young, R. Kumar, H. Simhan and J. C. Celedón | 2014 | Maternal Obesity in Pregnancy, Gestational Weight Gain, and Risk of Childhood Asthma |
| Garcia-Larsen, V., S. R. Del Giacco, A. Moreira, M. Bonini, D. Charles, T. Reeves, K. H. Carlsen, T. Haahtela, S. Bonini, J. Fonseca, I. Agache, N. G. Papadopoulos and L. Delgado | 2016 | Asthma and dietary intake: an overview of systematic reviews |
| Goldstein, M. F., J. J. Fallon Jr and R. Harning | 1999 | Chronic glucocorticoid therapy-induced osteoporosis in patients with obstructive lung disease |
| Golec, M., M. K. Lemieszek, J. Dutkiewicz, J. Milanowski and S. Barteit | 2022 | A Scoping Analysis of Cathelicidin in Response to Organic Dust Exposure and Related Chronic Lung Illnesses |
| Gray, K. W. | 2021 | Pediatric Asthma for the Primary Care Physician |
| Ibrahim, W., S. Natarajan, M. Wilde, R. Cordell, P. S. Monks, N. Greening, C. E. Brightling, R. Evans and S. Siddiqui | 2021 | A systematic review of the diagnostic accuracy of volatile organic compounds in airway diseases and their relation to markers of type-2 inflammation |
| Irwin, R. S. and N. D. Richardson | 2006 | Side effects with inhaled corticosteroids: The physician's perception |
| Jain, R. P., D. Als, T. Vaivada and Z. A. Bhutta | 2022 | Prevention and Management of High-Burden Noncommunicable Diseases in School-Age Children: A Systematic Review |
| Jartti, T., K. Bønnelykke, V. Elenius and W. Feleszko | 2020 | Role of viruses in asthma |
| Jiang, Y., E. Forno, Y. Y. Han, Z. Xu, D. Hu, N. Boutaoui, C. Eng, E. Acosta-Pérez, S. Huntsman, A. Colón-Semidey, K. L. Keys, J. R. Rodríguez-Santana, M. Alvarez, M. Pino-Yanes, G. Canino, W. Chen, E. G. Burchard and J. C. Celedón | 2021 | A genome-wide study of DNA methylation in white blood cells and asthma in Latino children and youth |
| Jung, J. W., H. R. Kang, J. Y. Kim, S. H. Lee, S. S. Kim and S. H. Cho | 2014 | Are asthmatic patients prone to bone loss? |
| KIMUTAI, F. | 2021 | 射干的化学成分及其抗氧化和抗炎活性的研究 硕士, 中国科学院大学. |
| Kiss, M., Z. Czimmerer and L. Nagy | 2013 | The role of lipid-activated nuclear receptors in shaping macrophage and dendritic cell function: From physiology to pathology |
| Kohn, C. M. and P. Paudyal | 2017 | A systematic review and meta-analysis of complementary and alternative medicine in asthma |
| Larenas-Linnemann, D. E. S., D. R. Pietropaolo-Cienfuegos and M. A. Calderón | 2011 | Evidence of effect of subcutaneous immunotherapy in children: Complete and updated review from 2006 onward |
| Li, N., X. T. Wu, W. Zhuang, C. C. Wu, Z. Y. Rao, L. Du and Y. Zhou | 2022 | Cruciferous vegetable and isothiocyanate intake and multiple health outcomes |
| Liu, T., M. Yue, K. M. Gaietto, F. J. Rosser, C. Qoyawayma, W. Chen and J. C. Celedon | 2025 | Nasal Epithelial Expression of RNA Isoforms in Youth With T2-high and T2-low Asthma |
| Liu, T., M. Yue, F. J. Rosser, S. Kim, K. M. Gaietto, E. Forno, Y. Han, W. Chen and J. C. Celedon | 2025 | A Genome-wide Study of Nasal Epithelial DNA Methylation and Asthma Endotypes |
| Machado, M. E., L. C. Porto, M. G. Alves Galvão, C. C. Sant’Anna and J. R. Lapa e Silva | 2023 | SNPs, adipokynes and adiposity in children with asthma |
| McEvoy, C. T., D. Schilling, N. Clay, K. Jackson, M. D. Go, P. Spitale, C. Bunten, M. Leiva, D. Gonzales, J. Hollister-Smith, M. Durand, B. Frei, A. S. Buist, D. Peters, C. D. Morris and E. R. Spindel | 2014 | Vitamin C Supplementation for Pregnant Smoking Women and Pulmonary Function in Their Newborn Infants A Randomized Clinical Trial |
| Mikkelsen, H., E. M. Landt, M. Benn, B. G. Nordestgaard and M. Dahl | 2022 | Causal risk factors for asthma in Mendelian randomization studies: A systematic review and meta-analysis |
| Nct | 2024 | Aging Resilience Through Microbiota Optimization and Regulation |
| Netting, M., P. Middleton and M. Makrides | 2013 | Does maternal diet during pregnancy and lactation affect allergy outcomes in their offspring? A systematic review of food based approaches |
| Netting, M. J., P. F. Middleton and M. Makrides | 2014 | Does maternal diet during pregnancy and lactation affect outcomes in offspring? A systematic review of food-based approaches |
| Nurmatov, U., G. Devereux and A. Sheikh | 2010 | Nutrients and foods for the primary prevention of asthma and allergic disorders: A systematic review and meta-analysis |
| Nurmatov, U., G. Devereux and A. Sheikh | 2011 | Nutrients and foods for the primary prevention of asthma and allergy: Systematic review and meta-analysis |
| Nurmatov, U., B. I. Nwaru, G. Devereux and A. Sheikh | 2012 | Confounding and effect modification in studies of diet and childhood asthma and allergies |
| Omidkhoda, S. F. and H. Hosseinzadeh | 2022 | Saffron and its active ingredients against human disorders: A literature review on existing clinical evidence |
| Posadzki, P., A. Alotaibi and E. Ernst | 2012 | Adverse effects of homeopathy: A systematic review of published case reports and case series |
| Reichardt, S. D., A. Amouret, C. Muzzi, S. Vettorazzi, J. P. Tuckermann, F. Lühder and H. M. Reichardt | 2021 | The role of glucocorticoids in inflammatory diseases |
| Rice, J. B., A. G. White, L. M. Scarpati, G. Wan and W. W. Nelson | 2017 | Long-term Systemic Corticosteroid Exposure: A Systematic Literature Review |
| Ringe, J. D. and P. Farahmand | 2007 | Advances in the management of corticosteroid-induced osteoporosis with bisphosphonates |
| Robijn, A. L., M. P. Bokern, M. E. Jensen, D. Barker, K. J. Baines and V. E. Murphy | 2022 | Risk factors for asthma exacerbations during pregnancy: a systematic review and meta-analysis |
| Robison, R. and R. Kumar | 2010 | The effect of prenatal and postnatal dietary exposures on childhood development of atopic disease |
| Rosser, F. J., M. Yue, Y. Han, E. Forno, M. L. Manni, C. Qoyawayma, E. Acosta Perez, G. Canino, W. Chen and J. C. Celedon | 2024 | Long-term PM2.5 Exposure and Nasal Airway Epithelial Expression of CLCA1 in Youth With and Without Asthma |
| Rosser, F. J., M. Yue, Y. Y. Han, E. Forno, C. Qoyawayma, M. L. Manni, E. Acosta-Pérez, G. Canino, W. Chen and J. C. Celedón | 2025 | Long-Term PM2.5 Exposure and Upregulation of CLCA1 Expression in Nasal Epithelium from Youth with Asthma |
| Ruan, Z., Z. L. Shi, G. C. Zhang, J. S. Kou and H. Ding | 2020 | Asthma susceptible genes in children A meta-analysis |
| Saag, K. G. | 2003 | Glucocorticoid-induced osteoporosis |
| Saller, R., C. Römer-Lüthi, M. Müller, R. Brignoli, G. Noll and R. Meier | 2006 | Docosahexaenoic acid (DHA) and long chain omega-3 fatty acids: Clinical relevance in inflammatory and other dieases |
| Sausenthaler, S., B. Koletzko and J. Heinrich | 2006 | Dietary fat intake and allergic diseases |
| Savage, J. and C. Keet | 2011 | Nutrients and foods for the primary prevention of asthma and allergy: Systematic review and meta-analysis |
| Shah, J. L., I. L. C. Shadid, V. J. Carey, N. Laranjo, G. T. O'Connor, R. S. Zeiger, L. Bacharier, A. A. Litonjua, S. T. Weiss and H. Mirzakhani | 2023 | Early-Life Weight Status and Risk of Childhood Asthma or Recurrent Wheeze in Preterm and Term Offspring |
| Song, W. J., S. H. Kim, S. Lim, Y. J. Park, M. H. Kim, S. M. Lee, S. B. Lee, K. W. Kim, H. C. Jang, S. H. Cho, K. U. Min and Y. S. Chang | 2012 | Association between obesity and asthma in the elderly population: potential roles of abdominal subcutaneous adiposity and sarcopenia |
| Sposato, B. | 2013 | Could FEV1 decline have a role in daily clinical practice for asthma monitoring? |
| Staresinic, A. G. and C. A. Sorkness | 2002 | The use of inhaled corticosteroids in adult asthma |
| Susanto, N. H., D. Vicendese, A. Salim, A. J. Lowe, S. C. Dharmage, R. Tham, C. Lodge, F. Gardene, K. Allen, C. Svanes, J. Heinrich, M. J. Abramson and B. Erbas | 2017 | Effect of season of birth on cord blood IgE and IgE at birth: A systematic review and meta-analysis |
| Taghavizadeh Yazdi, M. E., M. Qayoomian, S. Beigoli and M. H. Boskabady | 2023 | Recent advances in nanoparticle applications in respiratory disorders: a review |
| Tamari, M. and T. Hirota | 2013 | Genome-wide association studies of asthma and atopic dermatitis in the Japanese population |
| Theodosiou, A. A., R. B. Dorey, J. R. Laver, D. W. Cleary, R. C. Read and C. E. Jones | 2021 | Manipulating the infant respiratory microbiomes to improve clinical outcomes: A review of the literature |
| Tizaoui, K., K. Hamzaoui and A. Hamzaoui | 2017 | Update on asthma genetics: Results from meta-analyses of candidate gene association studies |
| Tonacci, A., L. Billeci, L. Ruta, G. Tartarisco, G. Pioggia and S. Gangemi | 2017 | A systematic review of the association between allergic asthma and autism |
| Tu, W., X. J. Xiao, J. H. Lu, X. Y. Liu, E. R. Y. Wang, R. Y. Yuan, R. J. Wan, Y. C. Shen, D. M. Xu, P. C. Yang, M. Gong, P. S. Gao and S. K. Huang | 2023 | Vanadium exposure exacerbates allergic airway inflammation and remodeling through triggering reactive oxidative stress |
| Vahid, F. and D. Rahmani | 2021 | Can an anti-inflammatory diet be effective in preventing or treating viral respiratory diseases? A systematic narrative review |
| van Brakel, L., R. P. Mensink, G. Wesseling and J. Plat | 2020 | Nutritional interventions to improve asthma-related outcomes through immunomodulation: A systematic review |
| Vemer, P. and M. P. M. H. Rutten-Van Mölken | 2013 | The road not taken: Transferability issues in multinational trials |
| Venter, C., C. Agostoni, S. H. Arshad, M. Ben-Abdallah, G. Du Toit, D. M. Fleischer, M. Greenhawt, D. H. Glueck, M. Groetch, N. Lunjani, K. Maslin, A. Maiorella, R. Meyer, M. Antonella, M. J. Netting, B. Ibeabughichi Nwaru, D. J. Palmer, M. P. Palumbo, G. Roberts, C. Roduit, P. Smith, E. Untersmayr, L. A. Vanderlinden and L. O’Mahony | 2020 | Dietary factors during pregnancy and atopic outcomes in childhood: A systematic review from the European Academy of Allergy and Clinical Immunology |
| Venter, C., H. Arshad, K. Maslin, R. Meyer, M. Groetch, M. Palumbo, L. Vanderlinden, M. BenAbdallah, M. Greenhawt and D. Fleischer | 2020 | A comprehensive systematic review of the impact of dietary factors during pregnancy on childhood atopic outcomes |
| Vinding, R. K., T. S. Sejersen, B. L. Chawes, K. Bónnelykke, T. Buhl, H. Bisgaard and J. Stokholm | 2017 | Cesarean delivery and body mass index at 6 months and into childhood |
| Visser, E., A. ten Brinke, D. Sizoo, J. J. S. Pepels, L. ten Have, E. van der Wiel, T. van Zutphen, H. A. M. Kerstjens and K. de Jong | 2024 | Effect of dietary interventions on markers of type 2 inflammation in asthma: A systematic review |
| Wang, S., P. Yin, L. Yu, F. Tian, W. Chen and Q. Zhai | 2024 | Effects of Early Diet on the Prevalence of Allergic Disease in Children: A Systematic Review and Meta-Analysis |
| Weldon, D. | 2009 | The effects of corticosteroids on bone growth and bone density |
| Wu, J. H., Y. Yu, X. M. Yao, Q. Z. Zhang, Q. Zhou, W. H. Tang, X. L. Huang and C. Y. Ye | 2022 | Visualizing the knowledge domains and research trends of childhood asthma: A scientometric analysis with CiteSpace |
| Xu, Z., E. Forno, E. Acosta-Pérez, Y. Y. Han, F. Rosser, M. L. Manni, G. Canino, W. Chen and J. C. Celedón | 2022 | Differential gene expression in nasal airway epithelium from overweight or obese youth with asthma |
| Yang, F., J. Zhu, Z. Wang, L. Wang, T. Tan and L. Sun | 2022 | Relationship between maternal folic acid supplementation during pregnancy and risk of childhood asthma: Systematic review and dose-response meta-analysis |
| Yepes-Nuñez, J. J., J. L. Brożek, A. Fiocchi, R. Pawankar, C. Cuello-García, Y. Zhang, G. P. Morgano, A. Agarwal, S. Gandhi, L. Terracciano and H. J. Schünemann | 2018 | Vitamin D supplementation in primary allergy prevention: Systematic review of randomized and non-randomized studies |
| Yetmar, Z. A., S. Chesdachai, T. Kashour, M. Riaz, D. J. Gerberi, A. D. Badley, E. F. Berbari and I. M. Tleyjeh | 2021 | Prior Statin Use and Risk of Mortality and Severe Disease from Coronavirus Disease 2019: A Systematic Review and Meta-analysis |
| Yin, L., F. F. Zhang, X. Wang, M. M. Gao, A. N. Liu and F. Li | 2025 | Prevalence and Risk Factors of Asthma in Children: A Systematic Review and Meta-analysis |
| Yue, M., K. Gaietto, Y. Y. Han, F. J. Rosser, Z. Xu, C. Qoyawayma, E. Acosta-Perez, G. Canino, E. Forno, W. Chen and J. C. Celedón | 2025 | Transcriptomic Profiles in Nasal Epithelium and Asthma Endotypes in Youth |
| Yue, M., Z. Xu, Y. Han, F. J. Rosser, E. Acosta-Pérez, G. Canino, E. Forno, W. Chen and J. C. Celedon | 2023 | Airway Epithelial Expression of CCL8, CXCL11, FLG2, and CXCL9 Is Associated With Violence-related Distress and Atopic Asthma in Youth |
| Yue, M. L., K. Gaietto, Y. Y. Han, F. J. Rosser, Z. L. Xu, C. Qoyawayma, E. Acosta-Perez, G. Canino, E. Forno, W. Chen and J. C. Celedon | 2025 | Transcriptomic Profiles in Nasal Epithelium and Asthma Endotypes in Youth |
| Zhang, L., S. Zhang, C. He and X. Wang | 2020 | VDR Gene Polymorphisms and Allergic Diseases: Evidence from a Meta-analysis |
| 阿萨. | 2013 | 白藜芦醇通过调节抗氧化酶活性而呈现抗肿瘤效应 博士, 中南大学. |
| 陈沁竹. | 2015 | 维生素A补充对哮喘、过敏反应及肺功能影响的meta分析 硕士, 重庆医科大学 |
| 陈沁竹 and 徐王国 | 2020 | 维生素A补充与变应性疾病相关性的Meta分析 |
| 李利寻. | 2022 | 中成药真实世界研究：以喜炎平注射液治疗儿童肺炎个体化用药方案推荐为例 硕士, 中国中医科学院 |
| 刘艳. | 2013 | γ-维生素E对哮喘小鼠血清及肺泡灌洗液eotaxin、IFn-γ的影响 硕士, 中国医科大学 |
| 刘以撒. | 2022 | 基于LC-MS的代谢组学探究罗汉果润肺止咳的疗效 硕士, 桂林医学院 |
| 苏艳艳. | 2018 | 母孕期使用抗生素与后代患哮喘风险的Meta分析 硕士, 重庆医科大学 |
| 吴军, 张平, 周波, 匡嘉丽 and 郭亮 | 2009 | 变应性鼻炎、哮喘与饮食非过敏因素的循证医学相关研究 |
| 杨二兰. | 2020 | 马齿苋中儿茶酚型四氢异喹啉类生物碱的合成及其抗哮喘和抗炎活性研究 硕士, 山东大学 |
| 姚应水. | 2015 | 哮喘患病的环境因素系统评价 博士, 安徽理工大学 |
| 张潮. | 2018 | 益肺固表方联合匹多莫德治疗儿童反复呼吸道感染的效果评价 |

3 Animal Studies

| Hufnagl, K. and E. Jensen-Jarolim | 2018 | Vitamin A and D in allergy: From experimental animal models and cellular studies to human disease |
| --- | --- | --- |
| 李沁原. | 2024 | 1，25-二羟维生素D3调控GSN表达在哮喘气道上皮细胞凋亡中的作用及机制研究 博士, 重庆医科大学 |
| 尚帅. | 2016 | 氧化石墨烯加重小鼠过敏性哮喘机制的研究 硕士, 华中师范大学. |
| 宋颖芳, 柏长青, 戚好文 and 吴昌归 | 2008 | 1,25-二羟维生素D3对哮喘小鼠气道重塑及基质金属蛋白酶-9表达的影响 |
| 宋颖芳, 洪景芳, 柳德灵, 林庆安 and 赖国祥 | 2012 | 1,25-二羟维生素D3对哮喘气道重塑小鼠模型NF-κB信号通路的调控研究 |
| 宋颖芳, 洪景芳, 柳德灵, 林庆安, 叶嘉, 廖云海 and 赖国祥 | 2013 | 1,25-二羟维生素D3对慢性哮喘小鼠气道重塑中MMP-9及NF-κB表达的影响 |
| 宋颖芳, 赖国祥, 柳德灵 and 林庆安 | 2012 | 1,25-二羟维生素D3抑制慢性哮喘模型小鼠肺组织α-滑肌肌动蛋白的表达及气道重塑 |
| 宋颖芳, 赖国祥, 戚好文 and 吴昌归 | 2011 | 1,25-二羟维生素D2减轻哮喘小鼠的气道重塑 |
| 宋颖芳, 戚好文 and 吴昌归. | 2008 | 1,25-二羟维生素D3对哮喘小鼠气道重塑及基质金属蛋白酶-9表达的影响. 中华医学会第六届全国哮喘学术会议暨中国哮喘联盟第二次大会论文集. 苏州: 300-303 |
| 宋颖芳, 戚好文 and 吴昌归 | 2008 | 1,25-二羟维生素D3对支气管哮喘小鼠气道重塑及基质金属蛋白酶9表达的影响 |
| 谢君谋. | 2020 | 维生素D联合DNase I对中性粒细胞性哮喘小鼠抗炎作用的研究 硕士, 广州医科大学 |
| 张鹤. | 2020 | 1，25--二羟维生素D3通过TGF--β1/Smad2/3影响ROS调节气道重塑 硕士, 西南医科大学. |
| 张鹤, 张沄 and 王宋平 | 2020 | 1,25-二羟维生素D3通过TGF-β1/(Smad2/3)影响的ROS调节气道重塑 |

4 Clinical Research

|  | 2016 | A Randomized Control Trial of Vitamin D Prophylaxis in the Prevention of Hypertensive Disorders of Pregnancy |
| --- | --- | --- |
|  | 2016 | Vitamin D to Prevent Severe Asthma Exacerbations. |
|  | 2017 | Project 500 CHILD Study |
|  | 2021 | High-dose Vitamin D Supplement for the Prevention of Acute Asthma-like Symptoms in Preschool Children - a Double-blind, Randomized, Controlled Trial |
| Abdul-Razzak, J., M. Ionescu, R. Diaconu, A. D. Popescu, E. C. Niculescu, I. O. Petrescu, C. E. Singer, L. Radu, L. Anghelina and C. Gheonea | 2025 | Effect of COVID-19 Disease on Serum Vitamin D Status in Children with Asthma-A Retrospective Study |
| Al-Qerem, W., A. Jarab, Y. Jarrar, E. Al-Zayadneh, M. Al-Iede, J. Ling, K. Abu Hammour, S. S. Alabdullah, A. S. Alabdullah, Y. Al Refaie, D. Lubbad, A. Alassi, S. Ibrahim, M. Al-Ibadah and A. Q. Al Bawab | 2024 | Correlation of vitamin D receptor genotypes, specific IgE levels and other variables with asthma control in children |
| Batmaz, S. B., T. Arikoglu, N. Uyar, I. Barlas and S. Kuyucu | 2017 | The Effect of Vitamin D Pathway Genes on Asthma Susceptibility, Asthma Control and Vitamin D Levels in Turkish Asthmatic Children |
| Bindayel, I. A. | 2021 | Effect of age and body mass index on vitamin D level in children with asthma in Riyadh |
| Elevli, M., A. E. Bozaci, K. Sahin, H. N. Duru, M. Çivilibal and B. B. Aktas | 2018 | Evaluation of serum 25-hidroxy vitamin D and zinc levels in asthmatic patients |
| Ganji, V., A. Al-Obahi, S. Yusuf, Z. Dookhy and Z. M. Shi | 2020 | Serum vitamin D is associated with improved lung function markers but not with prevalence of asthma, emphysema, and chronic bronchitis |
| Gonsard, A., F. Marquant, C. Elie, M. Le Bourgeois, V. Houdouin, C. Delclaux, N. Beydon, A. Bellino, J. C. Souberbielle, I. Sermet-Gaudelus, C. Delacourt, A. Benachi and A. Hadchouel | 2025 | Specific airway resistance according to early maternal vitamin D status during pregnancy in children aged 5 to 6 years old from the FEPED cohort (RESPIFEPED) |
| Hart, P. H., R. M. Lucas, J. P. Walsh, G. R. Zosky, A. J. O. Whitehouse, K. Zhu, K. L. Allen, M. M. Kusel, D. Anderson and J. A. Mountain | 2015 | Vitamin D in Fetal Development: Findings From a Birth Cohort Study |
| Jat, K. R., N. Goel, N. Gupta, C. P. Gupta, S. Datta, R. Lodha and S. K. Kabra | 2020 | Efficacy of vitamin D supplementation in asthmatic children with vitamin D deficiency: A randomized controlled trial (ESDAC trial) |
| Jolliffe, D. A., C. Stefanidis, Z. C. Wang, N. Z. Kermani, V. Dimitrov, J. H. White, J. E. McDonough, W. Janssens, P. Pfeffer, C. J. Griffiths, A. Bush, Y. K. Guo, S. Christenson, I. M. Adcock, K. F. Chung, K. E. Thummel and A. R. Martineau | 2020 | Vitamin D Metabolism Is Dysregulated in Asthma and Chronic Obstructive Pulmonary Disease |
| Li, J., A. Tiwari, H. Mirzakhani, A. L. Wang, A. T. Kho, M. J. McGeachie, A. A. Litonjua, S. T. Weiss and K. G. Tantisira | 2021 | Circulating microrna: Incident asthma prediction and vitamin d effect modification |
| Lim, M. T., T. J. Lee, M. B. Ramamurthy and D. Y. Goh | 2018 | Vitamin D levels in asthmatic and healthy children in Singapore |
| Malheiro, A. P. G., L. Gianfrancesco, R. J. N. Nogueira, M. B. Grotta, A. M. Morcillo, J. D. Ribeiro and A. Toro | 2023 | Association between serum Vitamin D levels and asthma severity and control in children and adolescents |
| Mendez, K., M. Cote, M. Huang, R. S. Kelly, P. Kachroo, S. H. Chu, D. I. Soeteman, S. N. Reinke, C. E. Wheelock, J. C. Celedon, C. B. Clish, S. T. Weiss and J. A. Lasky-Su | 2021 | Metabolomic analysis of asthma phenotypes across multiple cohorts |
| Rojo-Tolosa, S., L. E. Pineda-Lancheros, J. M. Gálvez-Navas, J. A. Sánchez-Martínez, M. V. González-Gutiérrez, A. Fernández-Alonso, C. Morales-García, A. Jiménez-Morales and C. Pérez-Ramírez | 2023 | Association between Single Nucleotide Polymorphisms Related to Vitamin D Metabolism and the Risk of Developing Asthma |
| Sarioglu, N., A. D. Yalcin, F. Sahin, S. Soyyigit, F. M. Tepetam and F. Erel | 2021 | Does vitamin D deficiency in asthma affect clinical and functional parameters? A Turkish multicenter study |
| Sung, M. | 2023 | Trends of vitamin D in asthma in the pediatric population for two decades: a systematic review |
| Wan, M., L. J. Horsfall, E. Basatemur, J. P. Patel, R. Shroff and G. Rait | 2019 | Vitamin D prescribing in children in UK primary care practices: a population-based cohort study |
| Wang, Q., Q. Ying, W. Zhu and J. Chen | 2022 | Vitamin D and asthma occurrence in children: A systematic review and meta-analysis |
| Watkins, S., T. Harrison and S. Mushtaq | 2024 | A 12-week double-blind randomised controlled trial investigating the effect of dietary supplementation with 5000 μg/d (125 μg/d) vitamin D in adults with asthma |
| Xu, J. and J. L. Tang | 2025 | Associations between asthma and Life's Essential 8: a cross-sectional study |
| Zaçe, D., E. L. Gatta, A. Orfino, A. M. Viteritti and M. L. Di Pietro | 2022 | Knowledge, attitudes, and health status of childbearing age young women regarding preconception health - an Italian survey |
| Zhu, Y. Q., D. R. Jing, H. Y. Liang, D. W. Li, Q. Y. Chang, M. X. Shen, P. H. Pan, H. Liu and Y. Zhang | 2022 | Vitamin D status and asthma, lung function, and hospitalization among British adults |

| Salameh, L., W. Mahmood, R. Hamoudi, K. Almazrouei, M. Lochanan, S. Seyhoglu and B. Mahboub | 2023 | The Role of Vitamin D Supplementation on Airway Remodeling in Asthma: A Systematic Review |
| --- | --- | --- |

5 In vitro experiment

| Salameh, L., W. Mahmood, R. Hamoudi, K. Almazrouei, M. Lochanan, S. Seyhoglu and B. Mahboub | 2023 | The Role of Vitamin D Supplementation on Airway Remodeling in Asthma: A Systematic Review |
| --- | --- | --- |

6 Repetition

| Anitua, E., R. Tierno and M. H. Alkhraisat | 2022 | Current opinion on the role of vitamin D supplementation in respiratory infections and asthma/COPD exacerbations: A need to establish publication guidelines for overcoming the unpublished data |
| --- | --- | --- |
| Anitua, E., R. Tierno and M. H. Alkhraisat | 2022 | Current opinion on the role of vitamin D supplementation in respiratory infections and asthma/COPD exacerbations: A need to establish publication guidelines for overcoming the unpublished data |
| Autier, P., P. Mullie, A. Macacu, M. Dragomir, M. Boniol, K. Coppens, C. Pizot and M. Boniol | 2017 | Effect of vitamin D supplementation on non-skeletal disorders: a systematic review of meta-analyses and randomised trials |
| Beauchesne, A. R., K. C. Cara, D. M. Krobath, L. P. Penkert, S. P. Shertukde, D. S. Cahoon, B. Prado, R. G. Li, Q. S. Yao, J. Huang, T. Reh and M. Chung | 2022 | Vitamin D intakes and health outcomes in infants and preschool children: Summary of an evidence report |
| Beckhaus, A. A., L. Garcia-Marcos, E. Forno, R. M. Pacheco-Gonzalez, J. C. Celedón and J. A. Castro-Rodriguez | 2015 | Maternal nutrition during pregnancy and risk of asthma, wheeze, and atopic diseases during childhood: a systematic review and meta-analysis |
| Buelo, A., S. McLean, S. Julious, J. Flores-Kim, A. Bush, J. Henderson, J. Y. Paton, A. Sheikh, M. Shields, H. Pinnock and A. R. C. Grp | 2018 | At-risk children with asthma (ARC): a systematic review |
| Cannell, J. J. and B. W. Hollis | 2008 | Use of vitamin D in clinical practice |
| Cassim, R., M. A. Russell, C. J. Lodge, A. J. Lowe, J. J. Koplin and S. C. Dharmage | 2015 | The role of circulating 25 hydroxyvitamin D in asthma: a systematic review |
| Cassim, R., M. A. Russell, C. J. Lodge, A. J. Lowe, J. J. Koplin and S. C. Dharmage | 2015 | The role of circulating 25 hydroxyvitamin D in asthma: a systematic review |
| Chaudhary, U. and S. S. H. Gardezi | 2025 | Letter to the editor regarding "vitamin D supplementation decrease asthma exacerbations in children: a systematic review and meta-analysis of randomized controlled trials" |
| Chawes, B. L., H. Wolsk, A. A. Litonjua, B. W. Hollis, K. Bonnelykke, J. Waage, H. Bisgaard and S. Weiss | 2017 | Prenatal Vitamin D Supplementation Reduces Risk Of Asthma/recurrent Wheeze In Early Childhood: A Meta-Analysis Of Two Randomized Controlled Trials |
| Chawes, B. L., H. Wolsk, A. A. Litonjua, B. W. Hollis, K. Bonnelykke, J. Waage, H. Bisgaard and S. Weiss | 2017 | Prenatal vitamin d supplementation reduces risk of asthma/recurrent wheeze in early childhood: a meta-analysis of two randomized controlled trials |
| Chen, Z., C. Peng, J. Mei, L. Zhu and H. Kong | 2021 | Vitamin D can safely reduce asthma exacerbations among corticosteroid-using children and adults with asthma: a systematic review and meta-analysis of randomized controlled trials |
| Chen, Z. Y., C. Peng, J. Y. Mei, L. F. Zhu and H. Kong | 2021 | Vitamin D can safely reduce asthma exacerbations among corticosteroid-using children and adults with asthma: a systematic review and meta-analysis of randomized controlled trials |
| Curtis, J. R. and K. G. Saag | 2007 | Prevention and treatment of glucocorticoid-induced osteoporosis |
| El Abd, A., H. Dasari, P. Dodin, H. Trottier and F. M. Ducharme | 2024 | Associations between vitamin D status and biomarkers linked with inflammation in patients with asthma: a systematic review and meta-analysis of interventional and observational studies |
| El Abd, A., H. Dasari, P. Dodin, H. Trottier and F. M. Ducharme | 2024 | Associations between vitamin D status and biomarkers linked with inflammation in patients with asthma: a systematic review and meta-analysis of interventional and observational studies |
| El Abd, A., H. Dasari, P. Dodin, H. Trottier and F. M. Ducharme | 2024 | The effects of vitamin D supplementation on inflammatory biomarkers in patients with asthma: a systematic review and meta-analysis of randomized controlled trials |
| El Abd, A., H. Dasari, P. Dodin, H. Trottier and F. M. Ducharme | 2024 | The effects of vitamin D supplementation on inflammatory biomarkers in patients with asthma: a systematic review and meta-analysis of randomized controlled trials |
| Fares, M. M., L. H. Alkhaled, S. M. Mroueh and E. A. Akl | 2015 | Vitamin D supplementation in children with asthma: a systematic review and meta-analysis |
| Fedora, K., R. A. Setyoningrum, Q. Aina, L. N. Rosyidah, N. L. Ni'mah and F. F. Titiharja | 2024 | Vitamin D supplementation decrease asthma exacerbations in children: a systematic review and meta-analysis of randomized controlled trials |
| Fedora, K., R. A. Setyoningrum, Q. Aina, L. N. Rosyidah, N. L. Ni'mah and F. F. Titiharja | 2024 | Vitamin D supplementation decrease asthma exacerbations in children: a systematic review and meta-analysis of randomized controlled trials |
| Feng, H., P. Xun, K. Pike, A. K. Wills, B. L. Chawes, H. Bisgaard, W. Cai, Y. Wan and K. He | 2017 | In utero exposure to 25-hydroxyvitamin D and risk of childhood asthma, wheeze, and respiratory tract infections: A meta-analysis of birth cohort studies |
| Feng, H. X., P. C. Xun, K. Pike, A. K. Wills, B. L. Chawes, H. Bisgaard, W. Cai, Y. P. Wan and K. He | 2017 | In utero exposure to 25-hydroxyvitamin D and risk of childhood asthma, wheeze, and respiratory tract infections: A meta-analysis of birth cohort studies |
| Garcia-Larsen, V., S. R. Del Giacco, A. Moreira, M. Bonini, D. Charles, T. Reeves, K. H. Carlsen, T. Haahtela, S. Bonini, J. Fonseca, I. Agache, N. G. Papadopoulos and L. Delgado | 2016 | Asthma and dietary intake: an overview of systematic reviews |
| Glabska, D., A. Kolota, K. Lachowicz, D. Skolmowska, M. Stachon and D. Guzek | 2023 | Supplementation of Vitamin D and Mental Health in Adults with Respiratory System Diseases: A Systematic Review of Randomized Controlled Trials |
| Głąbska, D., A. Kołota, K. Lachowicz, D. Skolmowska, M. Stachoń and D. Guzek | 2023 | Supplementation of Vitamin D and Mental Health in Adults with Respiratory System Diseases: A Systematic Review of Randomized Controlled Trials |
| Grandinetti, R., V. Fainardi, C. Caffarelli, G. Capoferri, A. Lazzara, M. Tornesello, A. Meoli, B. M. Bergamini, L. Bertelli, L. Biserna, P. Bottau, E. Corinaldesi, N. De Paulis, A. Dondi, B. Guidi, F. Lombardi, M. S. Magistrali, E. Marastoni, S. Pastorelli, A. Piccorossi, M. Poloni, S. Tagliati, F. Vaienti, G. Gregori, R. Sacchetti, S. Mari, M. Musetti, F. Antodaro, A. Bergomi, L. Reggiani, F. Caramelli, A. De Fanti, F. Marchetti, G. Ricci, S. Esposito and E. R. A. S. G. Emilia-Romagna Asthma | 2022 | Risk Factors Affecting Development and Persistence of Preschool Wheezing: Consensus Document of the Emilia-Romagna Asthma (ERA) Study Group |
| Grant, W. B. | 2012 | RE: "ASTHMA AND CARIES: A SYSTEMATIC REVIEW AND META-ANALYSIS" |
| Griffith, R. J., J. Alsweiler, A. E. Moore, S. Brown, P. Middleton, E. Shepherd and C. A. Crowther | 2020 | Interventions to prevent women from developing gestational diabetes mellitus: an overview of Cochrane Reviews |
| Griffith, R. J., J. Alsweiler, A. E. Moore, S. Brown, P. Middleton, E. Shepherd and C. A. Crowther | 2020 | Interventions to prevent women from developing gestational diabetes mellitus: an overview of Cochrane Reviews |
| Han, J. C., J. Du, Y. J. Zhang, G. B. Qi, H. B. Li, Y. J. Zhang and X. L. Yu | 2016 | Vitamin D receptor polymorphisms may contribute to asthma risk |
| Hao, M. Q., R. X. Xu, N. C. Luo, M. W. Liu, J. P. Xie and W. X. Zhang | 2022 | The Effect of Vitamin D Supplementation in Children With Asthma: A Meta-Analysis |
| Harvey, N., C. Holroyd, G. Ntani, K. Javaid, P. Cooper, R. Moon, Z. Cole, T. Tinati, N. Bishop, K. Godfrey, E. Dennison, J. Baird and C. Cooper | 2013 | Maternal pregnancy vitamin D status and offspring bone health: A systematic review and meta-analysis |
| Harvey, N. C., C. Holroyd, G. Ntani, K. Javaid, P. Cooper, R. Moon, Z. Cole, T. Tinati, K. Godfrey, E. Dennison, N. J. Bishop, J. Baird and C. Cooper | 2014 | Vitamin D supplementation in pregnancy: a systematic review |
| He, L., X. G. Zhou, H. Mo, X. M. Li and S. L. Guo | 2022 | The association between vitamin D receptor gene polymorphisms and asthma: a systematic review and meta-analysis |
| Jain, N., R. Varman, J. A. Tarbox and T. Nguyen | 2021 | Biomolecular endotype factors involved in COVID-19 airway infectivity: A systematic review |
| Jain, R. P., D. Als, T. Vaivada and Z. A. Bhutta | 2022 | Prevention and Management of High-Burden Noncommunicable Diseases in School-Age Children: A Systematic Review |
| Jolliffe, D. A., C. A. Camargo, J. D. Sluyter, M. Aglipay, J. F. Aloia, D. Ganmaa, P. Bergman, H. A. Bischoff-Ferrari, A. Borzutzky, C. T. Damsgaard, G. Dubnov-Raz, S. Esposito, C. Gilham, A. A. Ginde, I. Golan-Tripto, E. C. Goodall, C. C. Grant, C. J. Griffiths, A. M. Hibbs, W. Janssens, A. V. Khadilkar, I. Laaksi, M. T. Lee, M. Loeb, J. L. Maguire, P. Majak, D. T. Mauger, S. Manaseki-Holland, D. R. Murdoch, A. Nakashima, R. E. Neale, H. Pham, C. Rake, J. R. Rees, J. Rosendahl, R. Scragg, D. Shah, Y. Shimizu, S. Simpson-Yap, G. Trilok-Kumar, M. Urashima and A. R. Martineau | 2021 | Vitamin D supplementation to prevent acute respiratory infections: a systematic review and meta-analysis of aggregate data from randomised controlled trials |
| Jolliffe, D. A., L. Greenberg and R. L. Hooper | 2018 | Vitamin D supplementation to prevent asthma exacerbations: a systematic review and meta-analysis of individual participant data (vol 5, pg 881, 2017) |
| Jolliffe, D. A., L. Greenberg, R. L. Hooper, C. J. Griffiths, C. A. Camargo, Jr., C. P. Kerley, M. E. Jensen, D. Mauger, I. Stelmach, M. Urashima and A. R. Martineau | 2017 | Vitamin D supplementation to prevent asthma exacerbations: a systematic review and meta-analysis of individual participant data |
| Kim, J. H., J. Y. Kim, J. Lee, G. H. Jeong, E. Lee, S. Lee, K. H. Lee, A. Kronbichler, B. Stubbs, M. Solmi, A. Koyanagi, S. H. Hong, E. Dragioti, L. Jacob, A. R. Brunoni, A. F. Carvalho, J. Radua, T. Thompson, L. Smith, H. Oh, L. Yang, I. Grabovac, F. Schuch, M. Fornaro, A. Stickley, T. B. Rais, G. S. de Pablo, J. I. Shin and P. Fusar-Poli | 2020 | Environmental risk factors, protective factors, and peripheral biomarkers for ADHD: an umbrella review |
| Kim, Y. H., K. W. Kim, M. J. Kim, I. S. Sol, S. H. Yoon, H. S. Ahn, H. J. Kim, M. H. Sohn and K. E. Kim | 2016 | Vitamin D levels in allergic rhinitis: a systematic review and meta-analysis |
| Kim, Y. H., K. W. Kim, M. J. Kim, I. S. Sol, S. H. Yoon, H. S. Ahn, H. J. Kim, M. H. Sohn and K. E. Kim | 2016 | Vitamin D levels in allergic rhinitis: a systematic review and meta-analysis |
| Kohn, C. M. and P. Paudyal | 2017 | A systematic review and meta-analysis of complementary and alternative medicine in asthma |
| Kumar, J., P. Kumar, J. P. Goyal, C. Thakur, P. Choudhary, J. Meena, J. Charan, K. Singh and A. Gupta | 2022 | Vitamin D supplementation in childhood asthma: a systematic review and meta-analysis of randomised controlled trials |
| Ladeira, J., O. Zacas, A. M. Ferreira, P. C. Gomes Stegun, M. B. Grotta and A. Toro | 2022 | The role of vitamin D in the severity and control of asthma in children and adolescents: A protocol for systematic review and meta-analysis |
| Ladeira, J., O. Zacas, A. M. Ferreira, P. C. G. Stegun, M. B. Grotta and A. Toro | 2022 | The role of vitamin D in the severity and control of asthma in children and adolescents: A protocol for systematic review and meta-analysis |
| Li, J., A. Tiwari, H. Mirzakhani, A. L. Wang, A. T. Kho, M. J. McGeachie, A. A. Litonjua, S. T. Weiss and K. G. Tantisira | 2021 | Circulating MicroRNA: Incident Asthma Prediction and Vitamin D Effect Modification |
| Li, Q., X. S. Xu, Y. Liu, S. Yin, Q. Hu, Q. Ji, Y. Zhong and F. Y. Zhu | 2025 | The effects of prenatal vitamin D supplementation on respiratory and allergy-related outcomes in children: A systematic review and meta-analysis of randomized controlled trials |
| Li, Q., Q. Zhou, G. Zhang, X. Tian, Y. Li, Z. Wang, Y. Zhao, Y. Chen and Z. Luo | 2022 | Vitamin D Supplementation and Allergic Diseases during Childhood: A Systematic Review and Meta-Analysis |
| Li, Q. Y., Q. Zhou, G. L. Zhang, X. Y. Tian, Y. Y. Li, Z. L. Wang, Y. Zhao, Y. L. Chen and Z. X. Luo | 2022 | Vitamin D Supplementation and Allergic Diseases during Childhood: A Systematic Review and Meta-Analysis |
| Li, W., Z. Qin, J. Gao, Z. Jiang, Y. Chai, L. Guan, Y. Ge and Y. Chen | 2019 | Vitamin D supplementation during pregnancy and the risk of wheezing in offspring: a systematic review and dose-response meta-analysis |
| Li, W., Z. Qin, J. Gao, Z. B. Jiang, Y. H. Chai, L. C. Guan, Y. H. Ge and Y. Z. Chen | 2019 | Vitamin D supplementation during pregnancy and the risk of wheezing in offspring: a systematic review and dose-response meta-analysis |
| Linseisen, J. | 2023 | Update on extra-skeletal health effects of vitamin D - implications for recommendations |
| Litonjua, A. A. | 2019 | Vitamin D and childhood asthma: causation and contribution to disease activity |
| Litonjua, A. A. | 2019 | Vitamin D and childhood asthma: causation and contribution to disease activity |
| Liu, J., Y. Q. Dong, J. Yin, J. Yao, J. Shen, G. J. Sheng, K. Li, H. F. Lv, X. Fang and W. F. Wu | 2019 | Meta-analysis of vitamin D and lung function in patients with asthma |
| Luo, C., Y. N. Sun, Z. J. Zeng, Y. Liu and S. L. Peng | 2022 | Vitamin D supplementation in pregnant women or infants for preventing allergic diseases: a systematic review and meta-analysis of randomized controlled trials |
| Luo, J., D. Liu and C. T. Liu | 2015 | Can Vitamin D Supplementation in Addition to Asthma Controllers Improve Clinical Outcomes in Patients With Asthma? A Meta-Analysis |
| Luo, J., D. Liu and C. T. Liu | 2015 | Can Vitamin D Supplementation in Addition to Asthma Controllers Improve Clinical Outcomes in Patients With Asthma?: A Meta-Analysis |
| Luo, J. A., D. Liu and C. T. Liu | 2016 | Can Vitamin D Supplementation in Addition to Asthma Controllers Decrease Asthmatic Exacerbations in Patients With Asthma? A Meta-analysis |
| Machado, M. E., L. C. Porto, M. G. A. Galvao, C. C. Sant'Anna and J. Silva | 2023 | SNPs, adipokynes and adiposity in children with asthma |
| Makoui, M. H., D. Imani, M. Motallebnezhad, M. Azimi and B. Razi | 2020 | Vitamin D receptor gene polymorphism and susceptibility to asthma Meta-analysis based on 17 case-control studies |
| Malden, S., J. Gillespie, A. Hughes, A. M. Gibson, A. Farooq, A. Martin, C. Summerbell and J. J. Reilly | 2021 | Obesity in young children and its relationship with diagnosis of asthma, vitamin D deficiency, iron deficiency, specific allergies and flat-footedness: A systematic review and meta-analysis |
| Malliaraki, N., K. Lakiotaki, R. Vamvoukaki, G. Notas, I. Tsamardinos, M. Kampa and E. Castanas | 2020 | Translating vitamin D transcriptomics to clinical evidence: Analysis of data in asthma and chronic obstructive pulmonary disease, followed by clinical data meta-analysis |
| Malliaraki, N., K. Lakiotaki, R. Vamvoukaki, G. Notas, I. Tsamardinos, M. Kampa and E. Castanas | 2020 | Translating vitamin D transcriptomics to clinical evidence: Analysis of data in asthma and chronic obstructive pulmonary disease, followed by clinical data meta-analysis |
| Man, L. X., Z. Zhang, M. Zhang, Y. Y. Zhang, J. Li, N. Zheng, Y. H. Cao, M. Chi, Y. J. Chao, Q. Huang, C. M. Song and B. Xu | 2015 | Association between vitamin D deficiency and insufficiency and the risk of childhood asthma: evidence from a meta-analysis |
| Maretzke, F., A. Bechthold, S. Egert, J. B. Ernst, D. M. van Lent, S. Pilz, J. Reichrath, G. I. Stangl, P. Stehle, D. Volkert, M. Wagner, J. Waizenegger, A. Zittermann, J. Linseisen and D. G. E. German Nutr Soc | 2020 | Role of Vitamin D in Preventing and Treating Selected Extraskeletal Diseases-An Umbrella Review |
| Martineau, A. R., C. J. Cates, M. Urashima, M. Jensen, A. P. Griffiths, U. Nurmatov, A. Sheikh and C. J. Griffiths | 2016 | Vitamin D for the management of asthma |
| Martineau, A. R., C. J. Cates, M. Urashima, M. Jensen, A. P. Griffiths, U. Nurmatov, A. Sheikh and C. J. Griffiths | 2016 | Vitamin D for the management of asthma: Cochrane systematic review and meta-analysis |
| Martineau, A. R., C. J. Cates, M. Urashima, M. Jensen, A. P. Griffiths, U. Nurmatov, A. Sheikh and C. J. Griffiths | 2016 | Vitamin D for the management of asthma: Cochrane systematic review and meta-analysis |
| Mateussi, M. V., C. D. C. Latorraca, J. P. Daou, A. L. C. Martimbianco, R. Riera, R. L. Pacheco and D. V. Pachito | 2017 | What do Cochrane systematic reviews say about interventions for vitamin D supplementation? |
| Nelson, H. S. | 2018 | Immunotherapy for house-dust mite allergy |
| Netting, M. J., P. F. Middleton and M. Makrides | 2014 | Does maternal diet during pregnancy and lactation affect outcomes in offspring? A systematic review of food-based approaches |
| Nitzan, I., F. B. Mimouni, A. Bin Nun, Y. Kasirer and J. Mendlovic | 2022 | Vitamin D and Asthma: a Systematic Review of Clinical Trials |
| Nitzan, I., F. B. Mimouni, A. B. Nun, Y. Kasirer and J. Mendlovic | 2022 | Vitamin D and Asthma: a Systematic Review of Clinical Trials |
| Niu, H. Y., H. J. He, Z. L. Zhao, X. M. Lu and G. Zhao | 2024 | Asthmatic patients with vitamin D deficiency: Can vitamin D supplementation make a difference |
| Nurmatov, U., G. Devereux and A. Sheikh | 2011 | Nutrients and foods for the primary prevention of asthma and allergy: systematic review and meta-analysis |
| Nurmatov, U., G. Devereux and A. Sheikh | 2011 | Nutrients and foods for the primary prevention of asthma and allergy: Systematic review and meta-analysis |
| Pacheco-González, R. M., L. García-Marcos and E. Morales | 2018 | Prenatal vitamin D status and respiratory and allergic outcomes in childhood: A meta-analysis of observational studies |
| Patchen, B. K., C. M. Best, J. Boiteau, B. S. Solvik, A. Vonderschmidt, J. Xu, R. T. Cohen and P. A. Cassano | 2025 | Vitamin D supplementation in pregnant or breastfeeding women or young children for preventing asthma |
| Patchen, B. K., C. M. Best, J. Boiteau, B. S. Solvik, A. Vonderschmidt, J. Xu, R. T. Cohen and P. A. Cassano | 2025 | Vitamin D supplementation in pregnant or breastfeeding women or young children for preventing asthma |
| Pfeffer, P. E. and C. M. Hawrylowicz | 2018 | Vitamin D in Asthma Mechanisms of Action and Considerations for Clinical Trials |
| Pfeffer, P. E. and C. M. Hawrylowicz | 2018 | Vitamin D in Asthma: Mechanisms of Action and Considerations for Clinical Trials |
| Pojsupap, S., K. Iliriani, T. Sampaio, K. O'Hearn, T. Kovesi, K. Menon and J. D. McNally | 2015 | Efficacy of high-dose vitamin D in pediatric asthma: a systematic review and meta-analysis |
| Pojsupap, S., K. Iliriani, T. Z. Sampaio, K. O'Hearn, T. Kovesi, K. Menon and J. D. McNally | 2015 | Efficacy of high-dose vitamin D in pediatric asthma: a systematic review and meta-analysis |
| Pullar, J., K. Wickramasinghe, A. R. Demaio, N. Roberts, K. M. Perez-Blanco, K. Noonan and N. Townsend | 2019 | The impact of maternal nutrition on offspring's risk of non-communicable diseases in adulthood: a systematic review |
| Rajabbik, M. H., T. Lotfi, L. Alkhaled, M. Fares, G. El-Hajj Fuleihan, S. Mroueh and E. A. Akl | 2014 | Association between low vitamin D levels and the diagnosis of asthma in children: a systematic review of cohort studies |
| Reinehr, T., D. Schnabel, M. Wabitsch, S. B. D. Pozzalla, C. Bührer, B. Heidtmann, F. Jochum, T. Kauth, A. Körner, W. Mihatsch, C. Prell, S. Rudloff, B. Tittel, J. Wölfle, K. P. Zimmer, B. Koletzko and K. Deutschen Gesellschaft | 2018 | Vitamin D supplementation beyond the second year of life. Joint statement of the Nutrition Committee of the German Society for Pediatric and Adolescent Medicine (DGKJ) and the German Society for Pediatric Endocrinology and Diabetology (DGKED) |
| Rice, J. B., A. G. White, L. M. Scarpati, G. Wan and W. W. Nelson | 2017 | Long-term Systemic Corticosteroid Exposure: A Systematic Literature Review |
| Riverin, B. D., J. L. Maguire and P. Li | 2015 | Vitamin D Supplementation for Childhood Asthma: A Systematic Review and Meta-Analysis |
| Riverin, B. D., J. L. Maguire and P. Li | 2015 | Vitamin D Supplementation for Childhood Asthma: A Systematic Review and Meta-Analysis |
| Robison, R. and R. Kumar | 2010 | The effect of prenatal and postnatal dietary exposures on childhood development of atopic disease |
| Rosser, F. J., M. L. Yue, Y. Y. Han, E. Forno, C. Qoyawayma, M. L. Manni, E. Acosta-Pérez, G. Canino, W. Chen and J. C. Celedón | 2025 | Long-Term PM2.5 Exposure and Upregulation of CLCA1 Expression in Nasal Epithelium from Youth with Asthma |
| Saag, K. G. | 2003 | Glucocorticoid-induced osteoporosis |
| Salameh, L., W. Mahmood, R. Hamoudi, K. Almazrouei, M. Lochanan, S. Seyhoglu and B. Mahboub | 2023 | The Role of Vitamin D Supplementation on Airway Remodeling in Asthma: A Systematic Review |
| Salameh, L., W. Mahmood, R. Hamoudi, K. Almazrouei, M. Lochanan, S. Seyhoglu and B. Mahboub | 2023 | The Role of Vitamin D Supplementation on Airway Remodeling in Asthma: A Systematic Review |
| Shen, S. Y., W. Q. Xiao, J. H. Lu, M. Y. Yuan, J. R. He, H. M. Xia, X. Qiu, K. K. Cheng and K. B. H. Lam | 2018 | Early life vitamin D status and asthma and wheeze: a systematic review and meta-analysis |
| Shen, S. Y., W. Q. Xiao, J. H. Lu, M. Y. Yuan, J. R. He, H. M. Xia, X. Qiu, K. K. Cheng and K. B. H. Lam | 2018 | Early life vitamin D status and asthma and wheeze: a systematic review and meta-analysis |
| Shi, D., D. Wang, Y. Meng, J. Chen, G. Mu and W. Chen | 2021 | Maternal vitamin D intake during pregnancy and risk of asthma and wheeze in children: a systematic review and meta-analysis of observational studies |
| Shi, D., D. M. Wang, Y. D. Meng, J. H. Chen, G. Mu and W. H. Chen | 2021 | Maternal vitamin D intake during pregnancy and risk of asthma and wheeze in children: a systematic review and meta-analysis of observational studies |
| Sobczak, M. and R. Pawliczak | 2023 | Relationship between vitamin D and asthma from gestational to adulthood period: a meta-analysis of randomized clinical trials |
| Sobczak, M. and R. Pawliczak | 2023 | Relationship between vitamin D and asthma from gestational to adulthood period: a meta-analysis of randomized clinical trials |
| Sobczak, M. and R. Pawliczak | 2023 | Relationship between vitamin D and asthma from gestational to adulthood period: a meta-analysis of randomized clinical trials |
| Song, H., L. Yang and C. Jia | 2017 | Maternal vitamin D status during pregnancy and risk of childhood asthma: A meta-analysis of prospective studies |
| Song, H. H., L. Yang and C. Q. Jia | 2017 | Maternal vitamin D status during pregnancy and risk of childhood asthma: A meta-analysis of prospective studies |
| Tareke, A. A., A. A. Hadgu, A. M. Ayana and T. A. Zerfu | 2020 | Prenatal vitamin D supplementation and child respiratory health: A systematic review and meta-analysis of randomized controlled trials |
| TePoel, M. R. W., A. F. Saftlas and A. B. Wallis | 2011 | Association of seasonality with hypertension in pregnancy: a systematic review |
| Thorsteinsdottir, F., K. C. Walker, S. E. Runstedt, R. Jacobsen, E. Maslova, V. Backer, B. L. Heitmann and M. N. Händel | 2022 | The role of prenatal vitamin D on the development of childhood asthma and wheeze: An umbrella review of systematic reviews and meta-analyses |
| Thorsteinsdottir, F., K. C. Walker, S. E. Runstedt, R. Jacobsen, E. Maslova, V. Backer, B. L. Heitmann and M. N. Händel | 2022 | The role of prenatal vitamin D on the development of childhood asthma and wheeze: An umbrella review of systematic reviews and meta-analyses |
| Tizaoui, K., A. Berraies, B. Hamdi, W. Kaabachi, K. Hamzaoui and A. Hamzaoui | 2014 | Association of Vitamin D Receptor Gene Polymorphisms with Asthma Risk: Systematic Review and Updated Meta-analysis of Case-Control Studies |
| Tong, X. L., X. Y. Zhang, M. Y. Wang, Z. J. Wang, F. W. Dong, E. Y. Gong, T. Zuberbier and Y. M. Li | 2024 | Non-pharmacological interventions for asthma prevention and management across the life course: Umbrella review |
| Vahdaninia, M., H. Mackenzie, S. Helps and T. Dean | 2017 | Prenatal Intake of Vitamins and Allergic Outcomes in the Offspring: A Systematic Review and Meta-Analysis |
| van Brakel, L., R. P. Mensink, G. Wesseling and J. Plat | 2020 | Nutritional Interventions to Improve Asthma-Related Outcomes through Immunomodulation: A Systematic Review |
| Venter, C., C. Agostoni, S. H. Arshad, M. Ben-Abdallah, G. Du Toit, D. M. Fleischer, M. Greenhawt, D. H. Glueck, M. Groetch, N. Lunjani, K. Maslin, A. Maiorella, R. Meyer, M. Antonella, M. J. Netting, B. I. Nwaru, D. J. Palmer, M. P. Palumbo, G. Roberts, C. Roduit, P. Smith, E. Untersmayr, L. A. Vanderlinden and L. O'Mahony | 2020 | Dietary factors during pregnancy and atopic outcomes in childhood: A systematic review from the European Academy of Allergy and Clinical Immunology |
| Visser, E., A. ten Brinke, D. Sizoo, J. J. S. Pepels, L. ten Have, E. V. Wiel, T. van Zutphen, H. A. M. Kerstjens and K. de Jong | 2024 | Effect of dietary interventions on markers of type 2 inflammation in asthma: A systematic review |
| Walker, K. C., F. Thorsteinsdottir, H. T. Christesen, V. E. Hjortdal, B. L. Heitmann, I. O. Specht and M. N. Händel | 2023 | Vitamin D Supplementation and Vitamin D Status during Pregnancy and the Risk of Congenital Anomalies-A Systematic Review and Meta-Analysis |
| Wang, M., M. Liu, C. Wang, Y. Xiao, T. An, M. Zou and G. Cheng | 2019 | Association between vitamin D status and asthma control: A meta-analysis of randomized trials |
| Wang, M. M., M. C. Liu, C. R. Wang, Y. Xiao, T. An, M. J. Zou and G. Cheng | 2019 | Association between vitamin D status and asthma control: A meta-analysis of randomized trials |
| Wang, Q., Q. Ying, W. Zhu and J. Chen | 2022 | Vitamin D and asthma occurrence in children: A systematic review and meta-analysis |
| Wang, Q., Q. L. Ying, W. Zhu and J. G. Chen | 2022 | Vitamin D and asthma occurrence in children: A systematic review and meta-analysis |
| Wang, S. M., P. P. Yin, L. L. Yu, F. W. Tian, W. Chen and Q. X. Zhai | 2024 | Effects of Early Diet on the Prevalence of Allergic Disease in Children: A Systematic Review and Meta-Analysis |
| Wang, Y., J. Wang, L. Chen, H. Zhang, L. Yu, Y. Chi, M. Chen and Y. Cai | 2022 | Efficacy of vitamin D supplementation on COPD and asthma control: A systematic review and meta-analysis |
| Wang, Y. H., J. Wang, L. Chen, H. Zhang, L. Yu, Y. L. Chi, M. L. Chen and Y. Cai | 2022 | Efficacy of vitamin D supplementation on COPD and asthma control: A systematic review and meta-analysis |
| Wei, Z., J. Zhang and X. Yu | 2016 | Maternal vitamin D status and childhood asthma, wheeze, and eczema: A systematic review and meta-analysis |
| Wei, Z. Z., J. Zhang and X. D. Yu | 2016 | Maternal vitamin D status and childhood asthma, wheeze, and eczema: A systematic review and meta-analysis |
| Williamson, A., A. R. Martineau, A. Sheikh, D. Jolliffe and C. J. Griffiths | 2023 | Vitamin D for the management of asthma |
| Williamson, A., A. R. Martineau, A. Sheikh, D. Jolliffe and C. J. Griffiths | 2023 | Vitamin D for the management of asthma |
| Williamson, A., A. R. Martineau, A. Sheikh, D. Jolliffe and C. J. Griffiths | 2023 | Vitamin D for the management of asthma |
| Williamson, A. E., C. J. Griffiths, A. Sheikh and A. R. Martineau | 2022 | UPDATED COCHRANE SYSTEMATIC REVIEW: NO EVIDENCE THAT VITAMIN D REDUCES ASTHMA EXACERBATIONS OR IMPROVES ASTHMA CONTROL |
| Wolsk, H. M., B. L. Chawes, A. A. Litonjua, B. W. Hollis, J. Waage, J. Stokholm, K. Bonnelykke, H. Bisgaard and S. T. Weiss | 2017 | Prenatal vitamin D supplementation reduces risk of asthma/recurrent wheeze in early childhood: A combined analysis of two randomized controlled trials |
| Xiao, L. M., C. Xing, Z. R. Yang, S. J. Xu, M. Wang, H. R. Du, K. Liu and Z. H. Huang | 2015 | Vitamin D supplementation for the prevention of childhood acute respiratory infections: a systematic review of randomised controlled trials |
| Yepes-Nuñez, J. J., J. L. Brozek, A. Fiocchi, R. Pawankar, C. Cuello-García, Y. Zhang, G. P. Morgano, A. Agarwal, S. Gandhi, L. Terracciano and H. J. Schünemann | 2018 | Vitamin D supplementation in primary allergy prevention: Systematic review of randomized and non-randomized studies |
| Yepes-Nuñez, J. J., A. Fiocchi, R. Pawankar, C. A. Cuello-Garcia, Y. Zhang, G. P. Morgano, K. Ahn, S. Al-Hammadi, A. Agarwal, S. Gandhi, K. Beyer, W. Burks, G. W. Canonica, M. Ebisawa, R. Kamenwa, B. W. Lee, H. Q. Li, S. Prescott, J. J. Riva, L. Rosenwasser, H. Sampson, M. Spigler, L. Terracciano, A. Vereda, S. Waserman, H. J. Schünemann and J. L. Brozek | 2016 | World Allergy Organization-McMaster University Guidelines for Allergic Disease Prevention (GLAD-P): Vitamin D |
| Zhang, L., S. N. Zhang, C. He and X. H. Wang | 2020 | VDR Gene Polymorphisms and Allergic Diseases: Evidence from a Meta-analysis |
| Zhang, L. L., J. Gong and C. T. Liu | 2014 | Vitamin D with asthma and COPD: not a false hope? A systematic review and meta-analysis |
| Zhang, L. L., J. Gong and C. T. Liu | 2014 | Vitamin D with asthma and COPD: not a false hope? A systematic review and meta-analysis |
| Zhao, D. D., D. D. Yu, Q. Q. Ren, B. Dong, F. Zhao and Y. H. Sun | 2017 | Association of vitamin D receptor gene polymorphisms with susceptibility to childhood asthma: A meta-analysis |
| Zhou, Y. and S. Li | 2022 | Meta-Analysis of Vitamin D Receptor Gene Polymorphisms in Childhood Asthma |
| 陈慧, 林晓霞 and 陈燕惠. | 2017 | 孕期摄入维生素D对婴幼儿哮喘发生率影响的系统回顾和荟萃分析. 2017丝路国际儿童健康高峰论坛暨《中国儿童保健杂志》第五届编委换届会议论文集. 西安: 344-345. |
| 陈慧, 林晓霞 and 陈燕惠 | 2018 | 孕期摄入维生素D对婴幼儿哮喘发生率影响的系统回顾和荟萃分析 |
| 陈慧, 林晓霞 and 陈燕惠 | 2018 | 孕期摄入维生素D对婴幼儿哮喘发生率影响的系统回顾和荟萃分析 |
| 陈沁竹. | 2015 | 维生素A补充对哮喘、过敏反应及肺功能影响的meta分析 硕士, 重庆医科大学. |
| 陈沁竹 and 符州. | 2015 | 维生素A补充对哮喘、过敏反应及肺功能影响的meta分析. 中华医学会第二十次全国儿科学术大会论文集. 厦门: 138-138. |
| 陈沁竹 and 徐王国 | 2020 | 维生素A补充与变应性疾病相关性的Meta分析 |
| 杜梦思, 宋露露, 李星, 于斐 and 王艺琳 | 2023 | 血清维生素D水平与儿童哮喘风险的meta分析 |
| 杜梦思, 宋露露, 李星, 于斐 and 王艺琳 | 2023 | 血清维生素D水平与儿童哮喘风险的meta分析 |
| 郝畅. | 2019 | 维生素D补充与儿童哮喘的系统评价及meta分析 硕士, 重庆医科大学. |
| 郝畅, 应林燕 and 符州 | 2019 | 维生素D补充与儿童哮喘的系统评价及meta分析 |
| 郝畅, 应林燕 and 符州 | 2019 | 维生素D补充与儿童哮喘的系统评价及meta分析 |
| 郝宏霞. | 2018 | 维生素D在缓解期哮喘患者治疗中的有效性和安全性meta分析 硕士, 山西医科大学. |
| 郝宏霞 and 田新瑞 | 2017 | 维生素D在缓解期哮喘患者治疗中的有效性和安全性Meta分析 |
| 郝宏霞 and 田新瑞 | 2017 | 维生素D在缓解期哮喘患者治疗中的有效性和安全性Meta分析 |
| 胡冀生. | 2024 | 孕期维生素D水平与子代过敏性疾病发生风险的Meta分析 硕士, 河北医科大学. |
| 景伟超, 刘璐佳, 关洋洋 and 王有鹏 | 2017 | 维生素D辅助治疗儿童哮喘Meta分析 |
| 李晓娅, 王小妮 and 陆小霞 | 2016 | 维生素D与儿童哮喘相关性的Meta分析 |
| 李晓娅, 王小妮 and 陆小霞 | 2016 | 维生素D与儿童哮喘相关性的Meta分析 |
| 刘瑞芳, 张蓉芳, 李桂荣, 雷苗 and 高文龙 | 2019 | 维生素D受体基因多态性与哮喘相关性meta分析 |
| 邱戌旦. | 2014 | 维生素D受体基因多态性与哮喘相关性的Meta分析 硕士, 浙江大学. |
| 孙倩. | 2024 | 补充维生素D及其类似物对支气管哮喘的影响：一项随机对照试验的荟萃分析 硕士, 南昌大学医学部. |
| 田超, 史强, 赵紫楠, 孟瑶 and 王晓玲 | 2018 | 维生素D补充剂对儿童支气管哮喘获益相关性的系统评价和meta分析 |
| 田超, 史强, 赵紫楠, 孟瑶 and 王晓玲 | 2018 | 维生素D补充剂对儿童支气管哮喘获益相关性的系统评价和meta分析 |
| 王晓丽, 荆玉兰 and 牛焕红 | 2020 | 孕期摄入维生素D预防婴幼儿哮喘的meta分析 |
| 王晓丽, 荆玉兰 and 牛焕红 | 2020 | 孕期摄入维生素D预防婴幼儿哮喘的meta分析 |
| 吴军, 张平, 周波, 匡嘉丽 and 郭亮 | 2009 | 变应性鼻炎、哮喘与饮食非过敏因素的循证医学相关研究 |
| 姚应水. | 2015 | 哮喘患病的环境因素系统评价 博士, 安徽理工大学. |
| 于少飞, 冯万禹, 柴少卿 and 朱华. | 2017 | 25羟维生素D水平与儿童哮喘关系的Meta分析. 中华医学会第二十二次全国儿科学术大会论文集. 苏州: 1802-1802. |
| 余其梅, 林慧, 叶方立 and 周婷 | 2019 | 血清维生素D水平与成人哮喘严重程度关系的Meta分析 |
| 余其梅, 林慧, 叶方立 and 周婷 | 2019 | 血清维生素D水平与成人哮喘严重程度关系的Meta分析 |
| 员笑笑. | 2018 | 血清中维生素D水平与成人支气管哮喘关系的Meta分析 硕士, 山西医科大学. |
| 员笑笑, 吴世满 and 张红红 | 2018 | 维生素D水平与成人支气管哮喘关系的Meta分析 |
| 员笑笑, 吴世满 and 张红红 | 2018 | 维生素D水平与成人支气管哮喘关系的Meta分析 |

7 **Review**

|  | 2000 | 2000年第9卷总目录 |
| --- | --- | --- |
|  | 2001 | 心血管康复医学杂志2000年第9卷主题索引 |
| Rapid vs Maintenance Vitamin D Supplementation in Deficient Children With Asthma to Prevent Exacerbations. | 2011 | Rapid vs Maintenance Vitamin D Supplementation in Deficient Children With Asthma to Prevent Exacerbations. |
|  | 2018 | Corrections: Vitamin D supplementation to prevent asthma exacerbations: a systematic review and meta-analysis of individual participant data (The Lancet Respiratory Medicine (2017) 5(11) (881–890)(S2213260017303065)(10.1016/S2213-2600(17)30306-5)) |
| Alsharairi, N. A. | 2023 | Antioxidant Intake and Biomarkers of Asthma in Relation to Smoking Status—A Review |
| Ames, B. N., W. B. Grant and W. C. Willett | 2021 | Does the High Prevalence of Vitamin D Deficiency in African Americans Contribute to Health Disparities? |
| Andújar-Espinosa, R. and L. Salinero-González | 2021 | Vitamin D Supplementation: A Treatment With Possible Benefits in Asthma |
| Argano, C., A. Torres, V. Orlando, V. Cangialosi, D. Maggio, C. Pollicino and S. Corrao | 2025 | Molecular Insight into the Role of Vitamin D in Immune-Mediated Inflammatory Diseases |
| Awadh, A. A., D. E. Hilleman, E. Knezevich, M. A. Malesker and J. C. Gallagher | 2021 | Vitamin D supplements: The pharmacists' perspective |
| Barua, P. and M. S. O'Mahony | 2005 | Overcoming gaps in the management of asthma in older patients: New insights |
| Best, C. M., J. Xu, B. K. Patchen and P. A. Cassano | 2019 | Vitamin d supplementation in pregnant or breastfeeding women or young children for preventing asthma |
| Bischoff-Ferrari, H. A. | 2011 | Vitamin D - Role in Pregnancy and Early Childhood |
| Boot, E., S. Óskarsdóttir, J. C. Y. Loo, T. B. Crowley, A. Orchanian-Cheff, D. M. Andrade, J. M. Arganbright, R. M. Castelein, C. Cserti-Gazdewich, S. de Reuver, A. M. Fiksinski, G. Klingberg, A. E. Lang, M. R. Mascarenhas, E. M. Moss, B. A. Nowakowska, E. Oechslin, L. Palmer, G. M. Repetto, N. G. D. Reyes, M. Schneider, C. Silversides, K. E. Sullivan, A. Swillen, T. A. M. J. van Amelsvoort, J. P. Van Batavia, C. Vingerhoets, D. M. McDonald-McGinn and A. S. Bassett | 2023 | Updated clinical practice recommendations for managing adults with 22q11.2 deletion syndrome |
| Braegger, C., C. Campoy, V. Colomb, T. Decsi, M. Domellof, M. Fewtrell, I. Hojsak, W. Mihatsch, C. Molgaard, R. Shamir, D. Turck and J. Van Goudoever | 2013 | Vitamin d in the healthy European paediatric population |
| Buendía, J. A., C. E. Rodriguez-Martinez and M. P. Sossa-Briceño | 2023 | Cost utility of vitamin D supplementation in adults with mild to moderate asthma |
| Calogiuri, G., L. H. Garvey, E. Nettis, F. Casciaro, S. Al-Sowaidi, C. Foti and A. Vacca | 2021 | Hypersensitivity to vitamins with a focus on immediate-type reactions: Food or drug allergy? |
| Cannell, J. J. and B. W. Hollis | 2008 | Use of vitamin D in clinical practice |
| Cediel, G., J. Pacheco-Acosta and C. Castillo-Durán | 2018 | Vitamin D deficiency in pediatric clinical practice |
| Chalmers, J. D. and S. H. Chotirmall | 2018 | Bronchiectasis: new therapies and new perspectives |
| Chaudhary, U., A. Zain Ul and S. S. Hassan Gardezi | 2025 | Letter to the editor regarding “vitamin D supplementation decrease asthma exacerbations in children: a systematic review and meta-analysis of randomized controlled trials” |
| Davidson, B. L. | 2018 | Administration of placebo vitamin D to non-consenting children |
| Di Mauro, G., R. Bernardini, S. Barberi, A. Capuano, A. Correra, G. L. De'Angelis, I. D. Iacono, M. De Martino, D. Ghiglioni, D. Di Mauro, M. Giovannini, M. Landi, G. L. Marseglia, A. Martelli, V. L. Miniello, D. Peroni, L. R. M. G. Sullo, L. Terracciano, C. Vascone, E. Verduci, M. C. Verga and E. Chiappini | 2016 | Prevention of food and airway allergy: Consensus of the Italian Society of Preventive and Social Paediatrics, the Italian Society of Paediatric Allergy and Immunology, and Italian Society of Pediatrics |
| Dyussenova, S. B., M. Y. Gordiyenko, M. S. Askarov, G. K. Tuleuova, S. B. Suleimenova, G. S. Khussainova and B. I. Abisheva | 2021 | The role of Vitamin D in respiratory viral infections and other infectious diseases |
| Eigenmann, P. | 2018 | Editorial comments on this issue of the Journal |
| Entrenas-Castillo, M., L. Salinero-González, L. M. Entrenas-Costa and R. Andújar-Espinosa | 2022 | Calcifediol for Use in Treatment of Respiratory Disease |
| Fournier, J., L. Barret, C. Khouri, F. Naudet, R. Boussageon and M. Roustit | 2024 | The evidence base of the 10 most prescribed drugs in England, France, and the United States: a scoping review |
| Gaudet, M., M. Plesa, A. Mogas, N. Jalaleddine, Q. Hamid and S. Al Heialy | 2022 | Recent advances in vitamin D implications in chronic respiratory diseases |
| Goyal, J. P. | 2024 | Vitamin D and Respiratory Diseases |
[truncated: 16,462 more chars]
